# Phylogenetic evidence of allopatric speciation of bradyrhizobia nodulating cowpea (*Vigna unguiculata* L. walp) in South African and Mozambican soils

**DOI:** 10.1093/femsec/fiz067

**Published:** 2019-05-16

**Authors:** Mamadou Dabo, Sanjay K Jaiswal, Felix D Dakora

**Affiliations:** 1 Department of Crop Sciences, Tshwane University of Technology, Private Bag X680, Pretoria 0001, South Africa; 2 Chemistry Department, Tshwane University of Technology, Private Bag X680, Pretoria 0001, South Africa

**Keywords:** IAA, phosphate solubilisation, housekeeping genes, phylogeny, horizontal gene transfer, CCA

## Abstract

The legume host and soil environment play a major role in establishing effective symbiosis with diverse rhizobia for plant growth promotion and nodule formation. The aim of this study was to assess the morpho-physiology, distribution and phylogenetic position of rhizobia nodulating cowpea from South Africa and Mozambique. The results showed that the isolates were highly diverse in their appearance on yeast mannitol agar plates. The isolates tested also showed an ability to produce IAA at concentrations ranging from 0.64 to 56.46 μg.ml^−1^ and to solubilise phosphorus at levels from 0 to 3.55 index. Canonical correspondence analysis showed that soil pH and mineral nutrients significantly influenced bradyrhizobial distribution. Analysis of BOX-PCR placed the isolates in eight major clusters with 0.01 to 1.00 similarity coefficient which resulted in 45 unique BOX-types. Phylogenetic analyses based on 16S rRNA, *atpD*, *glnII, gyrB* and *recA* gene sequences showed distinct novel evolutionary lineages within the genus *Bradyrhizobium*, with some of them being closely related to *Bradyrhizobium kavangense*, *B. subterraneum* and *B. pachyrhizi*. Furthermore, symbiotic gene phylogenies suggested that the isolates’ *sym* loci probably relates to the isolates’ geographical origin. The results indicated that geographical origin did affect the isolates’ phylogenetic placement and could be the basis for allopatric speciation

## Teaser

Morpho-physiology and phylogeny of bradyrhizobia

## INTRODUCTION

Legumes are a major component of agricultural systems throughout the world due to their N_2_-fixing ability when in symbiosis with soil bacteria called rhizobia. This makes nodulated legumes good candidates for improving soil fertility, especially nitrogen (N) (Mohale, Belane and Dakora 2013). On a global scale, symbiotic bacteria contribute the largest amount of N through BNF to terrestrial ecosystems, and are thus vital for agricultural production (Unkovich and Pate 2000; Echevarria-Zomeño *et al*. 2009). N_2_ fixation is usually performed by the bacteria in root nodules, and the NH_3_ produced is exchanged with the host plant for photosynthate (Prell and Poole 2006). The distinctive feature of the legume symbiosis is the formation of root or stem nodules following a series of molecular exchanges between the microsymbiont and the host plant (Chidebe, Jaiswal and Dakora 2018).

Cowpea is one of the most important indigenous legume crops in Africa, with both wild and cultivated forms growing across the continent (Mohammed, Jaiswal and Dakora 2018). West Africa is the centre of origin of this crop (Jaiswal and Dakora 2019; Puozaa, Jaiswal and Dakora 2019). Cowpea can meet 66% to 96% of its N nutrition from symbiotic fixation (Jaiswal and Dakora 2019).

The high cost of N fertilizers and the environmental pollution associated with their frequent use to increase grain legume production can be overcome through the use of highly effective rhizobia that can efficiently meet the crop's N demand in a sustainable and environmentally friendly manner. In Africa, the most common factor limiting legume productivity is low availability of mineral nutrients, especially N and phosphorus (P) (Dakora and Keya 1997). Plants tend to perform better in the presence of sufficient numbers of effective soil rhizobia that can fix atmospheric N_2_, solubilize soil P, and produce plant growth-promoting compounds such as indole acetic acid (IAA) which promote root growth and water nutrient uptake.

Plant growth-promoting bacteria can therefore influence plant development directly by synthesizing root-growth hormones thus facilitating the supply and uptake of mineral nutrients from the soil. Solubilization of P compounds in soil by rhizobia is an important strategy for enhancing plant growth and productivity (Marra *et al*. 2012). Although P is an important macronutrient for plant growth, about 95% to 99% of soil P occurs in the insoluble form that cannot be utilized by plants (Vassilev *et al*. 2001). Some rhizobial bacteria are however capable of solubilizing unavailable soil P complexes for plant uptake and development, just as some rhizobia are also capable of producing IAA, a hormone that is involved in root formation and root elongation for increased uptake of water and nutrients (Etesami *et al*. 2009). Thus, the identification of IAA-producing and P-solubilizing traits among N_2_-fixing rhizobia is important for selecting rhizobia for inoculant production.

A robust rhizobial taxonomy and phylogeny is key to identifying microsymbionts for improving legume productivity using biological N_2_ fixation (Chidebe, Jaiswal and Dakora 2018). Such studies have increased our understanding of the genetic diversity of rhizobia and significantly improved rhizobial classification through the re-definition of species. Previous reports from the different parts of the world such as Botswana, Ghana, South Africa, Senegal, Mozambique, Ethiopia, Greece, Spain and Brazil have identified *Bradyrhizobium* as the main microsymbiont nodulating cowpea (Pule-meulenberg *et al*. 2010; Bejarano *et al*. 2014; Wade *et al*. 2014; Degefu *et al*. 2017; Marinho *et al*. 2017; Tampakaki *et al*. 2017; Chidebe, Jaiswal and Dakora 2018; Mohammed, Jaiswal and Dakora 2018; Jaiswal and Dakora 2019; Puozaa, Jaiswal and Dakora 2019). However, knowledge gaps still exist in the genetic diversity and biogeographic distribution of indigenous N_2_-fixing rhizobia in Southern Africa. Since cowpea is an indigenous legume crop of the vast and agro-ecologically divergent African continent, identification and characterization of native rhizobia nodulating cowpea cultivated in different geographic regions of Africa will help to understand and unravel the factors shaping rhizobial diversity and distribution in African soils. Therefore, the aim of this study was to describe the morpho-physiological and molecular characteristics of rhizobial isolates nodulating cowpea from different ecological environments as well as to assess these bacteria for P solubilisation and IAA production.

## MATERIALS AND METHODS

### Field experimentation, nodule collection and isolation

**Figure 1.**
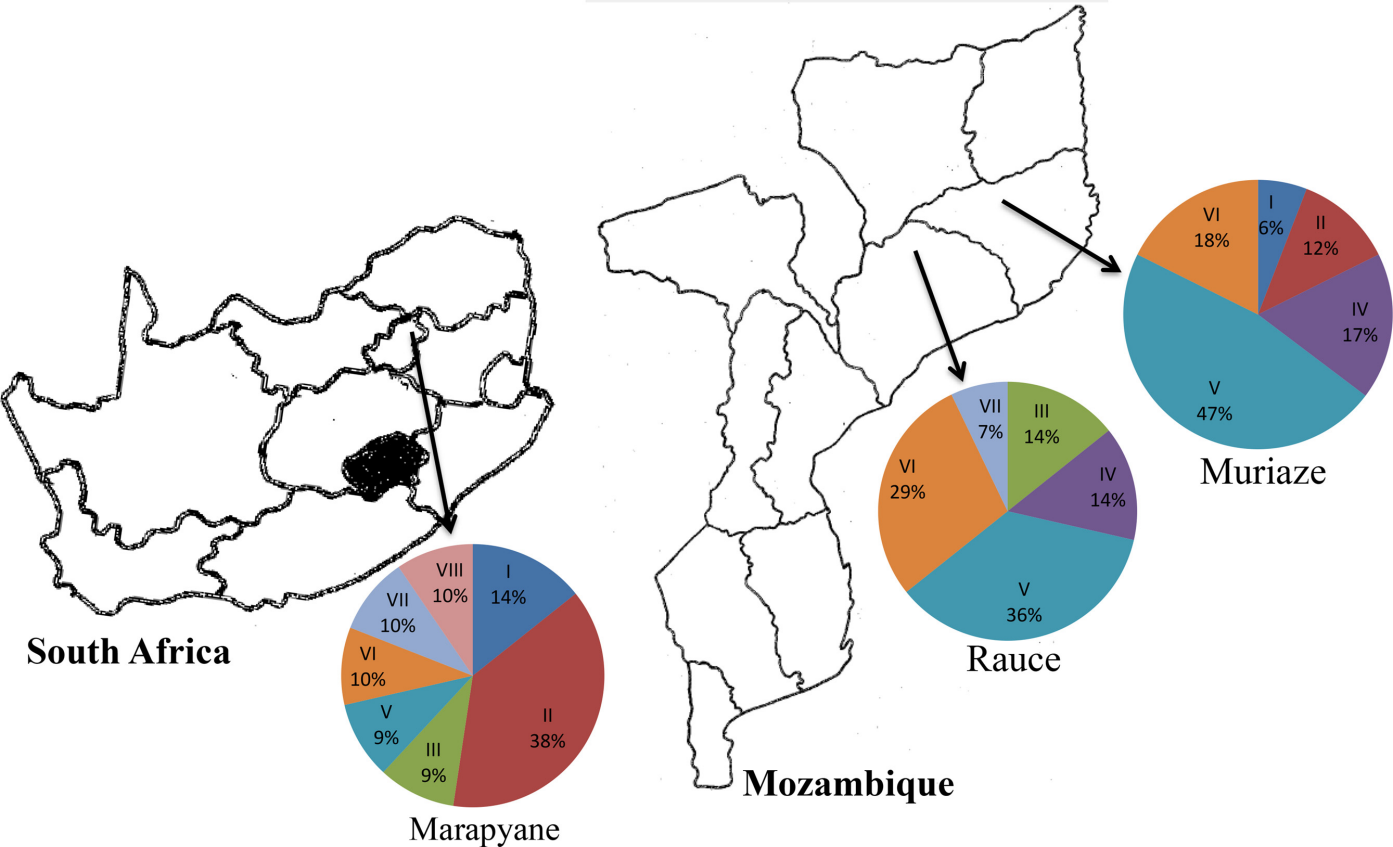
Test locations in South Africa and Mozambique, and rhizobial distribution in different clusters based on BOX-PCR analysis.

Nodules were collected from cowpea genotypes grown in the field at Marapyane in Nkangala District, west of the Mpumalanga Province of South Africa and at Ruace and Muriaze of Mozambique. Marapyane is located at the geographical coordinates of 24° 57ʹ 60ʺ S, 28° 45ʹ 76ʺ E and elevation 1022 meters above sea level, while Ruace and Muriaze are located at 15 ^o^ 14ʹ 17.5ʹʹ S, 26^o^ 43ʹ 44.8ʹʹ E and 15^o^ 09ʹ 12.9ʹʹ S, 30^o^ 19ʹ 20ʹʹ E geographical coordinates, respectively (Fig. 1). The test sites had been under cultivation of various cereals and grain legumes for the past five years. Before planting cowpea, soil samples were collected at a depth of 0–30 cm for physico-chemical analysis (Table S1, Supporting Information). The experiment was laid out as a randomised complete block design with three replications. Six and five cowpea genotypes were used to trap microsymbionts in the fields of South Africa and Mozambique, respectively (Table   1). There were three common cowpea genotypes (IT97K-1069–6, IT00K-126–3, and IT18) grown in the test locations of both South Africa and Mozambique (Table   1). Each experimental plot measured 1.2 × 3 m^2^. There were four rows in each plot, with 1.2 m row length. Seeds were planted with an inter-row spacing of 75 cm and intra-row spacing of 15 cm.

**Table 1:** Colony types, biochemical and molecular characterization of cowpea-nodulating isolates.

			Colony characteristic					Biochemical characteristic		Box-PCR Type
Isolate	Location	Genotype	Appearance day	Size	Texture	Shape	colour	IAA µg ml−1	PSI	
South Africa										
TUTVUMp5	Marapyane	IT97K-1069–6	8	1.5	Gummy	Irregular	Translucent	26.38±0.05	1.43	1
TUTVUMp6	Marapyane	IT97K-390–2	4	1	Gummy	Conical	Whitish	2.16±0.05	2.72	30
TUTVUMp7	Marapyane	IT97K-390–2	7	3	Gummy	Domed	Milky	15.60±0.05	2	5
TUTVUMp8	Marapyane	IT00K-126–3	6	2	Dry	Irregular	Whitish	19.28±0.5	2.13	33
TUTVUMp11	Marapyane	IT06K-128	5	1	Dry	Domed	Translucent	17.25±3	3.33	5
TUTVUMp12	Marapyane	IT00K-126–3	7	2	Gummy	Irregular	Milky	26.77±0.05	1.47	5
TUTVUMp26	Marapyane	IT00K-126–3	8	1.5	Dry	Conical	Whitish	13.42±1	1.47	5
TUTVUMp28	Marapyane	Sudan 1	7	2	Gummy	Watery	Translucent	28.01±2.5	1.27	5
TUTVUMp37	Marapyane	IT00K-126–3	8	4.5	Gummy	Irregular	Milky	8.89±1.5	1.62	2
TUTVUMp41	Marapyane	IT97K-390–2	6	4	Gummy	Watery	Translucent	31.55±0.005	1.67	5
TUTVUMp48	Marapyane	IT18	9	2	Gummy	Domed	Whitish	35.02±3	0	16
TUTVUMp49	Marapyane	IT97K-390–2	4	1	Gummy	Irregular	Translucent	44.63±3	1.54	22
TUTVUMp50	Marapyane	Sudan 1	7	2.5	Gummy	Irregular	Milky	20.85±2	1.55	5
TUTVUMp53	Marapyane	IT18	8	6	Gummy	Domed	Whitish	39.21±5	2.5	7
TUTVUMp56	Marapyane	Sudan 1	8	2	Gummy	Irregular	Milky	17.89±4.5	1.76	6
TUTVUMp57	Marapyane	IT18	4	6	Gummy	Watery	Translucent	41.54±1.5	2.5	8
TUTVUMp58	Marapyane	Sudan 1	6	3	Gummy	Irregular	Translucent	41.88±0.5	2.16	35
TUTVUMp59	Marapyane	Sudan 1	3	6	Gummy	Irregular	Milky	35.02±3	1.68	3
TUTVUMp60	Marapyane	IT97K-1069–6	5	4	Elastic	Irregular	Milky	8.25±3	0	32
TUTVUMp65	Marapyane	IT97K-1069–6	8	2	Gummy	Domed	Whitish	26.22±1	2	17
TUTVUMp75	Marapyane	IT97K-1069–6	4	2.5	Dry	Irregular	Translucent	35.48±0.5	2.9	25
Mozambique										
TUTVUMr80	Ruace	IT00K-126–3	6	3.5	Gummy	Domed	Whitish	1.01±2	3.55	18
TUTVUMr82	Ruace	IT00K-126–3	5	3	Gummy	Irregular	Translucent	37±4	0	20
TUTVUMr83	Ruace	IT97K-1069–6	7	1	Dry	Conical	Whitish	40±2	2	19
TUTVUMr84	Ruace	IT97K-1069–6	6	1	Dry	Conical	Milky	14.32±2	0	29
TUTVUMr85	Ruace	IT97K-1069–6	6	2	Gummy	Domed	Whitish	29.59±5.5	0	23
TUTVUMr89	Ruace	IT18	10	0.5	Dry	Circular	Whitish	2.88±2	2.08	24
TUTVUMr90	Ruace	IT18	3	6	Gummy	Domed	Milky	22.22±3	1.22	11
TUTVUMr93	Ruace	IT97K-1069–6	3	3	Gummy	Domed	Milky	0.64±0.1	1.38	9
TUTVUMr94	Ruace	IT97K-1069–6	6	4.5	Gummy	Irregular	Whitish	4.29±0.05	0	31
TUTVUMr95	Ruace	IT97K-1069–6	3	6	Elastic	Irregular	Milky	6.926±3.5	0	27
TUTVUMr99	Ruace	IT97K-1069–6	6	6	Gummy	Irregular	Milky	5.7±1	0	10
TUTVUMr100	Ruace	IT97K-1069–6	8	1	Dry	Conical	Whitish	4.20±0.05	2.5	26
TUTVUMr101	Ruace	IT18	7	3	Gummy	Flat	Whitish	2.58±0.05	2.33	19
TUTVUMr102	Ruace	IT00K-126–3	3	6	Elastic	Domed	Milky	46.38±1.5	1.6	12
TUTVUMr103	Ruace	IT16	4	6	Gummy	Irregular	Milky	1.94±1.5	0	21
TUTVUMm104	Muriaze	IT97K-1069–6	10	1	Dry	Conical	Whitish	34.98±3	1.81	19
TUTVUMm105	Muriaze	IT97K-1069–6	9	2	Gummy	Irregular	Whitish	40.76±2.5	0	19
TUTVUMm106	Muriaze	IT97K-1069–6	5	2	Elastic	Domed	Whitish	4.49±0.05	0	19
TUTVUMm107	Muriaze	IT97K-1069–6	7	3	Gummy	Domed	Whitish	46.73±0.05	1.83	19
TUTVUMm108	Muriaze	IT97K-1069–6	3	6	Elastic	Irregular	Milky	24.63±0.05	0	6
TUTVUMm109	Muriaze	IT97K-1069–6	6	4	Gummy	Irregular	Milky	21.67±2	2.36	6
TUTVUMm110	Muriaze	IT97K-1069–6	6	2.5	Gummy	Irregular	Milky	40.45±0.05	2.18	22
TUTVUMm111	Muriaze	IT97K-1069–6	7	5	Gummy	Watery	Translucent	35.04±0.05	4	22
TUTVUMm112	Muriaze	IT16	6	3	Elastic	Domed	Whitish	1.07±0.05	1.5	28
TUTVUMm113	Muriaze	IT16	7	2.5	Gummy	Domed	Whitish	46.01±1	0	13
TUTVUMm114	Muriaze	IT16	5	2	Gummy	Domed	Whitish	22.89±1.5	0	14
TUTVUMm115	Muriaze	IT18	6	2	Gummy	Domed	Whitish	42.19±1.5	0	15
TUTVUMm116	Muriaze	IT04K-227–4	6	2.5	Gummy	Domed	Whitish	26.56±2.5	0	19
TUTVUMm117	Muriaze	IT04K-227–4	6	5	Gummy	Irregular	Milky	20.13±1.5	0	4
TUTVUMm118	Muriaze	IT97K-1069–6	5	3	Gummy	Domed	Milky	50.12±0.01	1.57	19
TUTVUMm119	Muriaze	IT16	4	6	Gummy	Domed	Milky	56.46±1.5	1.71	19
TUTVUMm120	Muriaze	IT97K-1069–6	5	4.5	Gummy	Irregular	Milky	52.56±4	1.47	19

At 50 days after planting, plants were carefully dug out from the Marapyane site (South Africa) and from two farmers’ fields at Ruace and Muriaze (Mozambique) (Fig. 1), and transported to the laboratory. The roots with attached nodules were separated and washed in running tap water to remove adhered soil. Healthy, unbroken and firm root nodules were selected and detached from the root using forceps and scissors and stored in vials containing silica gel at 4°C until bacterial isolation. During isolation, the nodules were hydrated in water, and surface-sterilized by immersion in 75% ethanol for 1 minute, then washed in 3.5% NaOCl for 3 minutes, followed by rinsing five times with sterile distilled water. Each surface-sterilized nodule was then crushed in a loop of sterile distilled water in a sterile petri dish, and the nodule macerate streaked on yeast mannitol agar (YMA) plates and incubated at 28°C. Colony appearance was observed from two to twelve days after incubation, and colony characteristics such as diameter, shape, colour and texture were recorded on YMA plates after 15 days of incubation for each isolate.

### Nodulation test with isolates

To fulfil Koch's postulates, the single-colony bacterial isolates were examined for their ability to form root nodules on cowpea, their homologous host plant. Healthy cowpea seeds were surface-sterilized by immersion in 75% alcohol for 10 seconds. The alcohol was drained, and the seeds placed in 3% sodium hypochlorite for 1 to 2 minutes. The seeds were then rinsed six times with sterile distilled water and two seeds planted in sterile autoclaved sand contained in sterile pots (5006.05 cm^3^) using a randomised complete block design with three replications under glasshouse conditions. After germination, the seedlings were thinned to one plant per pot. Six-day-old seedlings were inoculated with 1 mL exponential phase-grown bacterial suspension (∼ 10^6^–10^7^ cells/ml) using sterile micropipettes. Uninoculated, plants were included as control. The test seedlings were supplied with sterile N-free nutrient solution (Broughton and Dilworth 1971) twice a week and sterile distilled water when necessary. At 8 weeks after planting, the plants were harvested, and nodulation data recorded. Deep green leaf colour and pink nodules were considered as an indication of effective nodulation.

### Biochemical characterization of rhizobia

Two biochemical properties were assessed, namely, the production of the root growth-promoting hormone auxin (indole-3-acetic acid) and P-solubilization. Production of IAA by rhizobial cells was estimated, as described by Raddadi et al (Raddadi *et al*. 2008). Standard curves were generated from absorbances of 0.5,10,10,20,50 and 100 µg mL^−1^ IAA measured at 530 nm (Fig. S1, Supporting Information).

The ability of rhizobial isolates to solubilize P was assessed by the Double-layered plate method (Yanni *et al*. 2001). At the exponential growth stage, 10 µL rhizobial culture was spotted onto double-layered agar medium plate and incubated at 28°C for 7 days. The formation of a clear halo zone around a bacterial spot was taken as indicative of P solubilisation. The diameter of each halo zone was measured to identify isolates having high P-solubilising activity. Phosphate-solubilizing capacity was calculated in terms of phosphate solubilization index (PSI) as PSI = A/B, where A is the total diameter of the halo zone, and B is the colony diameter. The isolates showing PSI ≥ 2 were considered as high phosphate-solubilizing bacteria (Rahman *et al*. 2014).

### DNA extraction and BOX-PCR fingerprinting of bacterial isolates

Bacterial genomic DNA was extracted using Sigma's Bacterial Genomic DNA Kit according to the manufacturer's instructions (GenElute^TM^), and used for rep-PCR (BOX- PCR) with a Box-A1R primer (Table S2, Supporting Information). The final volume of the PCR reaction mixture was 25 µL and contained 1 µL (50–70 ng µL^−1^) genomic DNA, 12.5 µL MyTaq PCR master-mix (2x), 1µL (10 µM) BOX-A1R primer, and 10.5 mL sterile ultrapure water. PCR amplification was done in a Thermal cycler (T100 BIORAD, USA) following standard temperature profiles (Table S2, Supporting Information). The PCR-amplified products were electrophoresed in 1.2% agarose gel for 6 hours, visualized and photographed using gel documentation system (BIO-RAD Gel Doc^TM^ XR+). The sizes of bands were determined using the Image Lab software (Bio-Rad version 4.1). The band pattern was recorded in a binary form (1, 0) and cluster analysis was carried out with the UPGMA (Unweighted Pair Group Method with Arithmetic mean) algorithm using the NtSyspc 2.1 software (Rohlf FJ, 2009).

### PCR amplification of the 16S rRNA, housekeeping (*atpD, glnII, gyrB, recA*) and symbiotic (*nifH* and *nodC*) genes

PCR amplification of 16S rRNA, protein-coding (*atpD, glnII, gyrB* and *recA*) and symbiotic (*nifH* and *nodC*) genes were each done in 25 µL reaction volume. The reaction mixture contained 1 µL DNA (50–70 ng µL^−1^), 3 µL of myTaq buffer (5x), 1 µL (10 µM) each of forward and reverse primers of the gene of interest, 0.1 µL Taq polymerase (5U) (Bioline, USA) and 18.9 µL double distilled ultrapure water. The PCR process was carried out with Bio-Rad T100 thermal cycler using standard temperature profiles **(**Table S2, Supporting Information). The amplified gene products were confirmed by gel electrophoresis in 1.2% agarose gel stained with ethidium bromide in 1X TAE buffer at 85V for 1 hour.

### Sequencing and phylogenetic analysis

For sequencing, the amplified PCR products were purified using PCR Cleanup kit (NEB, USA) according to the manufacturer's instruction. The purified amplified DNA was sent to Macrogen (Netherlands) for sequencing. Thereafter, the quality of sequences was verified using the software BioEdit 7.0.9.0 (Hall 1999). The BLASTn program was used to identify closely related species in the NCBI database. Pairwise and multiple sequence alignments were done with CLUSTALW, and phylogenetic trees constructed by means of the maximum likelihood statistical method using MEGA 7 software (Kumar, Stecher and Tamura 2016). The robustness of branching was estimated using 1000 bootstrap replicates (Felsenstein 1985). The obtained sequences were deposited in the NCBI GenBank to get the accession numbers which were indicated in phylogenetic tress. The test gene sequences of the isolates used for phylogenetic tree constructions are shown in Figure S2 (Supporting Information).

### Statistical analysis

The effect of soil factors on the distribution of cowpea-nodulating bradyrhizobia was examined using canonical correspondence analysis (CCA) with vegan (version 2.4–2) (Oksanen *et al*. 2010) of R software (R Core Team 2015). Analysis was done with only the soil parameters which showed significant ecological contribution to the distribution of the test cowpea-nodulating bradyrhizobia. The general permutation test was used to assess the statistical significance of the ordination axes

## RESULTS

### Plant nodulation data

A total of 102 isolates were obtained from the cowpea nodules collected from all three study sites in South Africa and Mozambique. Out of the102 isolates, 61 could elicit nodule formation in cowpea (their homologous host) under glasshouse conditions. Isolates that produced green leaves and pink nodules were considered as effective rhizobia. Only effective nodule-forming isolates were used for further studies.

### Colony characteristics and biochemical behavior of the rhizobial isolates

Colony texture showed that 72% of the isolates were gummy, 17% dry, and 11% elastic. Colony sizes also differed, with 55% having a diameter of 2 to 4 mm, 28% a diameter of 4.5 to 6 mm, while 17% showed ≤1.5 mm in diameter. About 64% of the rhizobial isolates appeared in 6 to 10 days, while 36% took 3 to 5 days to appear on YMA plates. In terms of colony shape, 42% were irregular, 36% domed, 11% conical, 7.5% watery, 1.8% circular and 1.8% flat. Colony colour also differed, with 41% being whitish, 41% milky, and 18% translucent on YMA plates (Table 1).

### IAA production and phosphate-solubilising activities by authenticated isolates

The test rhizobial isolates varied in their ability to produce IAA, with concentrations ranging from 0.64 μg ml^−1^ to 56.46 μg ml^−1^ (Table 1). The highest IAA producer was isolate TUTVUMm119 (56.46 μg ml^−1^) and the least isolate TUTVUMr93 (0.64 μg ml^−1^) (Table 1).

Most of the isolates tested showed their ability to solubilize phosphate (Ca _3_PO_4_). The halo zone produced by P solubilization appeared within 3 days after spotting culture on the plate. Out of 53 isolates tested, 36 were able to solubilize inorganic phosphate. The phosphate solubilisation index (PSI) differed significantly (*P* < 0.05) among the isolates, with an index range of 0 to 4.0 (Table 1). The highest solubilization index was produced by the Marapyane isolates, followed by Muriaze, and then Ruace. Of the isolates selected from Marapyane for this study, 90% were found to solubilize P, with 10% lacking solubilisation ability (no halo zone formation). The selected isolates from Mozambique showed 53% solubilization, with 47% not being able to solubilize P (Table   1). The total solubilisation by the test isolates stood at 66%.

### Correlation analysis

There was a significant correlation between P solubilization and IAA production. Isolates that elicited high IAA production also showed greater P solubilization index (r = 0.187*), suggesting that greater P accumulation supported IAA production (Fig. S3, Supporting Information).

### Box-PCR fingerprinting analysis of genomic DNA

PCR amplification of the BOX region of the rhizobial genomic DNA from 54 representative isolates (including the commercial isolate TUTVUI122) resulted in distinct banding patterns that ranged from 1 to 14 fragments per DNA profile, with band sizes of 10 037 to 440 bp. Based on banding pattern results, the bacterial isolates were grouped into eight clusters (Clusters I-VIII) with 0.01 to 1.00 similarity coefficient, and yielded 45 unique BOX-fingerprints in the test rhizobial populations (Table   1; Fig. S4, Supporting Information). The rhizobial strains obtained from the three study sites were distributed across almost all the identified clusters, except for Cluster IV which consisted only of Mozambican isolates. The majority of the rhizobial isolates from the three experimental sites grouped in Cluster V. Isolates obtained from Marapyane were highly diverse and occupied seven clusters (Clusters I, II, III, V, VI, VII and VIII) while those from Muriaze and Ruace in Mozambique were distributed in five clusters within the dendrogram (Fig. S4, Supporting Information). Based on the test cowpea genotypes, variety IT97K-1069–6 attracted highly diverse microsymbionts which occupied six clusters (Clusters I, II, III, V, VI, and VII), followed by genotypes IT00K-126–3 and IT18 which attracted diverse isolates contained in five (I, II, IV, V, and VIII) and four (III, IV, V and VI) clusters, respectively (Table   1; Fig. S4, Supporting Information). Based on BOX-PCR fingerprinting, there was a 100% genomic similarity for isolates TUTVUMp7 and TUTVUMp12; isolates TUTUVMp26 and TUTVUMp28; isolates TUTVUMm108 and TUTVUMm109; isolates TUTVUMr101, TUTVUMm104, TUTVUMm105 and TUTVUMm107; as well as isolates TUTVUMm110 and TUTVUMm111, with all those isolates originating from the same site, except for isolate TUTVUMr101 (Fig. S4, Supporting Information).

### Phylogenetic positons of the test isolates

**Figure 2.**
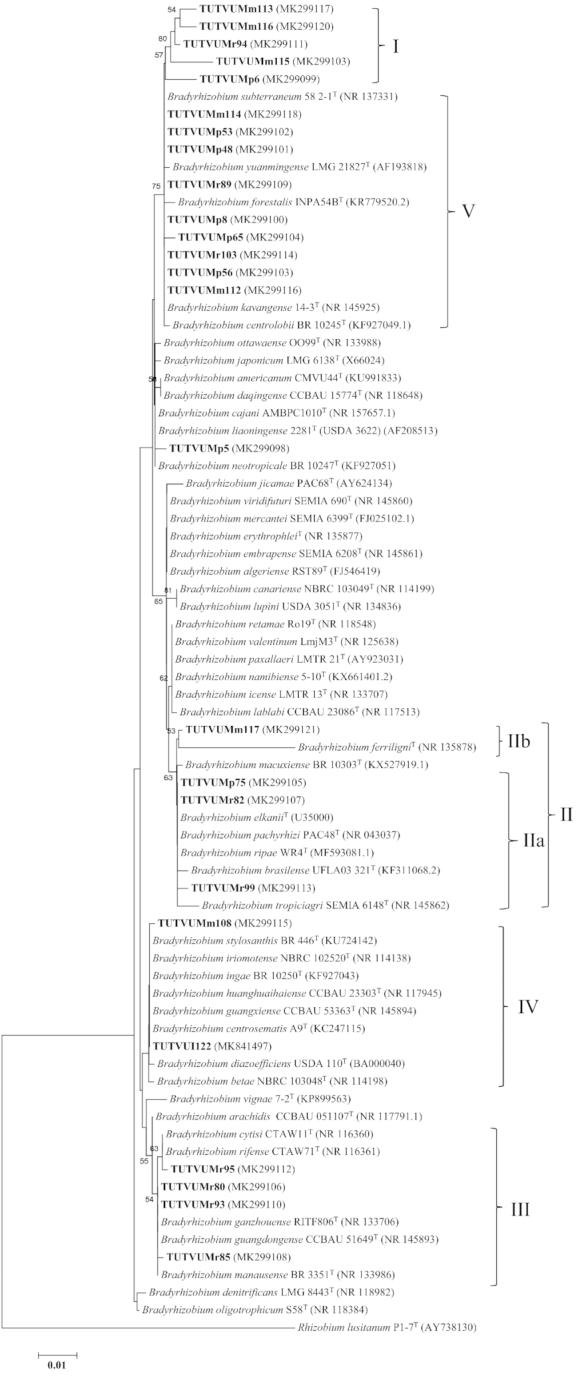
Maximum-likelihood molecular phylogenetic analysis of 16S rRNA nucleotide sequences of rhizobia nodulating cowpea collected from South Africa and Mozambique. The associated taxa clustered together in the 1000 bootstrap support and percentage are shown next to the branches. The evolutionary distances were computed using the Kimura 2-parameter method and are in the units of the number of base substitutions per site. The analysis involved 76 nucleotide sequences. Codon positions included were 1st+2nd+3rd+Noncoding. All positions containing gaps and missing data were eliminated. Evolutionary analyses were conducted in MEGA7.

Isolates were selected from each cluster of BOX-PCR dendrogram for further phylogenetic analysis. The PCR amplification of 16S rRNA gene yielded ∼1.5 kb amplicons of full length for the test isolates. The BLAST_n_ 16S rRNA sequence analysis of these isolates showed that all the strains had high sequence similarities with *Bradyrhizobium* with 73.8% conserved and 24.4% variable sequence sites (Table S2, Supporting Information). The maximum likelihood phylogeny of the 16S rRNA gene placed the test isolates into five distinct clusters (Clusters I to V) (Fig. 2). Cluster I included isolates TUTVUMm113, TUTVUMm115, TUTVUMm116, TUTVUMp6 and TUTVUMr94 which did not group with any of the reference type strains, but *B. kavangense* 14–3^T^ and *B. subterraneum* 58 2–1^T^ were the closest related species with 99.1%–99.5% sequence identity. Cluster II comprised two sub-clusters (IIa and IIb). Isolates TUTVUMp75, TUTVUMr82 and TUTVUMr99 which shared 99.7% to 100% sequence identity with *B. elkanii* USDA 76^T^ and *B. pachyrhizi* PAC48^T^ in sub-cluster IIa, and isolate TUTVUMm117 grouped with *B. ferriligni* in sub-cluster IIb. Isolates TUTVUMr80, TUTVUMr85, TUTVUMr93 and TUTVUMr95 shared 99.7%–100% identical sequences with the reference type strains of *B. ganzhouense* RITF806^T^, *B. gangdongens* CCBAU 51649^T^ and *B. manausense* BR 3351^T^ in Cluster III. Cluster IV comprised isolates TUTVUMm108 and TUTVUI122 which had a 99.8% sequence similarity with reference type strains *B. centrosematis* A9^T^, *B. guangxiense* CCBAU 23303^T^, *B. huanghuaihaiense* CCBAU 23303^T^, *B. ingae* BR 10250^T^, *B. stylosanthis* BR 446^T^ and *B. iriomotense* NBRC 102520^T^. Isolates TUTVUMp8, TUTVUMp48, TUTVUMp53, TUTVUMp56, TUTVUMp65, TUTVUMr89, TUTVUMr103, TUTVUMm112 and TUTVUMm114 recorded a 100% sequence similarity with the type reference strains of *B. kavangense* 14–3^T^, and *B. subterraneum* 58 2–1^T^ with 75% bootstrap support in Cluster V while isolate TUTVUMp5 showed outgroup of Cluster V (Fig. 2).

### Sequence and phylogenetic analysis of *atpD, glnII, gyrB* and *recA* genes

PCR-amplification of *atpD, glnII, gyrB and recA* genes, which are responsible for ATP synthase subunit beta, glutamine synthetase II, DNA gyrase subunit B and recombinase A (in that order), yielded 600, 600, 750 and 700 bp band lengths, respectively. The results of sequence analysis showed that *atpD* gene had the highest conserved sequences (63.2%) and *gyrB* gene the lowest (48.7%). The highest (33.9%) parsimony-informative sites were found in *gyrB* gene (Table S3, Supporting Information). Except for *recA* phylogeny, the topography of the individual (*atpD, glnII* and *gyrB*) housekeeping gene phylogenies were very similar and congruent with the 16S rRNA phylogeny with little inconsistency (Fig. S5–S7, Supporting Information). For example, isolates TUTVUMm110 and TUTVUMr84 grouped with *B. yuanmingense* in Cluster I of *atpD* phylogeny, with *B. arachidis* in Cluster IV of *glnII* and *gyrB* phylogenies (Fig. S5–S7, Supporting Information).

**Figure 3.**
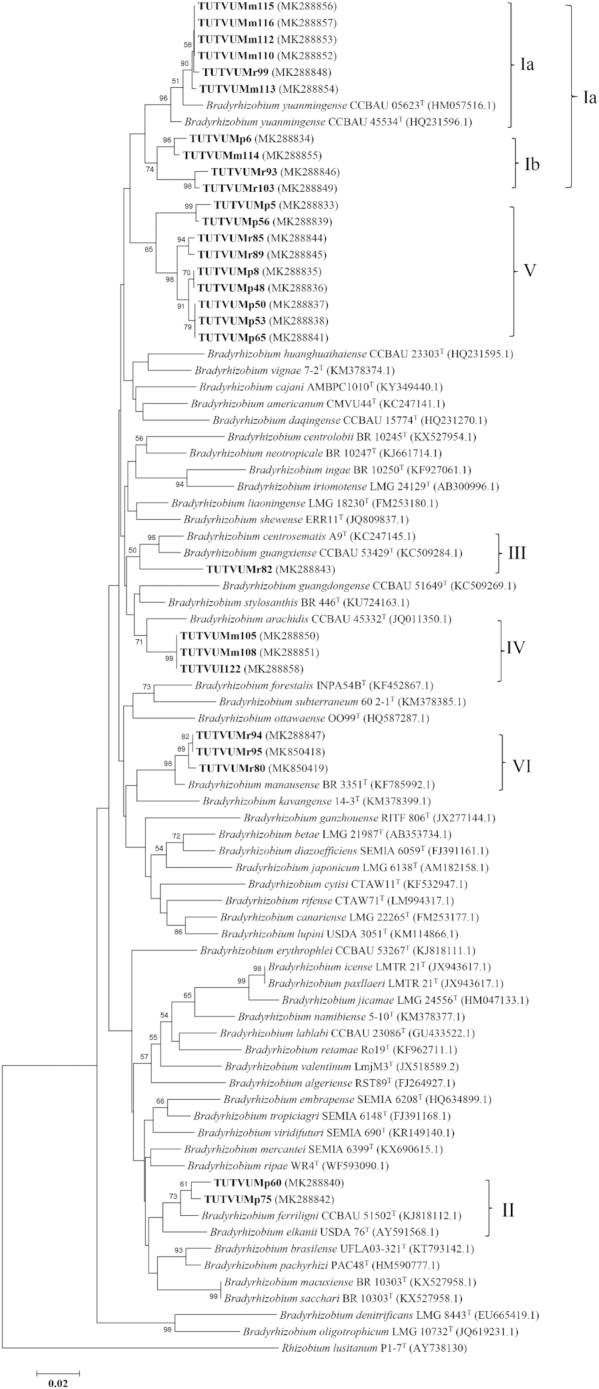
Maximum-likelihood molecular phylogenetic analysis of *recA* nucleotide sequences of rhizobia nodulating cowpea collected from South Africa and Mozambique. The associated taxa clustered together in the 1000 bootstrap support and percentage are shown next to the branches. The evolutionary distances were computed using the Kimura 2-parameter method and are in the units of the number of base substitutions per site. The analysis involved 82 nucleotide sequences. Codon positions included were 1st+2nd+3rd+Noncoding. All positions containing gaps and missing data were eliminated. Evolutionary analyses were conducted in MEGA7.

The *recA* phylogeny showed discrepancy compared to the other gene phylogenies. For example, isolates TUTVUMr99 and TUTVUMr82 grouped with *B. yuanmingense* CCBAU 05623^T^ and *B. centrosematis* A9^T^ in Cluster I and Cluster III, respectively (Fig. 3), while they showed proximal relation with the *B. elkanii* group in Cluster II of the *atpD*, *glnII* and *gyrB* phylogenies (Figs. 3, S5-S7, Supporting Information). Similarly, isolate TUTVUMm105 in Cluster IV aligned with isolates TUTVUMm108 and TUTVUI122 clustered with *B. arachidis* in *recA* phylogeny, as an outgroup while it grouped with isolate TUTVUMr94 in Cluster I in *atpD* and *gln*II phylogenies which had *B. yuanmingense* as the closest related species. In the same way, isolate TUTVUMr93 grouped with isolates of Cluster I in *recA* phylogeny, while it grouped with isolates of Cluster III in the other phylogenies (Figs. 3; S5–S7, Supporting Information).

### Concatenated gene phylogeny

**Figure 4.**
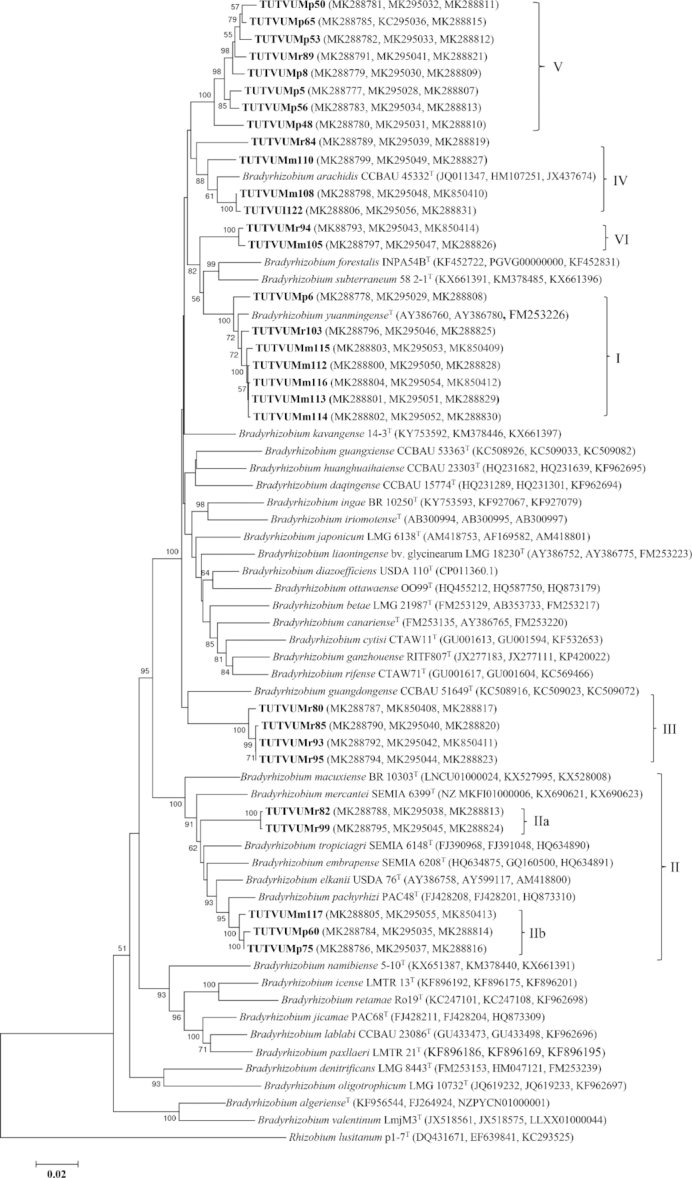
Maximum-likelihood molecular phylogenetic analysis of concatenated (*atpD*+*glnII+gyrB*) nucleotide sequences of rhizobia nodulating cowpea collected from South Africa and Mozambique. The associated taxa clustered together in the 1000 bootstrap support and percentage are shown next to the branches. The evolutionary distances were computed using the Kimura 2-parameter method and are in the units of the number of base substitutions per site. The analysis involved 67 nucleotide sequences. Codon positions included were 1st+2nd+3rd+Noncoding. All positions containing gaps and missing data were eliminated. Evolutionary analyses were conducted in MEGA7. Clusters lines in the map showed isolates belong to that locations.

If each separate gene tree represents a partial view of the information contained in the genome, all four housekeeping genes together could lead to a better understanding of the relationships between the rhizobial strains. A concatenated gene sequence analysis was therefore performed to refine the phylogenetic positions of the test isolates within the *Bradyrhizobium* lineage. A maximum likelihood phylogeny was constructed using *atpD+glnII+gyrB* concatenated aligned sequences of the test isolates and type reference strains common across the three housekeeping genes. Due to inconsistency in *recA* sequences, we did not include it in the concatenated sequence and phylogenetic analysis. The concatenated consensus sequence (1421 bp) comprised 818 conserved, 603 variables, 422 parsimonies informative and 181 singleton sites (Table S3, Supporting Information). The phylograms constructed from concatenated sequences of housekeeping genes was congruent to individual gene phylograms. Like the individual gene phylogenies, the isolates occupied five clusters in the concatenated gene tree. Isolates TUTVUMr103, TUTVUMm112, TUTVUMm113, TUTVUMm114, TUTVUMm115, TUTVUMm116 and TUTVUMp6 grouped together in Cluster I with type reference strain *B. yuanmingense* CCBAU 10071^T^ with 98.8%–99% sequence similarity and 100% bootstrap support (Fig. 4), while in the same Cluster I, isolates TUTVUMm105 and TUTVUMr94 clustered together as an outgroup and had *B. yuanmingense* as the closest related species with a low 95.8% sequence similarity. The isolates in Cluster II belonged to the *B. elkanii* group formed two sub-clusters (IIa and IIb) but with a low (<97%) sequence similarity; only isolate TUTVUMp60 showed >97% sequence identity with *B. pachyrhizi* PAC48^T^. The isolates in Cluster III, IV and V did not tightly group with any reference type strains, but they had 94%–96.8% sequence similarity with *B. kavangense* 14–3^T^ and *B. arachidis* CCBAU 45332^T^ as the closest related species (Fig. 4).

### Phylogenetic analysis based on symbiotic *nifH* and *nodC* gene sequences

**Figure 5.**
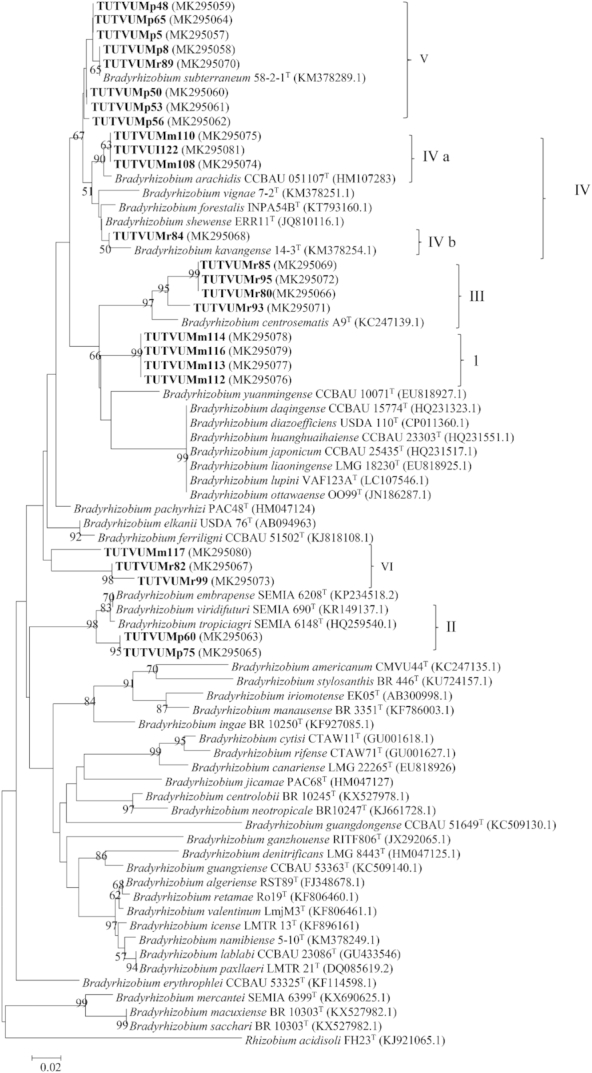
Maximum-likelihood molecular phylogenetic analysis of *nifH* nucleotide sequences of rhizobia nodulating cowpea collected from South Africa and Mozambique. The associated taxa clustered together in the 1000 bootstrap support and percentage are shown next to the branches. The evolutionary distances were computed using the Kimura 2-parameter method and are in the units of the number of base substitutions per site. The analysis involved 73 nucleotide sequences. Codon positions included were 1st+2nd+3rd+Noncoding. All positions containing gaps and missing data were eliminated. Evolutionary analyses were conducted in MEGA7.

**Figure 6.**
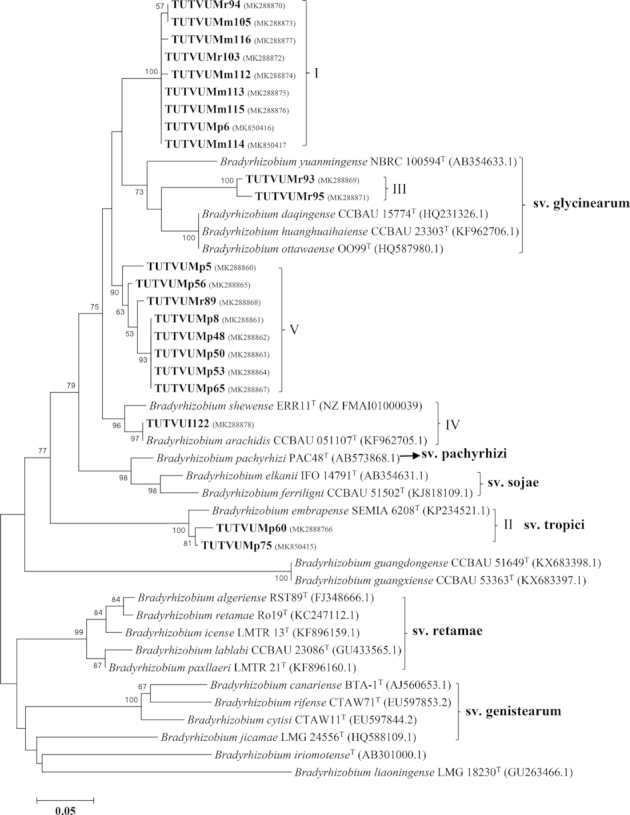
Maximum-likelihood molecular phylogenetic analysis of *nodC* nucleotide sequences of rhizobia nodulating cowpea collected from South Africa and Mozambique. The associated taxa clustered together in the 1000 bootstrap support and percentage are shown next to the branches. The evolutionary distances were computed using the Kimura 2-parameter method and are in the units of the number of base substitutions per site. The analysis involved 45 nucleotide sequences. Codon positions included were 1st+2nd+3rd+Noncoding. All positions containing gaps and missing data were eliminated. Evolutionary analyses were conducted in MEGA7.

The PCR amplification of full-length amplicons of the symbiotic *nifH* and *nodC* genes resulted in clear bands of 650 and 300 bp, respectively, as the amplified products. We did not find *nodC* amplification of all selected isolates. Therefore, we could not include all selected isolates with *nodC* sequences in the phylogenetic analysis. The non-amplification of *nodC* genomic region could be due to incompatibility of used primer pairs with the isolates’ genomes. In the maximum likelihood phylogeny, the isolates clustered into five groups (Figs   5–6) which were congruent with the phylogenies of the individual housekeeping genes, as well as with the phylogeny based on concatenated gene sequences (Figures 2  and 4; Figures S5–S7, Supporting Information).

The isolates in Cluster IV were closely related to *B. arachidis* CCBAU051107^T^ with a sequence identity of 97% in both *nifH* and *nodC* phylogenies, while isolate TUTVUMr84 in the same cluster was highly related to *B. kavangense* 14–3^T^ with 98.5% sequence identity in the *nifH* phylogeny. Isolates in Cluster V showed a close relationship with *B. subterraneum* 58–2-1^T^ with 99.5%–100% sequence similarity, but this group stood alone in the *nodC* phylogeny due to the absence of *B. subterraneum* in the tree. Isolates TUTVUMr93, TUTVUMr95, TUTVUMr80 and TUTVUMr85 grouped with *B. centrosematis* A9^T^ in Cluster III in *nifH* phylogeny, but TUTVUMr93 and TUTVUMr95 stood separately and showed proximal relation with sv. glycinearum in *nodC* phylogeny. Isolates TUTVUMp60 and TUTVUMp75 in Cluster II formed sv. tropici group in both *nifH* and *nodC* phylogenies. The isolates in Clusters I and III stood alone, and did not show close relationship with any reference type strains (Figs. 5 and 6).

### Influence of the soil environment on bradyrhizobial distribution

**Figure 7.**
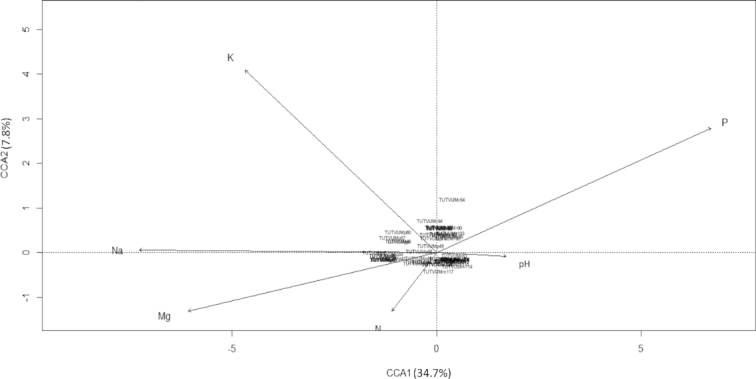
CCA plot representation of association between soil environmental variables and BOX-PCR banding pattern data of rhizobial isolates.

CCA analysis was used to correlate bradyrhizobial distribution with soil pH and soil mineral nutrients (namely, Ca, Mg, Na, N, P and K) (Fig. 7). A biplot ordination graph was constructed with only those variables which showed significant influence on bradyrhizobial distribution (*p* ≤ 0.005 at permutation 999). In the CCA plot, the total mean square contingency coefficient (inertia) was 7.29, of which 0.42 was explainable and 6.8 was unexplainable. Axes CCA1 and CCA2 were able to explain the combined 0.41 of the total explainable variation. The results of the CCA plot indicated that soil pH and Na concentration showed stronger affinity with the first canonical axis (CCA1), although the effect of Na was in the opposite direction (Fig. 7). According to the length of the arrows and angles with CCA 1 axis, Na showed stronger correlation with the axis. Soil nutrients such as K, Mg, N and P showed significant correlation (*P* ≤ 0.001) with both canonical axes (CCA1 and CCA2), as well as intermediate influence. However, soil K and N contents were orthogonal to each other (Fig. 7). From the CCA axis, the CCA biplot seemed to suggest that soil pH, as well as Na and Mg levels strongly influenced the genomic variation of isolates collected from Muriaze and Marapyane.

## DISCUSSION

Cowpea is a very important legume in southern Africa and is known to be promiscuous in its nodulation. Increased cowpea production for global food security depends on increasing and maintaining higher grain yield, which is currently not possible without major nutrient inputs, especially N and P. Cowpea-nodulating rhizobia can be preferred by farmers include those producing growth-promoting hormones such as IAA and those solubilizing phosphate complexes to increase rhizosphere P availability.

This study focused on the analysis of genetic diversity, plant growth-promoting traits and the phylogeny of rhizobia needed to define the criteria for bacterial use in inoculant production for increased cowpea production. Inorganic phosphate solubilisation and IAA production were thus explored in cowpea isolates obtained from South Africa and Mozambique. Although there are a few reports on IAA-producing microsymbionts responsible for *Vigna radiata* nodulation (Anjum *et al*. 2011; Ahmad *et al*. 2013), there is little information on IAA-producing microsymbionts that nodulate *Vigna unguiculata* L. Walp. Yet Hunter (Hunter 1989) has suggested that IAA in nodules might be involved in several stages of the legume symbiosis. The genes induced by IAA are probably responsible for the many cellular functions of plants (Abel *et al*. 1996). It has also been suggested that IAA released during rhizobial infection triggers the division of cortical cells in the roots, leading to nodule formation (Datta and Basu 2000).

In this study, most of the IAA-producing rhizobial isolates also exhibited phosphate-solubilizing activity. However, the relationship between the two traits remain unknown. But Chaiharn *et al*. (Chaiharn and Lumyong 2011) found that the isolates that had the ability to solubilize inorganic phosphate could also produce IAA. It was therefore not surprising that the two traits were highly correlated in this study (see Fig. S3, Supporting Information). The phosphate solubilization results showed a strong variation in the radial distance of the clear zone surrounding the colony periphery, probably indicating that isolate diversity closely mirrored isolate ability to solubilize phosphate.

In this study, the BOX-PCR data revealed the presence of high genetically-diverse rhizobial populations in soils from the agro-ecological regions of South Africa and Mozambique. The 54 authenticated cowpea nodule bacteria used in this study grouped into eight distinct clusters in the Box-PCR analysis. Of the 54 isolates, 45 unique BOX-types were obtained, which suggests that the isolates had variable distances in repetitive and conserved sequences, a finding consistent with the report by Chidebe *et al*. (Chidebe, Jaiswal and Dakora 2018). The isolates’ distribution in clusters within the dendrogram showed that the South African isolates originating from Marapyane exhibited greater diversity when compared to the Mozambican isolates, which showed similar distribution to the results reported by Pule-Meulenberg *et al*. (Pule-meulenberg *et al*. 2010). Many isolates were grouped closely together in clusters according to their location of origin in this study, a finding similar to the reports from Senegal, Greece, South Africa and Mozambique (Kouyaté *et al*. 2014; Tampakaki *et al*. 2017; Chidebe, Jaiswal and Dakora 2018; Mohammed, Jaiswal and Dakora 2018). The genome of the Biofix (TUTVUI122) commercial inoculant strain did not match with any of the isolates tested, indicating poor nodule occupancy possibly from weak competitiveness.

Environmental factors, especially soil pH and mineral nutrients were found to influence rhizobial community structure and function (Mohammed, Jaiswal and Dakora 2018). The genomic DNA analysis of bradyrhizobial isolates using BOX-PCR and the CCA analysis seem to suggest the existence of a relationship between bradyrhizobial distribution and the physicochemical properties of soil. For example, bradyrhizobial isolates collected from Marapyane, South Africa, were highly influenced by Na content of soil, and by soil pH of isolates from Muriaze in Mozambique. Ndungu *et al*. (Ndungu *et al*. 2018) reported that the occurrence and abundance of diverse cowpea rhizobial populations in Kenya was influenced by soil pH, which often affected the bioavailability of mineral nutrients in soils. Recently, Mohammed *et al*. (Mohammed, Jaiswal and Dakora 2018) and Puozaa *et al*.(Puozaa, Jaiswal and Dakora 2019) also reported that soil pH and mineral nutrients can influence the diversity of bradyrhizobial populations in South African and Ghanaian soils, and found that N, P and Na concentrations could specifically influence South African isolates. The results of this study also suggested that bradyrhizobial distribution in soil was strongly controlled by soil physico-chemical properties such as pH, and mineral nutrients. Therefore, in the African agro-ecosystem, edaphic effects can alter the bardyrhizobial distribution in soils (Mohammed, Jaiswal and Dakora 2019).

The evolutionary relationship of the bacterial isolates from cowpea, based on 16S rRNA, housekeeping (*atpD, glnII, gyrB* and *recA*) and symbiotic *nifH* and *nodC* gene sequences, revealed the presence of diverse bradyrhizobial species in both South African and Mozambican soils. The isolates were grouped in diverse monophyletic clusters within individual gene trees constructed from 16S rRNA, housekeeping and symbiotic gene sequences. Except for *recA* phylogeny, all other single gene phylogenies were very congruent to each other. The congruency of isolates from soils of South Africa and Mozambique in all five clusters of the phylogenetic trees could suggest the acquisition of symbiotic genes through vertical gene transfer, except for isolate TUTVUMm110 in *atpD* phylogeny. In the *recA* phylogeny, isolates TUTVUMr93, TUTVUMr85, TUTVUMm105 and TUTVUMr94 showed inconsistencies which could be attributed to horizontal gene transfer, subsequent recombination events, and/or differences in the evolutionary history of the gene (Menna and Hungria 2011; Naamala, Jaiswal and Dakora 2016; Jaiswal, Msimbira and Dakora 2017; Chidebe, Jaiswal and Dakora 2018). An important consideration is that, in general, all groups in the phylogenies of the housekeeping genes *atpD, glnII* and *gyrB*, as well as the symbiotic *nifH* and *nodC* genes were similar to the 16S rRNA gene tree, thus confirming the usefulness of these genes for phylogenetic reconstruction of the genus *Bradyrhizobium*. In this study, geographic origin did affect the phylogenetic placement of isolates in the housekeeping gene trees, which diminished the hypothesis of a common origin for *Bradyrhizobium* and supported the evolution of these isolates by allopatric speciation. For example, isolates from Marapyane, Muriaze and Ruace did not cluster together, and/or aligned with any known *Bradyrhizobium* reference type strains.

To get a clearer resolution in the relationship between test isolates and type reference strains, phylogenetic information was deduced from combined sequence analysis of more than one gene (Chidebe, Jaiswal and Dakora 2018). In this study, concatenated *atpD+glnII+gyrB* gene sequence analysis yielded the same results as the individual phylogenetic trees, with the isolates grouping in five clusters. In Cluster I, isolate TUTVUMp6 obtained from South Africa, and isolates TUTVUMr103, TUTVUMm112, TUTVUMm113 and TUTVUMm114 from Mozambique, all showed very close relationship with *B. yuanmingense* CCBAU 10071^T^ which was originally isolated from the genus *Lespedeza* in China (Yao *et al*. 2002). Isolates TUTVUMp60, TUTVUMp75 from South Africa and TUTVUMm117 from Mozambique shown in Cluster II, was closely related to *B. pachyrhizi* PAC48^T^ originally obtained from root nodules of *Pachyrhizus erosus* in Costa Rica (Ramírez-Bahena *et al*. 2009). Recently, *B. pachyrhizi* was also identified as a cowpea-nodulating microsymbiont in Angola (Grönemeyer *et al*. 2014), Spain (Bejarano *et al*. 2014), South Africa (Mohammed, Jaiswal and Dakora 2018) and Mozambique (Chidebe, Jaiswal and Dakora 2018). Isolate TUTVUMm110 in Cluster IV obtained from Mozambican soil and the commercial Biofix strain TUTVUI122 were very close to *B. arachidis* CCBAU 055107^T^, a finding consistent with the results of Chidebe *et al*. (Chidebe, Jaiswal and Dakora 2018). Due to the absence of *gyrB* sequence of *B. arachidis* CCBAU 055107^T^, it was excluded in the concatenated phylogeny. The isolates in Clusters IIa, III and V did not show any close relationship with known reference type strains of *Bradyrhizobium*, and could therefore be novel *Bradyrhizobium* species in South African and Mozambican soils, this indicating allopatric speciation.

Taken together, the bradyrhizobial isolates from this study exhibited varying morpho-physiological and molecular characteristics, which clearly suggested that uncultivated soils of the South African site and farmers’ fields in Mozambique harboured diverse and novel bradyrhizobia of African origin capable of releasing IAA and solubilising P for plant growth. The monophyletic grouping of the isolates within phylogenetic trees could suggest their geographic isolation in the African continent, and hence prove their allopatric speciation. The acidic soils and other unique edaphic factors were probably responsible for the presence of these diverse indigenous and novel bradyrhizobia nodulating cowpea in Africa. These isolates should be further studied systematically to ascertain their identity and symbiotic performance for subsequent testing under field conditions to identify strains for inoculant production.

## FUNDING

This work was supported with grants from the Bill and Melinda Gates Foundation Project on Capacity Building in Legume Sciences in Africa, the South African Department of Science and Technology, the Tshwane University of Technology, the National Research Foundation in Pretoria, and the South African Research Chair in Agrochemurgy and Plant Symbioses.

## Supplementary Material

fiz067_Supplement_Files

## References

[bib1] Abel S, Ballas N, Wong L-M et al. DNA elements responsive to auxin. Bioessays. 1996;18:647–54. 8760338 10.1002/bies.950180808

[bib2] Ahmad M, Zahir ZA, Nazli F et al. Effectiveness of halo-tolerant, auxin producing *Pseudomonas* and *Rhizobium* strains to improve osmotic stress tolerance in mung bean (*Vigna**radiata* L.). Braz J Microbiol. 2013;1348:1341–8. 10.1590/s1517-83822013000400045 PMC3958208 24688532

[bib3] Anjum SA, Xie X, Wang L et al. Morphological, physiological and biochemical responses of plants to drought stress. African J Agric Res. 2011;6:2026–32.

[bib4] Bejarano A, Ramírez-Bahena MH, Velázquez E et al. *Vigna* *unguiculata* is nodulated in Spain by endosymbionts of Genisteae legumes and by a new symbiovar (vignae) of the genus *Bradyrhizobium*. Syst Appl Microbiol. 2014;37:533–40. 24867807 10.1016/j.syapm.2014.04.003

[bib5] Broughton WJ, Dilworth MJ Control of leghaemoglobin synthesis in snake beans. Biochem J. 1971;125:1075–80. 5144223 10.1042/bj1251075 PMC1178271

[bib6] Chaiharn M, Lumyong S Screening and optimization of indole-3-acetic acid production and phosphate solubilization from rhizobacteria aimed at improving plant growth. Curr Microbiol. 2011;62:173–81. 20552360 10.1007/s00284-010-9674-6

[bib7] Chidebe IN, Jaiswal SK, Dakora FD Distribution and phylogeny of microsymbionts associated with cowpea (*Vigna**unguiculata*) nodulation in three agroecological regions of Mozambique. Appl Environ Microbiol. 2018;84:1–25. 10.1128/AEM.01712-17 PMC5752868 29101189

[bib8] Dakora FD, Keya SO Contribution of legume nitrogen fixation to sustainable agriculture in Sub-Saharan Africa. Soil Biol Biochem. 1997;29:809–17.

[bib9] Datta C, Basu PS Indole acetic acid production by a *Rhizobium* species from root nodules of a leguminous shrub, Cajanus cajan Microbiol Res. 2000;155:123–7. 10950195 10.1016/S0944-5013(00)80047-6

[bib10] Degefu T, Wolde-meskel E, Woliy K et al. Phylogenetically diverse groups of *Bradyrhizobium* isolated from nodules of tree and annual legume species growing in Ethiopia. Syst Appl Microbiol. 2017;40:205–14. 28499469 10.1016/j.syapm.2017.04.001

[bib11] Echevarria-Zomeño S, Ariza D, Jorge I et al. Changes in the protein profile of *Quercus**ilex* leaves in response to drought stress and recovery. J Plant Physiol. 2009;166:233–45. 18778874 10.1016/j.jplph.2008.05.008

[bib12] Etesami H, Alikhani HA, Akbari AA et al. Evaluation of plant growth hormones production (IAA) ability by Iranian soils rhizobial strains and effects of superior strains application on wheat growth indexes. World Appl Sci J. 2009;6:1576–84.

[bib13] Felsenstein J Confidence limits on phylogenies: an approach using the bootstrap. Evolution. 1985;39:783–91. 28561359 10.1111/j.1558-5646.1985.tb00420.x

[bib14] Grönemeyer JL, Kulkarni A, Berkelmann D et al. Identification and characterization of rhizobia indigenous to the Okavango region in Sub-Saharan Africa. Appl Environ Microbiol. 2014, DOI: 10.1128/AEM.02417-14. PMC4249195 25239908

[bib15] Hall TA BioEdit: a user-friendly biological sequence alignment editor and analysis program for Windows 95/98/NT. InNucleic acid symposium series. 1999;41:95–8.

[bib16] Hunter WJ Indole-3-acetic acid production by bacteroids from soybean root nodules. Physiol Plant. 1989;76:31–6.

[bib17] Jaiswal SK, Dakora FD Widespread distribution of highly adapted *Bradyrhizobium* species nodulating diverse legumes in Africa. Front Microbiol. 2019;10:310. 30853952 10.3389/fmicb.2019.00310 PMC6395442

[bib18] Jaiswal SK, Msimbira LA, Dakora FD Phylogenetically diverse group of native bacterial symbionts isolated from root nodules of groundnut (*Arachis**hypogaea* L.) in South Africa. Syst Appl Microbiol. 2017;40:215–26. 28372899 10.1016/j.syapm.2017.02.002 PMC5460907

[bib19] Kouyaté Z, Krasova-Wade T, Yattara II et al. Effects of cropping system and Cowpea variety (*Vigna**unguiculata* L. Walp) on the diversity of native cowpea bradyrhizobia and millet yield in the Sudano Sahelian zone of Mali. Int Res J Agric Sci Soil Sci. 2014;4:2251–44.

[bib20] Kumar S, Stecher G, Tamura K MEGA7 : molecular evolutionary genetics analysis version 7.0 for bigger datasets. Mol Biol Evol. 2016;33:1870–4. 27004904 10.1093/molbev/msw054 PMC8210823

[bib21] Marinho R de CN, Ferreira L de VM, Silva AF da et al. Symbiotic and agronomic efficiency of new cowpea rhizobia from Brazilian Semi-Arid. Bragantia. 2017;76:273–81.

[bib22] Marra LM, Soares CRFS, de Oliveira SM et al. Biological nitrogen fixation and phosphate solubilization by bacteria isolated from tropical soils. Plant Soil. 2012;357:289–307.

[bib23] Menna P, Hungria M Phylogeny of nodulation and nitrogen-fixation genes in *Bradyrhizobium*: supporting evidence for the theory of monophyletic origin, and spread and maintenance by both horizontal and vertical transfer. Int J Syst Evol Microbiol. 2011;61:3052–67. 21357454 10.1099/ijs.0.028803-0

[bib24] Mohale KC, Belane AK, Dakora FD Symbiotic N nutrition, C assimilation, and plant water use efficiency in Bambara groundnut (*Vigna**subterranea* L. Verdc) grown in farmers’ fields in South Africa, measured using ^15^N and ^13^C natural abundance. Biol Fertil Soils. 2013;50:307–19.

[bib25] Mohammed M, Jaiswal SK, Dakora FD Distribution and correlation between phylogeny and functional traits of cowpea (*Vigna**unguiculata* L. Walp.)-nodulating microsymbionts from Ghana and South Africa. Sci Rep. 2018;8:1–19. 30573737 10.1038/s41598-018-36324-0 PMC6302100

[bib26] Mohammed M, Jaiswal SK, Dakora FD Insights into the phylogeny, nodule functioning and biogeographic distribution of microsymbionts nodulating the orphan Kersting's groundnut [*Macrotyloma**geocarpum* (Harms) Marechal & Baudet] in African soils. Appl Environ Microbiol. 2019: doi:10.1128/AEM.00342-19. PMC6532025 30952658

[bib27] Naamala J, Jaiswal SK, Dakora FD Microsymbiont diversity and phylogeny of native bradyrhizobia associated with soybean (*Glycine max* L. Merr.) nodulation in South African soils. Syst Appl Microbiol. 2016;39:336–44. 27324571 10.1016/j.syapm.2016.05.009 PMC4958686

[bib28] Ndungu SM, Messmer MM, Ziegler D et al. Cowpea (*Vigna**unguiculata* L. Walp) hosts several widespread bradyrhizobial root nodule symbionts across contrasting agro-ecological production areas in Kenya. Agric Ecosyst Environ. 2018;261:161–71. 29970945 10.1016/j.agee.2017.12.014 PMC5946706

[bib29] Oksanen J, Blanchet FG, Kindt R et al. others (2010) vegan: community ecology package. R Packag version. 2010;2016:0–2.

[bib30] Prell J, Poole P Metabolic changes of rhizobia in legume nodules. Trends Microbiol. 2006;14:161–8. 16520035 10.1016/j.tim.2006.02.005

[bib31] Pule-meulenberg F, Belane AK, Krasova-wade T et al. Symbiotic functioning and bradyrhizobial biodiversity of cowpea (*Vigna**unguiculata* L. Walp.) in Africa. BMC Microbiol. 2010;10:89. 20331875 10.1186/1471-2180-10-89 PMC2858033

[bib32] Puozaa DK, Jaiswal SK, Dakora FD Phylogeny and distribution of *Bradyrhizobium* symbionts nodulating cowpea (*Vigna**unguiculata* L. Walp) and their association with the physicochemical properties of acidic African soils. Syst Appl Microbiol. 2019 10.1016/j.syapm.2019.02.004 PMC6542415 30803810

[bib33] R Core Team. R: A language and environment for statistical computing, R foundation for Statistical Computing, Vienna, Austria. 2015 https://www.R.project.org/

[bib34] Raddadi N, Cherif A, Boudabous A et al. Screening of plant growth promoting traits of *Bacillus**thuringiensis*. Ann Microbiol. 2008;58:47–52.

[bib35] Rahman MS, Forhad Quadir Q, Rahman A et al. Agriculture, livestock and fisheries screening and characterization of phosphorus solubilizing bacteria and their effect on rice seedlings. Res Agric, Livest Fish Res. 2014;1:27–35.

[bib36] Ramírez-Bahena MH, Peix A, Rivas R et al. *Bradyrhizobium* *pachyrhizi* sp. nov. and *Bradyrhizobium**jicamae* sp. nov., isolated from effective nodules of *Pachyrhizus**erosus*, Int J Syst Evol Microbiol–34. 2009;59:1929–34. 10.1099/ijs.0.006320-0 19567584

[bib37] Rohlf FJ, Applied Biostatistics I, Exeter Software (Firm). NTSYS-Pc : Numerical Taxonomy and Multivariate Analysis System. Applied Biostatistics, Inc, 2009.

[bib38] Tampakaki AP, Fotiadis CT, Ntatsi G et al. Phylogenetic multilocus sequence analysis of indigenous slow-growing rhizobia nodulating cowpea (*Vigna**unguiculata* L.) in Greece. Syst Appl Microbiol. 2017;40:179–89. 28214058 10.1016/j.syapm.2017.01.001

[bib39] Unkovich MJ, Pate JS. An appraisal of recent field measurements of symbiotic N_2_ fixation by annual legumes. Field Crops Res. 2000;65:211–28.

[bib40] Vassilev N, Vassileva M, Fenice M et al. Immobilized cell technology applied in solubilization of insoluble inorganic (rock) phosphates and P plant acquisition. Bioresour Technol. 2001;79:263–71. 11499580 10.1016/s0960-8524(01)00017-7

[bib41] Wade TK, Le Quéré A, Laguerre G et al. Eco-geographical diversity of cowpea bradyrhizobia in Senegal is marked by dominance of two genetic types. Syst Appl Microbiol. 2014;37:129–39. 24373721 10.1016/j.syapm.2013.10.002

[bib42] Yanni YG, Rizk RY, El-Fattah FKA et al. The beneficial plant growth-promoting association of *Rhizobium**leguminosarum* bv. trifolii with rice roots. Funct Plant Biol. 2001;28:845–70.

[bib43] Yao ZY, Kan FL, Wang ET et al. Characterization of rhizobia that nodulate legume species of the genus *Lespedeza* and description of *Bradyrhizobium**yuanmingense* sp. nov. Int J Syst Evol Microbiol. 2002;52:2219–30. 12508891 10.1099/00207713-52-6-2219

