# Supplementary material for: Phylogenetic evidence of allopatric speciation of bradyrhizobia nodulating cowpea (Vigna unguiculata L. walp) in South African and Mozambican soils
Source: FEMS Microbiol Ecol. 2019 May 16;95(6):fiz067. doi: 10.1093/femsec/fiz067 (PMC6531793; doi:10.1093/femsec/fiz067)
Supplement: fiz067_Supplement_Files [file fiz067_supplement_files.zip › Supplementary.docx]

**Table S1: Soil pH and macronutrient information of study sites**

| **Locations** | **pH** | **Organic C** | **N** | **Ca** | **Mg** | **Na** | **K** | **P** |
| --- | --- | --- | --- | --- | --- | --- | --- | --- |
|  | **(KCl)** | **(%)** | | mg kg^-1^ | | | | |
| **Marapeyane** | **5.2** | **1.21** | **0.16** | **5** | **2.83** | **35** | **246** | **8** |
| **Muriaze** | **6** | **1.3** | **0.12** | **6.06** | **0.85** | **11** | **134** | **21** |
| **Ruace** | **5.9** | **-** | **0.05** | **4.02** | **0.56** | **13.2** | **221** | **26.1** |

**Table S2**: Primers and PCR temperature profiles used for DNA amplification.

| **Primers** | **Sequences 5’ – 3’** | **Temperature profiles** | | **References** |
| --- | --- | --- | --- | --- |
| BOXA1R | 5’CTACGGCAAGGCGACGCTGACG3’ | 7 min at 95°C, 34 × (1 min at 94°C, 1 min at 52.8°C, and 8 min at 65^o^C), 16 min at 65^o^C | | Versalovic *et al.* (1994) |
|  |  |  | |  |
| 16S rRNA F  16S rRNA R | 5’AGAGTTTGATCCTGGCTCAG3’  5’CTTAAGGAGGTGATCCAGCC3’ | 5 min at 95°C, 35 × (1 min at 95°C, 1 min at 55°C, 1 min at 72°C), 10 min at 72°C | | Weisburg *et al.* (1991) |
|  |  | | | |
| *nifH* F  *nifH* R | 5’TACGGNAARGGSGGNATCGGCAA3’  5’AGCATGTCYTCSAGYTCNTCCA3’ | 5 min at 94°C, 20 × [30s at 94°C, 30s at 65°C (-0.5°C\cycle), 90s at 72°C], 25 x (30s at 94°C, 30s at 55°C, 90s at 72°C), 10 min at 72°C | | Nzoué *et al.* (2009) |
|  |  | | | |
| *glnll* F  *glnII* R | 5’AAGCTCGAGTACATCTGGCTCGACGG3’  5’SGAGCCGTTCCAGTCGGTGGTGTCG3’ | | 2 min at 95°C, 35 × (45s at 95°C, 30s at 65°C, 90s at 72°C), 10 min at 72°C | Stepkowski *et al.* (2011) |
|  |  | | | |
| *gyrB* F  *gyrB* R | 5’TTCGACCAGAAYTCCTAYAAGG3’  5’AGCTTGTCCTTSGTCTGCG3’ | 10 min at 95°C, 35 × (30s at 94°C, 30s at 58°C, 1 min at 72°C), 10 min at 72°C | | Marek-Kozaczuk *et al.* (2013) |
|  |  | | | |
| *atpD* F  *atpD* R | 5’TCTGGTCCGYGGCCAGGAAG3’  5’CGACACTTCCGARCCSGCCTG3’ | 2 min at 95^o^C, 35 × (45s at 95^o^C, 30s at 65^o^C, 1.5min at 72^o^C), 10 min at 72^o^C | | Jaiswal *et al.* (2017) |
|  |  | | | |
| *nodC* F  *nodC* R | 5’GTC GAT TGC MRG TCA AGA CTA CG3’  5’GCC AGG TCT IGT TGC GAT TGC TC3’ | 30s at 94°C, 40 × (30s at 94°C, 1 min at 55.4°C, 30s at 72°C), 5 min 72°C | | Sterner and Parker (1999) |
|  |  | | | |
| *recA* F  *recA* R | 5’CAACTGCMYTGCGTATCGTCGAAGG3’  5’CGGATCTGGTTGATGAAGATCACCATG3’ | 5 min at 95 ºC, 34 × (45s at 98 ºC, 30s at 79.3 ºC, 90s at 72 ºC), 10 min at 72 ºC | | Stępkowski *et al.* (2011) |

**REFERENCES**

Jaiswal SK, Msimbira LA, Dakora FD. Phylogenetically diverse group of native bacterial symbionts isolated from root nodules of groundnut (*Arachis hypogaea* L.) in South Africa. *Syst* *Appl* *Microbiol* 2017;**40**:215-26.

Marek-Kozaczuk M, Leszcz A, Wielbo J, Wdowiak-Wróbel S, Skorupska A. *Rhizobium pisi* sv. trifolii K3.22 harboring nod genes of the *Rhizobium leguminosarum* sv. trifolii cluster. *Syst Appl Microbiol* 2013;**36**:252–258.

Nzoué A, Miché L, Klonowska A, Laguerre G, de Lajudie P, Moulin L. Multilocus sequence analysis of bradyrhizobia isolated from *Aeschynomene* species in Senegal. *Syst Appl Microbiol* 2009;**32**:400–412.

Stepkowski T, Zak M, Moulin L, Króliczak J, Golińska B, Narozna D, Safronova VI, Madrzak CJ. *Bradyrhizobium* *canariense* and *Bradyrhizobium japonicum* are the two dominant rhizobium species in root nodules of lupin and serradella plants growing in Europe. *Syst Appl Microbiol* 2011;**34**:368–375.

Sterner JP, Parker MA. Diversity and Relationships of Bradyrhizobia from *Amphicarpaea* *bracteata* Based on partial nod and Ribosomal Sequences. *Syst* *Appl* *Microbiol* 1999;**22**:387-392.

Versalovic J, Schneider M, De Bruihn FJ, Lupski JR. Genomic fingerprinting of bacteria using Repetitive Sequence-Based ploymerase chain reaction. *J Clin Microbiol* 1994;**5**:25–40.

Weisburg WG, Barns SM, Pelletier DA *et al.* 16S ribossomal DNA amplification for phylogenetic study. *J Bacteriol* 1991;**173**:697–703.

**Table S3:** Sequences information of 16S rRNA, housekeeping genes and symbiotic genes

| **Locus** | **Number of strains used for tree construction** | **Number of conserved sites** | **Number of variable sites** | **Number of parsimony-informative sites** | **Number of singleton sites** | **Total** | **T/C/A/G** |
| --- | --- | --- | --- | --- | --- | --- | --- |
| 16S rRNA | 76 | 545 (73.8) | 180 (24.4) | 36 (4.8) | 144 (19.5) | 738 | 20.5/22.0/26.2/31.4 |
| *atpD* | 73 | 266 (63.2) | 155 (36.8) | 105 (24.9) | 50 (11.9) | 421 | 19.6/31.3/15.2/ 33.9 |
| *glnII* | 82 | 287 (60.0) | 191 (39.9) | 152 (31.8) | 39 (8.2) | 478 | 17.1/33.6/19.2/ 30.1 |
| *gyrB* | 78 | 254 (48.7) | 268 (51.3) | 177 (33.9) | 91 (17.4) | 522 | 16.5/32.7/20.0/30.7 |
| *nifH* | 73 | 110 (42.1) | 91 (34.9) | 77(29.5) | 14 (5.4) | 201 | 19.1/27.1/19.5/ 34.3 |
| *nodC* | 45 | 28 (13.86) | 174 (86.13) | 153 (75.74) | 21 (10.39) | 202 | 20.3/26.7/24.2/28.9 |
| *recA* | 82 | 221 (58.2) | 159 (41.8) | 119 (31.3) | 40 (10.5) | 380 | 16.8/28.6/17.8/36.5 |
| Concatenate *(atpD+glnII+gyrB)* | 67 | 818(57.6) | 603(42.4) | 422(29.7) | 181(12.7) | 1421 | 17.6/32.6/18.3/31.5 |

**Fig. S1:** Standard curve of absorbance plotted against known IAA concentrations

**16S rRNA**

>Seq1 [organism= Bradyrhizobium sp. TUTVUMp5] [strain=TUTVUMp5] 16S ribosomal RNA gene, partial sequence

GCAGGCTTAACACATGCAAGTCGAGCGGGCGTAGCAATACGTCAGCGGCAGACGGGTGAG

TAACGCGTGGGAACGTACCTTTTGGTTCGGAACAACACAGGGAAACTTGTGCTAATACCG

GATAAGCCCTTACGGGGAAAGATTTATCGCCGAAAGATCGGCCCGCGTCTGATTAGCTAG

TTGGTGAGGTAATGGCTCACCAAGGCGACGATCAGTAGCTGGTCTGAGAGGATGATCAGC

CACATTGGGACTGAGACACGGCCCAAACTCCTACGGGAGGCAGCAGTGGGGAATATTGGA

CAATGGGGGCAACCCTGATCCAGCCATGCCGCGTGAGTGATGAAGGCCCTAGGGTTGTAA

AGCTCTCTTGTGCGGGAAGATAATGACGGTACCGCAAGAATAAGCCCCGGCTAACTTCGT

GCCAGCAGCCGCGGTAATACGAAGGGGGCTAGCGTTGCTCGGAATCACTGGGCGTAAAGG

GTGCGTAGGCGGGTCTTTAAGTCAGGGGTGAAATCCTGGAGCTCAACTCCAGAACTGCCT

TTGATACTGAAGATCTTGAGTTCGGGAGAGGTGAGTGGAACTGCGAGTGTAGAGGTGAAA

TTCGTAGATATTCGCAAGAACACCAGTGGCGAAGGCGGCTCACTGGCCCGATACTGACGC

TGAGGCACGAAAGCGTGGGGAGCAAACAGGATTAGATACCCTGGTAGTCCACGCCGTACA

CGATGAATGCCAGCCGTTAGTGGGTTTACTCACTAGTGGCGCAGCTAACGCTTTAAGCAT

TCCGCCTGGGGAGTACGGTCGCAAGATTAAAACTCAAAGGAATTGACGGGGGCCCGCACA

AGCGGTGGAGCATGTGGTTTAATTCGACGCAACGCGCAGAACCTTACCAGCCCTTGACAT

GTCCAGGACCGGTCGCAGAGATGTGACCCTCTCTTCGGAGCCTGGAACACAGGTGCTGCA

TGGCTGTCGTCAGCTCGTGTCGTGAGATGTTGGGTTAAGTCCCGCAACGAGCGCAACCCC

CGTCCTTAGTTGCTACCATTTAGTTGAGCACTCTAAGGAGACTGCCGTGGATAAGCCGCG

>Seq2 [organism= Bradyrhizobium sp. TUTVUMp6] [strain=TUTVUMp6] 16S ribosomal RNA gene, partial sequence

TAACATGCAAGTCGAGCGGGCGTAGCAATACGTCAGCGGCAGACGGGTGAGTAACGCGTG

GGAACGTACCTTTTGGTTCGGAACAACACAGGGAAACTTGTGCTAATACCGGATAAGCCC

TTACGGGGAAAGATTTATCGCCGAAAGATCGGCCCGCGTCTGATTAGCTAGTTGGTGAGG

TAATGGCTCACCAAGGCGACGATCAGTAGCTGGTCTGAGAGGATGATCAGCCACATTGGG

ACTGAGACACGGCCCAAACTCCTACGGGAGGCAGCAGTGGGGAATATTGGACAATGGGGG

CAACCCTGATCCAGCCATGCCGCGTGAGTGATGAAGGCCCTAGGGTTGTAAAGCTCTTTT

GTGCGGGAAGATAATGACGGTACCGCAAGAATAAGCCCCGGCTAACTTCGTGCCAGCAGC

CGCGGTAATACGAAGGGGGCTAGCGTTGCTCGGAATCACTGGGCGTAAAGGGTGCGTAGG

CGGGTCTTTAAGTCAGGGGTGAAATCCTGGAGCTCAACTCCAGAACTGCCTTTGATACTG

AAGATCTTGAGTCCGGGAGAGGTGAGTGGAACTGCGACTGTAGAGGTGAAATTCGTAGAT

ATTCGCAAGAACACCAGTGGCGAAGGCGGCTCACTGGCCCGGTACTGACGCTGAGGCACG

AAAGCGTGGGGAGCAAAGACGATTAGATCCACTGGTAGTCCACGCCGTAAACGATGAATGC

GAGCCGTTAGTGGGTTTACTCACTAGTGGCGCAGCTAACGCTTTAAGCATTCCGCCTGGG

GAGTACGGTCGCAAGATTAAAACTCAAAGGAATTGACGGGGGCCCGCACAAGCGGTGGAG

CATGTGGTTTAATCGTACGCAACGCGCAGAACCTTACCAGCCCTTGACATGTCCAGGACC

GGTCGCAGAGATGTGACCCTCTCTTCGGAGCCTGGAGCACAGGTGCTGCATGGCTGTCGT

CAGCTCGTGTCGTGAGATGTTGG

>Seq3 [organism= Bradyrhizobium sp. TUTVUMp8] [strain=TUTVUMp8] 16S ribosomal RNA gene, partial sequence

TGGCGGCAGGCTTAACACATGCAAGTCGAGCGGGCGTAGCAATACGTCAGCGGCAGACGG

GTGAGTAACGCGTGGGAACGTACCTTTTGGTTCGGAACAACACAGGGAAACTTGTGCTAA

TACCGGATAAGCCCTTACGGGGAAAGATTTATCGCCGAAAGATCGGCCCGCGTCTGATTA

GCTAGTTGGTGAGGTAATGGCTCACCAAGGCGACGATCAGTAGCTGGTCTGAGAGGATGA

TCAGCCACATTGGGACTGAGACACGGCCCAAACTCCTACGGGAGGCAGCAGTGGGGAATA

TTGGACAATGGGGGCAACCCTGATCCAGCCATGCCGCGTGAGTGATGAAGGCCCTAGGGT

TGTAAAGCTCTTTTGTGCGGGAAGATAATGACGGTACCGCAAGAATAAGCCCCGGCTAAC

TTCGTGCCAGCAGCCGCGGTAATACGAAGGGGGCTAGCGTTGCTCGGAATCACTGGGCGT

AAAGGGTGCGTAGGCGGGTCTTTAAGTCAGGGGTGAAATCCTGGAGCTCAACTCCAGAAC

TGCCTTTGATACTGAAGATCTTGAGTCCGGGAGAGGTGAGTGGAACTGCGAGTGTAGAGG

TGAAATTCGTAGATATTCGCAAGAACACCAGTGGCGAAGGCGGCTCACTGGCCCGGTACT

GACGCTGAGGCACGAAAGCGTGGGGAGCAAACAGGATTAGATACCCTGGTAGTCCACGCC

GTAAACGATGAATGCCAGCCGTTAGTGGGTTTACTCACTAGTGGCGCAGCTAACGCTTTA

AGCATTCCGCCTGGGGAGTACGGTCGCAAGATTAAAACTCAAAGGAATTGACGGGGGCCC

GCACAAGCGGTGGAGCATGTGGTTTAATTCGACGCAACGCGCAGAACCTTACCAGCCCTT

GACATGTCCAGGACCGGTCGCAGAGATGTGACCTTCTCTTCGGAGCCTGGAACACAGGTG

CTGCATGGCTGTCGTCAGCTCGTGTCGTGAGATGTTGGGTTAAGTCCCGCAACGAGCGCA

ACCCCCGTCCTTAGTTGCTACCATTTAGTTGAGCACTCTAAGGAGACTGCCGGTGATAAC

CCCCGAGAAAGGTGGGGATGACGTCAAGTCCTCAGGGCCCTTACGGGCTGGGCTACACAC

CTGCTACAATGGCGGTGACAATGGGATGCGAAGAACCCAGTCCTAGCAAATCTCAAAAAA

CCGCCTCATTTCGAATTGGGGCCTGCCACTCCAGCCCATGAAATTTGAATTCCTCTTAAT

CTTGGATCAGCACGCCACCGCGAATACTTTCCCGGGCCTGTTACACACCGCCGCTCACAC

CCAGGGAATTGGTTTTTCCTTAAGAAAGTGGCCTAACCCGCCAGGGAGGC

>Seq4 [organism= Bradyrhizobium sp. TUTVUMp48] [strain=TUTVUMp48] 16S ribosomal RNA gene, partial sequence

CACATGCAAGTCGAGCGGGCGTAGCAATGCGTCAGCGGCAGACG

GGTGAGTAACGCGTGGGAACGTACCTTTTGGTTCGGAACAACACAGGGAAACTTGTGCTA

ATACCGGATAAGCCCTTACGGGGAAAGATTTATCGCCGAAAGATCGGCCCGCGTCTGATT

AGCTAGTTGGTGAGGTAATGGCTCACCAAGGCGACGATCAGTAGCTGGTCTGAGAGGATG

ATCAGCCACATTGGGACTGAGACACGGCCCAAACTCCTACGGGAGGCAGCAGTGGGGAAT

ATTGGACAATGGGGGCAACCCTGATCCAGCCATGCCGCGTGAGTGATGAAGGCCCTAGGG

TTGTAAAGCTCTTTTGTGCGGGAAGATAATGACGGTACCGCAAGAATAAGCCCCGGCTAA

CTTCGTGCCAGCAGCCGCGGTAATACGAAGGGGGCTAGCGTTGCTCGGAATCACTGGGCG

TAAAGGGTGCGTAGGCGGGTCTTTAAGTCAGGGGTGAAATCCTGGAGCTCAACTCCAGAA

CTGCCTTTGATACTGAAGATCTTGAGTCCGGGAGAGGTGAGTGGAACTGCGAGTGTAGAG

GTGAAATTCGTAGATATTCGCAAGAACACCAGTGGCGAAGGCGGCTCACTGGCCCGGTAC

TGACGCTGAGGCACGAAAGCGTGGGGAGCAAACAGGATTAGATACCCTGGTAGTCCACGC

CGTAAACGATGAATGCCAGCCGTTAGTGGGTTTACTCACTAGTGGCGCAGCTAACGCTTT

AAGCATTCCGCCTGGGGAGTACGGTCGCAAGATTAAAACTCAAAGGAATTGACGGGGGCC

CGCACAAGCGGTGGAGCATGTGGTTTAATTCGACGCAACGCGCAGAACCTTACCAGCCCT

TGACATGTCCAGGACCGGTCGCAGAGATGTGACCTTCTCTTCGGAGCCTGGAACACAGGT

GCTGCATGGCTGTCGTCAGCTCGTGTCGTGAGATGTTGGGTTAAGTCCCGCAACGAGCGC

AACCCCCGTCCTTAGTTGCTACCATTTAGTTGAGCACTCTAAGGAGACTGCCGGTGATAA

GCCGCCAGGAAAGGGGGGGATGACGTCAAGTCCTCCTGGCCCTTACGGGCTGGGCTACAC

ACCTGCTACAATGGCGGGGACAATGGGATGCGAAGAACCCAGTCCTACCAAATCTCAAAA

AGCCGTCTCATTTCGGATTGGGGTCTGCCACCCGAGCCCCTGAAATTGGAATCCCTAGAT

AATCTGGGTCAACCCCCCACGGTGAAAACATTCCCGGGCCTGGAACACACCGCCCGCCCA

CCCATGGGAATTGGGTTTTCCTGGAAAAGGTGGGCTAACCCGCAAGGAGGGCCGCCGGCC

>Seq5 [organism= Bradyrhizobium sp. TUTVUMp53] [strain=TUTVUMp53] 16S ribosomal RNA gene, partial sequence

ACACATGCAAGTCGAGCGGGCGTAGCAATACGTCAGCGGCAGACGGGTGAGTAACGCGTG

GGAACGTACCTTTTGGTTCGGAACAACACAGGGAAACTTGTGCTAATACCGGATAAGCCC

TTACGGGGAAAGATTTATCGCCGAAAGATCGGCCCGCGTCTGATTAGCTAGTTGGTGAGG

TAATGGCTCACCAAGGCGACGATCAGTAGCTGGTCTGAGAGGATGATCAGCCACATTGGG

ACTGAGACACGGCCCAAACTCCTACGGGAGGCAGCAGTGGGGAATATTGGACAATGGGGG

CAACCCTGATCCAGCCATGCCGCGTGAGTGATGAAGGCCCTAGGGTTGTAAAGCTCTTTT

GTGCGGGAAGATAATGACGGTACCGCAAGAATAAGCCCCGGCTAACTTCGTGCCAGCAGC

CGCGGTAATACGAAGGGGGCTAGCGTTGCTCGGAATCACTGGGCGTAAAGGGTGCGTAGG

CGGGTCTTTAAGTCAGGGGTGAAATCCTGGAGCTCAACTCCAGAACTGCCTTTGATACTG

AAGATCTTGAGTCCGGGAGAGGTGAGTGGAACTGCGAGTGTAGAGGTGAAATTCGTAGAT

ATTCGCAAGAACACCAGTGGCGAAGGCGGCTCACTGGCCCGGTACTGACGCTGAGGCACG

AAAGCGTGGGGAGCAAACAGGATTAGATACCCTGGTAGTCCACGCCGTAAACGATGAATG

CCAGCCGTTAGTGGGTTTACTCACTAGTGGCGCAGCTAACGCTTTAAGCATTCCGCCTGG

GGAGTACGGTCGCAAGATTAAAACTCAAAGGAATTGACGGGGGCCCGCACAAGCGGTGGA

GCATGTGGTTTAATTCGACGCAACGCGCAGAACCTTACCAGCCCTTGACATGTCCAGGAC

CGGTCGCAGAGATGTGACCTTCTCTTCGGAGCCTGGAACACAGGTGCTGCATGGCTGTCG

TCAGCTCGTGTCGTGAGATGTTGGGTTAAGTCCCGCAACGAGCGCAACCCCCGTCCTTAG

TTGCTACCATTTAGTTGAGCACTCTAAGGAGACTGCCGGTGATAAGCCCCCAGGAAAGTG

GGGATGACGTCAAGTCCTCATGGCCCTTACGGGCTGGGCTACACCCCTGCTACAATGGGG

GGGACAATGGGATGCGAAAACCCAATTCCTATCAAATCTCAAAAAACCGCCTCTTTTCGG

ATTGGGGTCTTGCACTCCAGCCCCTGAAATTTGAATCGCTTTTAATCTTGGGACACCCCC

CCCCGGGAAAAAATTTCCCGGGCCTTGTACACACCGCCCGTCCAACCCAGGAGGTTGGTT

TTTTCTGAAAAAAGTGGGCTAACCCCCAAGGGAGGCAGCCGGCCACGGTA

>Seq6 [organism= Bradyrhizobium sp. TUTVUMp56] [strain=TUTVUMp56] 16S ribosomal RNA gene, partial sequence

CGGCAGGCTTAACACATGCAAGTCGAGCGGGCGTAGCAATACGTCAGCGGCAGACGGGTG

AGTAACGCGTGGGAACGTACCTTTTGGTTCGGAACAACACAGGGAAACTTGTGCTAATAC

CGGATAAGCCCTTACGGGGAAAGATTTATCGCCGAAAGATCGGCCCGCGTCTGATTAGCT

AGTTGGTGAGGTAATGGCTCACCAAGGCGACGATCAGTAGCTGGTCTGAGAGGATGATCA

GCCACATTGGGACTGAGACACGGCCCAAACTCCTACGGGAGGCAGCAGTGGGGAATATTG

GACAATGGGGGCAACCCTGATCCAGCCATGCCGCGTGAGTGATGAAGGCCCTAGGGTTGT

AAAGCTCTTTTGTGCGGGAAGATAATGACGGTACCGCAAGAATAAGCCCCGGCTAACTTC

GTGCCAGCAGCCGCGGTAATACGAAGGGGGCTAGCGTTGCTCGGAATCACTGGGCGTAAA

GGGTGCGTAGGCGGGTCTTTAAGTCAGGGGTGAAATCCTGGAGCTCAACTCCAGAACTGC

CTTTGATACTGAAGATCTTGAGTCCGGGAGAGGTGAGTGGAACTGCGAGTGTAGAGGTGA

AATTCGTAGATATTCGCAAGAACACCAGTGGCGAAGGCGGCTCACTGGCCCGGTACTGAC

GCTGAGGCACGAAAGCGTGGGGAGCAAACAGGATTAGATACCCTGGTAGTCCACGCCGTA

AACGATGAATGCCAGCCGTTAGTGGGTTTACTCACTAGTGGCGCAGCTAACGCTTTAAGC

ATTCCGCCTGGGGAGTACGGTCGCAAGATTAAAACTCAAAGGAATTGA

>Seq7 [organism= Bradyrhizobium sp. TUTVUMp65] [strain=TUTVUMp65] 16S ribosomal RNA gene, partial sequence

CATGCAAGTCGAGCGGGCGTAGCAATACGTCAGCGGCAGACGGGTGAGTAACGCGTGGGA

ACGTACCTTTTGGTTCGGAACAACACAGGGAAACTTGTGCTAATACCGGATAAGCCCTTA

CGGGGAAAGATTTATCGCCGAAAGATCGGCCCGCGTCTGATTAGCTAGTTGGTGAGGTAA

TGGCTCACCAAGGCGACGATCAGTAGCTGGTCTGAGAGGATGATCAGCCACATTGGGACT

GAGACACGGCCCAAACTCCTACGGGAGGCAGCAGTGGGGAATATTGGACAATGGGGGCAA

CCCTGATCCAGCCATGCCGCGTGAGTGATGAAGGCCCTAGGGTTGTAAAGCTCTTTTGTG

CGGGAAGATAATGACGGTACCGCAAGAATAAGCCCCGGCTAACTTCGTGCCAGCAGCCGC

GGTAATACGAAGGGGGCTAGCGTTGCTCGGAATCACTGGGCGTAAAGGGTGCGTAGGCGG

GTCTTTAAGTCAGGGGTGAAATCCTGGAGCTCAACTCCAGAACTGCCTTTGATACTGAAG

ATCTTGAGTCCGGGAGAGGTGAGTGGAACTGCGAGTGTAGAGGTGAAATTCCTAAATATT

CGCAAGAACACCAGTGGCGAAGGCGGCTCACTGGCCCGGTACTGACGCTGAGGCACGAAA

GCGTGGGGAGCAAACAGGATTAGATACCCTGGTAGTCCACGCCGTAAACGATGAATGCCA

GCCGTTAGTGGGTTTACTCACTAGTGGCGCAGCTAACGCTTTAAGCATTCCGCCTGGGGA

GTACGGTCGCAAGATTAAAACTCAAAGGAATTGACGGGGGCCCGCACAAGCGGTGGAGCA

TGTGGTTTAATTCGACGCAACGCGCAGAACCTTACCAGCCCTTGACATGTCCAGGACCGG

TCGCAGAGATGTGACCTTCTCTTCGGAGCCTGGAACACAGGTGCTGCATGGCTGTCGTCA

GCTCGTGTCGTGAGATGTTGGGTTAAGTCCCGCAACGAGCGCAACCCCCGTCCTTAGTTG

CTACCATTTAGTTGAGCACTCTAAGGAGACTGCCGGTGATAAGCCCCCAAGAAAGTGGGG

ATGACGTCAAGTCCTCATGGCCCTTACGGGCTGGGCTACACACCTGCTACAATGGCCGTG

AAAATGGGATGCCAAAACGCCAGTCCTAGCAAATTTCAAAAACCCGTTTTAATTCGGATT

GGGCTCTGCAACTCGAGGCCCTGAAGTTGGAATCCCTATATATCTGGGAACACCACGCCA

CGGTGAAAACGTTCCCGGCCTTGGTACACCCCGCCCGTCACACTGGGGAAGTTGGTTTTA

CCTGGAAAGGGTGCGCTTACCCGCAAGGAAGGCCGCC

>Seq8 [organism= Bradyrhizobium sp. TUTVUMp75] [strain=TUTVUMp75] 16S ribosomal RNA gene, partial sequence

TGTCGGGAAGCTTACACATGCAAGTCGAGCGGGCATAGCAATATGTCAGCGGCAGACGGG

TGAGTAACGCGTGGGAACGTACCTTTTGGTTCGGAACAACTGAGGGAAACTTCAGCTAAT

ACCGGATAAGCCCTTACGGGGAAAGATTTATCGCCGAAAGATCGGCCCGCGTCTGATTAG

CTAGTTGGTGAGGTAATGGCTCACCAAGGCGACGATCAGTAGCTGGTCTGAGAGGATGAT

CAGCCACATTGGGACTGAGACACGGCCCAAACTCCTACGGGAGGCAGCAGTGGGGAATAT

TGGACAATGGGCGCAAGCCTGATCCAGCCATGCCGCGTGAGTGATGAAGGCCCTAGGGTT

GTAAAGCTCTTTTGTGCGGGAAGATAATGACGGTACCGCAAGAATAAGCCCCGGCTAACT

TCGTGCCAGCAGCCGCGGTAATACGAAGGGGGCTAGCGTTGCTCGGAATCACTGGGCGTA

AAGGGTGCGTAGGCGGGTCTTTAAGTCAGGGGTGAAATCCTGGAGCTCAACTCCAGAACT

GCCTTTGATACTGAAGATCTTGAGTTCGGGAGAGGTGAGTGGAACTGCGAGTGTAGAGGT

GAAATTCGTAGATATTCGCAAGAACACCAGTGGCGAAGGCGGCTCACTGGCCCGATACTG

ACGCTGAGGCACGAAAGCGTGGGGAGCAAACAGGATTAGATACCCTGGTAGTCCACGCCG

TAAACGATGAATGCCAGCCGTTAGTGGGTTTACTCACTAGTGGCGCAGCTAACGCTTTAA

GCATTCCGCCTGGGGAGTACGGTCGCAAGATTAAAACTCAAAGGAATTGACGGGGGCCCG

CACAAGCGGTGGAGCATGTGGTTTAATTCGACGCAACGCGCAGAACCTTACCAGCCCTTG

ACATCCCGGTCGCGGACTCCAGAGACGGAGTTCTTCAGTTCGGCTGGACCGGAGACAGGT

GCTGCATGGCTGTCGTCAGCTCGTGTCGTGAGATGTTGGGTTAAGTCCCGCAACGAGCGC

AACCCCCGTCCTTAGTTGCTACCATTTAGTTGAGCACTCTAAGGAGACTGCCGGTGATAA

GCCGCTAAGAAGGTGGGGATGACGTCAAGTCCTCATGGCCCTTACGGGCTCGGCTACACC

CCTGCTACACCAGCGCAAACAAGGGGGGGACAAGGGCGGACCCTTCGGGAATCTCAAAAA

CCCGTTTCAATTCGGATTGGGGTCTGCAATTGGAGCCAATAAAAATTGAACCCCTAGAAA

TTCGGGATCCGCCCCCCACGGTGGAAACATTCCCGGGCCGGGTACACCCGGGCCGTGCCC

CCATGGGAATTGGTTTTCCCTGGAAAAGGGGGCTTTACCAAAAGGGGCCAGCCGGCC

>Seq9 [organism= Bradyrhizobium sp. TUTVUMr80] [strain=TUTVUMr80] 16S ribosomal RNA gene, partial sequence

ATTGCGGCAGCTTACCATGCAAGTCGAGCGGGCGTAGCAATACGTCAGCGGCAGACGGGT

GAGTAACGCGTGGGAACGTACCTTTTGGTTCGGAACAACACAGGGAAACTTGTGCTAATA

CCGGATAAGCCCTTACGGGGAAAGATTTATCGCCGAAAGATCGGCCCGCGTCTGATTAGC

TAGTTGGTAGGGTAATGGCCTACCAAGGCGACGATCAGTAGCTGGTCTGAGAGGATGATC

AGCCACATTGGGACTGAGACACGGCCCAAACTCCTACGGGAGGCAGCAGTGGGGAATATT

GGACAATGGGGGCAACCCTGATCCAGCCATGCCGCGTGAGTGATGAAGGCCCTAGGGTTG

TAAAGCTCTTTTGTGCGGGAAGATAATGACGGTACCGCAAGAATAAGCCCCGGCTAACTT

CGTGCCAGCAGCCGCGGTAATACGAAGGGGGCTAGCGTTGCTCGGAATCACTGGGCGTAA

AGGGTGCGTAGGCGGGTTTTTAAGTCAGGGGTGAAATCCTGGAGCTCAACTCCAGAACTG

CCTTTGATACTGAAGATCTTGAGTCCGGGAGAGGTGAGTGGAACTGCGAGTGTAGAGGTG

AAATTCGTAGATATTCGCAAGAACACCAGTGGCGAAGGCGGCTCACTGGCCCGGTACTGA

CGCTGAGGCACGAAAGCGTGGGGAGCAAACAGGATTAGATACCCTGGTAGTCCACGCCGT

AAACGATGAATGCCAGCCGTTAGTGGGTTTACTCACTAGTGGCGCAGCTAACGCTTTAAG

CATTCCGCCTGGGGAGTACGGTCGCAAGATTAAAACTCAAAGGAATTGACGGGGGCCCGC

ACAAGCGGTGGAGCATGTGGTTTAATTCTACGCAACGCGCAGAACCTTACCAGTCCTTGA

CATGTGCATGACCGGTCGCAGAGATGTGACCCTCTCTTCGCAGCCTGGAACACAGGTGCT

GCATGGCTGTCGTCAGCTCGTGTCTTGAGATGTTGGGTTAAGTCCCTTAACGAGCGCAAC

CTCCGTCCTTAGTTGCTACCATTTAGTTCACCACTCTAAGGATACTGCCGGTGATAAGCC

>Seq10 [organism= Bradyrhizobium sp. TUTVUMr82] [strain=TUTVUMr82] 16S ribosomal RNA gene, partial sequence

TAACACATGCAAGTCGAGCGGGCATAGCAATATGTCAGCGGCAGACGGGTGAGTAACGCG

TGGGAACGTACCTTTTGGTTCGGAACAACTGAGGGAAACTTCAGCTAATACCGGATAAGC

CCTTACGGGGAAAGATTTATCGCCGAAAGATCGGCCCGCGTCTGATTAGCTAGTTGGTGA

GGTAATGGCTCACCAAGGCGACGATCAGTAGCTGGTCTGAGAGGATGATCAGCCACATTG

GGACTGAGACACGGCCCAAACTCCTACGGGAGGCAGCAGTGGGGAATATTGGACAATGGG

CGCAAGCCTGATCCAGCCATGCCGCGTGAGTGATGAAGGCCCTAGGGTTGTAAAGCTCTT

TTGTGCGGGAAGATAATGACGGTACCGCAAGAATAAGCCCCGGCTAACTTCGTGCCAGCA

GCCGCGGTAATACGAAGGGGGCTAGCGTTGCTCGGAATCACTGGGCGTAAAGGGTGCGTA

GGCGGGTCTTTAAGTCAGGGGTGAAATCCTGGAGCTCAACTCCAGAACTGCCTTTGATAC

TGAAGATCTTGAGTTCGGGAGAGGTGAGTGGAACTGCGAGTGTAGAGGTGAAATTCGTAG

ATATTCGCAAGAACACCAGTGGCGAAGGCGGCTCACTGGCCCGATACTGACGCTGAGGCA

CGAAAGCGTGGGGAGCAAACAGGATTAGATACCCTGGTAGTCCACGCCGTAAACGATGAA

TGCCAGCCGTTAGTGGGTTTACTCACTAGTGGCGCAGCTAACGCTTTAAGCATTCCGCCT

GGGGAGTACGGTCGCAAGATTAAAACTCAAAGGAATTGACGGGGGCCCGCACAAGCGGTG

GAGCATGTGGTTTAATTCGACGCAACGCGCAGAACCTTACCAGCCCTTGACATCCCGGTC

GCGGACTCCAGATACAGAGTTCTTCTCTTCGGCTGGACCGGACACAGGTGCTGCATGGCT

GTCGTCAGCTCGTGTCTTGAGATGTTGGGTTAAGTCCCGCAACGAGCGCAACCCTCGTCC

TTAGGTGCTACCATTTATTTGAGCACTCTAAGGAGACTGCCGGTGATAGGCCCCAAGCAC

CGCGGGGATGACGGGCAGTACTCCTGGCCCTCCAGGGCTGGATTAACCCCGTGCTACAAC

GGCGGTGAAAATGGGAGGCCAAGGGGCGACCCTTGGGGAGTCCCAAAAACCGCTTCAAAT

ACGGATTGGGGTCTGGCAATAGGGCCACTTAATTTCGAACCCCATAGAATTGGGGAACCG

CCCCCCCCGTGGAAAACATTCCCTGGCC

>Seq11 [organism= Bradyrhizobium sp. TUTVUMr85] [strain=TUTVUMr85] 16S ribosomal RNA gene, partial sequence

ACACATGCAAGTCGAGCGGGCGTAGCAATACGTCAGCGGCAGACGGGTGAGTAACGCGTG

GGAACGTACCTTTTGGTTCGGAACAACACAGGGAAACTTGTGCTAATACCGGATAAGCCC

TTACGGGGAAAGATTTATCGCCGAAAGATCGGCCCGCGTCTGATTAGCTAGTTGGTAGGG

TAATGGCCTACCAAGGCGACGATCAGTAGCTGGTCTGAGAGGATGATCAGCCACATTGGG

ACTGAGACACGGCCCAAACTCCTACGGGAGGCAGCAGTGGGGAATATTGGACAATGGGGG

CAACCCTGATCCAGCCATGCCGCGTGAGTGATGAAGGCCCTAGGGTTGTAAAGCTCTTTT

GTGCGGGAAGATAATGACGGTACCGCAAGAATAAGCCCCGGCTAACTTCGTGCCAGCAGC

CGCGGTAATACGAAGGGGGCTAGCGTTGCTCGGAATCACTGGGCGTAAAGGGTGCGTAGG

CGGGTTTTTAAGTCAGGGGTGAAATCCTGGAGCTCAACTCCAGAACTGCCTTTGATACTG

AAGATCTTGAGTCCGGGAGAGGTGAGTGGAACTGCGAGTGTAGAGGTGAAATTCGTAGAT

ATTCGCAAGAACACCAGTGGCGAAGGCGGCTCACTGGCCCGGTACTGACGCTGAGGCACG

AAAGCGTGGGGAGCAAACAGGATTAGATACCCTGGTAGTCCACGCCGTAAACGATGAATG

CCAGCCGTTAGTGGGTTTACTCACTAGTGGCGCAGCTAACGCTTTAAGCATTCCGCCTGG

GGAGTACGGTCGCAAGATTAAAACTCATAGGAATTGACGGGGGCCCGCACAAGCGGTGGA

GCATGTGGTTTAATTCGACGCAACGCGCAGAACCTTACCAGCCCTTGACATGTCCAGGAC

CGGTCGCAGAGATGTGACCCTCTCTTCGGAGCCTGGAACACAGGTGCTGCATGGCTGTCG

TCAGCTCGTGTCTTGAGATGTTGGGTTAAGTCCCGCAACGAGCGCAACCTCCGTCCTTAG

TTGCTACCATTTAGTCGAGCACTCTAAGGATACTGCCGGTGATAAGCCGCCAAGAACGGG

GGGATGACGTCAAGTCCTCCTGGCCCTTCTGGGCTGGATTACCCCCGTGCTACAATGGCG

GTGACAATGGGGTGCTAAGGGGCGACCCTTGGGGATTCTCAAAAATCCATCCAAGTACGG

ATTGGGCTCTTGAATTCGAGCCCATGACTTCGAAACCCCTAGTAATCGGGGAACCGCCAC

CCACGTGGAATAAATTCCCGGGCCCTGGTAAAACCGTCGGGTGAACCTTGGAAATGGGTT

TTACTTGAAAAAAGTGGGCTTAAACCAAATGGGAACCCCGTCCC

>Seq12 [organism= Bradyrhizobium sp. TUTVUMr89] [strain=TUTVUMr89] 16S ribosomal RNA gene, partial sequence

CACATGCAAGTCGAGCGGGCGTAGCAATACGTCAGCGGCAGACGGGTGAGTAACGCGTGG

GAACGTACCTTTTGGTTCGGAACAACACAGGGAAACTTGTGCTAATACCGGATAAGCCCT

TACGGGGAAAGATTTATCGCCGAAAGATCGGCCCGCGTCTGATTAGCTAGTTGGTGAGGT

AATGGCTCACCAAGGCGACGATCAGTAGCTGGTCTGAGAGGATGATCAGCCACATTGGGA

CTGAGACACGGCCCAAACTCCTACGGGAGGCAGCAGTGGGGAATATTGGACAATGGGGGC

AACCCTGATCCAGCCATGCCGCGTGAGTGATGAAGGCCCTAGGGTTGTAAAGCTCTTTTG

TGCGGGAAGATAATGACGGTACCGCAAGAATAAGCCCCGGCTAACTTCGTGCCAGCAGCC

GCGGTAATACGAAGGGGGCTAGCGTTGCTCGGAATCACTGGGCGTAAAGGGTGCGTAGGC

GGGTCTTTAAGTCAGGGGTGAAATCCTGGAGCTCAACTCCAGAACTGCCTTTGATACTGA

AGATCTTGAGTCCGGGAGAGGTGAGTGGAACTGCGAGTGTAGAGGTGAAATTCGTAGATA

TTCGCAAGAACACCAGTGGCGAAGGCGGCTCACTGGCCCGGTACTGACGCTGAGGCACGA

AAGCGTGGGGAGCAAACAGGATTAGATACCCTGGTAGTCCACGCCGTAAACGATGAATGC

CAGCCGTTAGTGGGTTTACTCACTAGTGGCGCAGCTAACGCTTTAAGCATTCCGCCTGGG

GAGTACGGTCGCAAGATTAAAACTCAAAGGAATTGACGGGGGCCCGCACAAGCGGTGGAG

CATGTGGTTTAATTCGACGCAACGCGCAGAACCTTACCAGCCCTTGACATGTCCAGGACC

GGTCGCAGAGATGTGACCTTCTCTTCGGAGCCTGGAACACAGGTGCTGCATGGCTGTCGT

CAGCTCGTGTCGTGAGATGTTGGGTTAAGTCCCGCAACGAGCGCAACCCCCGTCCTTAGT

TGCTACCATTTAGTTGAGCACTCTAAGGAGACTGCCGGTGATAAGCCGCGAGGAAGTTGG

GGATGACGTCAAGTCCTCAGGGCCCTTACGGGCTGGGCTACACACGTGCTACAATGGCGG

TGACAATGGGATGCGAAGACCCAGTTCCTAGCAAATCTCAAAAAGCCTTCTCATTTCGGA

TTGGGGCCTTCCACTCCGGCCCCTGAAATTTGAATCCCTACTTATCTTGGATAACCACGC

CCCGGTGAAAACTTTCCCGGGCCTTGTACACACCGCCCTCCCCACCGGGGAAGTTGGTTT

TTCCTGGAAAACGTGCGCTTACCCGCCAGGGAGGCC

>Seq13 [organism= Bradyrhizobium sp. TUTVUMr93] [strain=TUTVUMr93] 16S ribosomal RNA gene, partial sequence

TGCAAGTCGAGCGGGCGTAGCAATACGTCAGCGGCAGACGGGTGAGTAACGCG

TGGGAACGTACCTTTTGGTTCGGAACAACACAGGGAAACTTGTGCTAATACCGGATAAGC

CCTTACGGGGAAAGATTTATCGCCGAAAGATCGGCCCGCGTCTGATTAGCTAGTTGGTAG

GGTAATGGCCTACCAAGGCGACGATCAGTAGCTGGTCTGAGAGGATGATCAGCCACATTG

GGACTGAGACACGGCCCAAACTCCTACGGGAGGCAGCAGTGGGGAATATTGGACAATGGG

GGCAACCCTGATCCAGCCATGCCGCGTGAGTGATGAAGGCCCTAGGGTTGTAAAGCTCTT

TTGTGCGGGAAGATAATGACGGTACCGCAAGAATAAGCCCCGGCTAACTTCGTGCCAGCA

GCCGCGGTAATACGAAGGGGGCTAGCGTTGCTCGGAATCACTGGGCGTAAAGGGTGCGTA

GGCGGGTTTTTAAGTCAGGGGTGAAATCCTGGAGCTCAACTCCAGAACTGCCTTTGATAC

TGAAGATCTTGAGTCCGGGAGAGGTGAGTGGAACTGCGAGTGTAGAGGTGAAATTCGTAG

ATATTCGCAAGAACACCAGTGGCGAAGGCGGCTCACTGGCCCGGTACTGACGCTGAGGCA

CGAAAGCGTGGGGAGCAAACAGGATTAGATACCCTGGTAGTCCACGCCGTAAACGATGAA

TGCCAGCCGTTAGTGGGTTTACTCACTAGTGGCGCAGCTAACGCTTTAAGCATTCCGCCT

GGGGAGTACGGTCGCAAGATTAAAACTCAAAGGAATTGACGGGGGCCCGCACAAGCGGTG

GAGCATGTGGTTTAATTCGACGCAACGCGCAGAACCTTACCAGCCCTTGACATGTCCAGG

ACCGGTCGCAGAGATGTGACCTTCTCTTCGGAGCCTGGAACACAGGTGCTGCATGGCTGTCG

TCAGCTCGTGTCGTGAGATGTTGGG

>Seq14 [organism= Bradyrhizobium sp. TUTVUMr94] [strain=TUTVUMr94] 16S ribosomal RNA gene, partial sequence

ACACATGCTAGTCGAGCGGGCGTAGCAATACGTCAGCGGCAGACGGGTGAGTAACGCGTG

GGAACGTACCTTTTGGTTCGGAACAACACAGGGAAACTTGTGCTAATACCGGATAAGCCC

TTACGGGGAAAGATTTATCGCCGAAAGATCGGCCCGCGTCTGATTAGCTAGTTGGTGAGG

TAATGGCTCACCAAGGCGACGATCAGTAGCTGGTCTGAGAGGATGATCAGCCACATTGGG

ACTGAGACACGGCCCAAACTCCTACGGGAGGCAGCAGTGGGGAATATTGGACAATGGGGG

CAACCCTGATCCAGCCATGCCGCGTGAGTGATGAAGGCCCTAGGGTTGTAAAGCTCTTTT

GTGCGGGAAGATAATGACGGTACCGCAAGAATAAGCCCCGGCTAACTTCGTGCCAGCAGC

CGCGGTAATACGAAGGGGGCTAGCGTTGCTCGGAATCACTGGGCGTAAAGGGTGCGTAGG

CGGGTCTTTAAGTCAGGGGTGAAATCCTGGAGCTCAACTCCAGAACTGCCTTTGATACTG

AAGATCTTGAGTCCGGGAGAGGTGAGTGGAACTGCGAGTGTAGAGGTGAAATTCGTAGAT

ATTCGCAAGAACACCAGTGGCGAAGGCGGCTCACTGGCCCGGTACTGACGCTGAGGCACG

AAAGCGTGGGGAGCAAACAGGATTAGATACCCTGGTAGTCCACGCCGTAAACGATGAATG

CCAGCCGTTAGTGGGTTTACTCACTAGTGGCGCAGCTAACGCTTTAAGCATTCCGCCTGG

CGAGTACGGTCGCGAGATTAAAACTCAAATGAATTGACGGGAGCCCGCACAAGCGGTGCA

GCATGTGGTTTAATTCTACTCAACGCGCAGAACCTTACCAGCCCTTGACCTGTCCATGAC

CGGCCGCAGATATGAGACCCTCTCTCCGCAGCCTGGAGCACAGGTGCTGCATGGCTGTCG

TCAGCTCCTTTCATGAGATGCTGGG

>Seq15 [organism= Bradyrhizobium sp. TUTVUMr95] [strain=TUTVUMr95] 16S ribosomal RNA gene, partial sequence

TGGCGGCAGGCTTAACACATGCAAGTCGAGCGGGCGTAGCAATACGTCAGCGGCAGACGG

GTGAGTAACGCGTGGGAACATACCTTTTGGTTCGGAACAACACAGGGAAACTTGTGCTAA

TACCGGATAAGCCCTTACGGGGAAAGATTTATCGCCGAAAGATTGGCCCGCGTCTGATTA

GCTAGTTGGTAGGGTAATGGCCTACCAAGGCGACGATCAGTAGCTGGTCTGAGAGGATGA

TCAGCCACATTGGGACTGAGACACGGCCCAAACTCCTACGGGAGGCAGCAGTGGGGAATA

TTGGACAATGGGGGCAACCCTGATCCAGCCATGCCGCGTGAGTGATGAAGGCCCTAGGGT

TGTAAAGCTCTTTTGTGCGGGAAGATAATGACGGTACCGCAAGAATAAGCCCCGGCTAAC

TTCGTGCCAGCAGCCGCGGTAATACGAAGGGGGCTAGCGTTGCTCGGAATCACTGGGCGT

AAAGGGTGCGTAGGCGGGTTTTTAAGTCAGGGGTGAAATCCTGGAGCTCAACTCCAGAAC

TGCCTTTGATACTGAAGATCTTGAGTCCGGGAGAGGTGAGTGGAACTGCGAGTGTAGAGG

TGAAATTCGTAGATATTCGCAAGAACACCAGTGGCGAAGGCGGCTCACTGGCCCGGTACT

GACGCTGAGGCACGAAAGCGTGGGGAGCAAACAGGATTAGATACCCTGGTAGTCCACGCC

GTAAACGATGAATGCCAGCCGTTAGTGGGTTTACTCACTAGTGGCGCAGCTAACGCTTTA

AGCATTCCGCCTGGGGAGTACGGTCGCAAGATTAAAACTCAAATGAATTGACGGGGGCCC

GCACAAGCGGTGGAGCATGTGGTTTAATTCGACGCAACGCGCAGAACCTTACCAGCCCTT

GACATGTCCAGGACCGGTCGCAGAGATGTGACCTTCTCTTCGGAGCCTGGAACACAGGTG

CTGCATGGCTGTCGTCAGCTCGTGTCGTGAGATGTTGGGTTAAGTCCCGCAACGAGCGCA

ACCCCCGTCCTTAGTTGCTACCATTTAGTTGAGCACTCTAAGGAGACTGCCGGTGATAAG

>Seq16 [organism= Bradyrhizobium sp. TUTVUMr99] [strain=TUTVUMr99] 16S ribosomal RNA gene, partial sequence

GCAAGCTTAACACATGCAAGTCGAGCGGGCATAGCAATATGTCAGCGGCAGACGGGTGAGTAA

CGCGTGGGAACGTACCTTTTGGTTCGGAACAACTGAGGGAAACTTCAGCTAATACCGGAT

AAGCCCTTACGGGGAAAGATTTATCGCCGAAAGATCGGCCCGCGTCTGATTAGCTAGTTG

GTGAGGTAATGGCTCACCAAGGCGACGATCAGTAGCTGGTCTGAGAGGATGATCAGCCAC

ATTGGGACTGAGACACGGCCCAAACTCCTACGGGAGGCAGCAGTGGGGAATATTGGACAA

TGGGCGCAAGCCTGATCCAGCCATGCCGCGTGAGTGATGAAGGCCCTAGGGTTGTAAAGC

TCTTTTGTGCGGGAAGATAATGACGGTACCGCAAGAATAAGCCCCGGCTAACTTCGTGCC

AGCAGCCGCGGTAATACGAAGGGGGCTAGCGTTGCTCGGAATCACTGGGCGTAAAGGGTG

CGTAGGCGGGTCTTTAAGTCAGGGGTGAAATCCTGGAGCTCAACTCCAGAACTGCCTTTG

ATACTGAAGATCTTGAGTTCGGGAGAGGTGAGTGGAACTGCGAGTGTAGAGGTGAAATTC

GTAGATATTCGCAAGAACACCAGTGGCGAAGGCGGCTCACTGGCCCGATACTGACGCTGA

GGCACGAAAGCGTGGGGAGCAAACAGGATTAGATACCCTGGTAGTCCACGCCGTAAACGA

TGAATGCCAGCCGTTAGTGGGTTTACTCACTAGTGGCGCAGCTAACGCTTTAAGCATTCC

TCCGGGGGAGTACGGTCGCAAGATTAAAACTCAAAGGAATTGACGGGGGCCCGCACAAGC

GGTGGAGCATGTGGTTTAATTCGACGCAACGCGCAGAACCTTACCAGCCCTTGACATCCC

GGTCGCGGACTCCAGAGACGGAGTTCTTCAGTTCGGCTGGACCGGAGACAGGTGCTGCAT

GGCTGTCGTCAGCTCGTGTCGTGAGATGTTGGGTTAAGTCC

>Seq17 [organism= Bradyrhizobium sp. TUTVUMr103] [strain=TUTVUMr103] 16S ribosomal RNA gene, partial sequence

GGCAGGCTTAACACATGCAAGTCGAGCGGGCGTAGCAATACGTCAGCGGCAGACGGGTGA

GTAACGCGTGGGAACGTACCTTTTGGTTCGGAACAACACAGGGAAACTTGTGCTAATACC

GGATAAGCCCTTACGGGGAAAGATTTATCGCCGAAAGATCGGCCCGCGTCTGATTAGCTA

GTTGGTGAGGTAATGGCTCACCAAGGCGACGATCAGTAGCTGGTCTGAGAGGATGATCAG

CCACATTGGGACTGAGACACGGCCCAAACTCCTACGGGAGGCAGCAGTGGGGAATATTGG

ACAATGGGGGCAACCCTGATCCAGCCATGCCGCGTGAGTGATGAAGGCCCTAGGGTTGTA

AAGCTCTTTTGTGCGGGAAGATAATGACGGTACCGCAAGAATAAGCCCCGGCTAACTTCG

TGCCAGCAGCCGCGGTAATACGAAGGGGGCTAGCGTTGCTCGGAATCACTGGGCGTAAAG

GGTGCGTAGGCGGGTCTTTAAGTCAGGGGTGAAATCCTGGAGCTCAACTCCAGAACTGCC

TTTGATACTGAAGATCTTGAGTCCGGGAGAGGTGAGTGGAACTGCGAGTGTAGAGGTGAA

ATTCGTAGATATTCGCAAGAACACCAGTGGCGAAGGCGGCTCACTGGCCCGGTACTGACG

CTGAGGCACGAAAGCGTGGGGAGCAAACAGGATTAGATACCCTGGTAGTCCACGCCGTAA

ACGATGAATGCCAGCCGTTAGTGGGTTTACTCACTAGTGGCGCAGCTAACGCTTTAAGCA

TTCCGCCTGGGGAGTACGGTCGCAAGATTAAAACTCAAAGGAATTGACGGGGGCCCGCAC

AAGCGGTGGAGCATGTGGTTTAATTCGACGCAACGCGCAGAACCTTACCAGCACTTGACA

TGTCCAGGACCGGTCGCAGAGATGTGACCCTCTCTTCGGAGCCTGGAGCACAGGTGCTGC

ATGGCTGTCGTCAGCTCGTGTCGTGAGATGTTGGGTTAAGTCCCGCAACGAGCGCAACCC

CCGTTCTTAGTTGCTACCATTTATTTGACCACTCTAAGGAGACTGCCGGTGATAAGGCGC

CTAGAACGTGGGGATGACGTCAAGTCCTCATGGCCCTTACGGGGTGGGCTACACACGTGC

TACATTGCCGGTGACAATGGGAGGCTAAGGGGCGACCCTCCGGAAGTCTCAAAAAGCCGT

CTCAATACGGATGGGGCTCTGCAACTGGAGGCCATGAAATTCGAATCCCTATTAATCTGG

GAACCGCCGGCCCAGGTGAAACCGTTTCCGGGCCTTGGAAACACCGGCCATTACACCATA

GGAAAATGGTTTTACCTG

>Seq18 [organism= Bradyrhizobium sp. TUTVUMm108] [strain=TUTVUMm108] 16S ribosomal RNA gene, partial sequence

GCGGCAGGCTTAACACATGCAAGTCGAGCGGGCGTAGCAATACGTCAGCGGCAGACGGGT

GAGTAACGCGTGGGAACGTACCTTTTGGTTCGGAACAACACAGGGAAACTTGTGCTAATA

CCGGATAAGCCCTTACGGGGAAAGATTTATCGCCGAAAGATCGGCCCGCGTCTGATTAGC

TAGTTGGTAGGGTAATGGCCTACCAAGGCGACGATCAGTAGCTGGTCTGAGAGGATGATC

AGCCACATTGGGACTGAGACACGGCCCAAACTCCTACGGGAGGCAGCAGTGGGGAATATT

GGACAATGGGGGCAACCCTGATCCAGCCATGCCGCGTGAGTGATGAAGGCCCTAGGGTTG

TAAAGCTCTTTTGTGCGGGAAGATAATGACGGTACCGCAAGAATAAGCCCCGGCTAACTT

CGTGCCAGCAGCCGCGGTAATACGAAGGGGGCTAGCGTTGCTCGGAATCACTGGGCGTAA

AGGGTGCGTAGGCGGGTCTTTAAGTCAGGGGTGAAATCCTGGAGCTCAACTCCAGAACTG

CCTTTGATACTGAAGATCTTGAGTTCGGGAGAGGTGAGTGGAACTGCGAGTGTAGAGGTG

AAATTCGTAGATATTCGCAAGAACACCAGTGGCGAAGGCGGCTCACTGGCCCGATACTGA

CGCTGAGGCACGAAAGCGTGGGGAGCAAACAGGATTAGATACCCTGGTAGTCCACGCCGT

AAACGATGAATGCCAGCCGTTAGTGGGTTTACTCACTAGTGGCGCAGCTAACGCTTTAAG

CATTCCGCCTGGGGAGTACGGTCGCAAGATTATAACTCAAAGGAATTGACGGGGGCCCGC

ACAAGCGGTGGAGCATGTGGATTAATTCGACGCAACGCGCAGAACCTTACCAGCACTTGA

CATGTCCAGGACCGGTCGCAGAGACATGACCTTCTCTTCGGAGCCTGGAACACAGGTGCT

GCATGGCTGTCGTCAGCTCGTGTCGTGAGATGTTGGAGTGAGTCCCGCAACGAGCGCAGC

CCCTGTCCTTAGTTGCTACCATTTATTTGAGCATTCTAAGGAGACTGCCAGAGATAGGCG

GCTAGCACCGTGGGGATGACGTCGAGTTCACCTGGCCCTTCCCGGGTGGGCTACACCCGT

GCTACATCGCCGGTGAAAATGGGTGGGGAAGGGGCAACCCCCAGGGAGTCCCAAAAACCC

CTCTCAAATAAGAATGGGCTCTGCCCCTCGTGGCCCGGAAGTTGGAATC

>Seq19 [organism= Bradyrhizobium sp. TUTVUMm112] [strain=TUTVUMm112] 16S ribosomal RNA gene, partial sequence

ACACATGCAAGTCGAGCGGGCGTAGCAATACGTCAGCGGCAGACGGGTGAGTAACGCGTG

GGAACGTACCTTTTGGTTCGGAACAACACAGGGAAACTTGTGCTAATACCGGATAAGCCC

TTACGGGGAAAGATTTATCGCCGAAAGATCGGCCCGCGTCTGATTAGCTAGTTGGTGAGG

TAATGGCTCACCAAGGCGACGATCAGTAGCTGGTCTGAGAGGATGATCAGCCACATTGGG

ACTGAGACACGGCCCAAACTCCTACGGGAGGCAGCAGTGGGGAATATTGGACAATGGGGG

CAACCCTGATCCAGCCATGCCGCGTGAGTGATGAAGGCCCTAGGGTTGTAAAGCTCTTTT

GTGCGGGAAGATAATGACGGTACCGCAAGAATAAGCCCCGGCTAACTTCGTGCCAGCAGC

CGCGGTAATACGAAGGGGGCTAGCGTTGCTCGGAATCACTGGGCGTAAAGGGTGCGTAGG

CGGGTCTTTAAGTCAGGGGTGAAATCCTGGAGCTCAACTCCAGAACTGCCTTTGATACTG

AAGATCTTGAGTCCGGGAGAGGTGAGTGGAACTGCGAGTGTAGAGGTGAAATTCGTAGAT

ATTCGCAAGAACACCAGTGGCGAAGGCGGCTCACTGGCCCGGTACTGACGCTGAGGCACG

AAAGCGTGGGGAGCAAACAGGATTAGATACCCTGGTAGTCCACGCCGTAAACGATGAATG

CCAGCCGTTAGTGGGTTTACTCACTAGTGGCGCAGCTAACGCTTTAAGCATTCCGCCTGG

GGAGTACGGTCGCAAGATTAAAACTCAAAGGAATTGACGGGGGCCCGCACAAGCGGTGGA

GCATGTGGTTTAATTCGACGCAACGCGCAGAACCTTACCAGCCCTTGACATGTCCAGGAC

CGGTCGCAGAGATGTGACCCTCTCTTCGGAGCCTGGAGCACAGGTGCTGCATGGCTGTCG

TCAGCTCGTGTCGTGAGATGTTGGGTTAAGTCCCGCAACGAGCGCAACCCCCGTCCTTAG

TTGCTACCATTTAGTTGAGCACTCTAAGGAGACTGCCGGTGATAAGCCGCGAGGAAGGTG

GGGATGACGTCAAGTCCTCATGGCCCTTACGGGCTGGGCTACACACGTGCTACAATGGCG

GTGACAATGGGATGCTAAGGGGCGACCCTTCGCAAATCTCAAAAAGCCGTCTCAGTTCGG

ATTGGGCTCTGCAACTCGAGCCCCTGAAATTGGAATCGCTAATAATCCGGGATCACCACC

CCCCGTGGAAAACTTTCCCGGGCCTTGTACACACCGCCCGTCAACCCATGGGAATTGGTT

TTACCTGAAAACGGTGCCCTAACCCCCAAGGGAGGCACCCGGGCCCCGTAGGGTC

>Seq20 [organism= Bradyrhizobium sp. TUTVUMm113] [strain=TUTVUMm113] 16S ribosomal RNA gene, partial sequence

GCCGCAGAATTAACACATGCAAGTCGAGCGGGCGTAGCAATACGTCAGCGGCAGACGGGT

GAGTAACGCGTGGGAACGTACCTTTTGGTTCGGAACAACACAGGGAAACTTGTGCTAATA

CCGGATAAGCCCTTACGGGGAAAGATTTATCGCCGAAAGATCGGCCCGCGTCTGATTAGC

TAGTTGGTGAGGTAATGGCTCACCAAGGCGACGATCAGTAGCTGGTCTGAGAGGATGATC

AGCCACATTGGGACTGAGACACGGCCCAAACTCCTACGGGAGGCAGCAGTGGGGAATATT

GGACAATGGGGGCAACCCTGATCCAGCCATGCCGCGTGAGTGATGAAGGCCCTAGGGTTG

TAAAGCTCTTTTGTGCGGGAAGATAATGACGGTACCGCAAGAATAAGCCCCGGCTAACTT

CGTGCCAGCAGCCGCGGTAATACGAAGGGGGCTAGCGTTGCTCGGAATCACTGGGCGTAA

AGGGTGCGTAGGCGGGTCTTTAAGTCAGGGGTGAAATCCTGGAGCTCAACTCCAGAACTG

CCTTTGATACTGAAGATCTTGAGTCCGGGAGAGGTGAGTGGAACTGCGAGTGTAGAGGTG

AAATTCGTAGATATTCGCAAGAACACCAGTGGCGAAGGCGGCTCACTGGCCCGGTACTGA

CGCTGAGGCACGAAAGCGTGGGGAGCAAACAGGATTAGATACCCTGGTAGTCCACGCCGT

AAACGATGAATGTCAGCCGTTAGTGGGTTTACTCACTACTGGCGCAGCTAACGCTTTAAG

CATTCCGCCTGGGGAGTACGGTCGCGGCATTAAAACTCAAATGAATTGACTGGGGCCCGC

ACTCCCGGTGCAGCATGTGGTTTAGTTCTACTCAGCGCCCAGAAGCTTACCGGCACTTGA

CCTGTCGAGATCCAGTCACAGATATGAGACCCTCTCTTCGGACCTTGCAGCACAGATG

CTGCATGACTGTCGTCAGCTCCTGTCCTGATATGTGGGGT

>Seq21 [organism= Bradyrhizobium sp. TUTVUMm114] [strain=TUTVUMm114] 16S ribosomal RNA gene, partial sequence

GCGGCAGGCTTAACACATGCAAGTCGAGCGGGCGTAGCAATACGTCAGCGGCAGACGGGT

GAGTAACGCGTGGGAACGTACCTTTTGGTTCGGAACAACACAGGGAAACTTGTGCTAATA

CCGGATAAGCCCTTACGGGGAAAGATTTATCGCCGAAAGATCGGCCCGCGTCTGATTAGC

TAGTTGGTGAGGTAATGGCTCACCAAGGCGACGATCAGTAGCTGGTCTGAGAGGATGATC

AGCCACATTGGGACTGAGACACGGCCCAAACTCCTACGGGAGGCAGCAGTGGGGAATATT

GGACAATGGGGGCAACCCTGATCCAGCCATGCCGCGTGAGTGATGAAGGCCCTAGGGTTG

TAAAGCTCTTTTGTGCGGGAAGATAATGACGGTACCGCAAGAATAAGCCCCGGCTAACTT

CGTGCCAGCAGCCGCGGTAATACGAAGGGGGCTAGCGTTGCTCGGAATCACTGGGCGTAA

AGGGTGCGTAGGCGGGTCTTTAAGTCAGGGGTGAAATCCTGGAGCTCAACTCCAGAACTG

CCTTTGATACTGAAGATCTTGAGTCCGGGAGAGGTGAGTGGAACTGCGAGTGTAGAGGTG

AAATTCGTAGATATTCGCAAGAACACCAGTGGCGAAGGCGGCTCACTGGCCCGGTACTGA

CGCTGAGGCACGAAAGCGTGGGGAGCAAACAGGATTAGATACCCTGGTAGTCCACGCCGT

AAACGATGAATGCCAGCCGTTAGTGGGTTTACTCACTAGTGGCGCAGCTAACGCTTTAAG

CATTCCGCCTGGGGAGTACGGTCGCAAGATTAAAACTCAAAGGAATTGACGGGGGCCCGC

ACAAGCGGTGGAGCATGTGGTTTAATTCGACGCAACGCGCAGAACCTTACCAGCCCTTGA

CATGTCCAGGACCGGTCGCAGAGATGTGACCCTCTCTTCGGAGCCTGGAGCACAGGTGCT

GCATGGCTGTCGTCAGCTCGTGTCGTGAGATGTTGGGTTAAGTCCCGCAACGAGCGCAAC

CCCCGTCCTTAGTTGCTACCATTTAGTTGAGCACTCTAAGGAGACTGCCGGTGATAAGCC

GCCAAGAAAGTGGGGATGACGTCAAGTCCTCAGGGCCCTTACGGGCTGGGCTACACACT

TGCTACAATGGCGGGAACAATGGGAGGCTAAGGGGCGACCCTTCGCAAATCTCAAAAAGC

CGTCTCATTTCGGATTGGGCTCTGCCACTCCAGCCCCTGAAATTGGAATCCCTATAAATC

TGGGATCAGCCCGCCCCGGTGAAAACTTTCCCGGGCCTTGTACACACCGCCCGTCCCACC

ATGGGGAATGGGTTTTACCTGAAAAAGGTGGGCTAACCCGCAAGGGAGGC

>Seq22 [organism= Bradyrhizobium sp. TUTVUMm115] [strain=TUTVUMm115] 16S ribosomal RNA gene, partial sequence

GGCTTTACACATGTTAGTCGAGCGGGCGTAGCAATACGTCACCGGCAGACGGGTGAGTAA

CGCGTGGGAACGTACCTTTTGGTTCGGAACAACACAGGGAAACTTGTGCTAATACCGGAT

AAGCCCTTACGGGGAAAGATTTATCGCCGAAAGATCGGCCCGCGTCTGATTAGCTAGTTG

GTGAGGTAATGGCTCACCAAGGCGACGATCAGTAGCTGGTCTGAGAGGATGATCAGCCAC

ATTGGGACTGAGACACGGCCCAAACTCCTACGGGAGGCAGCAGTGGGGAATATTGGACAA

TGGGGGCAACCCTGATCCAGCCATGCCGCGTGAGTGATGAAGGCCCTAGGGTTGTAAAGC

TCTTTTGTGCGGGAAGATAATGACGGTACCGCAAGAATAAGCCCCGGCTAACTTCGTGCC

AGCAGCCGCGGTAATACGAAGGGGGCTAGCGTTGCTCGGAATCACTGGGCGTAAAGGGTG

CGTAGGCGGGTCTTTAAGTCAGGGGTGAAATCCTGGAGCTCAACTCCAGAACTGCCTTTG

ATACTGAAGATCTTGAGTCCGGGAGAGGTGAGTGGAACTGCGAGTGTAGAGGTGAAATTC

GTAGATATTCGCAAGAACACCAGTGGCGAAGGCGGCTCACTGGCCCGGTACTGACGCTGA

GGCACGAAAGCGTGGGGAGCAAACAAGATTAGATACCCTGGTAGTCCACGCCGTAAGCGA

TGAATGCCTGCCGTTAGTGGGTTTACTCACTACTGGCGCAGCTCACGCTTTAAGCATTCC

GCCTGGCGAGGACGGTCGCAAGATTAAAACTCAATGGAAATGACTGGGGCCCGCACAAGC

GGTGGAGCATGTGGTTTAATTCGACGCAACGCGCAGAACCATACCAGCCCTTGACATGTC

CAGGACCGGTCCCAGAGATGTGACCCTCTCTTCGCAGCCTGGAGCACAGGTGCTGCATGG

CTGTCGTCAGCTCGTGTCGTGAGATGTTGGGGTGAGTCCCGGAATGAGCGCAAACCCCGT

CCTTAGTTGCTACCATTTAGTTGATCACTCTAAGGAGACTGCCGGTGATAAGGCGCTAGA

ACGGTGGGGATGACGGCAAGTCCTCATGTCCCTTCCGGGCTTGGCTACACGCCTGCTACA

ATTGCCGTGACAATGGGTTGCTAGGGGG

>Seq23 [organism= Bradyrhizobium sp. TUTVUMm116] [strain=TUTVUMm116] 16S ribosomal RNA gene, partial sequence

GGCTTAAACATGCAGTCGAGCGGGCGTAGCAATACGTCAGCGGCAGACGGGTGAGTAACG

CGTGGGAACGTACCTTTTGGTTCGGAACAACACAGGGAAACTTGTGCTAATACCGGATAA

GCCCTTACGGGGAAAGATTTATCGCCGAAAGATCGGCCCGCGTCTGATTAGCTAGTTGGT

GAGGTAATGGCTCACCAAGGCGACGATCAGTAGCTGGTCTGAGAGGATGATCAGCCACAT

TGGGACTGAGACACGGCCCAAACTCCTACGGGAGGCAGCAGTGGGGAATATTGGACAATG

GGGGCAACCCTGATCCAGCCATGCCGCGTGAGTGATGAAGGCCCTAGGGTTGTAAAGCTC

TTTTGTGCGGGAAGATAATGACGGTACCGCAAGAATAAGCCCCGGCTAACTTCGTGCCAG

CAGCCGCGGTAATACGAAGGGGGCTAGCGTTGCTCGGAATCACTGGGCGTAAAGGGTGCG

TAGGCGGGTCTTTAAGTCAGGGGTGAAATCCTGGAGCTCAACTCCAGAACTGCCTTTGAT

ACTGAAGATCTTGAGTCCGGGAGAGGTGAGTGGAACTGCGAGTGTAGAGGTGAAATTCGT

AGATATTCGCAAGAACACCACTGGCGAAGGCGGCTCACTGGCCCGGTACTGACGCTGAGG

CACGAAAGCGTGGGGAGCAAACAGGATTATATACCCTGGTAGTCCACGCCGTAAACGATG

AATGCCAGCCGTTAGTGGGTTTACTCACTACTGGCGCAGCTAACGCTTTAAGCATTCCGC

CTGGCGAGTACGGTCGCAACATTAAAACTCAAATGAATTGACGGGGGCCCGCACAACCGG

TGGAGCATGTGGATTAATTCTACGCAACGCGCAGAACCTTACCAGCCCTTGACCTGTGCA

TGACCGGTCGCAGATATGAGACCCTCTCCCCGCAGCCTGGAGCACAGGTGCTGCATGGCT

GTCGTCAGCTCCTCTCATGAGATGTTGGGTTGATTCCTTAAACTACCGCAAGCCCTGTTC

TTAGTTGCTACCATTTATTCGACCATTCTAAAGA

>Seq24 [organism= Bradyrhizobium sp. TUTVUMm117] [strain=TUTVUMm117] 16S ribosomal RNA gene, partial sequence

GCGGCAGGCTTACACATGCAAGTCGAGCGGGCATAGCAATATGTCAGCGGCAGACGGGTG

AGTAACGCGTGGGAACGTACCTTTTGGTTCGGAACAACTGAGGGAAACTTCAGCTAATAC

CGGATAAGCCCTTACGGGGAAAGATTTATCGCCGAAAGATCGGCCCGCGTCTGATTAGCT

AGTTGGTGAGGTAATGGCTCACCAAGGCGACGATCAATAGCTGGTCTGAGAGGATGATCA

GCCACATTGGGACTGAGACACGGCCCAAACTCCTACGGGAGGCAGCAGTGGGGAATATTG

GACAATGGGCGCAAGCCTGATCCAGCCATGCCGCGTGAGTGATGAAGGCCCTAGGGTTGT

AAAGCTCTTTTGTGCGGGAAGATAATGACGGTACCGCAAGAATAAGCCCCGGCTAACTTC

GTGCCAGCAGCCGCGGTAATACGAAGGGGGCTAGCGTTGCTCGGAATCACTGGGCGTAAA

GGGTGCGTAGGCGGGTCTTTAAGTCAGGGGTGAAATCCTGGAGCTCAACTCCAGAACTGC

CTTTGATACTGAAGATCTTGAGTTCGGGAGAGGTGAGTGGAACTGCGAGTGTAGAGGTGA

AATTCGTAGATATTCGCAAGAACACCAGTGGCGAAGGCGGCTCACTGGCCCGATACTGAC

GCTGAGGCACGAAAGCGTGGGGAGCAAACAGGATTAGATACCCTGGTAGTCCACGCCGTA

AACGATGAATGCCAGCCGTTAGTGGGTTTACTCACTAGTGGCGCAGCTAACGCTTTAAGC

ATTCCGCCTGGGGAGTACGGTCGCAAGATTAAAACTCAAAGGAATTGACGGGGGCCCGCA

CAAGCGGTGGAGCATGTGGTTTAATTCGACGCAACGCGCAGAACCTTACCAGCCCTTGAC

ATCCCGGTCGCGGACTCCAGAGACGGAGTTCTTCAGTTCGGCTGGACCGGACACAGGTGC

TGCATGGCTGTCGTCAGCTCGTGTCGTGAGATGTTGGGTTAAGTCCCGCAACGAGCGCAA

CCTCCGTCCTTAGTTGCTACCATTTAGTTGAGCACTCTAAGGAGACTGCCGGTGATGAGC

CGCCAAGAAGGCGAGGATGACGTCAAGTCCTCATGGCCCTTACGGGCTGGGCTACACAC

GTGAAAAAATGGCGGAGACAAAGGGGAGCTAGGGGGCGACCCTTGGGGGATCTCAAAAAA

CCCTCTCAGATCGGGATGGGGTCTCCAATTCAGGCCCCTGAACTCGCAATCCTTAATAA

TCAGGAATCCGCCCGCCTCCGTGAATACATTCCCGGGCCTAGTACACATGGCCCGTCAAA

CCCTGGGAATTGGTTTTTCCT

>Seq25 [organism= Bradyrhizobium sp. TUTVUI122] [strain=TUTVUI122] 16S ribosomal RNA gene, partial sequence

GCAGGCTTACACATGCAAGTCGAGCGGGCGTAGCAATACGTCAGCGGCAGACGGGTGAGT

AACGCGTGGGAACGTACCTTTTGGTTCGGAACAACACAGGGAAACTTGTGCTAATACCGG

ATAAGCCCTTACGGGGAAAGATTTATCGCCGAAAGATCGGCCCGCGTCTGATTAGCTAGT

TGGTAGGGTAATGGCCTACCAAGGCGACGATCAGTAGCTGGTCTGAGAGGATGATCAGCC

ACATTGGGACTGAGACACGGCCCAAACTCCTACGGGAGGCAGCAGTGGGGAATATTGGAC

AATGGGGGCAACCCTGATCCAGCCATGCCGCGTGAGTGATGAAGGCCCTAGGGTTGTAAA

GCTCTTTTGTGCGGGAAGATAATGACGGTACCGCAAGAATAAGCCCCGGCTAACTTCGTG

CCAGCAGCCGCGGTAATACGAAGGGGGCTAGCGTTGCTCGGAATCACTGGGCGTAAAGGG

TGCGTAGGCGGGTCTTTAAGTCAGGGGTGAAATCCTGGAGCTCAACTCCAGAACTGCCTT

TGATACTGAAGATCTTGAGTTCGGGAGAGGTGAGTGGAACTGCGAGTGTAGAGGTGAAAT

TCGTAGATATTCGCAAGAACACCAGTGGCGAAGGCGGCTCACTGGCCCGATACTGACGCT

GAGGCACGAAAGCGTGGGGAGCAAACAGGATTAGATACCCTGGTAGTCCACGCCGTAAAC

GATGAATGCCAGCCGTTAGTGGGTTTACTCACTAGTGGCGCAGCTAACGCTTTAAGCATT

CCGCCTGGGGAGTACGGTCGCAAGATTAAAACTCAAAGGAATTGACGGGGGCCCGCACAA

GCGGTGGAGCATGTGGTTTAATTCGACGCAACGCGCAGAACCTTACCAGCCCTTGACATG

TCCAGGACCGGTCGCAGAGACGTGACCTTCTCTTCGGAGCCTGGAACACAGGTGCTGCAT

GGCTGTCGTCAGCTCGTGTCGTGAGATGTTGGGTTAAGTCCCGCAACGAGCGCAACCCCC

GTCCTTAGTTGCTACCATTTAGTTGAGCACTCTAAGGAGACTGCCGGTGATAAGCCGCGA

GAAAGGTGGGGATGACGTCAAGTCCTCATGGCCCTTACGGGCTGGGCTACACACGTGCTA

CAATGGCGGTGACAATGGGATGCAAAGGGGCAACCCCTAGCAAATTTCAAAAAGCCGTCT

CATTTCGAATTGGGCTCTGCACCCCGAGCCCATGAAATTGGAATC

***atpD***

>Seq1 [organism=Bradyrhizobium sp. TUTVUMp5] [strain=TUTVUMp5] ATP synthase beta subunit (*atpD*) gene, partial cds,

CACGTCCTGGCCCTGGTCGCGGAAGTGCTCGGCGACGGTCAGACCGGTGAGGCCGACGCG

GGCGCGGGCGCCCGGCGGCTCGTTCATCTGGCCGAACACCAGCGCGCATTTCGACTTCACG

CTCGGATCCGGATTGTGCGGATCGGCGTTGACCTTGGACTCGATGAACTCGTGATAGAG

GTCGTTGCCCTCGCGGGTACGCTCGCCGACGCCGGCGAACACCGAGTAACCACCGTGCG

CCTTGGCGACGTTGTTGATCAGTTCCTGAATCAGCACGGTCTTGCCGACGCCGGCGCCG

CCGAACAGGCCGATCTTGCCGCCCTTGGCGTAGGGCGCGAGCAGGTCGACGACCTTGAT

GCCGGTGACGAGAATTTCAGCTTCGGTCGACTGGTCGGTATAGGTCGGCGCTTCCTGGT

GGATGGCGCGAGTGCCCTCCGACTTGATCGGGCCGGCCTCGTCGATCGGCTCGCCGATG

ACGTTCATGATGCGGCCCAGCGTACCGTCACCCACGGGCACCGAAATCGGCTGACCGGT

GTCGGTCACTTCCTGGCC

>Seq2 [organism=Bradyrhizobium sp. TUTVUMp6] [strain=TUTVUMp6] ATP synthase beta subunit (*atpD*) gene, partial cds,

TGTTGTCGACGAAGAACAGCACGTCCTGGCCCTGGTCGCGGAAGTGCTCGGCGACGGTCA

GACCGGTCAGGCCGACGCGGGCGCGGGCGCCCGGCGGCTCGTTCATCTGACCGAACAC

CAGCGCGCACTTCGACTTCACGCTCGGATCCGGATTGTGCGGATCGGCGTTGACCTTG

GACTCGATGAACTCGTGATAGAGGTCGTTGCCCTCGCGGGTACGTTCGCCGACGCCGG

CGAACACGGAGTAACCGCCGTGCGCCTTCGCGACGTTGTTGATCAGTTCCTGAATCAG

CACGGTCTTGCCCACGCCGGCGCCGCCGAACAGGCCGATCTTGCCGCCCTTCGCATAC

GGCGCGAGCAGATCGACGACCTTGATGCCGGTGACGAGAATTTCGGCTTCAGTCGACT

GGTCGGTGTAGGTCGGCGCTTCCTGGTGGATGGCGCGGGTGCCTTCCGACTTGATCGG

GCCGGCCTCGTCGATCGGCTCGCCGATGACGTTCATGATGCGGCCCAGCGTACCGTCC

CCCACGGGAACCGAGATCGGCTGACCGGTGTCGGTCA

>Seq3 [organism=Bradyrhizobium sp.TUTVUMp8] [strain=TUTVUMp8] ATP synthase beta subunit (*atpD*) gene, partial cds,

CACGTCCTGGCCCTGGTCGCGGAAGTGCTCGGCGACGGTCAGGCCGGTGAGGCCGACG

CGGGCACGGGCGCCCGGCGGCTCGTTCATCTGGCCGAACACCAGCGCGCATTTCGACT

TCACGCTCGGATCCGGATTGTGCGGATCGGCGTTGACCTTGGACTCGATGAACTCGTG

ATAGAGGTCGTTGCCCTCGCGGGTACGCTCGCCGACGCCGGCGAACACCGAGTAACCA

CCGTGCGCCTTGGCGACGTTGTTGATCAGCTCCTGAATCAGCACGGTCTTGCCGACGC

CGGCGCCGCCGAACAGGCCGATCTTGCCGCCCTTGGCGTAGGGCGCGAGCAGGTCGAC

GACCTTGATGCCCGTGACGAGAATTTCAGCTTCGGTCGACTGGTCGGTATAGGTCGGC

GCTTCCTGGTGGATGGCGCGAGTGCCCTCCGACTTGATCGGGCCAGCCTCGTCGATCG

GCTCGCCGATGACGTTCATGATGCGGCCCAGCGTACCGTCACCCACAGGCACCGAGAT

CGGCTGACCGGTGTCGGTCACTTCCTG

>Seq4 [organism=Bradyrhizobium sp. TUTVUMp48] [strain=TUTVUMp48] ATP synthase beta subunit (*atpD*) gene, partial cds,

CACGTCCTGGCCCTGGTCGCGGAAGTGCTCGGCGACGGTCAGGCCGGTGAGGCCGAC

GCGGGCACGGGCGCCCGGCGGCTCGTTCATCTGGCCGAACACCAGCGCGCATTTCGACTT

CACGCTCGGATCCGGATTGTGCGGATCGGCGTTGACCTTGGACTCGATGAACTCGTGATA

GAGGTCGTTGCCCTCGCGGGTACGCTCGCCGACGCCGGCGAACACCGAGTAACCACCGTG

CGCCTTGGCGACGTTGTTGATCAGCTCCTGAATCAGCACGGTCTTGCCGACGCCGGCGCC

GCCGAACAGGCCGATCTTGCCGCCCTTGGCGTAGGGCGCGAGCAGGTCGACGACCTTGAT

GCCGGTGACGAGAATTTCAGCTTCGGTCGACTGGTCGGTATAGGTCGGCGCTTCCTGGTG

GATGGCGCGAGTGCCCTCCGACTTGATCGGGCCAGCCTCGTCGATCGGCTCGCCGATGAC

GTTCATGATGCGGCCCAGCGTACCGTCACCCACGGGCACCGAGATCGGCTGACCGGTGTC

AGTCACTTC

>Seq5 [organism=Bradyrhizobium sp. TUTVUMp50] [strain=TUTVUMp50] ATP synthase beta subunit (*atpD*) gene, partial cds,

CACGTCCTGGCCCTGGTCGCGGAAGTGCTCGGCGACGGTCAGACCGGTGAGGCCGACGCG

GGCGCGGGCACCCGGCGGCTCGTTCATCTGGCCGAACACCAGCGCGCATTTCGACTTGAC

GCTCGGATCCGGATTGTGCGGATCGGCGTTGACCTTGGACTCGATGAATTCGTGATAGAG

GTCGTTGCCCTCGCGGGTACGCTCGCCGACGCCGGCGAACACCGAGTAACCACCGTGCGC

CTTGGCGACGTTGTTGATCAGTTCCTGAATCAGCACGGTCTTGCCGACGCCAGCGCCACC

GAACAGGCCGATCTTGCCGCCCTTGGCGTAGGGCGCGAGCAGGTCGACGACTTTGATGCC

CGTGACGAGAATTTCAGCTTCGGTCGACTGGTCGGTATAGGTCGGCGCTTCCTGGTGGAT

GGCGCGAGTGCCCTCCGACTTGATCGGGCCAGCCTCGTCGATCGGCTCGCCGATGACGTT

CATGATGCGGCCCAGCGTGCCGTCACCCACGGGCACCGAGATCGGCTGACCGGTGTCAGT

CACTTCCTG

>Seq6 [organism=Bradyrhizobium sp. TUTVUMp53] [strain=TUTVUMp53] ATP synthase beta subunit (*atpD*) gene, partial cds,

CACGTCCTGGCCCTGGTCGCGGAAGTGCTCGGCGACGGTCAGACCGGTGAGGCCGAC

GCGGGCGCGGGCACCCGGCGGCTCGTTCATCTGGCCGAACACCAGCGCGCATTTCGACTT

GACGCTCGGATCCGGATTGTGCGGATCGGCGTTGACCTTGGACTCGATGAATTCGTGATA

GAGGTCGTTGCCCTCGCGGGTACGCTCGCCGACGCCGGCGAACACCGAGTAACCACCGTG

CGCCTTGGCGACGTTGTTGATCAGTTCCTGAATCAGCACGGTCTTGCCGACGCCAGCGCC

ACCGAACAGGCCGATCTTGCCGCCCTTGGCGTAGGGCGCGAGCAGGTCGACGACTTTGAT

GCCCGTGACGAGAATTTCAGCTTCGGTCGACTGGTCGGTATAGGTCGGCGCTTCCTGGTG

GATGGCGCGAGTGCCCTCCGACTTGATCGGGCCAGCCTCGTCGATCGGCTCGCCGATGAC

GTTCATGATGCGGCCCAGCGTGCCGTCACCCACGGGCACCGAGATCGGCTGACCGGTGTC

AGTCACTTC

>Seq7 [organism=Bradyrhizobium sp. TUTVUMp56] [strain=TUTVUMp56] ATP synthase beta subunit (*atpD*) gene, partial cds,

CACGTCCTGGCCCTGGTCGCGGAAGTGCTCGGCGACGGTCAGACCGGTGAGGCCGACGCG

GGCGCGGGCGCCCGGCGGCTCGTTCATCTGGCCGAACACCAGCGCGCACTTCGACTTGAC

GCTCGGATCCGGATTGTGCGGATCGGCGTTGACCTTGGACTCGATGAACTCGTGATAGAG

GTCGTTGCCCTCGCGGGTACGCTCGCCGACGCCGGCGAACACCGAGTAGCCGCCGTGCGC

CTTGGCGACGTTGTTGATCAGCTCCTGAATCAGCACGGTCTTGCCGACGCCGGCGCCGCC

GAACAGGCCGATCTTGCCGCCCTTGGCGTAGGGCGCGAGCAGGTCGACGACCTTGATGCC

GGTGACGAGAATTTCAGCTTCGGTCGACTGGTCGGTATAGGTCGGCGCTTCCTGGTGGAT

GGCGCGAGTGCCCTCCGACTTGATCGGCCCGGCCTCGTCGATCGGCTCGCCGATGACGTT

CATGATGCGGCCCAGCGTACCGTCACCCACGGGCACCGAGATCGGCTGACCGGTGTCGGT

CACTTCCTCGC

>Seq8 [organism=Bradyrhizobium sp. TUTVUMp60] [strain=TUTVUMp60] ATP synthase beta subunit (*atpD*) gene, partial cds,

CGACGAAGAACAGCACGTCCTGGCCCTGGTCGCGGAAGTGTCCGGCAACGG

TGAGACCGGTCAGACCGACGCGGGCGCGGGCGCCCGGCGGCTCGTTCATCTGGCCGAACA

CCAGCGCGCACTTCGACTTCACGCTCGGATCCGGATTGTGCGGATCGGCGTTGACCTTCG

ATTCGATGAACTCGTGATAGAGGTCATTGCCTTCGCGGGTGCGCTCGCCGACGCCGGCGA

ACACCGAGTAACCACCGTGCGCCTTCGCGACGTTGTTGATGAGCTCCTGAATCAGCACGG

TCTTGCCGACGCCGGCGCCGCCGAACAGGCCGATCTTGCCGCCCTTCGCATACGGAGCAA

GAAGGTCGACGACCTTGATGCCGGTGACGAGAATTTCGGCTTCGGTCGACTGGTCGGTAT

AGGTCGGCGCTTCCTGATGGATGGCGCGCAGGCCGTCGGACTTGATCGGGCCTGCTTCAT

CGATCGGCTCGCCGATGACGTTGATGATGCGGCCGAGCGTGCCTTCGCCGACCGGCACCC

GGATCGGTTCGCCGGTGTCGGTCACTTCC

>Seq9 [organism=Bradyrhizobium sp. TUTVUMp65] [strain=TUTVUMp65] ATP synthase beta subunit (*atpD*) gene, partial cds,

CACGTCCTGGCCCTGGTCGCGGAAGTGCTCGGCGACGGTCAGACCGGTGAGGCCGAC

GCGGGCGCGGGCGCCCGGCGGCTCGTTCATCTGGCCGAACACCAGCGCGCATTTCGACTT

GACGCTCGGATCCGGATTGTGCGGATCGGCGTTGACCTTGGACTCGATGAATTCGTGATA

GAGGTCGTTGCCCTCGCGGGTACGCTCGCCGACGCCGGCGAACACCGAGTAACCACCGTG

CGCCTTGGCGACGTTGTTGATCAGTTCCTGAATCAGCACGGTCTTGCCGACGCCAGCGCC

ACCGAACAGGCCGATCTTGCCGCCCTTGGCGTAGGGCGCGAGCAGGTCGACGACTTTGAT

GCCCGTGACGAGAATTTCAGCTTCGGTCGACTGGTCGGTATAGGTCGGCGCTTCCTGGTG

GATGGCGCGAGTGCCCTCCGACTTGATCGGGCCAGCCTCGTCGATCGGCTCGCCGATGAC

GTTCATGATGCGGCCCAGCGTGCCGTCACCCACGGGCACCGAGATCGGCTGACCGGTGTC

AGTCACTTC

>Seq10 [organism=Bradyrhizobium sp. TUTVUMp75] [strain=TUTVUMp75] ATP synthase beta subunit (*atpD*) gene, partial cds,

GTTGTCGACGAAGAACAGCACGTCCTGGCCCTGGTCGCGGAAGTGCTCGGCAACGGTG

AGACCGGTCAGACCGACGCGGGCGCGGGCGCCCGGCGGCTCGTTCATCTGGCCGAACAC

CAGCGCGCACTTCGACTTCACGCTCGGATCCGGATTGTGCGGATCGGCGTTGACCTTCG

ATTCGATGAACTCGTGATAGAGGTCATTGCCTTCGCGGGTGCGCTCGCCGACGCCGGCG

AACACCGAGTAACCACCGTGCGCCTTCGCGACGTTGTTGATGAGCTCCTGAATCAGCAC

GGTCTTGCCGACGCCGGCGCCGCCGAACAGGCCGATCTTGCCGCCCTTCGCATACGGAG

CAAGAAGGTCGACGACCTTGATGCCGGTGACGAGAATTTCGGCTTCGGTCGACTGGTCG

GTATAGGTCGGCGCTTCCTGATGGATGGCGCGCAGGCCGTCGGACTTGATCGGGCCGGC

TTCATCGATCGGCTCGCCGATGACGTTGATGATGCGGCCGAGCGTGCCTTCGCCGACCG

GCACCCGGATCGGTTCGCCGGTGTCGGTC

>Seq11 [organism=Bradyrhizobium sp. TUTVUMr80] [strain=TUTVUMr80] ATP synthase beta subunit (*atpD*) gene, partial cds,

TCGACGAAGAACAGCACGTCCTGGCCCTGGTCGCGGAAGTGCTCGGCGACGGTGAGACCG

GTGAGGCCGACGCGGGCGCGGGCGCCGGGCGGCTCGTTCATCTGGCCGAACACCAGCGCGC

ATTTCGACTTCACGCTCGGATCCGGGTTGTTCGGATCGGCGTTGACCTTGGACTCGATGAA

CTCGTGATAGAGGTCGTTGCCCTCGCGGGTCCGCTCGCCGACGCCGGCGAACACGGAGTAA

CCGCCGTGCGCCTTCGCGACGTTGTTGATCAGCTCCTGAATCAGCACGGTCTTGCCGACGC

CGGCGCCGCCGAACAGGCCGATCTTGCCGCCCTTCGCATACGGAGCAAGAAGGTCGACGAC

CTTGATCCCGGTGACGAGAATTTCAGCCTCGGTGGACTGGTCGGTATAGGTCGGCGCTTCC

TGGTGGATGGCGCGGATGCCTTCCGACTTGATCGGGCCGGCCTCGTCGATCGGCTCGCCGA

TGACGTTCATGATGCGGCCCAGCGTACCGTCACCCACGGGAACGGAGATCGGCTGACCGGT

GTCGGTCACTTCCTGGCCGCGGACCAGA

>Seq12 [organism=Bradyrhizobium sp. TUTVUMr82] [strain=TUTVUMr82] ATP synthase beta subunit (*atpD*) gene, partial cds,

TCGACGAAGAACAGCACGTCCTGGCCCTGGTCGCGGAAATGCTCGGCGACCGTCAGGCCG

GTCAGACCGACGCGGGCACGGGCGCCCGGCGGCTCGTTCATCTGGCCGAACACCAGCG

CGCACTTCGACTTCACGCTCGGATCCGGATTGTGCGGATCGGCGTTGACCTTCGATTC

GATGAACTCGTGATAGAGGTCGTTGCCCTCGCGGGTGCGCTCGCCGACGCCGGCGAAC

ACGGAGTAACCGCCGTGCGCCTTCGCGACGTTGTTGATCAGCTCCTGAATCAGCACGG

TCTTGCCGACGCCGGCGCCGCCGAACAGGCCGATCTTGCCGCCCTTCGCGTACGGAGC

AAGCAGGTCGACGACCTTGATGCCGGTGACGAGAATTTCGGCTTCGGTGGACTGATCG

GTATAGGTCGGCGCTTCCTGGTGGATGGCGCGCACGCCGTCGGCCTTGATCGGACCGG

CTTCGTCGATCGGCTCGCCGATCACGTTGATGATGCGGCCGAGCGTGCCTTCGCCGAC

CGGCACCCGGATCGGTTCGCCGGTGTCGGTCACTTCCTGG

>Seq13 [organism=Bradyrhizobium sp. TUTVUMr84] [strain=TUTVUMr84] ATP synthase beta subunit (*atpD*) gene, partial cds,

GTTGTCGACGAAGAACAGCACGTCCTGGCCCTGGTCGCGGAAGTGCTCGGCGA

CCGTCAGACCGGTCAGGCCGACGCGGGCGCGCGCGCCCGGCGGCTCGTTCATCTGACCG

AACACCAGCGCGCACTTCGACTTCACGCTCGGATCCGGATTGTGCGGGTCGGCGTTGACC

TTGGACTCGATGAACTCGTGATAGAGGTCGTTGCCCTCGCGGGTACGCTCGCCGACGCCG

GCGAACACGGAGTAACCGCCGTGCGCCTTGGCGACGTTGTTGATCAGCTCCTGAATCAGC

ACGGTCTTGCCGACGCCGGCGCCGCCGAACAGGCCGATCTTGCCGCCCTTCGCATATGGA

GCGAGCAGATCGACGACCTTGATGCCGGTGACGAGAATTTCGGCTTCGGTCGACTGGTCG

GTATAGGTCGGCGCTTCCTGGTGGATGGCGCGGGTGCCTTCCGACTTGATCGGGCCGGCC

TCGTCGATCGGCTCGCCGATGACGTTCATGATGCGGCCCAGCGTACCGTCACCCACGGGC

ACCGAAATCGGCTGGCCGGTGTCGGTCA

>Seq14 [organism=Bradyrhizobium sp. TUTVUMr85] [strain=TUTVUMr85] ATP synthase beta subunit (*atpD*) gene, partial cds,

TGTTGTCGACGAAGAACAGCACGTCCTGGCCTTGGTCGCGGAAGTGCTCGGCGA

CGGTGAGACCAGTGAGGCCGACGCGGGCGCGGGCGCCGGGCGGCTCGTTCATCTGGCCG

AACACCAGCGCGCATTTCGACTTCACGCTCGGATCCGGGTTCTTCGGATCGGCGTTGACC

TTGGACTCGATGAACTCGTGATAGAGGTCGTTGCCCTCGCGGGTGCGCTCGCCGACGCCG

GCGAACACGGAGTAACCGCCGTGCGCCTTCGCGACGTTGTTGATCAGCTCCTGAATCAGC

ACGGTCTTGCCGACACCGGCGCCGCCGAACAGGCCGATCTTGCCGCCCTTCGCATACGGA

GCAAGAAGGTCGACGACCTTGATCCCGGTGACGAGAATTTCAGCCTCGGTGGACTGGTCG

GTATAGGTCGGCGCTTCCTGGTGGATGGCGCGGCTGCCTTCCGACTTGATCGGGCCGGCC

TCGTCGATCGGCTCGCCGATGACGTTCATGATGCGGCCCAGCGTACCGTCACCCAC

>Seq15 [organism=Bradyrhizobium sp. TUTVUMr89] [strain=TUTVUMr89] ATP synthase beta subunit (*atpD*) gene, partial cds,

CACGTCCTGGCCCTGGTCGCGGAAGTGCTCGGCGACGGTCAGACCGGTGAGGCCGACGCG

GGCGCGGGCGCCCGGCGGCTCGTTCATCTGGCCGAACACCAGCGCGCATTTCGACTTGAC

GCTCGGATCCGGATTGTGCGGATCGGCGTTGACCTTGGACTCGATGAACTCGTGATAGAG

GTCGTTGCCCTCGCGGGTACGCTCGCCGACGCCGGCGAACACCGAGTAACCACCATGCGC

CTTGGCGACGTTGTTGATCAGTTCCTGAATCAGCACGGTCTTGCCGACGCCGGCGCCACC

GAACAGGCCGATCTTGCCGCCCTTGGCGTAGGGCGCGAGCAAGTCGACGACCTTGATGCC

CGTGACGAGAATTTCAGCTTCGGTCGACTGGTCGGTATAGGTCGGCGCTTCCTGGTGGAT

GGCGCGAGTGCCCTCCGACTTGATCGGGCCAGCCTCGTCGATCGGCTCGCCGATGACGTT

CATGATGCGGCCCAGCGTACCGTCACCCACGGGCACCGAGATCGGCTGACCGGTGTCGGT

CACTTCCTGG

>Seq16 [organism=Bradyrhizobium sp. TUTVUMr93] [strain=TUTVUMr93] ATP synthase beta subunit (*atpD*) gene, partial cds,

GATGTTGTCGACGAAGAACAGCACGTCCTGGCCCTGGTCGCGGAAGTGCTCGGCGACGG

TGAGACCAGTGAGGCCGACGCGGGCGCGGGCGCCGGGCGGCTCGTTCATCTGGCCGA

ACACCAGCGCGCATTTCGACTTCACGCTCGGATCCGGGTTCTTCGGATCGGCGTTGA

CCTTGGACTCGATGAACTCGTGATAGAGGTCGTTGCCCTCGCGGGTGCGCTCGCCGA

CGCCGGCGAACACGGAGTAACCGCCGTGCGCCTTCGCGACGTTGTTGATCAGCTCCT

GAATCAGCACGGTCTTGCCGACACCGGCGCCGCCGAACAGGCCGATCTTGCCGCCCTT

CGCATACGGAGCAAGAAGGTCGACGACCTTGATCCCGGTGACGAGAATTTCAGCCTCG

GTGGACTGGTCGGTATAGGTCGGCGCTTCCTGGTGGATGGCGCGGCTGCCTTCCGACT

TGATCGGGCCGGCCTCGTCGATCGGCTCGCCGATGACGTTCATGATGCGGCCCAGCGT

ACCGTCACCCAC

>Seq17 [organism=Bradyrhizobium sp. TUTVUMr94] [strain=TUTVUMr94] ATP synthase beta subunit (*atpD*) gene, partial cds,

CGACGAAGAACAGCACGTCCTGGCCCTGGTCGCGGAAGTGCTCGGCGACAGTCAGGC

CGGTCAGGCCGACGCGGGCGCGGGCGCCCGGCGGCTCGTTCATCTGACCGAACACGAGAG

CGCACTTCGACTTCACGCTCGGATCCGGATTGTGCGGATCGGCGTTGACCTTGGACTCGA

TGAACTCGTGATAGAGGTCGTTGCCCTCGCGGGTACGCTCGCCGACGCCGGCGAACACGG

AGTAACCACCGTGCGCCTTCGCGACGTTGTTGATCAGCTCCTGAATCAGCACGGTCTTGC

CGACGCCGGCGCCGCCGAACAGGCCGATCTTGCCGCCCTTCGCATACGGAGCGAGCAGAT

CGACGACCTTGATGCCGGTGACGAGAATTTCGGCTTCGGTCGACTGGTCGGTATAGGTCG

GCGCTTCCTGGTGGATGGCGCGGATGCCTTCCGACTTGATCGGGCCGGCCTCGTCGATCG

GCTCGCCGATGACGTTCATGATGCGGCCGAGCGTACCGTCACCCACGGGAACCGAGATCG

GCTGACCGGTGTCGGTCACTTC

>Seq18 [organism=Bradyrhizobium sp. TUTVUMr95] [strain=TUTVUMr95] ATP synthase beta subunit (*atpD*) gene, partial cds,

GGAAGATGTTGTCGACGAAGAACAGCACGTCCTGGCCCTGGTCGCGGAAGTGCTCGGCGA

CGGTGAGACCAGTGAGGCCGACGCGGGCGCGGGCGCCGGGCGGCTCGTTCATCTGGCC

GAACACCAGCGCGCATTTCGACTTCACGCTCGGATCCGGGTTCTTCGGATCGGCGTTG

ACCTTGGACTCGATGAACTCGTGATAGAGGTCGTTGCCCTCGCGGGTGCGCTCGCCGA

CGCCGGCGAACACGGAGTAACCGCCGTGCGCCTTCGCGACGTTGTTGATCAGCTCCTG

AATCAGCACGGTCTTGCCGACACCGGCGCCGCCGAACAGGCCGATCTTGCCGCCCTTC

GCATACGGAGCAAGAAGGTCGACGACCTTGATCCCGGTGACGAGAATTTCAGCCTCGG

TGGACTGGTCGGTATAGGTCGGCGCTTCCTGGTGGATGGCGCGGCTGCCTTCCGACTT

GATCGGGCCGGCCTCGTCGATCGGCTCGCCGATGACGTTCATGATGCGGCCCAGCGTA

CCGTCACCCAC

>Seq19 [organism=Bradyrhizobium sp. TUTVUMr99] [strain=TUTVUMr99] ATP synthase beta subunit (*atpD*) gene, partial cds,

ACAGCACGTCCTGGCCCTGGTCGCGGAAATGCTCGGCGACCGTCAGGCCGGTCAGACCG

ACGCGGGCACGGGCGCCCGGCGGCTCGTTCATCTGGCCGAACACCAGCGCGCACTTCGAC

TTCACGCTCGGATCCGGATTGTGCGGATCGGCGTTGACCTTCGATTCGATGAACTCGTGA

TAGAGGTCGTTGCCCTCGCGGGTGCGCTCGCCGACGCCGGCGAACACGGAGTAACCGCCG

TGCGCCTTCGCGACGTTGTTGATCAGCTCCTGAATCAGCACGGTCTTGCCGACGCCGGCG

CCGCCGAACAGGCCGATCTTGCCGCCCTTCGCGTACGGAGCAAGCAGGTCGACGACCTTG

ATGCCGGTGACGAGAATTTCGGCTTCGGTGGACTGATCGGTATAGGTCGGCGCTTCCTGG

TGGATGGCGCGCACGCCGTCGGCCTTGATCGGACCGGCTTCGTCGATCGGCTCGCCGATC

ACGTTGATGATGCGGCCGAGCGTGCCTTCGCCGACCGGCACCCGGATCGGTTCGCCGGTG

TCGGTCACTTC

>Seq20 [organism=Bradyrhizobium sp. TUTVUMr103] [strain=TUTVUMr103] ATP synthase beta subunit (*atpD*) gene, partial cds,

TCGACGAAGAACAGCACGTCCTGGCCCTGGTCGCGGAAGTGCTCGGCAACGGTCAGACCGG

TCAGGCCGACGCGGGCGCGGGCGCCCGGCGGCTCGTTCATCTGACCGAACACGAGAGCG

CATTTCGACTTCACGCTCGGATCCGGATTGTGCGGATCGGCGTTGACCTTGGACTCGAT

GAACTCGTGGTAGAGGTCGTTGCCCTCGCGGGTCCGCTCGCCGACGCCGGCGAACACGG

AGTAACCGCCGTGCGCCTTGGCGACGTTGTTGATCAGCTCCTGAATCAGCACGGTCTTG

CCCACGCCGGCGCCGCCGAACAGGCCGATCTTGCCGCCCTTCGCATACGGAGCGAGCAG

ATCGACGACCTTGATGCCGGTGACGAGAATTTCGGCTTCGGTCGACTGGTCGGTATAGG

TCGGCGCTTCCTGGTGGATGGCGCGCATGCCTTCCGACTTGATCGGGCCGGCCTCGTCG

ATCGGCTCGCCGATGACGTTCATGATGCGGCCCAGCGTACCGTCACCCACGGGAACCGA

AATCGGCTGACCGGTGTCGGTCACTTCCTGGCCGGGGGCC

>Seq21 [organism=Bradyrhizobium sp.TUTVUMm105] [strain=TUTVUMm105] ATP synthase beta subunit (*atpD*) gene, partial cds,

CGACGAAGAACAGCACGTCCTGGCCCTGGTCGCGGAAGTGCTCGGCGACAGTCAGGC

CGGTCAGGCCGACGCGGGCGCGGGCGCCCGGCGGCTCGTTCATCTGACCGAACACGAGAG

CGCACTTCGACTTCACGCTCGGATCCGGATTGTGCGGATCGGCGTTGACCTTGGACTCGA

TGAACTCGTGATAGAGGTCGTTGCCCTCGCGGGTACGCTCGCCGACGCCGGCGAACACGG

AGTAACCACCGTGCGCCTTGGCGACGTTGTTGATCAGCTCCTGAATCAGCACGGTCTTGC

CGACGCCGGCGCCGCCGAACAGGCCGATCTTGCCGCCCTTCGCATACGGAGCGAGCAGAT

CGACGACCTTGATGCCGGTGACGAGAATTTCGGCTTCGGTCGACTGGTCGGTATAGGTCG

GCGCTTCCTGGTGGATGGCGCGGATGCCTTCCGACTTGATCGGGCCGGCCTCGTCGATCG

GCTCGCCGATGACGTTCATGATGCGGCCGAGCGTACCGTCACCCACGGGAACCGAGATCG

GCTGACCGGTGTCGGTCACT

>Seq22 [organism=Bradyrhizobium sp. TUTVUMm108] [strain=TUTVUMm108] ATP synthase beta subunit (*atpD*) gene, partial cds,

ACAGCACGTCCTGACCCTGGTCGCGGAAGTCCTCCGCGATGGTCAGACCCGTGAGCG

CGACGCGGGCGCGGGCGCCCGGCGGCTCGTTCATCTGGCCGAACACCAGCGCGCACTTCG

ACTTCACGCTCGGATCCGGATTCTTCGGGTCCGCGTTGACCTTGGACTCGATGAACTCGT

GATAGAGGTCGTTGCCCTCGCGGGTCCGCTCGCCGACGCCGGCGAACACGGAGTAACCGC

CGTGCGCCTTCGCGACGTTGTTGATCAGCTCCTGGATCAGCACGGTCTTGCCGACGCCGG

CGCCGCCGAACAGGCCGATCTTGCCGCCCTTGGCGTAGGGAGCGAGGAGGTCGACGACCT

TGATGCCGGTGACGAGAATTTCAGCTTCCGTGGACTGGTCGGTGTAAGTCGGCGCTTCCT

GGTGGATCGCGCGCAGGCCTTCCGTCTTGACCGGACCGGCTTCGTCGATCGGCTCGCCGA

TGACGTTGATGATGCGGCCGAGCGTGCCCTCGCCGACGGGAACGCGGATCGGAGCGCCGG

TGTCGGTGACTTCCTGCCCGGG

>Seq23 [organism=Bradyrhizobium sp. TUTVUMm110] [strain=TUTVUMm110] ATP synthase beta subunit (*atpD*) gene, partial cds,

TTGTCAACGAAGAACAGCACGTCCTGGCCCTGGTCGCGGAAGTGCTCGGCAACGGTCAGA

CCGGTCAGGCCGACGCGGGCGCGGGCGCCCGGCGGCTCGTTCATCTGACCGAACACGAG

AGCGCATTTCGACTTCACGCTCGGATCCGGATTGTGCGGATCGGCGTTGACCTTGGACT

CGATGAACTCGTGGTAGAGGTCGTTGCCCTCGCGGGTACGCTCGCCGACGCCGGCGAAC

ACGGAGTAACCGCCGTGCGCCTTGGCGACGTTGTTGATCAGCTCCTGAATCAGCACGGT

CTTGCCGACGCCGGCGCCGCCGAACAGGCCGATCTTGCCGCCCTTCGCATACGGAGCGA

GCAGATCGACGACCTTGATGCCGGTGACGAGAATTTCGGCTTCGGTCGACTGGTCGGTG

TAGGTCGGCGCTTCCTGGTGGATGGCGCGCGTGCCTTCCGACTTGATCGGGCCGGCCTC

GTCGATCGGCTCGCCGATGACGTTCATGATGCGGCCCAGCGTACCGTCACCCACGGGAA

CCGAAATCGGCTGACCGGTGTCGGTCA

>Seq24 [organism=Bradyrhizobium sp. TUTVUMm112] [strain=TUTVUMm112] ATP synthase beta subunit (*atpD*) gene, partial cds,

CGACGAAGAACAGCACGTCCTGGCCCTGGTCGCGGAAGTGCTCGGCAACGGTCAGA

CCGGTCAGGCCGACGCGGGCGCGGGCGCCCGGCGGCTCGTTCATCTGACCGAACACGAGA

GCGCATTTCGACTTCACGCTCGGATCCGGATTGTGCGGATCGGCGTTGACCTTGGACTCG

ATGAACTCGTGGTAGAGGTCGTTGCCCTCGCGGGTACGCTCGCCGACGCCGGCGAACACG

GAGTAACCGCCGTGCGCCTTGGCGACGTTGTTGATCAGCTCCTGAATCAGCACGGTCTTG

CCGACGCCGGCGCCGCCGAACAGGCCGATCTTGCCGCCCTTCGCATACGGAGCGAGCAGA

TCGACGACCTTGATGCCGGTGACGAGAATTTCGGCTTCGGTCGACTGGTCGGTGTAGGTC

GGCGCTTCCTGGTGGATGGCGCGCATGCCTTCCGACTTGATCGGGCCGGCCTCGTCGATC

GGCTCGCCGATGACGTTCATGATGCGGCCCAGCGTACCGTCACCCACGGGAACCGAAATC

GGCTGACCGGTGTCGGTCACTTCCTGGCCGCGGACC

>Seq25 [organism=Bradyrhizobium sp. TUTVUMm113] [strain=TUTVUMm113] ATP synthase beta subunit (*atpD*) gene, partial cds,

TCGACGAAGAACAGCACGTCCTGGCCCTGGTCGCGGAAGTGCTCGGCAACGGTCAGACCG

GTCAGGCCGACGCGGGCGCGGGCGCCCGGCGGCTCGTTCATCTGACCGAACACGAGAG

CGCATTTCGACTTCACGCTCGGATCCGGATTGTGCGGATCGGCGTTGACCTTGGACTC

GATGAACTCGTGGTAGAGGTCGTTGCCCTCGCGGGTACGCTCGCCGACGCCGGCGAAC

ACGGAGTAACCGCCGTGCGCCTTGGCGACGTTGTTGATCAGCTCCTGAATCAGCACGG

TCTTGCCGACGCCGGCGCCGCCGAACAGGCCGATCTTGCCGCCCTTCGCATACGGAGC

GAGCAGATCGACGACCTTGATGCCGGTGACGAGAATTTCGGCTTCGGTCGACTGGTCG

GTGTAGGTCGGCGCTTCCTGGTGGATGGCGCGCATGCCTTCCGACTTGATCGGGCCGG

CCTCGTCGATCGGCTCGCCGATGACGTTCATGATGCGGCCCAGCGTACCGTCACCCAC

GGGAACCGAAATCGGCTGACCGGTGTCGGTCACTTCCTGGCCGCGAACCAGA

>Seq26 [organism=Bradyrhizobium sp. TUTVUMm114] [strain=TUTVUMm114] ATP synthase beta subunit (*atpD*) gene, partial cds,

GATGTTGTCGACGAAGAACAGCACGTCCTGGCCCTGGTCGCGGAAGTGCTCGGCAACGGT

CAGACCGGTCAGGCCGACGCGGGCGCGGGCGCCCGGCGGCTCGTTCATCTGACCGAAC

ACGAGAGCGCATTTCGACTTCACGCTCGGATCCGGATTGTGCGGATCGGCGTTGACCT

TGGACTCGATGAACTCGTGGTAGAGGTCGTTGCCCTCGCGGGTACGCTCGCCGACGCC

GGCGAACACGGAGTAACCGCCGTGCGCCTTGGCGACGTTGTTGATCAGCTCCTGAATC

AGCACGGTCTTGCCGACGCCGGCGCCGCCGAACAGGCCGATCTTGCCGCCCTTCGCAT

ACGGAGCGAGCAGATCGACGACCTTGATGCCGGTGACGAGAATTTCGGCTTCGGTCGA

CTGGTCGGTGTAGGTCGGCGCTTCCTGGTGGATGGCGCGCGTGCCTTCCGACTTGATC

GGGCCGGCCTCGTCGATCGGCTCGCCGATGACGTTCATGATGCGGCCCAGCGTACCGT

CACCCACGGGAACCGAAATCGGCTGACCGGTGTCGGTCA

>Seq27 [organism=Bradyrhizobium sp. TUTVUMm115] [strain=TUTVUMm115] ATP synthase beta subunit (*atpD*) gene, partial cds,

TCGACGAAGAACAGCACGTCCTGGCCCTGGTCGCGGAAGTGCTCGGCAACGGTCAGACCG

GTCAGGCCGACGCGGGCGCGGGCGCCCGGCGGCTCGTTCATCTGACCGAACACGAGAGCG

CATTTCGACTTCACGCTCGGATCCGGATTGTGCGGATCGGCGTTGACCTTGGACTCGATG

AACTCGTGGTAGAGGTCGTTGCCCTCGCGGGTACGCTCGCCGACGCCGGCGAACACGGAG

TAACCGCCGTGCGCCTTGGCGACGTTGTTGATCAGCTCCTGAATCAGCACGGTCTTGCCG

ACGCCGGCGCCGCCGAACAGGCCGATCTTGCCGCCCTTCGCATACGGAGCGAGCAGATCG

ACGACCTTGATGCCGGTGACGAGAATTTCGGCTTCGGTCGACTGGTCGGTGTAGGTCGGC

GCTTCCTGGTGGATGGCGCGCATGCCTTCCGACTTGATCGGGCCGGCCTCGTCGATCGGC

TCGCCGATGACGTTCATGATGCGGCCCAGCGTACCGTCACCCACGGGAACCGAAATCGGC

TGACCGGTGTCGGTCACTTCC

>Seq28 [organism=Bradyrhizobium sp. TUTVUMm116] [strain=TUTVUMm116] ATP synthase beta subunit (*atpD*) gene, partial cds,

TCGACGAAGAACAGCACGTCCTGGCCCTGGTCGCGGAAGTGCTCGGCAACGGTCAGAC

CGGTCAGGCCGACGCGGGCGCGGGCGCCCGGCGGCTCGTTCATCTGACCGAACACGAG

AGCGCATTTCGACTTCACGCTCGGATCCGGATTGTGCGGATCGGCGTTGACCTTGGAC

TCGATGAACTCGTGGTAGAGGTCGTTGCCCTCGCGGGTACGCTCGCCGACGCCGGCGA

ACACGGAGTAACCGCCGTGCGCCTTGGCGACGTTGTTGATCAGCTCCTGAATCAGCAC

GGTCTTGCCGACGCCGGCGCCGCCGAACAGGCCGATCTTGCCGCCCTTCGCATACGGA

GCGAGCAGATCGACGACCTTGATGCCGGTGACGAGAATTTCGGCTTCGGTCGACTGGT

CGGTGTAGGTCGGCGCTTCCTGGTGGATGGCGCGCATGCCTTCCGACTTGATCGGGCC

GGCCTCGTCGATCGGCTCGCCGATGACGTTCATGATGCGGCCCAGCGTACCGTCACCC

ACGGGAACCGAAATCGGCTGACCGGTGTCGGTCACTTCCTGGCCGCGGACC

>Seq29 [organism=Bradyrhizobium sp. TUTVUMm117] [strain=TUTVUMm117] ATP synthase beta subunit (*atpD*) gene, partial cds,

ATGTTGTCGACGAAGAACAGCACGTCCTGGCCCTGGTCGCGGAAGTGCTCGGCAACGGTG

AGACCGGTCAGACCGACGCGGGCGCGGGCGCCCGGCGGCTCGTTCATCTGGCCGAACA

CCAGCGCGCACTTCGACTTCACGCTCGGATCCGGATTGTGCGGATCGGCGTTGACCTT

CGATTCGATGAACTCGTGATAGAGGTCATTGCCTTCGCGGGTGCGCTCGCCGACGCCG

GCGAACACCGAGTAACCACCGTGCGCCTTCGCGACGTTGTTGATCAGCTCCTGAATCA

GCACGGTCTTGCCGACGCCGGCGCCGCCGAACAGGCCGATCTTGCCGCCCTTGGCGTA

CGGAGCAAGAAGGTCGACGACCTTGATGCCGGTGACGAGAATTTCGGCCTCGGTCGAC

TGGTCGGTATAGGTCGGCGCTTCCTGATGGATGGCGCGCAGGCCGTCGGACTTGATCG

GGCCTGCTTCATCGATCGGCTCGCCGATGACGTTGATGATGCGGCCGAGCGTGCCTTC

GCCGACCGGCACCCGGATCGGTTCGCCGGTGTCGGTC

>Seq30 [organism=Bradyrhizobium sp. TUTVUI122] [strain=TUTVUI122] ATP synthase beta subunit (*atpD*) gene, partial cds,

CGACGAAGAACAGCACGTCCTGACCTTGGTCGCGGAAGTCTTCCGCGATGGTCAGA

CCCGTGAGCGCGACGCGGGCGCGGGCGCCCGGCGGCTCGTTCATCTGGCCGAACACCAGC

GCGCACTTCGACTTCACGCTCGGATCCGGATTCTTCGGGTCCGCGTTGACCTTGGACTCG

ATGAACTCGTGATAGAGGTCGTTGCCCTCGCGGGTCCGCTCGCCGACGCCGGCGAACACG

GAGTAACCGCCGTGCGCCTTCGCGACGTTGTTGATCAGCTCCTGGATCAGCACGGTCTTG

CCGACGCCGGCGCCGCCGAACAGGCCGATCTTGCCGCCCTTGGCGTAGGGAGCGAGGAGG

TCGACGACCTTGATGCCGGTGACGAGAATTTCAGCTTCCGTGGACTGGTCGGTGTAAGTC

GGCGCTTCCTGGTGGATCGCGCGCAGGCCTTCCGTCTTGACCGGACCGGCTTCGTCGATC

GGCTCGCCGATGACGTTGATGATGCGGCCGAGCGTGCCCTCGCCGACGGGAACGCGGATC

GGAGCGCCGGTGTCGGTGACT

***glnII***

>Seq1 [organism=Bradyrhizobium sp. TUTVUMp5] [strain=TUTVUMp5] *glnII* gene for glutamine synthetase II, partial cds

TTGCGCGGCAAAACTCAGATCAAGGAATTCGCGTCGTTCCCGACGCTCGAGCAGCTTCCG

CTCTGGGGCTTCGATGGCTCCTCCACCCAGCAGGCCGAAGGTCATAGCTCCGATTGCGT

GCTGAAGCCGGTCGCCGTCTTCCCGGACGCCGCGCGCACCAATGGCGTGCTTGTGATGT

GCGAAGTCATGATGCCCGATGGCAAGACCCCGCATCCGTCAAACAAGCGCGCCACCATC

CTCGACGACGCCGGCGCCTGGTTCGGCTTCGAGCAGGAATACTTCTTCTACAAGGACGG

CCGCCCGCTCGGCTTCCCGACTGCCGGCTATCCGGCCCCGCAGGGTCCGTACTACACCG

GCGTCGGCTACTCGAATGTCGGCGACGTCGCCCGCAAGATCGTCGAAGAGCATCTCGAC

CTCTGCCTCGCTGCCGGCATCAACCATGAAGGCATCAACGCGGAAGTCGCCAAGGGTCA

GTGGGAATTCCAGATCTTCGGCAAGGGCTCCAAGACCGCTGCCGACCAGATGTGGATGG

CTCGCTACCTGATGCTGCGCCTCACCGAGAAGTACGGCATCGACATCGAGTTCCACTGC

AAGCCGCTCGGCGAC

>Seq2 [organism=Bradyrhizobium sp. TUTVUMp6] [strain=TUTVUMp6] *glnII* gene for glutamine synthetase II, partial cds

AACTCAGATCAAGGAATTCGCGTCGTTCCCGACGCTCGAGCAGCTTC

CGCTCTGGGGCTTCGACGGCTCCTCCACCCAGCAGGCCGAAGGCCACAGCTCCGATTGCG

TGCTGAAGCCGGTCGCGGTGTTTCCGGACGGTGCGCGCACCAACGGCGTGCTGGTGATGT

GCGAAGTCATGATGCCCGATGGCAAGACCCCGCATCCGTCCAACAAGCGCGCCACCATCC

TTGACGATTCCGGCGCCTGGTTCGGCTTCGAGCAGGAATACTTCTTCTACAAGGACGGCC

GTCCGCTCGGCTTCCCGACCGCCGGCTATCCCGCGCCGCAAGGCCCGTACTACACCGGCG

TCGGCTACTCAAATGTCGGCGACGTCGCCCGCAAGATCGTCGAAGAGCACCTCGATCTCT

GCCTCGCCGCCGGCATCAACCATGAAGGCATCAACGCGGAAGTCGCCAAGGGTCAGTGGG

AATTCCAGATCTTCGGCAAGGGCTCCAAGACCGCTGCCGACCAGATGTGGATGGCCCGCT

ACCTGATGCTGCGCCTCACCGAGAAGTACGGCATCGACATCGAATTCCACTGCAAGCCGC

T

>Seq3 [organism=Bradyrhizobium sp. TUTVUMp8] [strain=TUTVUMp8] *glnII* gene for glutamine synthetase II, partial cds

GCCGACTCCGAATTTGCGCGGCAAAACTCAGATCAAGGAATTCGCGTCGTTCCCGAC

GCTCGAGCAGCTTCCGCTCTGGGGCTTCGATGGCTCTTCCACCCAGCAGGCCGAAGGT

CATAGCTCTGATTGCGTGCTGAAGCCGGTCGCCGTCTTCCCGGACGCCGCGCGCACCAA

TGGCGTGCTCGTGATGTGCGAAGTCATGATGCCCGATGGCAAGACCCCGCATCCGTCAA

ACAAGCGCGCCACCATCCTCGACGACGCCGGCGCATGGTTCGGCTTCGAGCAGGAATACT

TCTTCTACAAGGACGGCCGCCCGCTCGGCTTCCCGACTGCCGGCTATCCGGCCCCGCAGG

GTCCGTACTACACCGGCGTCGGCTACTCGAACGTCGGCGACGTCGCCCGCAAGATCGTCG

AAGAGCATCTCGACCTCTGCCTCGCTGCCGGCATCAACCATGAAGGCATCAACGCGGAAG

TCGCGAAGGGCCAGTGGGAATTCCAGATCTTCGGCAAGGGCTCCAAGACCGCTGCCGACC

AGATGTGGATGGCCCGCTACCTGATGCTGCGCCTCACCGAGAAGTACGGTATCGACATCG

AGTTCCACTGCACGCTCGGCGAC

>Seq4 [organism=Bradyrhizobium sp. TUTVUMp48] [strain=TUTVUMp48] *glnII* gene for glutamine synthetase II, partial cds

GCCGACTCCGAATTTGCGCGGCAAAACTCAGATCAAGGAATTCGCGTCGTTCCC

GACGCTCGAGCAGCTTCCGCTCTGGGGCTTCGATGGCTCCTCCACCCAGCAGGCC

GAAGGTCATAGCTCCGATTGCGTGCTGAAGCCGGTCGCCGTTTTCCCGGACGCCGCGC

GCACCAATGGCGTGCTCGTGATGTGCGAAGTCATGATGCCCGATGGCAAGACCCCGCA

TCCGTCCAACAAGCGCGCCACCATCCTCGATGACGCCGGCGCCTGGTTCGGCTTCGAG

CAGGAGTACTTCTTCTACAAGGACGGCCGCCCGCTCGGCTTCCCGACTGCCGGCTATC

CGGCCCCGCAGGGTCCGTACTACACCGGCGTCGGCTACTCGAATGTCGGCGACGTCGC

CCGCAAGATCGTCGAAGAGCATCTCGACCTCTGCCTCGCTGCCGGCATCAACCATGAA

GGCATCAACGCGGAAGTCGCGAAGGGCCAGTGGGAATTCCAGATCTTCGGCAAGGGCT

CCAAGACCGCTGCCGACCAGATGTGGATGGCCCGCTACCTGATGCTGCGCCTCACCGA

GAAGTACGGCATCGACATCGAGTTCCACTGCAAGCCGCTCGGCGAC

>Seq5 [organism=Bradyrhizobium sp. TUTVUMp50] [strain=TUTVUMp50] *glnII* gene for glutamine synthetase II, partial cds

TACGCCGACTCCGAATTTGCGCGGCAAAACTCAGATCAAGGAATTCGCGTCGTTCCC

GACGCTCGAGCAGCTTCCGCTCTGGGGCTTCGATGGCTCTTCCACCCAGCAGGCCGAAG

GTCACAGCTCTGATTGCGTGCTGAAGCCCGTCGCCGTCTTCCCGGACGCCGCGCGCACC

AATGGCGTGCTCGTGATGTGCGAAGTCATGATGCCCGATGGCAAGACCCCGCATCCGTC

GAACAAGCGCGCCACCATCCTCGACGACGCCGGCGCATGGTTCGGCTTCGAGCAGGAAT

ACTTCTTCTACAAGGACGGCCGCCCGCTCGGCTTCCCGACAGCCGGCTATCCCGCTCCG

CAGGGTCCGTACTACACCGGCGTCGGCTACTCGAACGTCGGCGACGTCGCCCGCAAAAT

CGTCGAAGAGCATCTCGATCTCTGCCTCGCTGCCGGCATCAACCATGAAGGCATCAACG

CCGAAGTGGCGAAGGGCCAGTGGGAATTCCAGATCTTCGGCAAGGGCTCCAAGACCGCT

GCCGACCAGATGTGGATGGCTCGCTACCTGATGCTGCGTCTCACCGAGAAGTATGGTAT

CGACATCGAATTCCATTGCACGCTCGGCGACACCGACTGGAACGG

>Seq6 [organism=Bradyrhizobium sp.TUTVUMp53] [strain=TUTVUMp53] *glnII* gene for glutamine synthetase II, partial cds

GCCGACTCCGAATTTGCGCGGCAAAACTCAGATCAAGGAATTCGCGTCGTTCCCGA

CGCTCGAGCAGCTTCCGCTCTGGGGCTTCGATGGCTCTTCCACCCAGCAGGCCGAAG

GTCATAGCTCTGATTGCGTGCTGAAGCCGGTCGCCGTCTTCCCGGACGCCGCGCGCACC

AATGGCGTGCTCGTGATGTGCGAAGTCATGATGCCCGATGGCAAGACCCCGCATCCGTC

AAACAAGCGCGCCACCATCCTCGACGACGCCGGCGCATGGTTCGGCTTCGAGCAGGAAT

ACTTCTTCTACAAGGACGGCCGCCCGCTCGGCTTCCCGACTGCCGGCTATCCGGCCCCG

CAGGGTCCGTACTACACCGGCGTCGGCTACTCGAACGTCGGCGACGTCGCCCGCAAGAT

CGTCGAAGAGCATCTCGACCTCTGCCTCGCTGCCGGCATCAACCATGAAGGCATCAACG

CGGAAGTCGCGAAGGGCCAGTGGGAATTCCAGATCTTCGGCAAGGGCTCCAAGACCGCT

GCCGACCAGATGTGGATGGCCCGCTACCTGATGCTGCGCCTCACCGAGAAGTACGGTAT

CGACATCGAGTTCCACTGCACGCTCGGCGACACC

>Seq7 [organism=Bradyrhizobium sp. TUTVUMp56] [strain=TUTVUMp56] *glnII* gene for glutamine synthetase II, partial cds

GCCGACTCCGAATTTGCGCGGCAAAACTCAGATCAAGGAATTCGCGTCGTTCCCGAC

GCTCGAGCAGCTTCCGCTCTGGGGCTTCGATGGCTCCTCCACCCAGCAGGCCGAAGGTCA

TAGCTCCGATTGCGTGCTGAAGCCGGTCGCCGTTTTCCCGGACGCCGCGCGCACCAATGG

CGTGCTCGTGATGTGCGAAGTCATGATGCCCGATGGCAAGACCCCGCATCCGTCCAACAA

GCGCGCCACCATCCTCGACGACGCCGGCGCCTGGTTCGGCTTCGAGCAGGAATACTTCTT

CTACAAGGACGGCCGCCCGCTCGGCTTCCCGACTGCCGGCTATCCGGCCCCGCAGGGTCC

GTACTACACCGGCGTCGGCTACTCGAATGTCGGCGACGTCGCCCGCAAGATCGTCGAAGA

GCATCTCGACCTCTGCCTCGCTGCCGGCATCAACCATGAAGGCATCAACGCGGAAGTCGC

CAAGGGTCAGTGGGAATTCCAGATCTTCGGCAAGGGCTCCAAGACCGCTGCCGACCAGAT

GTGGATGGCCCGCTACCTGATGCTGCGCCTCACCGAGAAGTACGGCATCGACATCGAGTT

CCACTGCAAGCCGCTCGGCGACACCGACTGGAACGG

>Seq8 [organism=Bradyrhizobium sp. TUTVUMp60] [strain=TUTVUMp60] *glnII* gene for glutamine synthetase II, partial cds

TACGCCGACGCCGAGCCTGCGCGGTAAGACGCAGATCAAGGAATTCGCGTCGTTCCCGAC

CCTCGAACAGCTTCCGCTGTGGGGCTTTGACGGTTCGTCCACCCAGCAGGCTGAAGGCCA

CAGCTCTGACTGCGTGCTGAAGCCGGTCGCCTGCTATCCCGACGCCGCGCGCGAGAACGG

CGTGCTGGTGATGTGCGAAGTCATGATGCCCGACGGCAAGACGCCGCATGTCTCGAACAA

GCGCGCCACCGTTCTGGATGACGAAGGCGCCTGGTTCGGCTTCGAGCAGGAATACTTCTT

CTACAAGGACGGCCGTCCGCTCGGCTTTCCGGAAGAGGGTTATCCGGCGCCGCAGGGCCC

GTACTACACCGGCGTCGGCTACAAGAACGTCGGCAGCGTCGCCCGCAAGATCGTCGAAGA

GCATCTCAATCTCTGCCTCGCCGCCGGCATCAACCACGAAGGCATCAACGCCGAAGTGGC

GAAGGGCCAGTGGGAATTCCAGATCTTCGGCAAGGGCTCCAAGACCGCCGCTGACCAGAT

GTGGATGGCCCGCTACCTGATGCTGCGCCTCACCGAGAGCTACGGCATCGATATCGAATT

CCATTGCAAGCCGCTCGGCGACACC

>Seq9 [organism=Bradyrhizobium sp. TUTVUMp65] [strain=TUTVUMp65] *glnII* gene for glutamine synthetase II, partial cds

CCGACTCCGAACTTGCGCGGCAAAACTCAGATCAAGGAATTCGCGTCGTTCCCGACG

CTCGAGCAGCTTCCGCTCTGGGGCTTCGATGGCTCTTCCACCCAGCAGGCCGAAGGTCA

TAGCTCTGATTGCGTGCTGAAGCCGGTCGCCGTCTTCCCGGACGCCGCGCGCACCAATGG

CGTGCTCGTGATGTGCGAAGTCATGATGCCCGATGGCAAGACCCCGCATCCGTCAAACAA

GCGCGCCACCATCCTCGACGACGCCGGCGCATGGTTCGGCTTCGAGCAGGAATACTTCTT

CTACAAGGACGGCCGCCCGCTCGGCTTCCCGACTGCCGGCTATCCGGCCCCGCAGGGTCC

GTACTACACCGGCGTCGGCTACTCGAACGTCGGCGACGTCGCCCGCAAGATCGTCGAAGA

GCATCTCGACCTCTGCCTCGCTGCCGGCATCAACCATGAAGGCATCAACGCGGAAGTCGC

GAAGGGCCAGTGGGAATTCCAGATCTTCGGCAAGGGCTCCAAGACCGCTGCCGACCAGAT

GTGGATGGCCCGCTACCTGATGCTGCGCCTCACCGAGAAGTACGGTATCGACATCGAGTT

CCACTGCACGCTCGGCGACACCGCG

>Seq10 [organism=Bradyrhizobium sp. TUTVUMp75] [strain=TUTVUMp75] *glnII* gene for glutamine synthetase II, partial cds

GCCGACGCCGAGCCTGCGCGGTAAGACGCAGATCAAGGAATTCGCGTCGTTCCCGACCCT

CGAACAGCTTCCGCTGTGGGGCTTTGACGGTTCGTCCACCCAGCAGGCTGAAGGCCACAG

CTCTGACTGCGTGCTGAAGCCGGTCGCCTGCTATCCCGACGCCGCGCGCGAGAACGGCGT

GCTGGTGATGTGCGAAGTCATGATGCCCGACGGCAAGACGCCGCATGTCTCGAACAAGCG

CGCCACCGTTCTGGATGACGAAGGCGCCTGGTTCGGCTTCGAGCAGGAATACTTCTTCTA

CAAGGACGGCCGTCCGCTCGGCTTTCCGGAAGAGGGTTATCCGGCGCCGCAGGGCCCGTA

CTACACCGGCGTCGGCTACAAGAACGTCGGCAGCGTCGCCCGCAAGATCGTCGAAGAGCA

TCTCAATCTCTGCCTCGCCGCCGGCATCAACCACGAAGGCATCAACGCCGAAGTGGCGAA

GGGCCAGTGGGAATTCCAGATCTTCGGCAAGGGCTCCAAGACCGCCGCTGACCAGATGTG

GATGGCCCGCTACCTGATGCTGCGCCTCACCGAGAGCTACGGCATCGATATCGAATTCCA

TTGCAAGCCGCTCGGCGACACC

>Seq11 [organism=Bradyrhizobium sp. TUTVUMr82] [strain=TUTVUMr82] *glnII* gene for glutamine synthetase II, partial cds

ACGCCGACGCCGAGCCTGCGCGGCAAGACGCAGATCAAGGAATTCGCGTCGTTCCCGACC

CTCGAACAGCTTCCGCTGTGGGGCTTTGACGGTTCGTCCACCCAGCAGGCTGAAGGCCAC

AGCTCTGACTGCGTGCTGAAGCCGGTCGCCTGCTATCCCGACGGTGCGCGCGAGAACGGC

GTGCTGGTGATGTGCGAAGTCATGATGCCCGATGGCAAGACGCCGCATGTCTCGAACAAG

CGCGCGACCATTCTGGACGACGAAGGCGCCTGGTTCGGGTTCGAGCAGGAATACTTCTTC

TACAAGGACGGCCGTCCGCTCGGCTTCCCGGAACAGGGTTATCCGGCTCCGCAGGGCCCG

TACTACACCGGCGTCGGTTACAAGAACGTGGGCAGTGTTGCCCGCAAGATCGTCGAAGAA

CATCTCAACCTGTGCCTGCACGCCGGCATCAACCACGAAGGCATCAACGCCGAGGTGGCG

AAGGGCCACTGGGAATTCCAGATCTTCGGCAAGGGCTCCAAGACCGCCGCTGACCAGATG

TGGATGGCCCGCTACCTGATGCTGCGTCTCACCGAGACCTACGGCATCGACATCGAATTC

CACTGCAAGCCGCTCGGCGACACCGAC

>Seq12 [organism=Bradyrhizobium sp. TUTVUMr84] [strain=TUTVUMr84] *glnII* gene for glutamine synthetase II, partial cds

CTCAGATCAAGGAATTCGCGTCGTTTCCGACGCTCGAGCAGCTTCCGCTCTGGGGCTT

CGATGGCTCCTCCACCCAGCAGGCCGAAGGCCACAGCTCCGATTGCGTGCTGAAGCCGGT

CGCCGTGTTCCCGGACGGGGCACGCACCAACGGCGTGCTCGTGATGTGCGAAGTCATGAT

GCCCGACGGCAAGACCCCGCATCCGTCCAACAAGCGCGCCACCATCCTCGATGACGCCGG

CGCCTGGTTCGGCTTCGAGCAGGAATACTTCTTCTACAAGGACGGCCGCCCGCTCGGCTT

CCCGACCGCCGGCTATCCGGCGCCGCAGGGGCCCTACTACACCGGCGTCGGCTTCTCGAA

CGTCGGCGACGTCGCCCGCAAGATCGTCGAAGAGCATCTCGACCTCTGCCTCGCAGCCGG

CATCAACCATGAAGGCATCAACGCGGAAGTCGCCAAGGGCCAGTGGGAATTCCAAATCTT

CGGCAAGGGCTCCAAGACCGCTGCCGACCAGATGTGGATGGCCCGCTACCTGATGCTGCG

CCTCACCGAGAAGTACGGCATCGACATCGAATTCCACTGCAAGCCGCTCGGCGAC

>Seq13 [organism=Bradyrhizobium sp. TUTVUMr85] [strain=TUTVUMr85] *glnII* gene for glutamine synthetase II, partial cds

CGAACTTGCGCGGCAAAACCCAGATCAAGGAATTTTCGTCGTTTCCGACGCTCGAGCAGC

TTCCGCTGTGGGGCTTCGACGGCTCCTCGACCCAGCAGGCCGAAGGCCACAGCTCCGATT

GCGTGCTGAAGCCGGTCGCCGTGTTCCCGGACGGTGCCCGCACCAATGGCGTGCTGGTGA

TGTGCGAAGTTATGATGCCCGACGGCAAGACCCCGCATCCGTCCAACAAGCGCGCCACCA

TCCTCGACGACGCCGGCGCCTGGTTCGGCTTCGAGCAGGAATACTTCTTCTACAAGAACG

GTCGTCCGCTCGGCTTCCCCGAGTCCGGCTATCCCGCGCCGCAGGGCCCGTACTACACCG

GCGTCGGTTATTCGAACGTCGGCGACGTCGCCCGCAAGATCGTCGAAGAGCATCTCGACC

TCTGCCTCGCTGCCGGCATCAACCATGAAGGCATCAACGCGGAAGTCGCGAAGGGCCAGT

GGGAATTCCAGATCTTCGGCAAGGGTTCCAAGACCGCTGCCGACCAGATGTGGATGGCCCG

CTACCTGATGCTGCGTCTGACCGAGAAATACGGCATCGACATCGAATTCCACTGCAC

GCTCGGCGACACGGACTGGAACGGCT

>Seq14 [organism=Bradyrhizobium sp. TUTVUMr89] [strain=TUTVUMr89] *glnII* gene for glutamine synthetase II, partial cds

GCCGACTCCGAATTTGCGCGGCAAAACTCAGATCAAGGAATTCGCGTCGTTTCCGAC

GCTCGAGCAGCTTCCGCTCTGGGGCTTCGATGGCTCCTCCACCCAGCAGGCCGAAGGTC

ATAGCTCTGATTGCGTGCTGAAGCCGGTCGCCGTCTTCCCGGACGCCGCGCGCACCAATG

GCGTGCTCGTGATGTGCGAAGTCATGATGCCCGATGGCAAGACCCCGCATCCGTCAAACA

AGCGCGCCACCATCCTCGACGACGCCGGCGCATGGTTCGGCTTCGAGCAGGAATACTTCT

TCTACAAGGACGGCCGCCCGCTCGGCTTCCCGACTGCCGGCTATCCGGCCCCGCAGGGTC

CGTACTACACCGGCGTCGGCTACTCGAACGTCGGCGACGTCGCCCGCAAGATCGTCGAAG

AGCATCTCGACCTCTGCCTCGCTGCCGGCATCAACCATGAAGGCATCAACGCGGAAGTCG

CGAAGGGCCAGTGGGAATTCCAGATCTTCGGCAAGGGCTCCAAGACCGCTGCCGACCAGA

TGTGGATGGCCCGCTACCTGATGCTGCGCCTCACCGAGAAGTACGGCATCGACATCGAGT

TCCACTGCACGCTCGGCGAC

>Seq15 [organism=Bradyrhizobium sp. TUTVUMr93] [strain=TUTVUMr93] *glnII* gene for glutamine synthetase II, partial cds

ACTTGCGCGGCAAAACCCAGATCAAGGAATTTTCGTCGTTTCCGACGCTCGAGCAGCTTC

CGCTGTGGGGCTTCGACGGCTCCTCGACCCAGCAGGCCGAAGGCCACAGCTCCGATTGC

GTGCTGAAGCCGGTCGCCGTGTTCCCGGACGGTGCCCGCACCAATGGCGTGCTGGTGAT

GTGCGAAGTTATGATGCCCGACGGCAAGACCCCGCATCCGTCCAACAAGCGCGCCACCA

TCCTCGACGACGCCGGCGCCTGGTTCGGCTTCGAGCAGGAATACTTCTTCTACAAGAAC

GGTCGTCCGCTCGGCTTCCCCGAGTCCGGCTATCCGGCGCCGCAGGGCCCGTACTACAC

CGGCGTCGGTTATTCGAACGTCGGCGACGTCGCCCGCAAGATCGTCGAAGAGCATCTCG

ACCTCTGCCTCGCTGCCGGCATCAACCATGAAGGCATCAACGCGGAAGTCGCGAAGGGC

CAGTGGGAATTCCAGATCTTCGGCAAGGGTTCCAAGACCGCTGCCGACCAGATGTGGAT

GGCCCGCTACCTGATGCTGCGTCTGACCGAGAAATACGGCATCGACATCGAATTCCACT

GCACGCTCGGCGACACCGAC

>Seq16 [organism=Bradyrhizobium sp. TUTVUMr94] [strain=TUTVUMr94] *glnII* gene for glutamine synthetase II, partial cds

TTGCGCGGCAAAACTCAGATCAAGGAATTCGCGTCGTTCCCGACGCTCGAGCAGCTTCCGC

TCTGGGGCTTCGACGGCTCCTCGACCCAGCAGGCCGAAGGCCACAGCTCCGATTGCGTGC

TGAAGCCGGTCGCCGTGTTCCCGGACGGTGCGCGCACCAACGGCGTGCTGGTGATGTGCG

AAGTCATGATGCCCGATGGCAAGACCCCGCACGCCTCCAACAAGCGCGCCACCATCCTCG

ACGACGCCGGCGCCTGGTTCGGCTTCGAGCAGGAGTATTTCTTCTACAAGGACGGCCGTC

CGCTCGGCTTCCCGACATCGGGCTATCCCGCGCCGCAGGGCCCGTACTACACCGGCGTCG

GCTTCTCGAATGTGGGCGACGTCGCCCGCAAGATCGTCGAAGAGCATCTCGACCTCTGCT

TGGCCGCCGGCATCAACCATGAAGGCATCAACGCGGAAGTCGCCAAGGGCCAGTGGGAAT

TCCAGATCTTCGGCAAGGGCTCCAAGACCGCTGCCGACCAGATGTGGATGGCCCGCTACC

TGATGCTGCGCCTGACCGAGAAGTACGGCATCGACATCGAATTCCACTGCAAGCCGCTCG

GCGACACCGAC

>Seq17 [organism=Bradyrhizobium sp. TUTVUMr95] [strain=TUTVUMr95] *glnII* gene for glutamine synthetase II, partial cds

GCCGACTCCGAACTTGCGCGGCAAAACCCAGATCAAGGAATTTTCGTCGTTTCCGA

CGCTCGAGCAGCTTCCGCTGTGGGGCTTCGACGGCTCCTCGACCCAGCAGGCCGAAGG

CCACAGCTCCGATTGCGTGCTGAAGCCGGTCGCCGTGTTCCCGGACGGTGCCCGCACCAA

TGGCGTGCTGGTGATGTGCGAAGTTATGATGCCCGACGGCAAGACCCCGCATCCGTCCAA

CAAGCGCGCCACCATCCTCGACGACGCCGGCGCCTGGTTCGGCTTCGAGCAGGAATACTT

CTTCTACAAGAACGGTCGTCCGCTCGGCTTCCCCGAGTCCGGCTATCCGGCGCCGCAGGG

CCCGTACTACACCGGCGTCGGTTATTCGAACGTCGGCGACGTCGCCCGCAAGATCGTCGA

AGAGCATCTCGACCTCTGCCTCGCTGCCGGCATCAACCATGAAGGCATCAACGCGGAAGT

CGCGAAGGGCCAGTGGGAATTCCAGATCTTCGGCAAGGGTTCCAAGACCGCTGCCGACCA

GATGTGGATGGCCCGCTACCTGATGCTGCGTCTGACCGAGAAATACGGCATCGACATCGA

ATTCCACTGCACGCTCGGCG

>Seq18 [organism=Bradyrhizobium sp. TUTVUMr99] [strain=TUTVUMr99] *glnII* gene for glutamine synthetase II, partial cds

GACGCCGAGCCTGCGCGGCAAGACGCAGATCAAGGAATTCGCGTCGTTCCCGACCCTCGA

ACAGCTTCCGCTGTGGGGCTTTGACGGTTCGTCCACCCAGCAGGCTGAAGGCCACAGCTC

TGACTGCGTGCTGAAGCCGGTCGCCTGCTATCCCGACGGTGCGCGCGAGAACGGCGTGCT

GGTGATGTGCGAAGTCATGATGCCCGATGGCAAGACGCCGCATGTCTCGAACAAGCGCGC

GACCATTCTGGACGACGAAGGCGCCTGGTTCGGGTTCGAGCAGGAATACTTCTTCTACAA

GGACGGCCGTCCGCTCGGCTTCCCGGAACAGGGTTATCCGGCTCCGCAGGGCCCGTACTA

CACCGGCGTCGGTTACAAGAACGTGGGCAGTGTTGCCCGCAAGATCGTCGAAGAACATCT

CAACCTGTGCCTGCACGCCGGCATCAACCACGAAGGCATCAACGCCGAGGTGGCGAAGGG

CCAGTGGGAATTCCAGATCTTCGGCAAGGGCTCCAAGACCGCCGCTGACCAGATGTGGAT

CGCCCGCTACCTGATGCTGCGTCTCACCGAGACCTACGGCATCGACATCGAATTCCACTG

CAAGCCGCTCGGCGACACCG

>Seq19 [organism=Bradyrhizobium sp. TUTVUMr103] [strain=TUTVUMr103] *glnII* gene for glutamine synthetase II, partial cds

GCCGACTCCGAACTTGCGCGGCAAAACTCAGATCAAGGAATTCGCGTCGTTCCC

GACGCTCGAGCAGCTTCCGCTCTGGGGCTTCGACGGCTCCTCCACCCAGCAGGCCGA

AGGCCACAGCTCCGATTGCGTGCTGAAGCCGGTCGCGGTGTTTCCGGACGGTGCGCGCAC

CAACGGCGTGCTGGTGATGTGCGAAGTCATGATGCCCGATGGCAAGACCCCGCATCCGTC

CAACAAGCGCGCCACCATCCTGGACGATTCCGGCGCCTGGTTCGGCTTCGAGCAGGAATA

CTTCTTCTACAAGGACGGCCGTCCGCTCGGCTTCCCGACCGCCGGCTATCCCGCGCCGCA

AGGCCCGTACTACACCGGCGTCGGCTACTCGAACGTCGGCGACGTCGCCCGCAAGATCGT

CGAAGAGCATCTCGACCTGTGCTTGGCTGCCGGCATCAACCATGAAGGCATCAACGCGGA

AGTCGCCAAGGGCCAGTGGGAATTCCAGATCTTCGGCAAGGGCTCCAAGACCGCTGCCGA

CCAGATGTGGATGGCCCGCTACCTGATGCTGCGCCTCACCGAGAAGTACGGCATCGACAT

CGAATTCCACTGCAAGCCGCTCGGCGACA

>Seq20 [organism=Bradyrhizobium sp. TUTVUMm105] [strain=TUTVUMm105] *glnII* gene for glutamine synthetase II, partial cds

ACGCCGACTCCGAATTTGCGCGGCAAAACTCAGATCAAGGAATTCGCGTCGTTCC

CGACGCTCGAGCAGCTTCCGCTCTGGGGCTTCGACGGCTCCTCGACCCAGCA

GGCCGAAGGCCACAGCTCCGATTGCGTGCTGAAGCCGGTCGCCGTGTTCCCGGACGGTGC

GCGCACCAACGGCGTGCTGGTGATGTGCGAAGTCATGATGCCCGATGGCAAGACCCCGCA

CGCCTCCAACAAGCGCGCCACGATCCTCGACGACGCCGGCGCCTGGTTCGGCTTCGAGCA

GGAGTATTTCTTCTACAAGGACGGCCGTCCGCTCGGCTTCCCGACATCGGGCTATCCCGC

GCCGCAGGGCCCGTACTACACCGGCGTCGGCTTCTCGAATGTCGGCGACGTCGCCCGCAA

GATCGTCGAAGAGCATCTCGACCTCTGCTTGGCCGCCGGTATCAACCATGAAGGCATCAA

CGCGGAAGTCGCCAAGGGCCAGTGGGAATTCCAGATCTTCGGCAAGGGCTCCAAGACCGC

TGCCGACCAGATGTGGATGGCCCGCTACCTGATGCTGCGCCTGACCGAGAAGTACGGCAT

CGACATCGAATTCCACTGCAAGCCGCTCGGCGAC

>Seq21 [organism=Bradyrhizobium sp. TUTVUMm108] [strain=TUTVUMm108] *glnII* gene for glutamine synthetase II, partial cds

CGCCGACTCCGAACTTGCGCGGCAAAACCCAGATCAAGGAATTTGCGTCGTTCCCGACGC

TCGAGCAGCTTCCGCTCTGGGGCTTCGATGGCTCCTCCACCCAGCAGGCCGAAGGCCACA

GCTCCGATTGCGTGCTGAAGCCGGTCGCCGTCTTCCCGGACGGCGCGCGCACCAACGGC

GTGCTGGTGATGTGCGAAGTCATGATGCCCGACGGCAAGACCCCGCATGCGTCCAACAA

GCGCGCCACCATCCTCGATGACGCCGGCGCCTGGTTCGGCTTCGAGCAGGAGTACTTCTT

CTACAAGGACGGCCGTCCGCTCGGCTTCCCGACCGCCGGCTATCCGGCGCCGCAGGGCCC

GTACTACACCGGCGTCGGCTTCTCGAACGTCGGCGACGTCGCCCGCAAGATCGTCGAGGA

GCATCTCGACCTCTGCCTGGCTGCCGGCATCAACCATGAAGGCATCAACGCGGAAGTCGC

GAAGGGCCAGTGGGAATTCCAGATCTTCGGCAAGGGCTCCAAGACCGCTGCCGACCAGAT

GTGGATGGCCCGCTACCTGATGCTGCGTCTCACGGAGAAGTACGGCATCGACATCGAGTT

CCACTGCAAGCCGCTCGGCGACACCGA

>Seq22 [organism=Bradyrhizobium sp. TUTVUMm110] [strain=TUTVUMm110] *glnII* gene for glutamine synthetase II, partial cds

ACGCCTACTCCGAACTTGCGCGGCAAAACCCAGATCAAGGAATTTGCGTCGTTCCCGA

CGCTCGAGCAGCTTCCGCTCTGGGGCTTCGATGGCTCCTCCACCCAGCAGGCCGAAGGCC

ACAGCTCCGATTGCGTGCTGAAGCCGGTCGCCGTCTTCCCGGACGGCGCGCGCACCAACG

GCGTGCTGGTGATGTGCGAAGTCATGATGCCCGACGGCAAGACCCCGCATGCGTCCAACA

AGCGCGCCACCATCCTCGATGACGCCGGCGCCTGGTTCGGCTTCGAGCAGGAGTACTTCT

TCTACAAGGACGGCCGTCCGCTCGGCTTCCCGACCGCCGGCTATCCGGCGCCGCAGGGCC

CGTACTACACCGGCGTCGGCTTCTCGAACGTCGGCGACGTCGCCCGCAAGATCGTCGAGG

AGCATCTCGACCTCTGCCTGGCTGCCGGCATCAACCATGAAGGCATCAACGCGGAAGTCG

CGAAGGGCCAGTGGGAATTCCAGATCTTCGGCAAGGGCTCCAAGACCGCTGCCGACCAGA

TGTGGATGGCCCGCTACCTGATGCTGCGTCTCACGGAGAAGTACGGCATCGACATCGAGT

TCCACTGCAAGCCGCTCGGCGAC

>Seq23 [organism=Bradyrhizobium sp. TUTVUMm112] [strain=TUTVUMm112] *glnII* gene for glutamine synthetase II, partial cds

TTGCGCGGCAAACTCAGATCAAGGAATTCGCGTCGTTCCCGACGCTCGAGCAGCTTCCGC

TCTGGGGCTTCGACGGCTCCTCCACCCAGCAGGCCGAAGGTCACAGCTCCGATTGCGTGC

TGAAGCCGGTCGCGGTGTTTCCGGACGGTGCGCGCACCAACGGCGTGCTGGTGATGTGCG

AAGTCATGATGCCCGATGGCAAGACCCCGCATCCGTCCAACAAGCGCGCCACCATCCTGG

ACGATTCCGGCGCCTGGTTCGGCTTCGAGCAGGAATACTTCTTCTACAAGGACGGCCGTC

CGCTCGGCTTCCCGACCGCCGGCTATCCCGCGCCGCAAGGCCCGTACTACACCGGCGTCG

GCTACTCGAACGTCGGCGACGTCGCCCGCAAGATCGTCGAAGAGCATCTCGACCTGTGCT

TGGCTGCCGGCATCAACCATGAAGGCATCAACGCGGAAGTCGCCAAGGGCCAGTGGGAAT

TCCAGATCTTCGGCAAGGGCTCCAAGACCGCTGCCGACCAGATGTGGATGGCCCGCTACC

TGATGCTGCGCCTCACCGAGAAGTACGGCATCGACATCGAATTCCACTGCAAGCCGCTCG

GCGAC

>Seq24 [organism=Bradyrhizobium sp. TUTVUMm113] [strain=TUTVUMm113] *glnII* gene for glutamine synthetase II, partial cds

TTGCGCGGCAAACTCAGATCAAGGAATTCGCGTCGTTCCCGACGCTCGAGCAGCTTCCG

CTCTGGGGCTTCGACGGCTCCTCCACCCAGCAGGCCGAAGGCCACAGCTCCGATTGCGTG

CTGAAGCCGGTCGCGGTGTTTCCGGACGGTGCGCGCACCAACGGCGTGCTGGTGATGTGC

GAAGTCATGATGCCCGATGGCAAGACCCCGCATCCGTCCAACAAGCGCGCCACCATCCTG

GACGATTCCGGCGCCTGGTTCGGCTTCGAGCAGGAATACTTCTTCTACAAGGACGGCCGT

CCGCTCGGCTTCCCGACCGCCGGCTATCCCGCGCCGCAAGGCCCGTACTACACCGGCGTC

GGCTACTCGAACGTCGGCGACGTCGCCCGCAAGATCGTCGAAGAGCATCTCGACCTGTGC

TTGGCTGCCGGCATCAACCATGAAGGCATCAACGCGGAAGTCGCCAAGGGCCAGTGGGAA

TTCCAGATCTTCGGCAAGGGCTCCAAGACCGCTGCCGACCAGATGTGGATGGCCCGCTAC

CTGATGCTGCGCCTCACCGAGAAGTACGGCATCGACATCGAATTCCACTGCAAGCCGCTG

GGCGACACCGACTGGAACG

>Seq25 [organism=Bradyrhizobium sp. TUTVUMm114] [strain=TUTVUMm114] *glnII* gene for glutamine synthetase II, partial cds

TTGCGCGGCAAACTCAGATCAAGGAATTCGCGTCGTTCCCGACGCTCGAGCAGCTTCCG

CTCTGGGGCTTCGACGGCTCCTCCACCCAGCAGGCCGAAGGCCACAGCTCCGATTGCGTG

CTGAAGCCGGTCGCGGTGTTTCCGGACGGTGCGCGCACCAACGGCGTGCTGGTGATGTGC

GAAGTCATGATGCCCGATGGCAAGACCCCGCATCCGTCCAGCAAGCGCGCCACCATCCTG

GACGATTCCGGCGCCTGGTTCGGCTTCGAGCAGGAATACTTCTTCTACAAGGACGGCCGT

CCGCTCGGCTTCCCGACCGCCGGCTATCCCGCGCCGCAAGGCCCGTACTACACCGGCGTC

GGCTACTCGAACGTCGGCGACGTCGCCCGCAAGATCGTCGAAGAGCATCTCGACCTGTGC

TTGGCTGCCGGCATCAACCATGAAGGCATCAACGCGGAAGTCGCCAAGGGCCAGTGGGAA

TTCCAGATCTTCGGCAAGGGCTCCAAGACCGCTGCCGACCAGATGTGGATGGCCCGCTAC

CTGATGCTGCGCCTCACCGAGAAGTACGGCATCGACATCGAATTCCACTGCAAGCCGCTC

GGCGACACCGAC

>Seq26 [organism=Bradyrhizobium sp. TUTVUMm115] [strain=TUTVUMm115] *glnII* gene for glutamine synthetase II, partial cds

GACTCCGAATTTGCGCGGCAAAACTCAGATCAAGGAATTCGCCTCGTTTCCGAC

GCTCGAGCAGCTTCCGCTCTGGGGCTTCGACGGCTCCTCTACTCAGCAGGCCGAAGGC

CACAGCTCCGATTGCGTGCTGAAGCCGGTCGCGGTGTTTCCGGACGGTGCGCGCACCAAC

GGCGTGCTGGTGATGTGCGAAGTCATGATGCCCGATGGCAAGACCCCGCATCCGTCCAAC

AAGCGCGCCACCATCCTGGACGATTCCGGCGCCTGGTTCGGCTTCGAGCAGGAATACTTC

TTCTACAAGGACGGCCGTCCGCTCGGCTTCCCGACCGCCGGCTATCCCGCGCCGCAAGGC

CCGTACTACACCGGCGTCGGCTACTCGAATGTCGGCGACGTCGCCCGCAAGATCGTCGAA

GAGCATCTCGACCTGTGCTTGGCTGCCGGCATCAACCATGAAGGCATCAACGCGGAAGTCGC

CAAGGGTCAGTGGGAATTCCAGATCTTCGGCAAGGGCTCCAAGACCGCTGCCGACCAGAT

GTGGATGGCCCGCTACCTGATGCTGCGCCTCACCGAGAAGTACGGCATCGACATCGAATT

CCACTGCAAGCCGCTCGGCGAC

>Seq27 [organism=Bradyrhizobium sp. TUTVUMm116] [strain=TUTVUMm116] *glnII* gene for glutamine synthetase II, partial cds

GACTCCGAACTTGCGCGGCAAAACTCAGATCAAGGAATTCGCGTCGTTCCCGA

CGCTCGAGCAGCTTCCGCTCTGGGGCTTCGACGGCTCCTCCACTCAGCAGGC

CGAAGGCCACAGCTCCGATTGCGTGCTGAAGCCGGTCGCGGTGTTTCCGGACGGTGCGCG

CACCAACGGCGTGCTGGTGATGTGCGAAGTCATGATGCCCGATGGCAAGACCCCGCATCC

GTCCAACAAGCGCGCCACCATCCTGGACGATTCCGGCGCCTGGTTCGGCTTCGAGCAGGA

ATACTTCTTCTACAAGGACGGCCGTCCGCTCGGCTTCCCGACCGCCGGCTATCCCGCGCC

GCAAGGCCCGTACTACACCGGCGTCGGCTACTCGAACGTCGGCGACGTCGCCCGCAAGAT

CGTCGAAGAGCATCTCGACCTGTGCTTGGCTGCCGGCATCAACCATGAAGGCATCAACGC

GGAAGTCGCCAAGGGCCAGTGGGAATTCCAGATCTTCGGCAAGGGCTCCAAGACCGCTGC

CGACCAGATGTGGATGGCCCGCTACCTGATGCTGCGCCTCACCGAGAAGTACGGCATCGA

CATCGAATTCCACTGCAAGCCGCTCGGCGAC

>Seq28 [organism=Bradyrhizobium sp. TUTVUMm117] [strain=TUTVUMm117] *glnII* gene for glutamine synthetase II, partial cds

CCGAGCCTGCGCGGTAAGACGCAGATCAAGGAATTCGCGTCGTTTCCGACTCTCGAA

CAGCTTCCGCTGTGGGGCTTTGACGGTTCGTCCACTCAGCAGGCTGAAGGCCACAGCTCT

GACTGCGTGCTGAAGCCGGTCGCCTGCTATCCCGACGCCGCGCGCGAGAACGGCGTGCTG

GTGATGTGCGAAGTCATGATGCCCGACGGCAAGACGCCGCATGTCTCGAACAAGCGCGCC

ACCGTTCTGGATGACGAAGGCGCCTGGTTCGGCTTCGAGCAGGAATACTTCTTCTACAAG

GACGGCCGTCCGCTCGGCTTTCCGGAAGAGGGTTATCCGGCGCCGCAGGGCCCGTACTAC

ACCGGCGTCGGCTACAAGAACGTCGGCAGCGTCGCCCGCAAGATCGTCGAAGAGCATCTC

AATCTCTGCCTCGCCGCCGGCATCAACCACGAAGGCATCAACGCCGAAGTGGCGAAGGGC

CAGTGGGAATTCCAGATCTTCGGCAAGGGCTCCAAGACCGCCGCTGACCAGATGTGGATG

GCCCGCTACCTGATGCTGCGCCTCACCGAGAGCTACGGCATCGATATCGAATTCCATTGC

AAGCCGCTCGGCGAC

>Seq29 [organism=Bradyrhizobium sp. TUTVUI122] [strain=TUTVUMI122] *glnII* gene for glutamine synthetase II, partial cds

GCCGACTCCGAACTTGCGCGGCAAAACCCAGATCAAGGAATTTGCGTCGTTCCCGACG

CTCGAGCAGCTTCCGCTCTGGGGCTTCGATGGCTCCTCCACCCAGCAGGCCGAAGGCCAC

AGCTCCGATTGCGTGCTGAAGCCGGTCGCCGTCTTCCCGGACGGCGCGCGCACCAACGGC

GTGCTGGTGATGTGCGAAGTCATGATGCCCGACGGCAAGACCCCGCATGCGTCCAACAAG

CGCGCCACCATCCTCGATGACGCCGGCGCCTGGTTCGGCTTCGAGCAGGAGTACTTCTTC

TACAAGGACGGCCGTCCGCTCGGCTTCCCGACCGCCGGCTATCCGGCGCCGCAGGGCCCG

TACTACACCGGCGTCGGCTTCTCGAACGTCGGCGACGTCGCCCGCAAGATCGTCGAGGAG

CATCTCGACCTCTGCCTGGCTGCCGGCATCAACCATGAAGGCATCAACGCGGAAGTCGCG

AAGGGCCAGTGGGAATTCCAGATCTTCGGCAAGGGCTCCAAGACCGCTGCCGACCAGATG

TGGATGGCCCGCTACCTGATGCTGCGTCTCACGGAGAAGTACGGCATCGACATCGAGTTC

CACTGCAAGCCGCTCGGCGAC

>Seq30 [organism=Bradyrhizobium sp. TUTVUMr80] [strain=TUTVUMr80] glnII gene for glutamine synthetase II, partial cds

CCCAATTTGCGCGGCAAAACCCAGATCAAGGAATTTTCGTCGTTTCCGACGCTCGAGCAG

CTTCCGCTGTGGGGCTTCGACGGCTCCTCGACCCAGCAGGCCGAAGGCCACAGCTCCGAT

TGCGTGCTGAAGCCGGTCGCCGTGTTCCCGGACGGTGCTCGCACCAATGGCGTGCTGGTG

ATGTGCGAAGTTATGATGCCCGATGGCAAGACCCCGCATCCGTCCAACAAGCGCGCCACC

ATTCTCGACGACGCCGGCGCCTGGTTCGGCTTCGAGCAGGAATACTTCTTCTACAAGAACG

GCCGTCCGCTCGGCTTCCCCGAGTCCGGCTATCCGGCGCCGCAGGGCCCGTATTACACCG

GCGTCGGTTATTCGAACGTCGGCGACGTCGCCCGCAAGATCGTCGAAGAGCATCTCGACC

TCTGCCTCGCTGCCGGCATCAACCATGAAGGCATCAACGCGGAAGTCGCGAAGGGCCAGT

GGGAATTCCAGATCTTCGGCAAGGGTTCCAAGACCGCTGCCGACCAGATGTGGATGGCCC

GCTACCTGATGCTGCGTCTGACCGAGAAATACGGCATCGACATCGAATTCCACTGCACGC

TCGGCGACACCG

***gyrB***

>Seq1 [organism=Bradyrhizobium sp. TUTVUMp5] [strain=TUTVUMp5] *gyrB* gene for DNA gyrase subunit B, partial cds,

GCGTCGGCGTCTCCGTCGTCAACGCGCTGTCCAGCAAGCTCGGCTTGCGGATCTGGCG

CGACAACAAGGAGCACTACATCGAATTCGCCCATGGCGATGCCGTGGCGCCGCTG

AAGATCGTCGCCGATGCGCCGGGCAAGCGCGGCACCGAGGTGACGTTCCTCGCCTCGACC

GAGACGTTCAAGAACGTCGAATATGATTTCGCCACGCTCGAGCATCGCCTGCGCGAGCTC

GCCTTCCTCAATTCCGGCGTCAACATCATCCTCTCCGACACGCGCCACGCGGTCGAGAAG

CGCGAGGAGATGCACTATTCCGGCGGCGTCGAGGAGTTCGTCAAATATCTCGACCGCAAC

AAGAAGGCGATCGTGCCGGCGCCGATCATGGTGCGTTCCGAAGCCAACGGCATCGGCGTC

GAGGCCGCACTGTGGTGGAACGACAGCTACCACGAGAACGTGCTGTGCTTCACCAACAAC

ATCCCGCAGCGTGACGGCGGCACCCATCTGGCCGGTTTCCGCGGCGCGCTGACGCGCCAG

GTCAACGGCTATGCCGAGGCCAATGCGAAAAAGGAAAAGATCGCGCTGACCGGCGACGAC

TGCCGCGAAGGTCTCACCGCCGTGCTGTCGGTGAAGGTGCCAGATCCAAAGTTCTCGT

>Seq2 [organism=Bradyrhizobium sp. TUTVUMp6] [strain=TUTVUMp6] *gyrB* gene for DNA gyrase subunit B, partial cds,

CGGCGTCGGCGTCTCCGTCGTCAACGCGCTGTCGAGCAAGCTCGGCTTGCGCATCTGGC

GCGACAACAAGGAGCACTATATCGAGTTCGCCCACGGCGATGCCGTCGCACCGCTCAAGG

TCGTCGGCGATGCCCCGGGCAAGCGCGGCACCGAGGTGACGTTCCTGGCCTCGACCGAGA

CCTTCAAGAACATCGAATACGACTTCGCCACGCTCGAGCATCGCTTACGCGAGCTCGCCT

TCCTCAATTCCGGCGTCAACATCGTGCTCTCCGACATGCGCCACGCGGTCGAGAAGCGCG

AGGAGATGTTCTATTCCGGCGGCGTCGAGGAGTTCGTCAAATATCTCGACCGCAACAAGA

AGGCCCTGGTGCCGGCGCCGATCATGGTGCGCTCGGAAGCCAACGGCATCGGCGTCGAGG

CCGCTTTGTGGTGGAACGACAGCTACCACGAGAACGTGCTGTGCTTCACCAACAACATCC

CGCAACGCGACGGGGGCACCCATCTCGCCGGCTTCCGCGGCGCGCTGACGCGCCAGGTCA

ACGGCTATGCCGAGGCCAATGCCAAGAAGGAAAAGATCGCGCTGACCGGCGACGATTGCC

GCGAAGGGCTCACCGCCGTTCTCTCGGTGAAGGTGCCCGATCCGAAGTTTTCGTCGCAGAC

CAGGGAACAAGCTA

>Seq3 [organism=Bradyrhizobium sp. TUTVUMp8] [strain=TUTVUMp8] *gyrB* gene for DNA gyrase subunit B, partial cds,

GCGTCGGCGTCTCCGTCGTCAACGCGCTGTCCAGCAAGCTCGGCTTGCGGATCTGGCGCG

ACAACAAGGAGCACTACATCGAATTCGCCCATGGCGATGCCGTGGCGCCGCTCAAGATCG

TCGGCGATGCGCCGGGCAAGCGCGGCACCGAGGTGACGTTCCTGGCCTCGACCCAGACGT

TCAAGAACGTCGAATATGATTTCGCCACGCTCGAGCATCGCCTGCGCGAGCTCGCCTTCC

TCAATTCCGGCGTCAACATCATCCTCTCCGACACGCGCCACGCGGTCGAGAAGCGCGAGG

AGATGCACTATTCCGGCGGTGTCGAGGAGTTCGTCAAATATCTCGACCGCAACAAGAAGG

CGATCGTGCCGGCGCCGATCATGGTGCGCTCGGAAGCCAACGGCATCGGCGTCGAGGCCG

CTCTGTGGTGGAACGACAGCTACCACGAGAACGTGTTGTGCTTCACCAACAACATCCCGC

AGCGTGATGGCGGCACCCATCTGGCCGGTTTCCGCGGCGCGCTGACGCGCCAGGTCAACG

GCTATGCCGAGGCCAATGCGAAAAAGGAAAAGATCGCGCTGACCGGCGACGACTGCCGCG

AAGGCCTCACCGCCGTGCTGTCGGTGAAGGTGCCCGATCCGAAGTTCTCGT

>Seq4 [organism=Bradyrhizobium sp. TUTVUMp48] [strain=TUTVUMp48] *gyrB* gene for DNA gyrase subunit B, partial cds,

TCGGCGTCTCCGTCGTCAACGCGCTGTCAAGTAAGCTCGGCTTGCGGATCTGGCGCGAT

GACAAGGAGCACTACATCGAGTTCGCCCATGGCGATGCGGTGGCGCCGCTGAAGGTAGTC

GGCGATGCGCCGGGCCGGCGCGGCACCGAGGTGACGTTCCTGGCCTCGACCGAGACGTT

CAAGAACGTCGAATATGATTTCGCCACGCTCGAGCACCGTCTGCGCGAGCTCGCCTTCC

TCAATTCCGGCGTCAACATCATCCTCTCCGACATGCGCCACGCGGTCGAGAAGCGCGAG

GAGATGCACTATTCCGGCGGCGTCGAGGAGTTCGTCAAATATCTCGACCGCAACAAGAA

GGCGCTGGTGCCCGCGCCAATCATGGTGCGCTCGGAAGCCAACGGCATCGGCGTCGAGG

CTGCTTTGTGGTGGAACGACAGCTACCACGAGAACGTGCTCTGCTTCACCAACAACATC

CCACAGCGTGACGGCGGTACCCATCTGGCCGGCTTCCGCGGCGCGCTGACGCGCCAGGT

CAACGGTTATGCCGAGGCCAATGCGAAAAAGGAAAAGATCGCGCTGACCGGCGACGACT

GCCGCGAAGGCCTCACCGCCGTGCTGTCGGTGAAGGTGCCGGACCCGAAGTTTTCGTCG

CAGACCA

>Seq5 [organism=Bradyrhizobium sp. TUTVUMp50] [strain=TUTVUMp50] *gyrB* gene for DNA gyrase subunit B, partial cds,

CGGCGTCGGCGTCTCCGTCGTCAACGCGCTGTCCAGCAAGCTCGGCTTGCGGATCTGGCG

CGACAACAAGGAGCACTACATCGAGTTCGCCCATGGCGATGCCGTGGCGCCGCTCAAGA

TCGTCGGCGATGCGCCGGGCAAGCGCGGCACCGAGGTGACGTTCCTGGCCTCGACCCAG

ACGTTCAAGAACGTCGAATATGATTTCGCCACGCTCGAGCATCGCCTGCGCGAGCTCGC

CTTCCTCAATTCCGGCGTCAACATCATCCTCTCCGACACGCGCCACGCGGTCGAGAAGC

GCGAGGAGATGCACTATTCCGGCGGCGTCGAGGAGTTCGTCAAATATCTCGACCGCAAC

AAGAAGGCGATCGTGCCGGCGCCGATCATGGTGCGCTCGGAAGCCAACGGCATCGGCGT

CGAGGCCGCTTTGTGGTGGAACGACAGCTACCACGAGAACGTGCTGTGCTTCACCAACA

ACATCCCGCAGCGTGACGGCGGCACCCATCTGGCCGGTTTCCGCGGCGCGCTGACGCGC

CAGGTCAACGGTTATGCCGAGGCCAATGCGAAAAAGGAAAAGATCGCGCTGACCGGCGA

CGACTGCCGCGAAGGCCTCACCGCCGTGCTGTCGGTGAAGGTGCCGGACCCGAAGTTTT

CGT

>Seq6 [organism=Bradyrhizobium sp. TUTVUMp53] [strain=TUTVUMp53] *gyrB* gene for DNA gyrase subunit B, partial cds,

CTGGGCTTGCGGATCTGGCGCGACAACAAGGAGCACTACATCGAGTTCGCCCATGGCG

ATGCCGTGGCGCCGCTGAAGATCGTCGGCGATGCGCCGGGCAAGCGCGGCACCGAGGTGAC

GTTCCTCGCCTCGACCGAGACGTTCAAGAACGTCGAATATGATTTCGCCACGCTCGAGC

ATCGCCTGCGCGAGCTCGCCTTCCTCAATTCCGGCGTCAACATCATCCTCTCCGACACG

CGCCATGCGGTCGAGAAGCGCGAGGAGATGCACTATTCCGGCGGCGTCGAGGAGTTCGT

CAAATATCTCGACCGGAACAAGAAGGCGATCGTGCCGGCGCCGATCATGGTGCGCTCGG

AAGCCAACGGCATCGGCGTCGAGGCCGCTTTGTGGTGGAACGACAGCTACCACGAGAAC

GTGCTGTGCTTCACGAACAACATCCCGCAACGTGACGGCGGCACCCATCTGGCCGGTTT

CCGCGGCGCGCTGACGCGCCAGGTCAACGGCTATGCCGAGGCCAATGCGAAAAAGGAAA

AGATCGCGCTGACCGGCGACGACTGCCGCGAAGGCCTCACCGCCGTGCTGTCGGTGAA

>Seq7 [organism=Bradyrhizobium sp. TUTVUMp56] [strain=TUTVUMp56] *gyrB* gene for DNA gyrase subunit B, partial cds,

GCGTCTCCGTCGTCAACGCGCTGTCCAGCAAGCTCGGCTTGCGGATCTGGCGCGACAACA

AGGAGCACTACATCGAGTTCGCCCATGGCGATGCCGTGGCGCCGCTGAAGATCGTCGGCG

ATGCGCCGGGCAAGCGCGGCACCGAGGTGACGTTCCTCGCCTCGACCCAGACGTTCAAGA

ACGTCGAATATGATTTCGCCACGCTCGAGCATCGCCTGCGCGAGCTCGCCTTCCTCAATT

CCGGCGTCAACATCATCCTCTCCGACACGCGCCACGCGGTCGAGAAGCGCGAGGAGATGC

ACTATTCCGGCGGAGTCGAGGAGTTCGTCAAATATCTCGACCGCAACAAGAAGGCGATCG

TGCCGGCGCCGATCATGGTGCGTTCCGAAGCCAACGGCATCGGCGTCGAGGCCGCACTGT

GGTGGAACGACAGCTACCACGAGAACGTGCTGTGCTTCACCAACAACATCCCGCAGCGTG

ACGGCGGCACCCATCTGGCCGGTTTCCGCGGCGCGCTGACGCGCCAGGTCAACGGCTATG

CCGAGGCCAATGCGAAAAAGGAAAAAATCGCACTGACCGGCGACGACTGCCGCGAAGGTC

TCACCGCCGTGCTGCGGGTGAAGGTGCCAGATCCAAAGTTCTCGT

>Seq8 [organism=Bradyrhizobium sp. TUTVUMp60] [strain=TUTVUMp60] *gyrB* gene for DNA gyrase subunit B, partial cds,

GCACGGCGTCGGCGTCTCCGTCGTCAACGCGCTCTCCAGCAAGCTCGCGCTGCGGATCTG

GCGCGACGACAAGGAGCACTACATCGAATTCGCGCACGGCGATGCCGTGGCGCCGCTCAA

AGTGGTCGGCGATGCCCCAGGCAAGCGCGGCACCGAGGTCACCTTCCTCGCCTCGGTCGA

GACCTTCAAGAACATCGAGTATGATTTCGCGACGCTCGAGCACCGGCTGCGCGAGCTCGC

CTTCCTCAATTCCGGCGTCAACATCGTGCTCTCCGACATGCGTCACGCGGTCGAGAAGCG

CGAGGAGATGCACTATTCCGGCGGCGTCGAGGAATTCGTCAAATATCTCGACCGCAACAA

GAAGGCGATCGTGCCGGCACCGATCATGGTGCGTTCGGAAGCCAACGGAATTGGCGTCGA

GGCGGCGCTGTGGTGGAACGACAGCTACCACGAGAACGTGCTGTGCTTCACCAACAACAT

CCCGCAGCGCGACGGCGGCACCCATCTGGCCGGCTTCCGCGGCGCGCTGACGCGCCAGGT

CAACGGCTATGCCGAGGCCAATGCGAAGAAGGAAAAGATCGCGCTGACCGGCGACGATTG

CCGCGAAGGCCTCACCGCGGTGCTGTCGGTGAAGGTGCCCGATCCGAAATTCTCGT

>Seq9 [organism=Bradyrhizobium sp. TUTVUMp65] [strain=TUTVUMp65] *gyrB* gene for DNA gyrase subunit B, partial cds,

GCGTCGGCGTCTCCGTCGTCAACGCGCTGTCCAGCAAGCTCGGCTTGCGGATCTGGCGC

GACAACAAGGAGCACTACATCGAGTTCGCCCATGGCGATGCCGTGGCGCCGCTCAAGATC

GTCGGCGATGCGCCGGGCAAGCGCGGCACCGAGGTGACGTTCCTGGCCTCGACCCAGACG

TTCAAGAACGTCGAATATGATTTCGCCACGCTCGAGCATCGCCTGCGCGAGCTCGCCTTC

CTCAATTCCGGCGTCAACATCATCCTCTCCGACACGCGCCACGCGGTCGAGAAGCGCGAG

GAGATGCACTATTCCGGCGGCGTCGAGGAGTTCGTCAAATATCTCGACCGCAACAAGAAG

GCGATCGTGCCGGCGCCGATCATGGTGCGCTCGGAAGCCAACGGCATCGGCGTCGAGGCC

GCTTTGTGGTGGAACGACAGCTACCACGAGAACGTGCTGTGCTTCACCAACAACATCCCG

CAGCGTGACGGCGGCACCCATCTGGCCGGTTTCCGCGGCGCGCTGACGCGCCAGGTCAAC

GGTTATGCCGAGGCCAATGCGAAAAAGGAAAAGATCGCGCTGACCGGCGACGACTGCCGC

GAAGGCCTCACCGCCGTGCTGTCGGTGAAGGTGCCGGACCCAAAGTT

>Seq10 [organism=Bradyrhizobium sp. TUTVUMp75] [strain=TUTVUMp75] *gyrB* gene for DNA gyrase subunit B, partial cds,

GCGTCGGCGTCTCCGTCGTCAACGCGCTCTCCAGCAAGCTCGCGCTGCGGATCTGGCGCG

ACGACAAGGAGCACTACATCGAATTCGCGCACGGCGATGCCGTGGCGCCGCTCAAAGTGG

TCGGCGATGCCCCAGGCAAGCGCGGCACCGAGGTCACCTTCCTCGCCTCGGTCGAGACCT

TCAAGAACATCGAGTATGATTTCGCGACGCTCGAGCACCGGCTGCGCGAGCTCGCCTTCC

TCAATTCCGGCGTCAACATCGTGCTCTCCGACATGCGTCACGCGGTCGAGAAGCGCGAGG

AGATGCACTATTCCGGCGGCGTCGAGGAATTCGTCAAATATCTCGACCGCAACAAGAAGG

CGATCGTGCCGGCACCGATCATGGTGCGTTCGGAAGCCAACGGCATCGGCGTCGAGGCGG

CGCTGTGGTGGAACGACAGCTACCACGAGAACGTGCTGTGCTTCACCAACAACATCCCGC

AGCGCGACGGCGGCACCCATCTGGCCGGCTTCCGCGGCGCGCTGACGCGCCAGGTCAACG

GCTATGCCGAGGCCAATGCGAAGAAGGAAAAGATCGCGCTGACCGGCGACGATTGCCGCG

AAGGCCTCACCGCGGTGCTGTCGGTGAAGGTGCCCGATCCGAAATTCTCGT

>Seq11 [organism=Bradyrhizobium sp. TUTVUMr80] [strain=TUTVUMr80] *gyrB* gene for DNA gyrase subunit B, partial cds,

GCGTCGGCGTCTCCGTCGTCAACGCACTGTCGAGCAAGCTCGGCCTGCGCATCTGGCG

CGATGACAAGGAACATTACATCGAGTTCGCCCATGGCGATGCGGTGGCACCGCTCA

AGGTCGTCGGCGATGCGCCGGGCAAGCGCGGCACCGAGGTGACGTTCCTGGCCTCGAGCG

AGACTTTCAAGAACATCGAATATGATTTTGCGACGCTCGAGCATCGCCTGCGCGAGCTCG

CCTTCCTCAATTCCGGCGTCAACATCGCGCTGTCAGACATGCGTCACGCGGTCGAGAAGC

GCGAGGAGATGTATTATTCCGGCGGCGTTGAGGAATTCGTCAAATATCTCGACCGCAACA

AGAAGGCCCTGGTGCCCTCGCCGATCATGGTGCGTGCGGAAGCCAACGGCATCGGCGTCG

AGGCCGCCTTGTGGTGGAACGACAGCTACCATGAGAACGTGCTGTGCTTCACCAACAACA

TCCCGCAGCGTGACGGCGGCACCCATTTGGCCGGCTTCCGCGGCGCGCTGACGCGCCAGG

TCAACGGTTATGCCGAGGCCAATGCCAAGAAGGAAAAGATCGCGCTCACCGGCGACGATT

GCCGCGAAGGCCTGACCGCGGTGCTGTCGGTGAAGGTGCCGGATCCAAAGTTTTCGTCGCA

>Seq12 [organism=Bradyrhizobium sp.TUTVUMr82] [strain=TUTVUMr82] *gyrB* gene for DNA gyrase subunit B, partial cds,

GCGTCGGCGTCTCCGTCGTCAACGCGCTGTCCAGCAAGCTCGCGCTGCGGATCTGGCG

CGACGACAAGGAGCACTACATCGAGTTCGCGCACGGTGACGCCGTGGCGCCGCTCAAGG

TGGTCGGCGATGCGCCGGGCAAGCGCGGCACCGAGGTCACCTTCCTGGCCTCGACCGAG

ACCTTCAAGAACATCGAGTATGATTTCGCGACGCTGGAGCACCGGCTGCGCGAGCTCGC

CTTCCTCAATTCCGGTGTCCACATCGTGCTGTCAGACATGCGCCACGCGGTCGAGAAGC

GCGAGGAGATGCATTATTCCGGCGGCGTCGAGGAATTCGTCAAATATCTCGACCGCAAC

AAGAAGGCGATCGTACCGACGCCGATCATGGTGCGTTCGGAGGCGAACGGCATCGGCGT

CGAGGCCGCGCTGTGGTGGAACGACAGCTACCACGAGAACGTGCTGTGCTTCACCAACA

ACATCCCGCAGCGTGACGGCGGCACCCATCTGGCCGGCTTCCGCGGCGCGCTGACGCGC

CAGGTCAACGGCTATGCCGAGGCCAACGCGAAGAAGGAAAAGATCGCGCTGACCGGCGA

CGATTGCCGTGAAGGTCTCACCGCGGTGCTGTCGGTGAAGGTGCCCGATCCAAAGTTCT

CGT

>Seq13 [organism=Bradyrhizobium sp. TUTVUMr84] [strain=TUTVUMr84] *gyrB* gene for DNA gyrase subunit B, partial cds,

GCGTCGGCGTCTCCGTCGTCAACGCACTGTCGAGCAAGCTCGGCTTACGCATCTGGCGAG

ACGACAAGGAGCACTACATCGAGTTCGCCCATGGCGATGCGGTGGCACCGCTGAAAGTGG

TCGGCGACGCGCCGGGTCGGCGCGGCACCGAAGTGACGTTCCTCGCCTCGACCGAGACGT

TCAAGAACGTCGAGTACGATTTCGCCACGCTCGAGCATCGCCTGCGCGAGCTCGCCTTCC

TCAATTCGGGCGTCAACATCATCCTCTCCGACATGCGCCATGCGGTCGAGAAGCGCGAGG

AGATGCACTATTCCGGCGGCGTCGAGGAGTTCGTCAAATATCTCGATCGCAACAAGAAGG

CGCTGGTGCCCGCGCCGATCATGGTGCGCTCGGAAGCCAACGGCATCGGGGTCGAGGCCG

CGCTGTGGTGGAACGACAGCTACCACGAAAACGTGCTGTGCTTCACCAACAACATCCCGC

AGCGTGACGGTGGCACCCATCTCGCCGGCTTCCGCGGCGCGCTGACGCGCCAGGTCAACG

GCTATGCCGAAGCCAACGCCAAGAAGGAAAAGATCGCGCTGACCGGCGACGACTGCCGCG

AGGGCCTCACCGCCGTGCTGTCGGTGAAG

>Seq14 [organism=Bradyrhizobium sp.TUTVUMr85] [strain=TUTVUMr85] *gyrB* gene for DNA gyrase subunit B, partial cds,

GCGTCGGCGTCTCCGTCGTCAACGCACTGTCGAGCAAGCTCGGCCTGCGCATCTGGCGCG

ATGACAAGGAACATTACATCGAGTTCGCCCATGGCGATGCGGTGGCACCGCTCAAGGTCG

TCGGCGATGCGCCGGGCAAGCGCGGCACCGAGGTGACGTTCCTGGCCTCGAGCGAGACTT

TCAAGAACATCGAATATGATTTTGCGACGCTCGAGCATCGCCTGCGCGAGCTCGCCTTCC

TCAATTCCGGCGTCAACATCGCGCTGTCAGACATGCGTCACGCGGTCGAGAAGCGCGAGG

AGATGTATTATTCCGGCGGCGTCGAGGAATTCGTCAAATATCTCGACCGCAACAAGAAGG

CCCTGGTGCCCTCGCCGATCATGGTGCGTGCGGAAGCCAACGGCATCGGCGTCGAGGCCG

CCTTGTGGTGGAACGACAGCTACCATGAGAACGTGCTGTGCTTCACCAACAACATCCCGC

AGCGTGACGGCGGCACCCATTTGGCCGGCTTCCGCGGCGCGCTGACGCGCCAGGTCAACG

GTTATGCCGAGGCCAACGCGAAGAAGGAAAAGATCGCGCTCACCGGCGACGATTGCCGCG

AGGGCCTGACCGCGGTGCTGTCGGTGAAGGTGCCGGATCCGAAGTTTTCGTCGCAGACCA

GGG

>Seq15 [organism=Bradyrhizobium sp. TUTVUMr89] [strain=TUTVUMr89] *gyrB* gene for DNA gyrase subunit B, partial cds,

GCGTCGGCGTCTCCGTCGTCAACGCACTGTCCAGCAAGCTCGGCTTGCGGATCTGGCGCG

ACAACAAGGAGCACTACATCGAATTCGCCCATGGCGATGCCGTGGCGCCGCTCAAGATCG

TCGGCGATGCGCCGGGCAAGCGCGGCACCGAGGTGACGTTCCTGGCCTCGACCCAGACGT

TCAAGAACGTCGAATATGATTTCGCGACACTCGAGCATCGCCTGCGCGAGCTCGCCTTCC

TCAATTCCGGCGTCAACATCATCCTCTCCGACACGCGCCACGCGGTCGAGAAGCGCGAGG

AGATGCACTATTCCGGCGGCGTCGAGGAGTTCGTCAAATATCTCGACCGCAACAAGAAGG

CGATCGTGCCGGCGCCGATCATGGTGCGCTCGGAAGCCAACGGCATCGGCGTCGAGGCCG

CTTTGTGGTGGAACGACAGCTACCACGAGAACGTGCTCTGCTTCACCAACAACATCCCGC

AGCGTGACGGCGGCACCCATCTGGCCGGTTTCCGCGGCGCGCTGACGCGCCAGGTCAACG

GCTATGCCGAGGCCAATGCGAAAAAGGAAAAGATCGCGCTGACCGGCGACGATTGCCGCG

AAGGCCTCACCGCCGTGTTGTCGGTGAAGGTGCCCGATCCAAAGTTCTCGT

>Seq16 [organism=Bradyrhizobium sp. TUTVUMr95] [strain=TUTVUMr95] *gyrB* gene for DNA gyrase subunit B, partial cds,

GCGTCGGCGTCTCCGTCGTCAACGCACTGTCGAGCAAGCTCGGCCTGCGCATCTGGCGCG

ATGACAAGGAACATTACATCGAGTTCGCCCATGGCGATGCGGTGGCACCGCTCAAGGTCG

TCGGCGATGCGCCGGGCAAGCGCGGCACCGAGGTGACGTTCCTGGCCTCGAGCGAGACTT

TCAAGAACATCGAATATGATTTTGCGACGCTCGAGCATCGCCTGCGCGAGCTCGCCTTCC

TCAATTCCGGCGTCAACATCGCGCTGTCAGACATGCGTCACGCGGTCGAGAAGCGCGAGG

AGATGTATTATTCCGGCGGCGTCGAGGAATTCGTCAAATATCTCGACCGCAACAAGAAGG

CCCTGGTGCCCTCGCCGATCATGGTGCGTGCGGAAGCCAACGGCATCGGCGTCGAGGCCG

CCTTGTGGTGGAACGACAGCTACCATGAGAACGTGCTGTGCTTCACCAACAACATCCCGC

AGCGTGACGGCGGCACCCATTTGGCCGGCTTCCGCGGCGCGCTGACGCGCCAGGTCAACG

GTTATGCCGAGGCCAACGCGAAGAAGGAAAAGATCGCGCTCACCGGCGACGATTGCCGCG

AGGGCCTGACCGCGGTGCTGTCGGTGAAGGTGCCGGATCCGAAGTTTTCGTCGCA

>Seq17 [organism=Bradyrhizobium sp. TUTVUMr99] [strain=TUTVUMr99] *gyrB* gene for DNA gyrase subunit B, partial cds,

GGCGTCGGCGTCTCCGTCGTCAACGCGCTGTCCAGCAAGCTCGCGCTGCGGATCTGGCGC

GACGACAAGGAGCACTACATCGAGTTCGCGCACGGTGACGCCGTGGCGCCGCTCAAGGTG

GTCGGCGATGCGCCGGGCAAGCGCGGCACCGAGGTCACCTTCCTGGCCTCGACCGAGACC

TTCAAGAACATCGAGTATGATTTCGCGACGCTGGAGCACCGGCTGCGCGAGCTCGCCTTC

CTCAATTCCGGTGTCCACATCGTGCTGTCAGACATGCGCCACGCGGTCGAGAAGCGCGAG

GAGATGCATTATTCCGGCGGCGTCGAGGAATTCGTCAAATATCTCGACCGCAACAAGAAG

GCGATCGTACCGACGCCGATCATGGTGCGTTCGGAGGCGAACGGCATCGGCGTCGAGGCC

GCGCTGTGGTGGAACGACAGCTACCACGAGAACGTGCTGTGCTTCACCAACAACATCCCG

CAGCGTGACGGCGGCACCCATCTGGCCGGCTTCCGCGGCGCGCTGACGCGCCAGGTCAAC

GGCTATGCCGAGGCCAACGCGAAGAAGGAAAAGATCGCGCTGACCGGCGACGATTGCCGT

GAAGGTCTCACCGCGGTGCTGTCGGTGAAGGTGCCCGATCCAAAGTTCTCGT

>Seq18 [organism=Bradyrhizobium sp. TUTVUMr103] [strain=TUTVUMr103] *gyrB* gene for DNA gyrase subunit B, partial cds,

ACGGCGTCGGCGTCTCCGTCGTCAACGCGCTGTCGAGCAAGCTCGGCTTGCGCATCTGGC

GCGACAACAAGGAGCACTATATCGAGTTCGCCCACGGCGATGCCGTGGCACCGCTCAAGG

TCGTCGGCGATGCCCCGGGCAAGCGCGGCACCGAGGTGACGTTCCTGGCCTCGACCGAGA

CCTTCAAGAACATCGAATACGACTTCGCCACGCTCGAGCATCGCTTACGCGAGCTCGCCT

TCCTCAATTCCGGCGTCAACATCGTGCTCTCCGACATGCGCCACGCGGTCGAGAAGCGCG

AGGAGATGTTCTATTCCGGCGGCGTCGAGGAGTTCGTCAAATATCTCGACCGCAACAAGA

AGGCCCTGGTGCCGGCGCCGATCATGGTGCGCTCGGAAGCCAACGGCATCGGCGTCGAGG

CCGCTTTGTGGTGGAACGACAGCTACCACGAGAACGTGCTGTGCTTCACCAACAACATCC

CGCAACGCGACGGGGGCACCCATCTCGCCGGCTTCCGCGGCGCGCTGACGCGCCAGGTCA

ACGGCTATGCCGAGGCCAATGCCAAGAAGGAAAAGATCGCGCTGACCGGCGACGATTGCC

GCGAAGGGCTCACCGCCGTTCTCTCGGTGAAGGTGCCCGATCCGAAGTTTTCGTCGCAGAC

CAGGGAACAAGCTA

>Seq19 [organism=Bradyrhizobium sp. TUTVUMm105] [strain=TUTVUMm105] *gyrB* gene for DNA gyrase subunit B, partial cds,

CTGCACGGCGTCGGCGTCTCCGTCGTCAACGCGCTGTCGAGCAAGCTCGGCTTGCGCA

TCTGGCGCGACAACAAGGAGCATTACATCGAGTTCGCCCACGGCGATGCCGTGGCGCCGC

TGAAGGTCGTCGGCGACGCCCCGGGCAAGCGCGGCACCGAAGTGACGTTCCTGGCCTCGA

CCGAGACCTTCAAGAACATCGAATATGATTTCGCTACGCTCGAGCATCGGCTGCGCGAGC

TCGCCTTCCTCAATTCCGGCGTCAACATTGCGCTCTCCGACATGCGCCACGCGGTCGAGA

AGCGCGAGGAGATGTACTATTCCGGCGGCGTCGAGGAGTTCGTCAAATATCTCGACCGCA

ACAAGAAGGCGCTGGTGCCCGCCCCGATCATGGTGCGCTCGGAAGCCAACGGCATCGGCG

TCGAGGCGGCGCTGTGGTGGAACGACAGCTACCACGAGAACGTGCTGTGCTTCACCAACA

ACATCCCGCAGCGTGACGGCGGCACCCATCTGGCCGGCTTCCGCGGCGCGCTGACGCGCC

AGGTCAACGGCTATGCCGAGGCCAATGCCAAGAAGGAAAAGATCGCGCTGACCGGCGACG

ATTGCCGCGAAGGTCTCACCGCCGTGCTCTCGGTGAAGGTGCCGGACCCG

>Seq20 [organism=Bradyrhizobium sp. TUTVUMm110] [strain=TUTVUMm110] *gyrB* gene for DNA gyrase subunit B, partial cds,

GCGCTGTCGAGCAAGCTCGGCCTGCGGATCTGGCGCGACGACAAGGAGCACTACATCGAG

TTCGCCCATGGCGATGCCGTCGCACCGCTGAAGGTGGTCGGCGACGCACCGGGCCGGCGC

GGCACCGAGGTGACGTTTCTCGCCTCGACCGAGACGTTCAAGAACGTCGAATATGATTTC

GCCACGCTCGAGCATCGTTTGCGCGAGCTCGCCTTCCTCAATTCCGGCGTCAACATCATC

CTCTCCGACATGCGTCACGCGGTCGAGAAGCGCGAGGAGATGCATTATTCCGGCGGCGTC

GAGGAATTCGTCAAATATCTCGATCGCAACAAGAAGGCCTTGGTGCCGGCGCCGATCATG

GTGCGCTCCGAAGCCAACGGCATCGGCGTCGAGGCCGCCTTGTGGTGGAACGACAGCTAC

CACGAGAACGTGCTGTGCTTCACCAACAACATCCCGCAGCGTGACGGCGGCACCCATCTC

GCCGGCTTCCGCGGCGCGCTGACGCGCCAGGTCAACGGCTATGCCGAGGCCAATGCGAAA

AAGGAAAAGATCGCGCTGACCGGCGACGATTGCCGCGAAGGCCTCACGGCCGTGCTGTCG

GTGAAGGTGCCCGATCCGAAATTCTCGT

>Seq21 [organism=Bradyrhizobium sp. TUTVUMm112] [strain=TUTVUMm112] *gyrB* gene for DNA gyrase subunit B, partial cds,

GCGTCGGCGTCTCCGTCGTCAACGCGCTGTCGAGCAAGCTCGGCTTGCGCATCTGGCGTG

ACAACAAGGAGCACTATATCGAGTTCGCTCACGGCGATGCCGTCGCACCGCTGAAGGTGG

TCGGCGATGCGCCGGGCAAGCGCGGCACCGAGGTGACGTTCCTGGCCTCGACCGAGACCT

TCAAGAACATCGAATACGACTTCGCCACGCTCGAGCATCGCTTACGCGAGCTCGCCTTCC

TCAATTCCGGCGTCAACATCGTGCTCTCCGACATGCGCCACGCGGTCGAGAAGCGCGAGG

AGATGTTCTATTCCGGCGGCGTCGAGGAGTTCGTCAAATATCTCGACCGCAACAAGAAGG

CCCTGGTGCCGGCGCCGATCATGGTGCGCTCGGAAGCCAACGGCATCGGCGTCGAGGCCG

CTTTGTGGTGGAACGACAGCTACCACGAGAACGTGCTGTGCTTCACCAACAACATCCCGC

AACGCGACGGGGGCACCCATCTCGCCGGCTTCCGCGGCGCGCTGACGCGCCAGGTCAACG

GCTATGCCGAGGCCAATGCCAAGAAGGAAAAGATCGCGCTGACCGGCGACGATTGCCGCG

AAGGGCTCACCGCCGTTCTCTCGGTGAAGGTGCCCGATCCGAAGTTTTCGT

>Seq22 [organism=Bradyrhizobium sp. TUTVUMm113] [strain=TUTVUMm113] *gyrB* gene for DNA gyrase subunit B, partial cds,

GCGTCGGCGTCTCCGTCGTCAACGCGCTGTCGAGCAAGCTCGGCTTGCGCATCTGGCGTG

ACAACAAGGAGCACTATATCGAGTTCGCTCACGGCGATGCCGTCGCACCGCTGAAGGTG

GTCGGCGATGCGCCGGGCAAGCGCGGCACCGAGGTGACGTTCCTGGCCTCGACCGAGAC

CTTCAAGAACATCGAATACGACTTCGCCACGCTCGAGCATCGCTTACGCGAGCTCGCCT

TCCTCAATTCCGGCGTCAACATCGTGCTCTCCGACATGCGCCACGCGGTCGAGAAGCGC

GAGGAGATGTTCTATTCCGGCGGCGTCGAGGAGTTCGTCAAATATCTCGACCGCAACAA

GAAGGCCCTGGTGCCGGCGCCGATCATGGTGCGCTCGGAAGCCAACGGCATCGGCGTCG

AGGCCGCTTTGTGGTGGAACGACAGCTACCACGAGAACGTGCTGTGCTTCACCAACAAC

ATCCCGCAACGCGACGGGGGCACCCATCTCGCCGGCTTCCGCGGCGCGCTGACGCGCCA

GGTCAACGGCTATGCCGAGGCCAATGCCAAGAAGGAAAAGATCGCGCTGACCGGCGACG

ATTGCCGCGAAGGGCTCACCGCCGTTCTCTCGGTGAAGGTGCCCGATCCGAAGTTTTCGTCG

>Seq23 [organism=Bradyrhizobium sp. TUTVUMm114] [strain=TUTVUMm114] *gyrB* gene for DNA gyrase subunit B, partial cds,

GGCGTCGGCGTCTCCGTCGTCAACGCGCTGTCGAGCAAGCTCGGCTTGCGCATCTGGCGT

GACAACAAGGAGCACTATATCGAGTTCGCTCACGGCGATGCCGTCGCACCGCTGAAGGTG

GTCGGCGATGCGCCGGGCAAGCGCGGCACCGAGGTGACGTTCCTGGCCTCGACCGAGACC

TTCAAGAACATCGAATACGACTTCGCCACGCTCGAGCATCGCTTACGCGAGCTCGCCTTC

CTCAATTCCGGCGTCAACATCGTGCTCTCCGACATGCGCCACGCGGTCGAGAAGCGCGAG

GAGATGTTCTATTCCGGCGGCGTCGAGGAGTTCGTCAAATATCTCGACCGCAACAAGAAG

GCCCTGGTGCCGGCGCCGATCATGGTGCGCTCGGAAGCCAACGGCATCGGCGTCGAGGCC

GCTTTGTGGTGGAACGACAGCTACCACGAGAACGTGCTGTGCTTCACCAACAACATCCCG

CAACGCGACGGGGGCACCCATCTCGCCGGCTTCCGCGGCGCGCTGACGCGCCAGGTCAAC

GGCTATGCCGAGGCCAATGCCAAGAAGGAAAAGATCGCGCTGACCGGCGACGATTGCCGC

GAAGGGCTCACCGCCGTTCTCTCGGTGAAGGTGCCCGATCCGAAGTTTTCGTCGCAGACC

AGGGAACAAGCT

>Seq24 [organism=Bradyrhizobium sp. TUTVUI122] [strain=TUTVUI122] *gyrB* gene for DNA gyrase subunit B, partial cds,

TCGGAGTCTCAGTCGTCAACGCGCTGTCGAGCAAGCTCGGTCTGCGGATCTGGCGCGACG

ACAAGGAGCACTACATCGAGTTCGCCCATGGCGATGCCGTCGCACCGCTGAAGGTGGTCG

GCGACGCACCGGGCCGGCGCGGCACCGAGGTGACGTTTCTCGCCTCGACCGAGACGTTCA

AGAACGTCGAATATGATTTCGCCACGCTCGAGCATCGTTTGCGCGAGCTCGCCTTCCTCA

ATTCCGGCGTCAACATCATCCTCTCCGACATGCGTCACGCGGTCGAGAAGCGCGAGGAGA

TGCATTATTCCGGCGGCGTCGAGGAATTCGTCAAATATCTCGATCGCAACAAGAAGGCCT

TGGTGCCGGCGCCGATCATGGTGCGCTCCGAAGCCAACGGCATCGGCGTCGAGGCCGCCT

TGTGGTGGAACGACAGCTACCACGAGAACGTGCTGTGCTTCACCAACAACATCCCGCAGC

GTGACGGCGGCACCCATCTCGCCGGCTTCCGCGGCGCGCTGACGCGCCAGGTCAACGGCTA

TGCCGAGGCCAATGCGAAAAAGGAAAAGATCGCGCTGACCGGCGACGATTGCCGCGAAGGC

CTCACGGCCGTGCTGTCGGTGAAGGTGCCCGATCCGAAATTCTCGTCGCAGAC

>Seq25 [organism=Bradyrhizobium sp. TUTVUMm115] [strain=TUTVUMm115] gyrB gene for DNA gyrase subunit B, partial cds,

CGGCCTGCACGGCGTCGGCGTCTCCGTCGTCAACGCGCTGTCGAGCAAGCTCGGCTTGC

GCATCTGGCGCGACAACAAGGAGCACTATATCGAGTTCGCTCACGGCGATGCCGTCGCAC

CGCTGAAGGTGGTCGGCGATGCGCCGGGCAAGCGCGGCACCGAGGTGACGTTCCTGGCCT

CGACCGAGACCTTCAAGAACATCGAATACGACTTCGCCACGCTCGAGCATCGCTTACGCG

AGCTCGCCTTCCTCAATTCCGGCGTCAACATCGTGCTCTCCGACATGCGCCACGCGGTCG

AGAAGCGCGAGGAGATGTTCTATTCCGGCGGCGTCGAGGAGTTCGTCAAATATCTCGACC

GCAACAAGAAGGCCCTGGTGCCGGCGCCGATCATGGTGCGCTCGGAAGCCAACGGCATCG

GCGTCGAGGCCGCTTTGTGGTGGAACGACAGCTACCACGAGAACGTGCTGTGCTTCACCA

ACAACATCCCGCAACGCGACGGGGGCACCCATCTCGCCGGCTTCCGCGGCGCGCTGACGC

GCCAGGTCAACGGCTATGCCGAGGCCAATGCCAAGAAGGAAAAGATCGCGCTGACCGGCG

ACGATTGCCGCGAAGGGCTCACCGCCGTTCTCTCGGTGAAGGTGCCCGATCCGAAGTTTT

CGTCGCAGACCAGGGAACAAGCT

>Seq26 [organism=Bradyrhizobium sp. TUTVUMm108] [strain=TUTVUMm108] gyrB gene for DNA gyrase subunit B, partial cds,

CGGGCTGCACGGCGTCGGCGTCTCAGTCGTCAACGCGCTGTCGAGCAAGCTCGGCCTGC

GGATCTGGCGCGACGACAAGGAGCACTACATCGAGTTCGCCCATGGCGATGCCGTCGCAC

CGCTGAAGGTGGTCGGCGACGCACCGGGCCGGCGCGGCACCGAGGTGACGTTTCTCGCCT

CGACCGAGACGTTCAAGAACGTCGAATATGATTTCGCCACGCTCGAGCATCGTTTGCGCG

AGCTCGCCTTCCTCAATTCCGGCGTCAACATCATCCTCTCCGACATGCGTCACGCGGTCG

AGAAGCGCGAGGAGATGCATTATTCCGGCGGCGTCGAGGAATTCGTCAAATATCTCGATC

GCAACAAGAAGGCCTTGGTGCCGGCGCCGATCATGGTGCGCTCCGAAGCCAACGGCATCG

GCGTCGAGGCCGCCTTGTGGTGGAACGACAGCTACCACGAGAACGTGCTGTGCTTCACCA

ACAACATCCCGCAGCGTGACGGCGGCACCCATCTCGCCGGCTTCCGCGGCGCGCTGACGC

GCCAGGTCAACGGCTATGCCGAGGCCAATGCGAAAAAGGAAAAGATCGCGCTGACCGGCG

ACGATTGCCGCGAAGGCCTCACGGCCGTGCTGTCGGTGAAGGTGCCCGATCCGAAATTCT

CGTCGCAGAC

>Seq27 [organism=Bradyrhizobium sp. TUTVUMr93] [strain=TUTVUMr93] gyrB gene for DNA gyrase subunit B, partial cds,

GGCCTGCACGGCGTCGGCGTCTCCGTCGTCAACGCACTGTCGAGCAAGCTCGGCCTGCGC

ATCTGGCGCGATGACAAGGAACATTACATCGAGTTCGCCCATGGCGATGCGGTGGCACCG

CTCAAGGTCGTCGGCGATGCGCCGGGCAAGCGCGGCACCGAGGTGACGTTCCTGGCCTCG

AGCGAGACTTTCAAGAACATCGAATATGATTTTGCGACGCTCGAGCATCGCCTGCGCGAG

CTCGCCTTCCTCAATTCCGGCGTCAACATCGCGCTGTCAGACATGCGTCACGCGGTCGAG

AAGCGCGAGGAGATGTATTATTCCGGCGGCGTCGAGGAATTCGTCAAATATCTCGACCGC

AACAAGAAGGCCCTGGTGCCCTCGCCGATCATGGTGCGTGCGGAAGCCAACGGCATCGGC

GTCGAGGCCGCCTTGTGGTGGAACGACAGCTACCATGAGAACGTGCTGTGCTTCACCAAC

AACATCCCGCAGCGTGACGGCGGCACCCATTTGGCCGGCTTCCGCGGCGCGCTGACGCGC

CAGGTCAACGGTTATGCCGAGGCCAACGCGAAGAAGGAAAAGATCGCGCTCACCGGCGAC

GATTGCCGCGAGGGCCTGACCGCGGTGCTGTCGGTGAAGGTGCCGGATCCGAAGTTTTCG

TCGCAGACCA

>Seq28 [organism=Bradyrhizobium sp. TUTVUMm116] [strain=TUTVUMm116] gyrB gene for DNA gyrase subunit B, partial cds,

GGCGGGCTGCACGGCGTCGGCGTCTCCGTCGTCAACGCGCTGTCGAGCAAGCTCGGCTTG

CGCATCTGGCGTGACAACAAGGAGCACTATATCGAGTTCGCTCACGGCGATGCCGTCGCA

CCGCTGAAGGTGGTCGGCGATGCGCCGGGCAAGCGCGGCACCGAGGTGACGTTCCTGGCC

TCGACCGAGACCTTCAAGAACATCGAATACGACTTCGCCACGCTCGAGCATCGCTTACGC

GAGCTCGCCTTCCTCAATTCCGGCGTCAACATCGTGCTCTCCGACATGCGCCACGCGGTC

GAGAAGCGCGAGGAGATGTTCTATTCCGGCGGCGTCGAGGAGTTCGTCAAATATCTCGAC

CGCAACAAGAAGGCCCTGGTGCCGGCGCCGATCATGGTGCGCTCGGAAGCCAACGGCATC

GGCGTCGAGGCCGCTTTGTGGTGGAACGACAGCTACCACGAGAACGTGCTGTGCTTCACC

AACAACATCCCGCAACGCGACGGGGGCACCCATCTCGCCGGCTTCCGCGGCGCGCTGACG

CGCCAGGTCAACGGCTATGCCGAGGCCAATGCCAAGAAGGAAAAGATCGCGCTGACCGGC

GACGATTGCCGCGAAGGGCTCACCGCCGTTCTCTCGGTGAAGGTGCCCGATCCGAAGTTT

TCGTCGCAGACCAGGGAACAAGCTA

>Seq29 [organism=Bradyrhizobium sp. TUTVUMm117] [strain=TUTVUMm117] gyrB gene for DNA gyrase subunit B, partial cds,

CGGCGTCGGCGTCTCCGTCGTCAACGCGCTCTCCAGCAAGCTCGCGCTGCGGATCTGGC

GCGACGACAAGGAGCACTACATCGAATTCGCGCACGGCGATGCCGTGGCGCCGCTCAAAG

TGGTCGGCGATGCCCCAGGCAAGCGCGGCACCGAGGTCACCTTCCTCGCCTCGGTCGAGA

CCTTCAAGAACATCGAATATGATTTCGCGACGCTAGAGCACCGGCTGCGCGAGCTCGCCT

TCCTCAATTCCGGCGTCAACATCGTGCTCTCCGACATGCGTCACGCGGTCGAGAAGCGCG

AGGAGATGCACTATTCCGGCGGCGTCGAGGAATTCGTCAAATATCTCGACCGCAACAAGA

AGGCGATCGTGCCGGCACCGATCATGGTGCGTTCGGAAGCCAACGGCATCGGCGTCGAGG

CGGCGCTGTGGTGGAACGACAGCTACCACGAGAACGTGCTGTGCTTCACCAACAACATCC

CGCAGCGCGACGGCGGCACCCATCTGGCCGGCTTCCGCGGCGCGCTGACGCGCCAGGTCA

ACGGCTATGCCGAGGCCAATGCGAAGAAGGAAAAGATCGCGCTGACCGGCGACGATTGCC

GCGAAGGCCTCACCGCGGTGCTGTCGGTGAAGGTGCCCGATCCGAAATTCTCGT

>Seq30 [organism=Bradyrhizobium sp. TUTVUMr94] [strain=TUTVUMr94] gyrB gene for DNA gyrase subunit B, partial cds,

TCCGGCGGCCTGCACGGCGTCGGCGTCTCCGTCGTCAACGCGCTGTCGAGCAAGCTCGGC

TTGCGCATCTGGCGCGACAACAAGGAGCATTACATCGAGTTCGCCCATGGCGATGCCGTG

GCGCCGCTGAAGGTCGTCGGCGACGCCCCGGGCAAGCGCGGCACCGAGGTGACGTTCCTG

GCCTCGACCGAGACCTTCAAGAACATCGAATATGATTTCGCTACCCTCGAGCATCGGCTG

CGCGAGCTCGCCTTCCTCAATTCCGGCGTCAACATTGCGCTCTCCGACATGCGCCACGCG

GTCGAGAAGCGCGAGGAGATGTACTATTCCGGCGGCGTCGAGGAGTTCGTCAAATATCTC

GACCGCAACAAGAAGGCGCTGGTGCCCGCCCCGATCATGGTGCGCTCGGAAGCCAACGGC

ATCGGCGTCGAGGCGGCGCTGTGGTGGAACGACAGCTACCACGAGAACGTGCTGTGCTTC

ACCAACAACATCCCGCAGCGTGACGGCGGCACCCATCTGGCCGGCTTCCGCGGCGCGCTG

ACGCGCCAGGTCAACGGCTATGCCGAGGCCAATGCCAAGAAGGAAAAGATCGCGCTGACC

GGCGACGATTGCCGCGAAGGTCTCACCGCCGTGCTCTCGGTGAAGGTGCCGGACCCG

***recA***

>Seq1 [organism=Bradyrhizobium sp. TUTVUMp5] [strain=TUTVUMp5] *recA* gene for recombinase A, partial cds,

GATGAAGCTCGGCAAGAGCGACCGCTCCATGGACATCGAGGCGGTGTCCTCGGGCTCGCT

CGGGCTCGATATCGCGCTCGGCATCGGCGGCCTGCCCAAGGGACGCATCGTCGAGATCTA

CGGGCCGGAATCGTCGGGCAAGACCACGCTGGCCCTGCATACGGTGGCGGAAGCGCAGAA

GAAGGGAGGAATCTGCGCCTTCATCGACGCCGAGCACGCGCTCGATCCGGTCTATGCCCG

CAAGCTCGGCGTCAACATTGACGAACTGCTGATCTCGCAGCCCGACACCGGCGAGCAGGC

GTTGGAAATTTGCGACACCTTGGTGCGCTCCGGCGCTGTGGACGTGCTGGTGGTCGATTC

GGTTGCGGCGCTGGTGCCGAAGGCCGAGCTCGAGGGCGAGATGGGCGATGCGCTGCCGGG

TCTGCAGGCCCGCCTGATGAGCCAGGCGCTGCGCAAGCTCACGGCCTCGATCAACAAGTC

CAACAC

>Seq2 [organism=Bradyrhizobium sp. TUTVUMp6] [strain=TUTVUMp6] *recA* gene for recombinase A, partial cds,

CCTCCATGGACAAGAGTAAAGCTCTGGCCGCCGCGCTCTCCCAGATCGAGCGGCAGTTCGGCA

AGGGCTCGGTGATGAAGCTCGGCAAGAACGATCGCTCCATGGACATCGAGGCGGTGTCCTCCG

GCTCGCTCGGGCTCGATATCGCGCTCGGCATCGGCGGCCTGCCCAAGGGACGCATCGTCGAGA

TCTACGGGCCGGAATCCTCGGGCAAGACCACGCTGGCGCTGCATACGGTGGCGGAAGCGCAGA

AGAAGGGCGGCATCTGCGCCTTCATCGACGCCGAGCACGCGCTCGATCCGGTCTATGCCCGCA

AGCTCGGAGTCAACATCGACGAGCTCCTGATCTCGCAGCCCGACACCGGCGAGCAGGCCCTGG

AGATCTGCGACACCCTGGTGCGCTCGGGCGCGGTCGATGTTCTCGTGGTCGATTCGGTGGCGG

CGCTGGTGCCGAAGGCCGAGCTCGAGGGCGAGATGGGCGATGCGCTGCCAGGCCTGCAGGCCC

GTCTGATGAGCCAGGCGCTGCGCAAGCTGACGGCCTCCATCAACAAGTCCAACACCATGGTGA

TCTTCACAGGGCAGATACGAGCGACCATTCTAATTCCAGTGGGGGGCAAGGCCGACAGCCGAC

CCGCCAGCTGCCCGCCCCGACGCCCCGCCACCTCGCGCACCCCAGCTCGCGCCCGCCGGGCCG

CGCGGGGGGCCGCCGCCCCCGGC

>Seq3 [organism=Bradyrhizobium sp. TUTVUMp8] [strain=TUTVUMp8] *recA* gene for recombinase A, partial cds,

CATGGACAAGAGTAAAGCCCTGGCTGCCGCGCTCTCTCAGATCGAGCGCCAGTTCGGCAA

GGGATCGGTGATGAAGCTCGGCAAGAGCGACCGCTCCATGGACATCGAGGCGGTGTCCTC

GGGCTCGCTCGGGCTCGATATCGCGCTCGGCATCGGCGGCCTGCCCAAGGGACGCATTGT

CGAGATCTACGGGCCGGAATCGTCGGGCAAGACCACGCTGGCGCTGCATACGGTGGCGGA

AGCGCAGAAGAAGGGCGGCATCTGCGCCTTCATCGATGCCGAGCACGCGCTCGACCCGGT

CTATGCCCGCAAGCTCGGCGTCAACATCGACGAGTTGCTGATCTCGCAGCCCGACACCGG

CGAGCAGGCGCTGGAAATCTGCGACACCTTGGTGCGCTCCGGCGCGGTCGACGTGCTCGT

GGTCGATTCGGTTGCGGCGCTGGTGCCGAAGGCCGAGCTCGAGGGCGAGATGGGCGATGC

GCTGCCAGGTCTGCAAGCCCGCCTGATGAGCCAGGCGCTGCGCAAGCTCACGGCCTCGAT

CAACAAGTCCAACACCATGGTGATCTTC

>Seq4 [organism=Bradyrhizobium sp. TUTVUMp48] [strain=TUTVUMp48] *recA* gene for recombinase A, partial cds,

AGAGTAAAGCCCTGGCTGCCGCGCTCTCTCAGATCGAGCGCCAGTTCGGCAAGGGATCGG

TGATGAAGCTCGGCAAGAGCGACCGCTCCATGGACATCGAGGCGGTGTCCTCGGGCTCGC

TCGGGCTCGATATCGCGCTCGGCATCGGCGGCCTGCCCAAGGGACGCATTGTCGAGATCT

ACGGGCCGGAATCGTCGGGCAAGACCACGCTGGCGCTGCATACGGTGGCGGAAGCGCAGA

AGAAGGGCGGCATCTGCGCCTTCATCGATGCCGAGCACGCGCTCGACCCGGTCTATGCCC

GCAAGCTCGGCGTCAACATCGACGAGTTGCTGATCTCGCAGCCCGACACCGGCGAGCAGG

CGCTGGAAATCTGCGACACCTTGGTGCGCTCCGGCGCGGTCGACGTGCTCGTGGTCGATT

CGGTTGCGGCGCTGGTGCCGAAGGCCGAGCTCGAGGGCGAGATGGGCGATGCGCTGCCAG

GTCTGCAAGCCCGCCTGATGAGCCAGGCGCTGCGCAAGCTCACGGCCTCGATCAACAAGT

CCAACACCATGGTGATCTTC

>Seq5 [organism=Bradyrhizobium sp. TUTVUMp50] [strain=TUTVUMp50] *recA* gene for recombinase A, partial cds,

AGTAAAGCCCTGGCTGCCGCGCTCTCTCAGATCGAGCGCCAGTTCGGCAAGG

GATCGGTGATGAAGCTCGGCAAGAGCGACCGCTCCATGGACATCGAGGCGGTGTCCTCG

GGCTCGCTCGGGCTCGATATCGCGCTCGGCATCGGCGGCCTGCCCAAGGGACGCATTGT

CGAGATCTACGGGCCGGAATCGTCGGGCAAGACCACGCTGGCGCTGCATACGGTGGCGG

AAGCGCAGAAGAAGGGCGGTATCTGCGCCTTCATCGATGCCGAGCACGCGCTCGACCCG

GTCTATGCCCGCAAGCTCGGCGTCAACATCGACGAGTTGCTGATCTCGCAGCCCGACAC

CGGCGAGCAGGCGCTGGAAATCTGCGACACCTTGGTGCGCTCCGGCGCGGTCGACGTGC

TGGTGGTCGATTCGGTTGCGGCGCTGGTGCCGAAGGCCGAGCTCGAGGGCGAGATGGGC

GATGCGCTGCCAGGTCTGCAAGCCCGCCTGATGAGCCAGGCGCTGCGCAAGCTCACGGC

CTCGATCAACAAGTCCAACACCATGGTGATCATC

>Seq6 [organism=Bradyrhizobium sp. TUTVUMp53] [strain=TUTVUMp53] *recA* gene for recombinase A, partial cds,

AGAGTAAAGCCCTGGCTGCCGCGCTCTCTCAGATCGAGCGCCAGTTCGGCAAGGGATCGG

TGATGAAGCTCGGCAAGAGCGACCGCTCCATGGACATCGAGGCGGTGTCCTCGGGCTCGC

TCGGGCTCGATATCGCGCTCGGCATCGGCGGCCTGCCCAAGGGACGCATTGTCGAGATCT

ACGGGCCGGAATCGTCGGGCAAGACCACGCTGGCGCTGCATACGGTGGCGGAAGCGCAGA

AGAAGGGCGGTATCTGCGCCTTCATCGATGCCGAGCACGCGCTCGACCCGGTCTATGCCC

GCAAGCTCGGCGTCAACATCGACGAGTTGCTGATCTCGCAGCCCGACACCGGCGAGCAGG

CGCTGGAAATCTGCGACACCTTGGTGCGCTCCGGCGCGGTCGACGTGCTGGTGGTCGATT

CGGTTGCGGCGCTGGTGCCGAAGGCCGAGCTCGAGGGCGAGATGGGCGATGCGCTGCCAG

GTCTGCAAGCCCGCCTGATGAGCCAGGCGCTGCGCAAGCTCACGGCCTCGATCAACAAGT

CCAACACCATGGTGATCT

>Seq7 [organism=Bradyrhizobium sp. TUTVUMp56] [strain=TUTVUMp56] *recA* gene for recombinase A, partial cds,

AGAGTAAAGCCCTGGCTGCCGCGCTCTCTCAGATCGAGCGCCAGTTCGGCAAGGGATCGG

TGATGAAGCTCGGCAAGAGCGACCGCTCCATGGACATCGAGGCGGTGTCCTCGGGCTCGC

TCGGGCTCGATATCGCGCTCGGCATCGGCGGCCTGCCCAAGGGACGCATCGTCGAGATCT

ACGGGCCGGAATCGTCGGGCAAGACCACGCTGGCGCTGCATACGGTGGCGGAAGCGCAGA

AGAAGGGTGGCATCTGCGCCTTCATCGACGCCGAGCACGCGCTCGATCCGGTCTATGCCC

GCAAGCTCGGCGTCAACATTGACGAACTGCTGATCTCGCAGCCCGACACCGGCGAGCAGG

CGTTGGAAATTTGCGACACCTTGGTGCGCTCCGGCGCTGTGGACGTGCTGGTGGTCGATT

CGGTTGCGGCGCTGGTGCCGAAGGCCGAGCTCGAGGGCGAGATGGGCGATGCGCTGCCGG

GTCTGCAGGCCCGCCTGATGAGCCAGGCGCTGCGCAAGCTCACGGCCTCGATCAACAAGT

CCAACAC

>Seq8 [organism=Bradyrhizobium sp. TUTVUMp60] [strain=TUTVUMp60] *recA* gene for recombinase A, partial cds,

TGGACAAGTCCAAGGCCCTCTCAGCCGCGCTCTCCCAGATCGAGCGCCAGTTCGGCAAGG

GCTCGGTGATGAAGCTCGGCAAGAACGACCGTTCGATGGATGTCGAGACGGTGTCGTCGG

GGTCGCTCGGGCTCGATATTGCGCTCGGCGTCGGCGGTCTGCCGAAGGGACGCGTCGTCG

AAATCTACGGGCCGGAATCCTCGGGCAAGACCACGCTGGCGCTGCACACCGTGGCGGAAG

GCCAGAAGAAGGGCGGCATCTGCGCCTTCATCGACGCTGAACACGCGCTCGACCCGGTCT

ATGCGCGCAAGCTCGGCGTCAACATCGACGAGCTCTTGATCTCGCAGCCGGACACCGGCG

AGCAGGCGCTGGAGATTTGCGACACGCTGGTGCGCTCCGGCGCGGTCGACGTGCTGGTGG

TCGATTCGGTTGCGGCGCTGGTGCCGAAGGCCGAGCTCGAGGGCGAGATGGGCGATGCGT

TGCCGGGTCTGCAGGCCCGCCTGATGAGCCAGGCGCTGCGCAAGCTGACCGCCTCGATCA

ACAAGTCCCACACCATGGTGATCTTC

>Seq9 [organism=Bradyrhizobium sp. TUTVUMp65] [strain=TUTVUMp65] *recA* gene for recombinase A, partial cds,

CGCCAGTTCGGCAAGGGATCGGTGATGAAGCTCGGCAAGAGCGACCGCTCCATGGACATC

GAGGCGGTGTCCTCGGGCTCGCTCGGGCTCGATATCGCGCTCGGCATCGGCGGCCTGCCC

AAGGGACGCATTGTCGAGATCTACGGGCCGGAATCGTCGGGCAAGACCACGCTGGCGCTG

CATACGGTGGCGGAAGCGCAGAAGAAGGGCGGTATCTGCGCCTTCATCGATGCCGAGCAC

GCGCTCGACCCGGTCTATGCCCGCAAGCTCGGCGTCAACATCGACGAGTTGCTGATCTCGC

AGCCCGACACCGGCGAGCAGGCGCTGGAAATCTGCGACACCTTGGTGCGCTCCGGCGCGG

TCGACGTGCTGGTGGTCGATTCGGTTGCGGCGCTGGTGCCGAAGGCCGAGCTCGAGGGCG

AGATGGGCGATGCGCTGCCAGGTCTGCAAGCCCGCCTGATGAGCCAGGCGCTGCGCAAGCT

CACGGCCTCGATCAACAAGTCCAACACCATGGCGATCTTC

>Seq10 [organism=Bradyrhizobium sp. TUTVUMp75] [strain=TUTVUMp75] *recA* gene for recombinase A, partial cds,

AAGTCCAAGGCCCTCTCAGCCGCGCTCTCTCAGATCGAGCGCCAGTTCGGCAAGGGCTCG

GTGATGAAGCTCGGCAAGAACGACCGTTCGATGGATGTCGAGACGGTGTCGTCGGGGTCG

CTCGGGCTCGATATTGCGCTCGGCGTCGGCGGTCTGCCGAAGGGACGCGTCGTCGAAATC

TACGGGCCGGAATCCTCGGGCAAGACCACGCTGGCGCTGCACACGGTGGCGGAAGGCCAG

AAGAAGGGCGGCATCTGCGCCTTCATCGACGCCGAACACGCGCTCGATCCGGTCTATGCG

CGCAAGCTCGGCGTCAACATCGACGAGCTCTTGATCTCGCAGCCGGACACCGGCGAGCAG

GCGCTGGAGATTTGCGACACGCTGGTGCGCTCCGGCGCGGTCGACGTGCTGGTGGTCGAT

TCGGTTGCGGCGCTGGTGCCGAAGGCCGAGCTCGAGGGCGAGATGGGCGATGCGCTACCG

GGTCTGCAGGCCCGCCTGATGAGCCAGGCGCTGCGCAAGCTGACCGCCTCGATCAACAAG

TCCCACACCATGGTGATC

>Seq11 [organism=Bradyrhizobium sp. TUTVUMr82] [strain=TUTVUMr82] *recA* gene for recombinase A, partial cds,

TCTCTCAGATCGAGCGCCAATTCGGCAAGGGCTCTGTGATGAAGCTGGGCAAGAACGAC

CGCTCCATGGACATCGAGGCGGTGTCCTCAGGCTCGCTCGGGCTCGATATCGCGCTTGG

CATCGGCGGCCTTCCCAAGGGGCGCATCGTCGAGATCTACGGGCCGGAATCCTCGGGCAA

GACCACGCTGGCGCTGCACACGGTGGCCGAAGCCCAGAAGAAGGGCGGAATCTGCGCCTT

CATCGACGCCGAGCACGCGCTCGACCCGGTCTATGCCCGCAAGCTCGGCGTCAACATCG

ACGAACTCCTCATCTCGCAGCCCGACACCGGCGAGCAGGCGCTGGAGATCTGCGACACCC

TGGTGCGCTCCGGCGCGGTCGACGTGATGGTGGTCGATTCGGTTGCGGCGCTGGTGCCG

AAGGCCGAGCTCGAGGGCGAGATGGGCGAATCGCTGCCGGGTCTGCAGGCCCGTCTGAT

GAGCCAGGCGCTGCGCAAGCTGACGGCCTCGATCAACAAGTCCAACACCATGGTGAT

>Seq12 [organism=Bradyrhizobium sp. TUTVUMr85] [strain=TUTVUMr85] *recA* gene for recombinase A, partial cds,

AGAGTAAAGCCCTGGCTGCCGCGCTCTCTCAGATCGAGCGCCAGTTCGGCAAGGGATCGG

TGATGAAGCTCGGCAAGAGCGACCGCTCCATGGACATCGAGGCGGTGTCCTCCGGCTCGC

TCGGACTCGATATCGCGCTCGGCATCGGCGGCCTGCCCAAGGGACGCATCGTCGAGATCT

ACGGGCCGGAATCGTCGGGCAAGACTACGCTGGCGCTGCATACGGTGGCGGAAGCGCAGA

AGAAGGGCGGCATCTGCGCCTTCATCGATGCCGAGCACGCGCTCGACCCGGTCTATGCCC

GCAAGCTCGGCGTCAACATCGACGAGCTGCTGATCTCGCAGCCCGACACCGGTGAGCAGG

CGCTGGAAATCTGCGACACCTTGGTGCGCTCCGGCGCGGTCGACGTGCTGGTGGTCGATT

CGGTTGCGGCGCTGGTGCCGAAGGCCGAGCTCGAGGGCGAGATGGGCGATGCGCTGCCAG

GTCTGCAAGCCCGCCTGATGAGCCAGGCGCTGCGCAAGCTTACGGCCTCGATCAACAAGT

CCAACACCATGGTGATCTT

>Seq13 [organism=Bradyrhizobium sp. TUTVUMr89] [strain=TUTVUMr89] *recA* gene for recombinase A, partial cds,

GGATCGGTGATGAAGCTCGGCAAGAGCGACCGCTCCATGGACATCGAGGCGGTGTCCTCC

GGCTCTCTCGGGCTCGATATCGCGCTCGGCATCGGCGGCCTGCCCAAGGGACGCATCGTC

GAGATCTACGGGCCGGAATCGTCGGGCAAGACTACGCTGGCGCTGCATACGGTGGCGGAAG

CGCAGAAGAAGGGCGGCATCTGCGCCTTCATCGATGCCGAGCACGCGCTCGACCCGGTCTA

TGCCCGCAAGCTCGGCGTCAACATCGACGAGCTGCTGATCTCGCAGCCCGACACCGGTGA

GCAGGCGCTGGAAATCTGCGACACCTTGGTGCGCTCCGGCGCGGTCGACGTGCTGGTGGT

CGATTCGGTTGCGGCGCTGGTGCCGAAGGCCGAGCTCGAGGGCGAGATGGGCGATGCGCT

GCCAGGTCTGCAAGCCCGCCTGATGAGCCAGGCGCTGCGCAAGCTTACGGCCTCGATCAA

CAAGTCCAACACCATGGTGATC

>Seq14 [organism=Bradyrhizobium sp. TUTVUMr93] [strain=TUTVUMr93] *recA* gene for recombinase A, partial cds,

GGACAAGAGTAAAGCTCTGGCCGCCGCGCTCTCCCAGATCGAGCGGCAGTTCGGCAAGGG

CTCGGTGATGAAGCTCGGCAAGAACGACCGCTCCATGGACATCGAGGCGGTGTCCTCCGG

CTCGCTCGGGCTCGATATCGCGCTGGGCATCGGCGGCCTGCCCAAGGGACGCATCGTCGA

GATCTACGGGCCGGAATCTTCGGGCAAGACCACGCTCGCGCTGCATACGGTGGCGGAAGC

GCAGAAGAAGGGTGGCATCTGCGCCTTCATCGACGCCGAGCACGCGCTCGATCCGGTCTA

TGCCCGCAAGCTCGGCGTCAACATCGACGAGCTCCTGATCTCGCAGCCCGACACCGGCGA

GCAGGCGCTGGAGATCTGCGACACCCTGGTGCGCTCGGGCGCCGTCGATGTTCTGGTGGT

CGATTCGGTGGCGGCGCTGGTGCCGAAGGCCGAGCTCGAAGGCGAGATGGGTGACGCGCT

GCCTGGCCTGCAGGCCCGCCTGATGAGCCAGGCGCTGCGCAAGCTGACGGCATCCATCAA

CAAGTCCAACACCATGGTGATCTTC

>Seq15 [organism=Bradyrhizobium sp. TUTVUMr94] [strain=TUTVUMr94] *recA* gene for recombinase A, partial cds,

AGAGTAAAGCTCTGGCTGCCGCGCTCTCCCAGATCGAGCGTCAGTTCGGCAAGGGCTCGG

TGATGAAGCTCGGCAAGAACGACCGTTCGATGGACATCGAGGCAGTGTCGTCAGGCTCGC

TCGGGCTCGATATCGCGCTCGGCATCGGCGGCCTGCCCAAGGGGCGCATCGTCGAGATCT

ACGGGCCGGAATCCTCAGGAAAAACCACGCTGGCGCTGCACACGGTGGCGGAAGCCCAGA

AGAAGGGCGGCATCTGCGCCTTCATCGACGCCGAGCACGCGCTCGACCCGGTCTATGCCC

GCAAGCTCGGGGTCAACATCGACGAGCTCTTGATCTCCCAGCCCGACACCGGCGAGCAGG

CGCTGGAAATCTGCGACACGTTGGTGCGCTCGGGCGCGGTGGACGTGCTGGTGGTCGATT

CGGTCGCGGCCCTGGTGCCGAAGGCCGAGCTCGAAGGCGAGATGGGTGATGCGCTGCCGG

GTCTTCAAGCGCGGTTGATGAGCCAGGCGCTGCGCAAGCTGACGGCCTCGATCAACAAGT

CCAATACCATGGTGATCTTCTC

>Seq16 [organism=Bradyrhizobium sp. TUTVUMr99] [strain=TUTVUMr99] *recA* gene for recombinase A, partial cds,

AGCTCTGGCTGCCGCGCTCTCTCAGATCGAGCGCCAGTTCGGCAAGGGCTCGGTGATGAA

GCTCGGCAAGAACGACCGCTCCATGGACATCGAGGCGGTGTCCTCCGGCTCGCTCGGGCT

CGATATCGCGCTCGGCATCGGCGGCCTGCCCAAGGGGCGCATCGTCGAGATCTACGGGCC

GGAATCGTCCGGCAAGACCACGCTGGCGCTGCATACGGTGGCGGAAGCGCAGAAGAAGGG

TGGCATCTGCGCCTTCATCGATGCCGAGCACGCGCTCGATCCGGTCTATGCCCGCAAGCT

CGGCGTCAACATCGACGAGCTCCTGATCTCGCAGCCCGACACCGGCGAGCAGGCGCTGGA

GATTTGCGACACCCTGGTGCGCTCGGGCGCGGTCGATGTTCTCGTGGTCGATTCGGTGGC

GGCGCTGGTGCCGAAGGCCGAGCTCGAGGGCGAGATGGGCGATGCGCTGCCGGGACTTCA

AGCACGGTTGATGAGCCAGGCGCTGCGCAAGCTCACGGCCTCCATCAACAAATCCAACAC

CATGGTGATCTTCATCACCC

>Seq17 [organism=Bradyrhizobium sp. TUTVUMr103] [strain=TUTVUMr103] *recA* gene for recombinase A, partial cds,

AGAGTAAAGCTCTGGCCGCCGCGCTCTCCCAGATCGAGCGGCAGTTCGGCAAGGGCTCGG

TGATGAAGCTCGGCAAGAACGACCGCTCAATGGATATCGAGGCGGTGTCCTCCGGCTCGC

TCGGGCTCGATATCGCGCTCGGCATCGGCGGCCTGCCGAAGGGACGCATCGTCGAGATCT

ACGGGCCGGAATCTTCGGGCAAGACCACGCTCGCGCTGCATACGGTGGCGGAAGCGCAGA

AGAAGGGTGGCATCTGCGCCTTCATCGACGCCGAGCACGCGCTCGATCCGGTCTATGCCC

GCAAGCTCGGCGTCAACATCGACGAGCTCCTGATCTCGCAGCCCGACACCGGCGAGCAGG

CGCTGGAGATCTGCGACACCCTGGTGCGCTCGGGCGCCGTCGATGTTCTGGTGGTCGATT

CGGTGGCGGCGCTGGTGCCGAAGGCCGAGCTCGAGGGCGAGATGGGTGACGCGCTGCCTG

GCCTGCAGGCCCGCCTGATGAGCCAGGCGCTGCGCAAGCTGACGGCCTCCATCAACAAGT

CCAACACCATGGTGATCTTC

>Seq18 [organism=Bradyrhizobium sp. TUTVUMm105] [strain=TUTVUMm105] *recA* gene for recombinase A, partial cds,

AGAGTAAAGCCCTGGCTGCCGCGCTCTCCCAGATCGAGCGCCAGTTCGGCAAGGGATCGG

TGATGAAGCTCGGCAAGAGCGACCGCTCCATGGACATCGAGGCGGTGTCTTCCGGCTCGC

TCGGGCTCGATATCGCGCTCGGCATCGGCGGTCTGCCGAAGGGGCGCATCGTCGAGATCT

ACGGGCCGGAATCCTCGGGCAAGACCACGCTGGCGCTGCACACGGTGGCGGAAGCACAGA

AGAAGGGCGGCATCTGCGCCTTCATCGACGCCGAGCACGCGCTCGACCCGGTCTATGCCC

GCAAGCTGGGCGTCAACATCGACGAGCTCCTGATCTCGCAGCCCGACACCGGCGAGCAGG

CGCTGGAAATTTGCGACACGCTGGTGCGTTCGGGCGCGGTGGACGTGCTGGTGGTCGATT

CGGTTGCGGCGCTGGTGCCGAAGGCCGAGCTCGAAGGCGAGATGGGCGATGCGCTGCCGG

GCCTGCAGGCACGTCTGATGAGCCAGGCGCTGCGCAAGCTGACGGCCTCCATCAACAAGT

CCAACAC

>Seq19 [organism=Bradyrhizobium sp. TUTVUMm108] [strain=TUTVUMp5] *recA* gene for recombinase A, partial cds,

AGAGTAAAGCCCTGGCTGCCGCGCTCTCCCAGATCGAGCGCCAGTTCGGCAAGGGATCGG

TGATGAAGCTCGGCAAGAGCGACCGCTCCATGGACATCGAGGCGGTGTCTTCCGGCTCGC

TCGGGCTCGATATCGCGCTCGGCATCGGCGGTCTGCCGAAGGGGCGCATCGTCGAGATCT

ACGGGCCGGAATCCTCGGGCAAGACCACGCTGGCGCTGCACACGGTGGCGGAAGCACAGA

AGAAGGGCGGCATCTGCGCCTTCATCGACGCCGAGCACGCGCTCGACCCGGTCTATGCCC

GCAAGCTGGGCGTCAACATCGACGAGCTCCTGATCTCGCAGCCCGACACCGGCGAGCAGG

CGCTGGAAATTTGCGACACGCTGGTGCGTTCGGGCGCGGTGGACGTGCTGGTGGTCGATT

CGGTTGCGGCGCTGGTGCCGAAGGCCGAGCTCGAAGGCGAGATGGGCGATGCGCTGCCGG

GCCTGCAGGCACGTCTGATGAGCCAGGCGCTGCGCAAGCTGACGGCCTCCATCAACAAGT

CCAACAC

>Seq20 [organism=Bradyrhizobium sp. TUTVUMm110] [strain=TUTVUMm110] *recA* gene for recombinase A, partial cds,

AGCTCTGGCTGCCGCGCTCTCTCAGATCGAGCGCCAGTTCGGCAAGGGCTCGGTGATGAA

GCTCGGCAAGAACGACCGCTCCATGGACATCGAGGCGGTGTCCTCCGGCTCGCTCGGGCT

CGATATCGCGCTCGGCATCGGCGGCCTGCCCAAGGGGCGCATCGTCGAGATCTACGGGCC

GGAATCGTCCGGCAAGACCACGCTGGCGCTGCATACGGTGGCGGAAGCGCAGAAGAAGGG

TGGCATCTGCGCCTTCATCGATGCCGAGCACGCGCTCGATCCGGTCTATGCCCGCAAGCT

CGGCGTCAACATCGACGAGCTCCTGATCTCGCAGCCCGACACCGGCGAGCAGGCGCTGGA

GATCTGCGACACCCTGGTGCGCTCGGGCGCGGTCGATGTTCTCGTGGTCGATTCGGTGGC

GGCGCTGGTGCCGAAGGCCGAGCTCGAGGGCGAGATGGGCGATGCGCTGCCGGGACTTCA

AGCACGGTTGATGAGCCAGGCGCTGCGCAAGCTCACGGCCTCCATCAACAAATCCAACAC

CATGGTGATCTTCATCACCC

>Seq21 [organism=Bradyrhizobium sp. TUTVUMm112] [strain=TUTVUMm112] *recA* gene for recombinase A, partial cds,

AGCTCTGGCTGCCGCGCTCTCTCAGATCGAGCGCCAGTTCGGCAAGGGCTCGGTGATGAA

GCTCGGCAAGAACGACCGCTCCATGGACATCGAGGCGGTGTCCTCCGGCTCGCTCGGGCT

CGATATCGCGCTCGGCATCGGCGGCCTGCCCAAGGGGCGCATCGTCGAGATCTACGGGCC

GGAATCGTCCGGCAAGACCACGCTGGCGCTGCATACGGTGGCGGAAGCGCAGAAGAAGGG

TGGCATCTGCGCCTTCATCGATGCCGAGCACGCGCTCGATCCGGTCTATGCCCGCAAGCT

CGGCGTCAACATCGACGAGCTCCTGATCTCGCAGCCCGACACCGGCGAGCAGGCGCTGGA

GATCTGCGACACCCTGGTGCGCTCGGGCGCGGTCGATGTTCTCGTGGTCGATTCGGTGGC

GGCGCTGGTGCCGAAGGCCGAGCTCGAGGGCGAGATGGGCGATGCGCTGCCGGGACTTCA

AGCACGGTTGATGAGCCAGGCGCTGCGCAAGCTCACGGCCTCCATCAACAAATCCAACAC

CATGGTGATCTTCATCAACC

>Seq22 [organism=Bradyrhizobium sp. TUTVUMm113] [strain=TUTVUMm113] *recA* gene for recombinase A, partial cds,

AGCTCTGGCTGCCGCGCTCTCTCAGATCGAGCGCCAGTTCGGCAAGGGCTCGGTGATGAA

GCTCGGCAAGAACGACCGCTCCATGGACATCGAGGCGGTGTCCTCCGGCTCGCTCGGGCT

CGATATCGCGCTCGGCATCGGCGGCCTGCCCAAGGGGCGCATCGTCGAGATCTACGGGCC

GGAATCGTCCGGCAAGACCACGCTGGCGCTGCATACGGTGGCGGAAGCGCAGAAGAAGGG

TGGCATCTGCGCCTTCATCGATGCCGAGCACGCGCTCGATCCGGTCTATGCCCGCAAGCT

CGGCGTCAACATCGACGAGCTCCTGATCTCGCAGCCCGACACCGGCGAGCAGGCGCTGGA

CATCTGCGACACCCTGGTGCGCTCGGGCGCGGTCGATGTTCTCGTGGTCGATTCGGTGGC

GGCGCTGGTGCCGAAGGCCGAGCTCGAGGGCGAGATGGGCGATGCGCTGCCGGGACTTCA

AGCACGGTTGATGAGCCAGGCGCTGCGCAAGCTCACGGCCTCCATCAACAAATCCAACAC

CATGGTGATCTTCATCAACC

>Seq23 [organism=Bradyrhizobium sp. TUTVUMm114] [strain=TUTVUMm114] *recA* gene for recombinase A, partial cds,

AGAGTAAAGCTCTGGCCGCCGCGCTCTCCCAGATCGAGCGGCAGTTCGGCAAGGGCTCGG

TGATGAAGCTCGGCAAGAACGACCGCTCCATGGACATCGAGGCGGTGTCCTCCGGCTCGC

TCGGGCTCGATATCGCGCTCGGCATCGGCGGCCTGCCCAAGGGACGCATCGTCGAGATCT

ACGGGCCGGAATCCTCGGGCAAGACCACGCTGGCGCTGCATACGGTGGCGGAAGCGCAGA

AGAAGGGCGGCATCTGCGCCTTCATCGACGCCGAGCACGCGCTCGATCCGGTCTATGCCC

GCAAGCTCGGCGTCAATATCGACGAGCTCCTGATCTCGCAGCCCGACACCGGCGAGCAGG

CCCTGGAGATCTGCGACACCCTGGTGCGCTCGGGCGCGGTCGATGTTCTCGTGGTCGATT

CGGTGGCGGCGCTGGTGCCGAAGGCCGAGCTCGAGGGCGAGATGGGCGATGCGCTGCCGG

GCCTGCAGGCCCGTCTGATGAGCCAGGCGCTGCGCAAGCTGACGGCCTCCATCAACAAGT

CCAACACCATGGTGATCTTCATCA

>Seq24 [organism=Bradyrhizobium sp. TUTVUMm115] [strain=TUTVUMm115] *recA* gene for recombinase A, partial cds,

AGCTCTGGCTGCCGCGCTCTCTCAGATCGAGCGCCAGTTCGGCAAGGGCTCGGTGATGAA

GCTCGGCAAGAACGACCGCTCCATGGACATCGAGGCGGTGTCCTCCGGCTCGCTCGGGCT

CGATATCGCGCTCGGCATCGGCGGCCTGCCCAAGGGGCGCATCGTCGAGATCTACGGGCC

GGAATCGTCCGGCAAGACCACGCTGGCGCTGCATACGGTGGCGGAAGCGCAGAAGAAGGG

TGGCATCTGCGCCTTCATCGATGCCGAGCACGCGCTCGATCCGGTCTATGCCCGCAAGCT

CGGCGTCAACATCGACGAGCTCCTGATCTCGCAGCCCGACACCGGCGAGCAGGCGCTGGA

GATCTGCGACACCCTGGTGCGCTCGGGCGCGGTCGATGTTCTCGTGGTCGATTCGGTGGC

GGCGCTGGTGCCGAAGGCCGAGCTCGAGGGCGAGATGGGCGATGCGCTGCCGGGACTTCA

AGCACGGTTGATGAGCCAGGCGCTGCGCAAGCTCACGGCCTCCATCAACAAATCCAACAC

CATGGTGATCTTCA

>Seq25 [organism=Bradyrhizobium sp. TUTVUMm116] [strain=TUTVUMm116] *recA* gene for recombinase A, partial cds,

AGCTCTGGCTGCCGCGCTCTCTCAGATCGAGCGCCAGTTCGGCAAGGGCTCGGTGATGAA

GCTCGGCAAGAACGACCGCTCCATGGACATCGAGGCGGTGTCCTCCGGCTCGCTCGGGCT

CGATATCGCGCTCGGCATCGGCGGCCTGCCCAAGGGGCGCATCGTCGAGATCTACGGGCC

GGAATCGTCCGGCAAGACCACGCTGGCGCTGCATACGGTGGCGGAAGCGCAGAAGAAGGG

TGGCATCTGCGCCTTCATCGATGCCGAGCACGCGCTCGATCCGGTCTATGCCCGCAAGCT

CGGCGTCAACATCGACGAGCTCCTGATCTCGCAGCCCGACACCGGCGAGCAGGCGCTGGA

GATCTGCGACACCCTGGTGCGCTCGGGCGCGGTCGATGTTCTCGTGGTCGATTCGGTGGC

GGCGCTGGTGCCGAAGGCCGAGCTCGAGGGCGAGATGGGCGATGCGCTGCCGGGACTTCA

AGCACGGTTGATGAGCCAGGCGCTGCGCAAGCTCACGGCCTCCATCAACAAATCCAACAC

CATGGTGATCTTCATCACCC

>Seq26 [organism=Bradyrhizobium sp.TUTVUI122] [strain=TUTVUI122] *recA* gene for recombinase A, partial cds,

AAGAGTAAAGCCCTGGCTGCCGCGCTCTCCCAGATCGAGCGCCAGTTCGGCAAGGGATCG

GTGATGAAGCTCGGCAAGAGCGACCGCTCCATGGACATCGAGGCGGTGTCTTCCGGCTCG

CTCGGGCTCGATATCGCGCTCGGCATCGGCGGTCTGCCGAAGGGGCGCATCGTCGAGATC

TACGGGCCGGAATCCTCGGGCAAGACCACGCTGGCGCTGCACACGGTGGCGGAAGCACAG

AAGAAGGGCGGCATCTGCGCCTTCATCGACGCCGAGCACGCGCTCGACCCGGTCTATGCC

CGCAAGCTGGGCGTCAACATCGACGAGCTCCTGATCTCGCAGCCCGACACCGGCGAGCAG

GCGCTGGAAATTTGCGACACGCTGGTGCGTTCGGGCGCGGTGGACGTGCTGGTGGTCGAT

TCGGTTGCGGCGCTGGTGCCGAAGGCCGAGCTCGAAGGCGAGATGGGCGATGCGCTGCCG

GGCCTGCAGGCACGTCTGATGAGCCAGGCGCTGCGCAAGCTGACGGCCTCCATCAACAAG

TCCAACAC

>Seq27 [organism=Bradyrhizobium sp. TUTVUMr95] [strain=TUTVUMr95] recA gene for recombinase A, partial cds,

TCCATGGACAAGAGTAAAGCTCTGGCTGCCGCGCTCTCCCAGATCGAGCGTCAGTTCGG

CAAGGGCTCGGTGATGAAGCTCGGCAAGAACGACCGTTCGATGGACATCGAGGCAGTGT

CGTCAGGCTCGCTCGGGCTCGATATCGCGCTCGGCATCGGCGGCCTGCCCAAGGGGCGC

ATCGTCGAGATCTACGGGCCGGAATCCTCAGGAAAAACCACGCTGGCGCTGCACACGGT

GGCGGAAGCCCAGAAGAAGGGCGGCATCTGCGCCTTCATCGACGCCGAGCACGCGCTCG

ACCCGGTCTATGCCCGCAAGCTCGGGGTCAACATCGACGAGCTCTTGATCTCCCAGCCC

GACACCGGCGAGCAGGCGCTGGAAATCTGCGACACGTTGGTGCGCTCGGGCGCGGTGGA

CGTGCTGGTGGTCGATTCGGTCGCGGCCCTGGTGCCGAAGGCCGAGCTCGAAGGCGAGA

TGGGTGATGCGCTGCCGGGTCTTCAAGCGCGGTTGATGAGCCAGGCGCTGCGCAAGCTG

ACGGCCTCGATCAACAAGTCCAATACCATGGTGATCTTC

>Seq28 [organism=Bradyrhizobium sp. TUTVUMr80] [strain=TUTVUMr80] recA gene for recombinase A, partial cds,

CTGGCTGCCGCGCTCTCCCAGATCGAGCGTCAGTTCGGCAAGGGCTCGGTGATGAAGCTC

GGCAAGAACGACCGTTCGATGGACATCGAGGCAGTGTCGTCAGGCTCGCTCGGGCTCGAT

ATCGCGCTCGGCATCGGCGGCCTGCCCAAGGGGCGCATCGTCGAGATCTACGGGCCGGAA

TCCTCAGGAAAAACCACGCTGGCGCTGCACACGGTGGCGGAAGCCCAGAAGAAGGGCGGC

ATCTGCGCCTTCATCGACGCCGAGCACGCGCTCGACCCGGTCTATGCCCGCAAGCTCGGG

GTCAACATCGACGAGCTCTTGATCTCCCAGCCCGACACCGGGGAGCAGGCGCTGGAAATC

TGCGACACGTTGGTGCGCTCGGGCGCGGTGGACGTGCTGGTGGTCGATTCGGTCGCGGCC

CTGGTGCCGAAGGCCGAGCTCGAAGGCGAGATGGGTGATGCGCTGCCGGGTCTTCAAGCG

CGGCTGATGAGCCAGGCGCTGCGCAAGCTGACGGCCTCGATCAACAAGTCCAATACCATG

GTGATCTTCATCAACC

***nifH***

>Seq1 [organism=Bradyrhizobium sp. TUTVUMp5] [strain=TUTVUMp5] *nifH* gene for nitrogenase reductase, partial cds,

GGCGCTGGCCGAGATGGGTCAGAAAATCCTGATTGTAGGGTGCGATCCGAAAGCGGACTC

GACCCGCCTGATTCTGCACGCCAAGGCGCAAGACACGATTTTGAGCCTTGCAGCGAGCGC

CGGCAGCGTGGAAGACCTCGAGCTCGAGGACGTAATGAAGGTCGGCTACAAGGACATTCG

CTGCGTGGAGTCTGGTGGTCCTGAGCCGGGTGTCGGCTGCGCCGGCCGCGGCGTCATCAC

CTCGATCAATTTCCTGGAGGAGAACGGCGCCTACGAGAACATTGACTATGTCTCATACGA

CGTGCTCGGCGACGTCGTTTGCGGTGGCTTTGCGATGCCAATCCGCGAGAACAAGGCGCA

GGAAATCTATATCGTGATGTCCGGTGAAATGATGGCGATGTATGCCGCAAACAACATTTC

CAAGGGCATCCTGAAATACGCGAACTCTGGTGGCGTGCGGCTGGGCGGTCTGATCTGCAA

CGAGCGGCAGACCGACAAGGAGCTGGAACTGGCGGAAGCGTTGGCCAAGAAGCTAGGCAC

TCAGCTGATCTACTTCGTGCCGCGCGACAACGTGGTGCAGCATGCCGAGTTACGCCGCAT

GACGGTGCTCGAGTATGCACCCGATTCCAAGCAGGCCGATCACTATCGCAATCTTGCGAC

CAAGGTTCACAACAATGGCGGCAAAGGCATCATCCCGACCCCGATCTCCATGGCGATGGA

>Seq2 [organism=Bradyrhizobium sp. TUTVUMp8] [strain=TUTVUMp8] *nifH* gene for nitrogenase reductase, partial cds,

GGCGCTGGCCGAGATGGGTCAGAAAATCCTGATTGTAGGGTGCGATCCGAAAGCGGACT

CGACCCGCCTGATTCTGCACGCCAAGGCGCAAGACACGATTTTGAGCCTTGCAGCGAGCGCC

GGCAGCGTGGAAGACCTCGAGCTCGAGGACGTAATGAAGGTCGGCTACAAGGACATTCGCTGC

GTGGAGTCTGGTGGTCCTGAGCCGGGTGTCGGCTGCGCCGGCCGCGGCGTCATCACCTCGATC

AATTTCCTGGAGGAGAACGGCGCCTATGAGAACATTGACTATGTCTCATACGACGTGCTCGGC

GACGTCGTTTGCGGTGGCTTTGCGATGCCAATCCGCGAGAACAAGGCGCAGGAAATCTATATC

GTGATGTCCGGTGAAATGATGGCGATGTATGCCGCAAACAACATTTCCAAGGGCATCCTGAAA

TACGCGAACTCTGGTGGCGTGCGGCTGGGCGGTCTGATCTGCAACGAGCGGCAGACCGACAAG

GAGCTGGAACTGGCGGAAGCGTTGGCCAAGAAGCTAGGTACTCAGCTGATCTACTTCGTGCCG

CGCGACAACGTGGTGCAGCATGCCGAGTTACGCCGCATGACCGTGCTCGAATATGCACCCGAT

TCCAAGCAGGCCGATCACTATCGCAATCTTGCGACCAAGGTTCACAATAATGGCGGCAAAGGC

ATCATCCCGACCCCGATCTCCATGGA

>Seq3 [organism=Bradyrhizobium sp. TUTVUMp48] [strain=TUTVUMp48] *nifH* gene for nitrogenase reductase, partial cds,

CCTGATTGTAGGGTGCGATCCGAAAGCGGACTCGACCCGCCTGATTCTGCACGCCAAGGC

GCAAGACACGATTTTGAGCCTTGCAGCGAGCGCCGGCAGCGTGGAAGACCTCGAGCTCG

AGGACGTAATGAAGGTCGGCTACAAGGACATTCGCTGCGTGGAGTCTGGTGGTCCTGAG

CCGGGTGTCGGCTGCGCCGGCCGCGGCGTCATCACCTCGATTAATTTCCTGGAGGAGAA

CGGCGCCTACGAGAACATTGACTATGTCTCATACGACGTGCTCGGCGACGTCGTTTGCG

GTGGCTTTGCGATGCCAATCCGCGAGAACAAGGCGCAGGAAATCTATATCGTGATGTCC

GGTGAAATGATGGCGATGTATGCCGCAAACAACATTTCCAAGGGCATCCTGAAATACGC

GAACTCTGGTGGCGTGCGGCTGGGCGGTCTGATCTGCAACGAGCGGCAGACAGACCAGG

AGCTGGAACTGGCGGAAGCGTTGGCCAAGAAGCTAGGCACTCAGCTGATCTACTTCGTGC

CGCGCGACAACGTGGTGCAGCATGCCGAGTTACGCCGCATGACGGTGCTCGAGTATG

CACCCGAGTCCAAGCAGGCCGATCACTATCGCAATCTTGCGACCAAGGTTCACAACAAT

GGCGGCAAAGGCATCATCCCGACCCCGATCTCCATGGACGAG

>Seq4 [organism=Bradyrhizobium sp. TUTVUMp50] [strain=TUTVUMp50] *nifH* gene for nitrogenase reductase, partial cds,

ACGCTGGCGGCGCTGGCCGAGATGGGTCAGAAAATCCTGATTGTGGGGTGCGATCCGAAA

GCGGACTCGACCCGCTTGATTCTGCACGCCAAGGCGCAAGACACGATTTTGAGTCTTGCA

GCGAGCGCCGGCAGCGTGGAGGACCTCGAGCTCGAGGACGTAATGAAGGTCGGCTACAAG

GACATTCGCTGCGTGGAGTCTGGTGGTCCTGAGCCGGGTGTCGGCTGCGCCGGCCGCGGC

GTCATCACCTCGATCAATTTCCTGGAGGAGAACGGCGCCTACGAGAACATTGACTATGTC

TCATACGACGTGCTCGGCGACGTCGTTTGCGGTGGCTTTGCGATGCCAATCCGCGAGAAC

AAGGCGCAGGAAATCTATATCGTGATGTCCGGTGAAATGATGGCGATGTATGCCGCAAAC

AACATTTCCAAGGGCATCCTGAAATACGCGAACTCTGGTGGCGTGCGGCTGGGCGGTCTGA

TCTGCAACGAGCGGCAGACCGACAAGGAGCTGGAACTGGCGGAAGCGTTGGCCAAGAAGCT

AGGCACTCAGCTGATCTACTTCGTGCCGCGCGACAACGTGGTGCAGCATGCCGAGTTACGC

CGCATGACGGTGCTCGAGTATGCACCCGAGTCCAAGCAGGCCGATCACTATCGCAATCTTG

CGACCAAGGTTCACAA

>Seq5 [organism=Bradyrhizobium sp. TUTVUMp53] [strain=TUTVUMp53] *nifH* gene for nitrogenase reductase, partial cds,

CACTTCGCAGAACACGCTGGCGGCGCTGGCCGAGATGGGTCAGAAAATCCTGATTGTGGGG

TGCGATCCGAAAGCGGACTCGACCCGCTTGATTCTGCACGCCAAGGCGCAAGACACGATT

TTGAGTCTTGCAGCGAGCGCCGGCAGCGTGGAGGACCTCGAGCTCGAGGACGTAATGAAG

GTCGGCTACAAGGACATTCGCTGCGTGGAGTCTGGTGGTCCTGAGCCGGGTGTCGGCTGC

GCCGGCCGCGGCGTCATCACCTCGATCAATTTCCTGGAGGAGAACGGCGCCTACGAGAAC

ATTGACTATGTCTCATACGACGTGCTCGGCGACGTCGTTTGCGGTGGCTTTGCGATGCCA

ATCCGCGAGAACAAGGCGCAGGAAATCTATATCGTGATGTCCGGTGAAATGATGGCGATG

TATGCCGCAAACAACATTTCCAAGGGCATCCTGAAATACGCGAACTCTGGTGGCGTGCGG

CTGGGCGGTCTGATCTGCAACGAGCGGCAGACCGACAAGGAGCTGGAACTGGCGGAAGCG

TTGGCCAAGAAGCTAGGCACTCAGCTGATCTACTTCGTGCCGCGCGACAACGTGGTGCAG

CATGCCGAGTTACGCCGCATGACGGTGCTCGAGTATGCACCCGAGTCCAAGCAGGCCGAT

CACTATCGCAATCTTGCGACCAAGGTTCACAACAATGGCGGCAAAGGCATCATCCCGACC

CCGATCTCCATGGACGAAC

>Seq6 [organism=Bradyrhizobium sp. TUTVUMp56] [strain=TUTVUMp56] *nifH* gene for nitrogenase reductase, partial cds,

GGCGCTGGCCGAGATGGGTCAGAAAATCCTGATTGTAGGGTGCGATCCGAAAGCGGACTC

GACCCGGTTGATTCTGCATGCCAAGGCGCAAGACACGATTTTGAGCCTTGCAGCGAGCGC

CGGCAGCGTGGAGGACCTCGAGCTCGAGGACGTAATGAAGGTCGGCTACAAGGACATTCG

CTGCGTGGAGTCGGGTGGTCCTGAGCCGGGTGTCGGCTGCGCCGGCCGCGGCGTCATCAC

CTCGATCAATTTCCTGGAGGAGAACGGCGCCTACGAGAACATTGACTATGTCTCATACGA

CGTGCTCGGCGACGTCGTTTGCGGTGGCTTTGCGATGCCAATCCGCGAAAACAAGGCGCA

GGAAATCTATATCGTGATGTCCGGTGAAATGATGGCGATGTATGCCGCAAACAACATTTC

CAAGGGCATCCTGAAATACGCGAACTCTGGTGGCGTGCGGCTGGGCGGTCTGATCTGCAA

CGAGCGGCAGACCGACAAGGAGCTGGAACTGGCGGAAGCGTTGGCCAAGAAGCTAGGCAC

TCAGCTGATCTACTTCGTGCCGCGCGACAACGTGGTGCAGCATGCCGAGTTACGCCGCAT

GACGGTGCTCGAGTATGCACCCGATTCCAAGCAGGCCGATCACTATCGCAATCTTGCGAC

CAAGGTTCACAACAATGGCGGCAAAGGCATCATCCCGACCCCGATCTCCATGGAGATGGA

>Seq7 [organism=Bradyrhizobium sp. TUTVUMp60] [strain=TUTVUMp60] *nifH* gene for nitrogenase reductase, partial cds,

ACGTTGGCGGCGCTGGCCGAGATGGGTCAGAAAATCCTGATTGTCGGGTGCGATCCTAAA

GCGGACTCGACCCGCTTGATCCTGCATGCCAAAGCGCAGGACACGATTCTGAGCTTGGCG

GCGAGTGCCGGCAGCGTGGAGGATCTTGAACTCGAGGACGTGATGAAGGTCGGCTACAAA

GACATCCGCTGCGTGGAGTCCGGTGGTCCTGAGCCGGGCGTCGGCTGCGCCGGTCGTGGC

GTCATCACTTCGATCAACTTCCTCGAGGAGAACGGCGCCTATGAGAACATTGACTACGTA

TCCTATGATGTGCTCGGCGACGTCGTTTGCGGTGGCTTTGCGATGCCAATCCGTGAAAAC

AAGGCGCAGGAGATCTATATCGTGATGTCCGGTGAAATGATGGCTATGTATGCCGCCAAC

AATATTTCCAAGGGTATCCTGAAATACGCCAACTCGGGCGGTGTGCGGCTGGGCGGCCTG

GTCTGCAATGAACGACAGACGGACAAGGAGTTGGAACTGGCGGAGGCGCTGGCCAAGAAG

CTAGGCACTCAGCTGATCTACTTCGTGCCGCGCGACAACGTGGTGCAGCATGCCGAACTC

CGCCGCATGACGGTGCTCGAGTATGCGCCGGATTCGAAGCAGGCCGATCACTATCGCAAT

CTCGCTACCAAGGTTCACAACAATGGCGGCAAGGGTATCA

>Seq8 [organism=Bradyrhizobium sp. TUTVUMp65] [strain=TUTVUMp65] *nifH* gene for nitrogenase reductase, partial cds,

GGCGCTGGCCGAGATGGGTCAGAAAATCCTGATTGTAGGGTGCGATCCGAAAGCGGACTC

GACCCGCCTGATTCTGCACGCCAAGGCGCAAGACACGATTTTGAGCCTTGCAGCGAGCGC

CGGCAGCGTGGAAGACCTCGAGCTCGAGGACGTAATGAAGGTCGGCTACAAGGACATTCG

CTGCGTGGAGTCTGGTGGTCCTGAGCCGGGTGTCGGCTGCGCCGGCCGCGGCGTCATCAC

CTCGATCAATTTCCTGGAGGAGAACGGCGCCTACGAGAACATTGACTATGTCTCATACGA

CGTGCTCGGCGACGTCGTTTGCGGTGGCTTTGCGATGCCAATCCGCGAGAACAAGGCGCA

GGAAATCTATATCGTGATGTCCGGTGAAATGATGGCGATGTATGCCGCAAACAACATTTC

CAAGGGCATCCTGAAATACGCGAACTCTGGTGGCGTGCGGCTGGGCGGTCTGATCTGCAA

CGAGCGGCAGACCGACAAGGAGCTGGAACTGGCGGAAGCGTTGGCCAAGAAGCTAGGCAC

TCAGCTGATCTACTTCGTGCCGCGCGACAACGTGGTGCAGCATGCCGAGTTACGCCGCAT

GACGGTGCTCGAGTATGCACCCGATTCCAAGCAGGCCGATCACTATCGCAATCTTGCGAC

CAAGGTTCACAACAATGGCGGCAAAGGCATCATCCCGACCCCGATCTCCATGGAGATGGA

>Seq9 [organism=Bradyrhizobium sp. TUTVUMp75] [strain=TUTVUMp75] *nifH* gene for nitrogenase reductase, partial cds,

ACGTTGGCGGCGCTGGCCGAGATGGGTCAGAAAATCCTGATTGTCGGGTGCGATCCTAAA

GCGGACTCGACCCGCTTGATCCTGCATGCCAAAGCGCAGGACACGATTCTGAGCTTGGCG

GCGAGTGCCGGCAGCGTGGAGGATCTTGAACTCGAGGACGTGATGAAGGTCGGCTACAAA

GACATCCGCTGCGTGGAGTCCGGTGGTCCTGAGCCGGGCGTCGGCTGCGCCGGTCGTGGC

GTCATCACTTCGATCAACTTCCTCGAGGAGAACGGCGCCTATGAGAACATTGACTACGTA

TCCTATGATGTGCTCGGCGACGTCGTTTGCGGTGGCTTCGCGATGCCAATCCGTGAAAAC

AAGGCGCAGGAGATCTATATCGTGATGTCCGGTGAAATGATGGCTATGTATGCCGCCAAC

AATATTTCCAAGGGTATCCTGAAATACGCCAACTCGGGCGGTGTGCGGCTGGGCGGCCTG

GTCTGCAATGAACGACAGACGGACAAGGAGTTGGAACTGGCGGAGGCGCTGGCCAAGAAG

CTAGGCACTCAGCTGATCTACTTCGTGCCGCGCGACAACGTGGTGCAGCATGCCGAACTC

CGCCGCATGACGGTGCTCGAGTATGCGCCGGATTCGAAGCAGGCCGATCACTATCGCAAT

CTCGCTACCAAGGTTCACAACAATGGCGGCAAGGGTATCA

>Seq10 [organism=Bradyrhizobium sp. TUTVUMr80] [strain=TUTVUMr80] *nifH* gene for nitrogenase reductase, partial cds,

CGCTGGCGGCGCTGGCTGAGATGGGTCAGAAAATTCTCATTGTGGGGTGCGATCCGAAGG

CGGACTCAACCCGGTTGATCTTGCACGCCAAGGCGCAAGATACGATTTTGAGCCTTGCAG

CGAACGCCGGAAGCGTGGAGGACCTAGAACTCGAAGACGTAATGAAAGTCGGCTATAGGG

ACATTCGCTGTGTGGAGTCCGGCGGTCCTGAGCCAGGTGTCGGCTGTGCGGGTCGCGGTG

TCATCACCTCAATCAATTTTCTGGAAGAGAACGGCGCCTACGAGAACATTGACTATGTCT

CATACGACGTGCTTGGCGACGTCGTTTGCGGTGGCTTTGCGATGCCGATTCGCGAGAACA

AGGCGCAGGAAATCTACATCGTGATGTCCGGTGAAATGATGGCGATGTATGCTGCAAACA

ACATTTCCAAGGGCATCCTGAAGTACGCGAACTCCGGTGGGGTGCGGCTGGGCGGTCTGG

TCTGCAATGAGCGGCAGACCGACAAGGAATTGGAACTGGCGGAAGCGCTGGCTAAGAAGC

TGGGCACTCAGTTGATCTACTTCGTGCCGCGCGACAACGTGGTGCAACATGCAGAGCTAC

GCCGGATGACGGTGCTTGAATATGCTCCCGACTCTAAACAGGCAGAGCACTATCGGAAGC

TCGCTCAAAAGGTTCACAACAATGGCGGTAAAGGCATCATCCCGACCCCG

ATCTCTATGG

>Seq11 [organism=Bradyrhizobium sp. TUTVUMr82] [strain=TUTVUMr82] *nifH* gene for nitrogenase reductase, partial cds,

GCACTGGCCGAGATGGGCCAGAAAATCCTAATTGTGGGATGCGATCCTAAGGCGGATTCG

ACCCGTCTGATCCTGCATGCCAAGGCGCAAGACACGATCTTGAGCCTTGCGGCGAGTGCC

GGCAGCGTGGAGGATCTCGAACTCGAGGATGTGATGAAGGTCGGCTACAAGGACATCCGC

TGCGTGGAGTCCGGTGGTCCTGAGCCCGGTGTCGGCTGTGCCGGTCGCGGCGTCATCACC

TCGATCAATTTCCTGGAGGAGAACGGCGCTTACGAGAACATCGACTATGTCTCCTACGAC

GTGCTCGGTGACGTCGTTTGCGGAGGCTTCGCGATGCCAATTCGCGAGAACAAGGCGCAG

GAGATCTATATCGTCATGTCGGGTGAAATGATGGCGATGTATGCCGCAAACAACATCTCC

AAGGGTATCCTGAAATACGCCAACTCGGGCGGCGTGCGGCTGGGTGGACTTGTCTGTAAC

GAGCGGCAGACCGACAAGGAGCTGGAATTGGCGGAGGCGCTGGCGAAGAAGTTGGGCACT

CAGCTGATCTACTTCGTGCCGCGCGACAATGTAGTGCAGCATGCGGAACTGCGGCGCATG

ACGGTGCTGGAGTATGCGCCCGATTCGCAACAGGCCGATCACTATCGCAACCTTGCCACC

AAGGTTCACAACAATGGCGGCAAGGGCATCATTCCGACCC

>Seq12 [organism=Bradyrhizobium sp. TUTVUMr84] [strain=TUTVUMr84] *nifH* gene for nitrogenase reductase, partial cds,

ACGCTGGCGGCGCTGGCTGAGATGGGTCAGAAAATCCTGATTGTAGGGTGCGACCCGAAG

GCGGACTCGACCCGCCTGATTCTGCACGCCAAGGCGCAAGACACGATTTTGAGCCTTGCAG

CGAGCGCCGGCAGCGTGGAGGACCTCGAGCTCGAGGACGTAATGAAGGTCGGCTACAAGGA

CATTCGCTGCGTGGAGTCCGGTGGTCCTGAGCCGGGTGTCGGCTGCGCCGGCCGCGGCGTC

ATTACTTCGATCAATTTCCTGGAGGAGAACGGCGCCTACGAAAACATTGACTATGTCTCAT

ACGACGTGCTCGGCGACGTCGTTTGCGGGGGCTTTGCGATGCCGATCCGCGAGAACAAGGC

GCAGGAAATCTATATCGTGATGTCCGGTGAAATGATGGCGATGTATGCCGCGAACAACATT

TCCAAGGGGATCCTGAAATACGCAAACTCTGGCGGCGTGCGGCTGGGCGGTCTGATCTGCA

ATGAGCGGCAGACCGACAAGGAGCTGGAACTGGCGGAAGCGTTGGCCAAGAAGCTAGGCAC

TCAGCTGATCTACTTCGTGCCGCGCGACAACGTGGTGCAGCATGCCGAGTTACGTCGCATG

ACGGTGCTCGAATATGCGCCCGATTCCAAGCAGGCCGATCACTATCGCAATCTTGCGAACA

CGGTTCATAAAAATGGCGGCAAAGGCATCATCCCGACTCCAATCTCCATGGA

>Seq13 [organism=Bradyrhizobium sp. TUTVUMr85] [strain=TUTVUMr85] *nifH* gene for nitrogenase reductase, partial cds,

CGCTGGCGGCGCTGGCTGAGATGGGTCAGAAAATTCTCATTGTGGGGTGCGATCCAAAGG

CGGACTCAACCCGGTTGATCTTGCACGCCAAGGCGCAAGATACGATTTTGAGCCTTGCAG

CGAGCGCCGGAAGCGTGGAGGACCTAGAACTCGAAGACGTAATGAAAGTCGGCTATAGGG

ACATTCGCTGTGTGGAGTCCGGCGGTCCTGAGCCAGGTGTCGGCTGTGCGGGTCGCGGTG

TCATCACCTCAATCAATTTTCTGGAAGAGAACGGCGCCTACGAGAACATTGACTATGTCT

CATACGACGTGCTTGGCGACGTCGTTTGCGGTGGCTTTGCGATGCCGATTCGCGAGAACA

AGGCGCAGGAAATCTACATCGTGATGTCCGGTGAAATGATGGCGATGTATGCTGCAAACA

ACATTTCCAAGGGCATCCTGAAGTACGCGAACTCCGGTGGGGTGCGGCTGGGCGGTCTGG

TCTGCAATGAGCGGCAGACCGACAAGGAATTGGAACTGGCGGAAGCGTTGGCTAAGAAGC

TGGGCACTCAGTTGATCTACTTCGTGCCGCGCGACAACGTGGTGCAACATGCAGAGCTAC

GCCGGATGACGGTGCTTGAATATGCTCCCGACTCTAAACAGGCAGAGCACTATCGGAAGC

TCGCTCAAAAGGTTCACAACAATGGCGGTAAAGGCATCATCCCGACCCCG

>Seq14 [organism=Bradyrhizobium sp. TUTVUMr89] [strain=TUTVUMr89] *nifH* gene for nitrogenase reductase, partial cds,

GGCGCTGGCCGAGATGGGTCAGAAAATCCTGATTGTAGGGTGCGATCCGAAAGCGGACTC

GACCCGCCTGATTCTGCACGCCAAGGCGCAAGACACGATTTTGAGCCTTGCAGCGAGCGC

CGGCAGCGTGGAAGACCTCGAGCTCGAGGACGTAATGAAGGTCGGCTACAAGGACATTCG

CTGCGTGGAGTCTGGTGGTCCTGAGCCGGGTGTCGGCTGCGCCGGCCGCGGCGTCATCAC

CTCGATCAATTTCCTGGAGGAGAACGGCGCCTATGAGAACATTGACTATGTCTCATACGA

CGTGCTCGGCGACGTCGTTTGCGGTGGCTTTGCGATGCCAATCCGCGAGAACAAGGCGCA

GGAAATCTATATCGTGATGTCCGGTGAAATGATGGCGATGTATGCCGCAAATAACATTTC

CAAGGGTATCCTGAAATACGCGAACTCTGGCGGCGTGCGGCTGGGCGGTCTGATCTGCAA

CGAGCGGCAGACCGACAAGGAGCTGGAACTGGCGGAAGCGTTGGCCAAGAAGCTAGGCAC

TCAGCTGATCTACTTCGTACCGCGCGACAACGTGGTGCAGCATGCCGAGTTACGCCGCAT

GACGGTGCTCGAGTATGCACCCGATTCCAAGCAGGCCGATCACTATCGCAATCTTGCGAC

CAAGGTTCACAATAATGGCGGCAAGGGCATCATCCCGACCCCGATCTCCATGGA

>Seq15 [organism=Bradyrhizobium sp. TUTVUMr93] [strain=TUTVUMr93] *nifH* gene for nitrogenase reductase, partial cds,

ACGCTGGCGGCGCTGGCTGAGATGGGTCAGAAAATTCTGATTGTGGGGTGTGATCCGAA

GGCGGACTCGACCCGGCTGATCTTGCACGCCAAGGCGCAAGATACGATTTTGAGCCTTGC

AGCGAGCGCCGGCAGCGTGGAGGACCTAGAACTCGAAGACGTCATGAAGGTCGGCTACAG

GGACATTCGCTGCGTGGAGTCCGGCGGTCCCGAGCCAGGTGTCGGCTGTGCGGGTCGCGG

TGTCATCACCTCAATCAATTTTCTGGAAGAAAACGGCGCCTACGAGAACATTGACTATGT

CTCATACGACGTGCTTGGCGACGTCGTTTGCGGTGGCTTTGCCATGCCGATCCGCGAGAA

CAAGGCGCAGGAAATCTACATCGTGATGTCCGGTGAAATGATGGCTATGTATGCTGCAAA

CAACATTTCCAAGGGCATCCTGAAGTATGCGAACTCCGGCGGGGTGCGGCTGGGAGGTCT

GGTCTGCAATGAGCGGCAGACCGACAAGGAATTGGAACTAGCGGAAGCGTTGGCTAAGAA

GCTGGGCACTCAGTTGATCTACTTCGTGCCGCGCGACAACGTGGTGCAACATGCAGAGCT

ACGCCGTATGACGGTGCTTGAATATGCTCCCGACTCTAAACAGGCAGAGCACTATCGGAA

GCTCGCTCAAAAGGTTCACAACAATGGCGGTAAAGGCATCATCCCGACCCCG

>Seq16 [organism=Bradyrhizobium sp. TUTVUMr95] [strain=TUTVUMr95] *nifH* gene for nitrogenase reductase, partial cds,

CGCTGGCGGCGCTGGCTGAGATGGGTCAGAAAATTCTCATTGTGGGGTGCGATCCAAAGG

CGGACTCAACCCGGTTGATCTTGCACGCCAAGGCGCAAGATACGATTTTGAGCCTTGCAG

CGAACGCCGGAAGCGTGGAGGACCTAGAACTCGAAGACGTAATGAAAGTCGGCTATAGGG

ACATTCGCTGTGTGGAGTCCGGCGGTCCTGAGCCAGGTGTCGGCTGTGCGGGTCGCGGTG

TCATCACCTCAATCAATTTTCTGGAAGAGAACGGCGCCTACGAGAACATTGACTATGTCT

CATACGACGTGCTTGGCGACGTCGTTTGCGGTGGCTTTGCGATGCCGATTCGCGAGAACA

AGGCGCAAGAAATCTACATCGTGATGTCCGGTGAAATGATGGCGATGTATGCTGCAAACA

ACATTTCCAAGGGCATCCTGAAGTACGCGAACTCCGGTGGGGTGCGGCTGGGCGGTCTGG

TCTGCAATGAGCGGCAGACCGACAAGGAATTGGAACTGGCGGAAGCGTTGGCTAAGAAGC

TGGGCACTCAGTTGATCTACTTCGTGCCGCGCGACAACGTGGTGCAACATGCAGAGCTAC

GCCGGATGACGGTGCTTGAATATGCTCCCGACTCTAAACAGGCAGAGCACTATCGGAAGC

TCGCTCAAAAGGTTCACAACAATGGCGGTAAAGGCATCATCCCGACCCCG

>Seq17 [organism=Bradyrhizobium sp. TUTVUMr99] [strain=TUTVUMr99] *nifH* gene for nitrogenase reductase, partial cds,

GCACTGGCCGAGATGGGCCAGAAAATCCTAATTGTGGGATGCGATCCTAAGGCGGATTC

GACCCGTCTGATCCTGCATGCCAAGGCGCAAGACACGATCTTGAGCCTTGCGGCGAGTGC

CGGCAGCGTGGAGGATCTCGAACTCGAGGATGTGATGAAGGTGGGCTACAAGGACATCCG

CTGCGTGGAGTCCGGTGGTCCTGAGCCCGGTGTCGGCTGTGCCGGTCGCGGCGTCATCAC

CTCGATTAATTTCCTGGAGGAGAACGGCGCTTACGAGAACATCGACTATGTCTCCTACGA

CGTGCTCGGTGACGTCGTTTGCGGAGGCTTCGCGATGCCAATTCGCGAGAACAAGGCGCA

GGAGATCTATATCGTCATGTCGGGTGAAATGATGGCGATGTATGCCGCAAACAACATCTC

CAAGGGTATCCTGAAATACGCCAACTCGGGCGGCGTGCGGCTGGGTGGACTTGTCTGTAA

CGAGCGGCAGACCGACAAGGAGCTGGAATTGGCGGAGGCGCTGGCGAAGAAGTTGGGCAC

TCAGCTGATCTACTTCGTGCCGCGCGACAATGTAGTGCAGCATGCCGAGCTACGGCGCAT

GACGGTGCTGGAGTATGCGCCCGATTCGCAGCAGGCCGATCATTATCGCAACCTGGCGA

CCAAGGTTCACAACAATGGCGGCAAAGGCATCATTCCGACC

>Seq18 [organism=Bradyrhizobium sp. TUTVUMm108] [strain=TUTVUMm108] *nifH* gene for nitrogenase reductase, partial cds,

GAGATGGGTCAGAAAATCCTGATTGTGGGGTGCGACCCGAAAGCGGACTCGACCCGCCTG

ATTTTGCACGCCAAGGCGCAAGACACGATTTTGAGCCTTGCCGCGAGTGCCGGCAGCGTG

GAGGACCTCGAGCTCGAGGACGTAATGAAGGTCGGCTACAAGGACATTCGCTGCGTGGAG

TCTGGTGGTCCTGAGCCGGGTGTCGGCTGCGCCGGCCGCGGCGTCATTACCTCGATCAAT

TTCCTGGAGGAGAACGGCGCCTACGAAAACATTGACTATGTCTCATACGACGTGCTCGGC

GACGTCGTTTGCGGTGGCTTTGCGATGCCGATCCGCGAGAACAAGGCGCAGGAAATCTAT

ATTGTGATGTCCGGTGAAATGATGGCTATGTATGCCGCGAACAATATTTCCAAGGGAATC

CTGAAATACGCAAACTCTGGCGGTGTGCGGCTGGGCGGTCTGATCTGCAATGAGCGGCAG

ACCGACAAGGAGCTGGAACTGGCGGAAGCGTTGGCCAAGAAGCTAGGCACTCAGCTGATC

TACTTCGTGCCGCGCGACAACGTGGTGCAGCATGCCGAGTTACGCCGCATGACGGTGCTC

GAGTATGCACCCGATTCCAAGCAGGCCGATCACTATCGCAATCTTGCGATCAAGGTTCAC

AAAAATGGCGGCAAAGGCATCATCCCGACTCCAATCTCCATGGAAGAAC

>Seq19 [organism=Bradyrhizobium sp. TUTVUMm110] [strain=TUTVUMm110] *nifH* gene for nitrogenase reductase, partial cds,

GAGATGGGTCAGAAAATCCTGATTGTGGGGTGCGACCCGAAAGCGGACTCGACCCGCCTG

ATTTTGCACGCCAAGGCGCAAGACACGATTTTGAGCCTTGCCGCGAGTGCCGGCAGCGTG

GAGGACCTCGAGCTCGAGGACGTAATGAAGGTCGGCTACAAGGACATTCGCTGCGTGGAG

TCTGGTGGTCCTGAGCCGGGTGTCGGCTGCGCCGGCCGCGGCGTCATTACCTCGATCAAT

TTCCTGGAGGAGAACGGCGCCTACGAAAACATTGACTATGTCTCATACGACGTGCTCGGC

GACGTCGTTTGCGGTGGCTTTGCGATGCCGATCCGCGAGAACAAGGCGCAGGAAATCTAT

ATTGTGATGTCCGGTGAAATGATGGCTATGTATGCCGCGAACAATATTTCCAAGGGAATC

CTGAAATACGCAAACTCTGGCGGTGTGCGGCTGGGCGGTCTGATCTGCAATGAGCGGCAG

ACCGACAAGGAGCTGGAACTGGCGGAAGCGTTGGCCAAGAAGCTAGGCACTCAGCTGATC

TACTTCGTGCCGCGCGACAACGTGGTGCAGCATGCCGAGTTACGCCGCATGACGGTGCTC

GAGTATGCACCCGATTCCAAGCAGGCCGATCACTATCGCAATCTTGCGACCAAGGTTCAC

AAAAATGGCGGCAAAGGCATCATCCCGACTCCAATCTCCATGGA

>Seq20 [organism=Bradyrhizobium sp. TUTMm112] [strain=TUTMm112] *nifH* gene for nitrogenase reductase, partial cds,

ACGCTAGCGGCGCTAGCCGAGCTGGGTCAGAAAATCCTGATTGTGGGGTGTGATCC

GAAGGCGGACTCGACCCGCCTAATTCTGCACGCCAAGGCGCAAGACACAATTTTGA

GCTTGGCTGCGAGCGCCGGCAGCGTGGAGGACCTAGAGCTCGAGGACGTCATGAAGGT

CGGCTACAAGGACATTCGCTGCGTGGAGTCCGGTGGTCCTGAACCAGGTGTCGGCTGC

GCCGGCCGCGGTGTCATCACCTCGATTAATTTTCTGGAAGAGAACGGCGCCTACGAGA

ACATTGACTACGTCTCATACGACGTGCTTGGCGACGTTGTTTGCGGTGGCTTTGCGAT

GCCAATCCGCGAGAACAAGGCGCAGGAAATCTACATTGTGATGTCCGGTGAAATGATG

GCGATGTATGCCGCGAACAACATCTCCAAGGGCATCCTGAAATACGCAAACTCCGGCG

GCGTGCGGCTGGGCGGGCTGATCTGCAACGAGCGGCAGACCGACAAGGAGCTCGAATT

GGCGGAAGCGTTGGCCAAGAAGCTAGGCACTCAGCTGATCTACTTCGTGCCGCGCGAC

AACGTGGTGCAGCATGCGGAATTACGGCGCATGACGGTACTTGAATATGCCCCCGATT

CCAAGCAGGCGGATCACTATCGCAATCTTGCGACCAAGGTTCACAATAATGGCGGCAA

AGGCATCATCCCGACCCCGATTTCCATGGA

>Seq21 [organism=Bradyrhizobium sp. TUTVUMm113] [strain=TUTVUMm113] *nifH* gene for nitrogenase reductase, partial cds,

CACTTCGCAGAACACGCTAGCGGCGCTAGCCGAGCTGGGTCAGAAAATCCTGATTGTGGG

GTGTGATCCGAAGGCGGACTCGACCCGCCTAATTCTGCACGCCAAGGCGCAAGACACAAT

TTTGAGCTTGGCTGCGAGCGCCGGCAGCGTGGAGGACCTAGAGCTCGAGGACGTCATGAA

GGTCGGCTACAAGGACATTCGCTGCGTGGAGTCCGGTGGTCCTGAACCAGGTGTCGGCTG

CGCCGGCCGCGGTGTCATCACCTCGATTAATTTTCTGGAAGAGAACGGCGCCTACGAGAAC

ATTGACTACGTCTCATACGACGTGCTTGGCGACGTTGTTTGCGGTGGCTTTGCGATGCCAA

TCCGCGAGAACAAGGCGCAGGAAATCTACATTGTGATGTCCGGTGAAATGATGGCGATGTA

TGCCGCGAACAACATCTCCAAGGGCATCCTGAAATACGCAAACTCCGGCGGCGTGCGGCTG

GGCGGGCTGATCTGCAACGAGCGGCAGACCGACAAGGAGCTCGAATTGGCGGAAGCGTTGG

CCAAGAAGCTAGGCACTCAGCTGATCTACTTCGTGCCGCGCGACAACGTGGTGCAGCATGC

GGAATTACGGCGCATGACGGTACTTGAATATGCCCCCGATTCCAAGCAGGCGGATCACTAT

CGCAATCTTGCGACCAAGGTTCACAATAATGGCGGCAAAGGCATCATCCCGACCCCGATCT

CCATGGA

>Seq22 [organism=Bradyrhizobium sp. TUTVUMm114] [strain=TUTVUMm114] *nifH* gene for nitrogenase reductase, partial cds,

ACGCTAGCGGCGCTAGCCGAGCTGGGTCAGAAAATCCTGATTGTGGGGTGTGATCCGAAG

GCGGACTCGACCCGCCTAATTCTGCACGCCAAGGCGCAAGACACAATTTTGAGCTTGGCT

GCGAGCGCCGGCAGCGTGGAGGACCTAGAGCTCGAGGACGTCATGAAGGTCGGCTACAAG

GACATTCGCTGCGTGGAGTCCGGTGGTCCTGAACCAGGTGTCGGCTGCGCCGGCCGCGGT

GTCATCACCTCGATTAATTTTCTGGAAGAGAACGGCGCCTACGAGAACATTGACTACGTC

TCATACGACGTGCTTGGCGACGTTGTTTGCGGTGGCTTTGCGATGCCAATCCGCGAGAAC

AAGGCGCAGGAAATCTACATTGTGATGTCCGGTGAAATGATGGCGATGTATGCCGCGAAC

AACATCTCCAAGGGCATCCTGAAATACGCAAACTCCGGCGGCGTGCGGCTGGGCGGGCTG

ATCTGCAACGAGCGGCAGACCGACAAGGAGCTCGAATTGGCGGAAGCGTTGGCCAAGAAG

CTAGGCACTCAGCTGATCTACTTCGTGCCGCGCGACAACGTGGTGCAGCATGCGGAATTA

CGGCGCATGACGGTACTTGAATATGCCCCCGATTCCAAGCAGGCGGATCACTATCGCAAT

CTTGCGACCAAGGTTCACAATAATGGCGGCAAAGGCATCATCCCGACCCCGATCTCCATG

GA

>Seq23 [organism=Bradyrhizobium sp. TUTVUMm116] [strain=TUTVUMm116] *nifH* gene for nitrogenase reductase, partial cds,

CACTTCGCAGAACACGCTAGCGGCGCTAGCCGAGCTGGGCCAGAAAATCCTGATTGT

GGGGTGTGATCCGAAGGCGGACTCGACCCGCCTAATTCTGCACGCCAAGGCGCAAGA

CACAATTTTGAGCTTGGCTGCGAGCGCCGGCAGCGTGGAGGACCTAGAGCTCGAGGACGT

CATGAAGGTCGGCTACAAGGACATTCGCTGCGTGGAGTCCGGTGGTCCTGAACCAGGTGT

CGGCTGCGCCGGCCGCGGTGTCATCACCTCGATTAATTTTCTGGAAGAGAACGGCGCCTA

CGAGAACATTGACTACGTCTCATACGACGTGCTTGGCGACGTTGTTTGCGGTGGCTTTGC

GATGCCAATCCGCGAGAACAAGGCGCAGGAAATCTACATTGTGATGTCCGGTGAAATGAT

GGCGATGTATGCCGCGAACAACATCTCCAAGGGCATCCTGAAATACGCAAACTCCGGCGG

CGTGCGGCTGGGCGGGCTGATCTGCAACGAGCGGCAGACCGACAAGGAGCTCGAATTGGC

GGAAGCGTTGGCCAAGAAGCTAGGCACTCAGCTGATCTACTTCGTGCCGCGCGACAACGT

GGTGCAGCATGCGGAATTACGGCGCATGACGGTACTTGAATATGCCCCCGATTCCAAGCA

GGCGGATCACTATCGCAATCTTGCGACCAAGGTTCACAATAATGGCGGCAAAGGCATCAT

CCCGACCCCGATCTCCATGGA

>Seq24 [organism=Bradyrhizobium sp. TUTVUMm117] [strain=TUTVUMm117] *nifH* gene for nitrogenase reductase, partial cds,

AAATCCTGATTGTGGGATGCGATCCTAAGGCGGATTCGACCCGCCTGATCCTGCATGCCA

AGGCGCAGGACACGATCTTGAGTCTTGCAGCGAGCGCCGGCAGCGTGGAGGATCTCGAAC

TCGAGGATGTGATGAAGGTCGGCTACAAGGACATCCGCTGCGTGGAGTCCGGTGGCCCTG

AGCCGGGTGTCGGCTGCGCCGGCCGCGGTGTCATCACCTCGATCAATTTCCTGGAGGAGA

ACGGCGCCTACGAGAACATTGACTATGTCTCCTACGATGTGCTCGGCGACGTCGTTTGCG

GGGGCTTCGCGATGCCAATCCGCGAGAACAAGGCGCAGGAAATCTATATCGTCATGTCGG

GTGAAATGATGGCGATGTATGCCGCAAACAACATCTCCAAGGGCATCCTGAAATACGCCA

ACTCGGGTGGCGTGCGGCTGGGTGGCCTGGTCTGTAATGAGCGGCAGACCGATAAGGAGC

TGGAGCTGGCGGAGGCGCTGGCGAAGAAGTTAGGCACTCAGCTGATCTACTTCGTGCCGC

GCGACAATGTGGTGCAGCATGCCGAGCTGCGGCGCATGACGGTGCTCGAGTATGCGCCCG

AGTCGCAGCAGGCCGATCACTATCGCAATCTTGCAACCAAAATCCACAACAATGGCGGCA

AGGGCATCATTCCCACCCCGATCTCTATGGACGAACTCGAGGAACATGCT

>Seq25 [organism=Bradyrhizobium sp. TUTVUI122] [strain=TUTVUI122] *nifH* gene for nitrogenase reductase, partial cds,

GAGATGGGTCAGAAAATCCTGATTGTGGGGTGCGACCCGAAAGCGGACTCGACCCGCCTG

ATTTTGCACGCCAAGGCGCAAGACACGATTTTGAGCCTTGCCGCGAGTGCCGGCAGCGTG

GAGGACCTCGAGCTCGAGGACGTAATGAAGGTCGGCTACAAGGACATTCGCTGCGTGGAG

TCTGGTGGTCCTGAGCCGGGTGTCGGCTGCGCCGGCCGCGGCGTCATTACCTCGATCAAT

TTCCTGGAGGAGAACGGCGCCTACGAAAACATTGACTATGTCTCATACGACGTGCTCGGC

GACGTCGTTTGCGGTGGCTTTGCGATGCCGATCCGCGAGAACAAGGCGCAGGAAATCTAT

ATTGTGATGTCCGGTGAAATGATGGCTATGTATGCCGCGAACAATATTTCCAAGGGAATC

CTGAAATACGCAAACTCTGGCGGTGTGCGGCTGGGCGGTCTGATCTGCAATGAGCGGCAG

ACCGACAAGGAGCTGGAACTGGCGGAAGCGTTGGCCAAGAAGCTAGGCACTCAGCTGATC

TACTTCGTGCCGCGCGACAACGTGGTGCAGCATGCCGAGTTACGCCGCATGACGGTGCTC

GAGTATGCACCCGATTCCAAGCAGGCCGATCACTATCGCAATCTTGCGACCAAGGTTCAC

AAAAATGGCGGCAAAGGCATCATCCCGACTCCCATCTCCATGGA

***nodC***

>Seq1 [organism=Bradyrhizobium sp. TUTVUMp5] [strain=TUTVUMp5] *nodC* gene for N-acetylglucosaminyltransferase, partial cds,

CGACGGATCTGCAAATCGCGGCGTTGTCGCGCCTGTACACAAGATCTATGCGAACGATC

CGAGATTCAGCATCATCCTGCTGGCGAACAACGTTGGAAAGCGCAAGGCGCAGATCGCT

GCAATACGCAGCTCATCCGGAGACCTGGTCCTCAACGTCGATTCGGATACGATACTTGC

TGCTGACGTTGTCACGAAGCTTGTAGTGAAGATGCATGACCCGGAAATCGGGGCGGCCA

TGGGTCAGTTGATAGCGAGCAATC

>Seq2 [organism=Bradyrhizobium sp. TUTVUMp8] [strain=TUTVUMp8] *nodC* gene for N-acetylglucosaminyltransferase, partial cds,

CGACGACGGATCTGCAAATCGCGGCGTTGTCGCGCCTGTACACAAGATCTATGCAAACG

ATCCGAGATTCAGCATCATCTTGCTGGCAAACAACGTTGGGAAGCGCAAGGCGCAGATC

GCTGCAATACGCAGCTCATCCGGGGACCTGGTGCTCAACGTGGACTCGGATACGATACTT

GCTGCTGACGTTGTCACGAAGCTTGTAGTGAAGATGCATGACCCAGAAATCGGTGCGGCC

ATGGGTCAGTTGATAGCGAGCAATCGCAGCGAAACCTGGC

>Seq3 [organism=Bradyrhizobium sp. TUTVUMp48] [strain=TUTVUMp48] *nodC* gene for N-acetylglucosaminyltransferase, partial cds,

CGACGACGGATCTGCAAATCGCGGCGTTGTCGCGCCTGTACACAAGATCTATGCAA

ACGATCCGAGATTCAGCATCATCTTGCTGGCAAACAACGTTGGGAAGCGCAAGGCGC

AGATCGCTGCAATACGCAGCTCATCCGGGGACCTGGTGCTCAACGTGGACTCGGATAC

GATACTTGCTGCTGACGTTGTCACGAAGCTTGTAGTGAAGATGCATGACCCAGAAATC

GGTGCGGCCATGGGTCAGTTGATAGCGAGCAATCGCAGCGAAACCTGGC

>Seq4 [organism=Bradyrhizobium sp. TUTVUMp50] [strain=TUTVUMp50] *nodC* gene for N-acetylglucosaminyltransferase, partial cds,

CGACGACGGATCTGCAAATCGCGGCGTTGTCGCGCCTGTACACAAGATCTATGCAA

ACGATCCGAGATTCAGCATCATCTTGCTGGCAAACAACGTTGGGAAGCGCAAGGCGC

AGATCGCTGCAATACGCAGCTCATCCGGGGACCTGGTGCTCAACGTGGACTCGGATAC

GATACTTGCTGCTGACGTTGTCACGAAGCTTGTAGTGAAGATGCATGACCCAGAAATC

GGTGCGGCCATGGGTCAGTTGATAGCGAGCAATCGCAGCGAAACCTGGC

>Seq5 [organism=Bradyrhizobium sp. TUTVUMp53] [strain=TUTVUMp53] *nodC* gene for N-acetylglucosaminyltransferase, partial cds,

CGACGACGGATCTGCAAATCGCGGCGTTGTCGCGCCTGTACACAAGATCTATGCAA

ACGATCCGAGATTCAGCATCATCTTGCTGGCAAACAACGTTGGGAAGCGCAAGGCGCAGA

TCGCTGCAATACGCAGCTCATCCGGGGACCTGGTGCTCAACGTGGACTCGGATACGATAC

TTGCTGCTGACGTTGTCACGAAGCTTGTAGTGAAGATGCATGACCCAGAAATCGGTGCGG

CCATGGGTCAGTTGATAGCGAGCAATCGCAGCGAAACCTGGC

>Seq6 [organism=Bradyrhizobium sp. TUTVUMp56] [strain=TUTVUMp56] *nodC* gene for N-acetylglucosaminyltransferase, partial cds,

CAGGTCTATGTGGTCGACGACGGATCTGCAAATCGCGGCGTTGTCGCGCCTGTA

CACAAGATCTATGCAAACGATCCGAGATTCAGCATCATCTTGCTGGCAAACAACGTTGGG

AAGCGCAAGGCGCAGATCGCTGCAATACGCAGCTCATCCGGGGACCTGGTCCTCAACGTC

GACTCGGATACGATACTTGCTGCTGACGTTGTCACGAAGCTTGTAGTGAAGATGCATGAC

CCGGAAATCGGTGCGGCCATGGGTCAGTTGATAGCGAGCAATCGCA

>Seq7 [organism=Bradyrhizobium sp. TUTVUMp60] [strain=TUTVUMp60] *nodC* gene for N-acetylglucosaminyltransferase, partial cds,

CGACGATGGCTCGGCAAATCGCGACGTTGTGGGGCCTGTACATAAGATCTATGCCAAC

GATGCGCGCTTCAGCATCATCTTGCTTGCCAGGAATGTTGGCAAGCGCAAAGCGCAGATC

GCCGCAATACGTGGCTCGTCCGGTGACTTGGTGCTTAACGTCGATTCGGATACGATACTG

GCCGCCGACGTGGTCACCAAGCTAGCGGCGAAGATGCGGGACCCCGACATTGGTGCCGCG

ATGGGCCAGCTGGTAGCGAGCAATCGCAACGATACCTGGC

>Seq8 [organism=Bradyrhizobium sp. TUTVUMp65] [strain=TUTVUMp65] *nodC* gene for N-acetylglucosaminyltransferase, partial cds,

CGACGACGGCTCTGCAAATCGCGGCGTTGTCGCGCCTGTACACAAGATCTATGCAAACG

ATCCGAGATTCAGCATCATCTTACTGGCAAACAACGTTGGGAAGCGCAAGGCGCAGAT

CGCTGCAATACGCAGCTCATCCGGGGACCTGGTGCTCAACGTGGACTCGGATACGATA

CTTGCTGCTGACGTTGTCACGAAGCTTGTAGTGAAGATGCATGACCCAGAAATCGGTG

CGGCCATGGGTCAGTTGATAGCGAGCAATCGCAGCGAAACCTGGC

>Seq9 [organism=Bradyrhizobium sp. TUTVUMr89] [strain=TUTVUMp89] *nodC* gene for N-acetylglucosaminyltransferase, partial cds,

GGTCGACGACGGATCTGCCAATCGCGGCGTTGTCGCGCCTGTACACAAAAT

CTATGCGAACGATCCGAGATTCAGCATCATCTTGCTGGCAAACAACGTTGGAAA

GCGCAAGGCGCAGATCGCTGCAATACGCAGCTCATCCGGGGACCTGGTGCTCAACGTCGA

CTCGGATACGATACTTGCTGCTGACGTTGTCACGAAGCTTGTAGTGAAGATGCATGACCC

AGAAATCGGGGCGGCCATGGGTCAGTTGATAGCGAGCAATCGCA

>Seq10 [organism=Bradyrhizobium sp. TUTVUMr93] [strain=TUTVUMp93] *nodC* gene for N-acetylglucosaminyltransferase, partial cds,

GCAAACCGCGACGTTGTCGCGCCTGTACACCGGATATATGCGAGCGATCCAAGATTCAG

TTTCATCTTGTTGGCAAACAATGTTGGAAAGCGCAAAGCGCAAATCGCCGCGATACGTAG

CTCATCCGGTGATCTGGTTCTCAACGTCGACTCCGATACCATACTTGCTGCTGACGTCGT

CACAAAGCTCGTACTGAAGATGCATGACCCGGGAATTGGCGCGGCGATGGGTCAGCTGAT

AGCGAGCAATCGCAACCAGACCTGGC

>Seq11 [organism=Bradyrhizobium sp. TUTVUMr94] [strain=TUTVUMp94] *nodC* gene for N-acetylglucosaminyltransferase, partial cds,

GGTCGATGACGGATCTGCAAACCGCGACGTCGTCGCGCCTGTACACCAGATATATGCCAA

TGATCCGAGATTTAGTATCATCTTGCTGCCAAACAACGTGGGAAAGCGCAAGGCGCAGA

TCGCTGCAATACGCAGCTCGTCCGGCGACCTGGTCCTCAACGTCGACTCAGATACGATA

CTTGCTGCTGACGTCGTCTCGAAGCTTGTTTTGAAGATGCATGACCCGGAAATCGGTGC

GGCCATGGGTCAGCTGATAGCGAGCAATCGCA

>Seq12 [organism=Bradyrhizobium sp. TUTVUMr95] [strain=TUTVUMp95] *nodC* gene for N-acetylglucosaminyltransferase, partial cds,

AACCGCGACGTTGTCGCGCCTGTACACCGGATATATGCGAGCGATCCAAGATTCAGTTT

CATCTTGCTCGCAAACAATGTTGGAAAGCGCAAAGCGCAAATCGCCGCGATACGTAGCTC

ATCCGGTGATCTGGTTCTCAACGTCGACTCCGATACCATACTTGCTGCTGACGTCGTCAC

AAAGCTCGTACTGAAGATGCATGATCCGGGAATTGGCGCGGCGATGGGTCAGCTGATAGC

GAGCAATCGCAAC

>Seq13 [organism=Bradyrhizobium sp. TUTVUMr103] [strain=TUTVUMp103] *nodC* gene for N-acetylglucosaminyltransferase, partial cds,

GATGACGGATCTGCAAACCGCGACGTCGTCGCGCCTGTACACCAGATATATGCCAATGAT

CCGAGATTTAGTATCATCTTGCTGCCAAACAACGTGGGAAAGCGCAAGGCGCAGATCGCT

GCAATACGCAGCTCGTCCGGCGACCTGGTCCTCAACGTCGACTCAGATACGATACTTGCT

GCTGACGTCGTCTCGAAGCTTGTATTGAAGATGCATGACCCGGAAATCGGTGCGGCCATG

GGTCAGCTGATAGCGAGCAATCGCA

>Seq14 [organism=Bradyrhizobium sp. TUTVUMm105] [strain=TUTVUMp105] *nodC* gene for N-acetylglucosaminyltransferase, partial cds,

CGATGACGGATCTGCAAACCGCGACGTCGTCGCGCCTGTACACCAGATATATGCCAATG

ATCCGAGATTTAGTATCATCTTGCTGCCAAACAACGTGGGAAAGCGCAAGGCGCAGATC

GCTGCAATACGCAGCTCGTCCGGCGACCTGGTCCTCAACGTCGACTCAGATACGATACT

TGCTGCTGACGTCGTCTCGAAGCTTGTTTTGAAGATGCATGACCCGGAAATCGGTGCGG

CCATGGGTCAGCTGATAGCGAGCAATCGCA

>Seq15 [organism=Bradyrhizobium sp. TUTVUMm112] [strain=TUTVUMp112] *nodC* gene for N-acetylglucosaminyltransferase, partial cds,

GGTCTATGTGGTCGATGACGGATCTGCAAACCGCGACGTCGTCGAGCCTGTACACCA

GATTTATGCCAATGATCCGAGATTTAGTATCATCTTGCTGCCAAACAACGTGGGAA

AGCGCAAGGCGCAGATCGCTGCAATACGCAGCTCGTCCGGCGACCTGGTCCTCAACGT

CGACTCAGATACGATACTCGCTGCTGACGTCGTCTCGAAGCTTGTATTGAAGATGCA

TGACCCGGAAATCGGTGCGGCCATGGGTCAGCTGATAGCGAGCAATCG

>Seq16 [organism=Bradyrhizobium sp. TUTVUMm113] [strain=TUTVUMp113] *nodC* gene for N-acetylglucosaminyltransferase, partial cds,

GGTCGATGACGGATCTGCAAACCGCGACGTCGTCGCGCCTGTACACCAGATATATG

CCAATGATCCAAGATTTAGTATCATCTTGCTGCCAAACAACGTGGGAAAGCGCAAGGCG

CAGATCGCTGCAATACGCAGCTCGTCCGGCGACCTGGTCCTCAACGTCGACTCAGATAC

GATACTTGCTGCTGACGTCGTCTCGAAGCTTGTATTGAAGATGCATGACCCGGAAATCG

GTGCGGCCATGGGTCAGCTGATAGCGAGCAATCGC

>Seq17 [organism=Bradyrhizobium sp. TUTVUMm115] [strain=TUTVUMp115] *nodC* gene for N-acetylglucosaminyltransferase, partial cds,

CTATGTGGTCGATGACGGATCTGCAAACCGCGACGTCGTCGCGCCTGTACACCAGATA

TATGCCAATGATCCGAGATTTAGTATCATCTTGCTGCCAAACAACGTGGGAAAGCGCAA

GGCGCAGATCGCTGCAATACGCAGCTCGTCCGGCGACCTGGTCCTCAACGTCGACTCAG

ATACGATACTTGCTGCTGACGTCGTCTCGAAGCTTGTATTGAAGATGCATGACCCGGAA

ATCGGTGCGGCCATGGGTCAGCTGATAGCGAGCAATCGCA

>Seq18 [organism=Bradyrhizobium sp. TUTVUMm116] [strain=TUTVUMp116] *nodC* gene for N-acetylglucosaminyltransferase, partial cds,

GTCTATGTGGTCGATGACGGATCTGCAAACCGCGACGTCGTCGCGCCTGTACACCAGATA

TATGCCAATGATCCGAGATTTAGTATCATCTTGCTGCCAAACAACGTGGGAAAGCGCAA

GGCGCAGATCGCTGCAATACGCAGCTCGTCCGGCGACCTGGTCCTCAACGTCGACTCAG

ATACGATACTTGCTGCTGACGTCGTCTCGAAGCTTGTATTGAAGATGCGTGACCCGGAA

ATCGGTGCGGCCATGGGTCAGCTGATAGCGAGCAATCG

>Seq19 [organism=Bradyrhizobium sp. TUTVUI122] [strain=TUTVUI122] *nodC* gene for N-acetylglucosaminyltransferase, partial cds,

CGTTGTCGCGCCTGTACACAAAATCTATGCGAACGATCCAAGGTTCAGCGTCATCTTGC

TGGCAAACAACGTTGGAAAGCGCAAGGCGCAGATCGCCGCAATACGCAGCTCATCCGGTG

ACTTGGTCCTCAATGTCGACTCGGATACGATACTTGCCGCTGACGTTGTCACGAAGCTTG

TATTGAAGATGCACGACCCGGAAGTCGGTGCGGCCATGGGTCAGTTGATAGCGAGCAATC

GCA

>Seq20 [organism=Bradyrhizobium sp. TUTVUMp75] [strain=TUTVUMp75] nodC gene for N-acetylglucosaminyltransferase, partial cds,

GGTCTATGTGGTCGACGATGGCTCGGCAAATCGCGACGTTGTGGGGCCTGTACATAAG

ATTTATGCCAACGATGCGCGCTTCAGCATCATCTTGCTTGCCAGGAATGTTGGCAAGCG

CAAAGCGCAGATCGCCGCAATACGCGGCTCGTCCGGTGACTTGGTGCTTAACGTCGATT

CGGATACGATACTGGCCGCCGACGTGGTCACCAAGCTTGCGGCGAAGATGCGGGACCCC

GACATTGGTGCCGCGATGGGCCAGTTGGTAGCGAGC

>Seq21 [organism=Bradyrhizobium sp. TUTVUMp6] [strain=TUTVUMp6] nodC gene for N-acetylglucosaminyltransferase, partial cds,

CAGGTCTATGTGGTAGATGACGGATCTGCAAACCGCGACGTCGTCGCGCCTGTACACCAG

ATATATGCCAATGATCCGAGATTTAGTATCATCTTGCTGCCAAACAACGTGGGAAAGCGC

AAGGCGCAGATCGCTGCAATACGCAGCTCGTCCGGCGACCTGGTCCTCAACGTCGACTCA

GATACGATACTTGCTGCTGACGTCGTCTCGAAGCTTGTATTGAAGATGCATGACCCGGAA

ATCGGTGCGGCCATGGGTCAGCTGATAGCGAGCAATCGCA

>Seq22 [organism=Bradyrhizobium sp. TUTVUMm114] [strain=TUTVUMm114] nodC gene for N-acetylglucosaminyltransferase, partial cds,

CGATGACGGATCTGCAAACCGCGACGTCGTCGCGCCTGTACACCAGATATATGCCAATG

ATCCGAGATTTAGTATCATCTTGCTGCCAAACAACGTGGGAAAGCGCAAGGCGCAGATCG

CTGCAATACGCAGCTCGTCCGGCGACCTGGTCCTCAACGTCGACTCAGATACGATACTTG

CTGCTGACGTCGTCTCGAAGCTTGTATTGAAGATGCATGACCCGGAAATCGGTGCGGCCA

TGGGTCAGCTGATAGCGAGCAATCGCA

**Fig. S2**: Nucleotide sequences of test genes used in phylogenetic trees construction

**Fig. S3:** Correlation between IAA concentration and phosphate solubilisation by bacteria


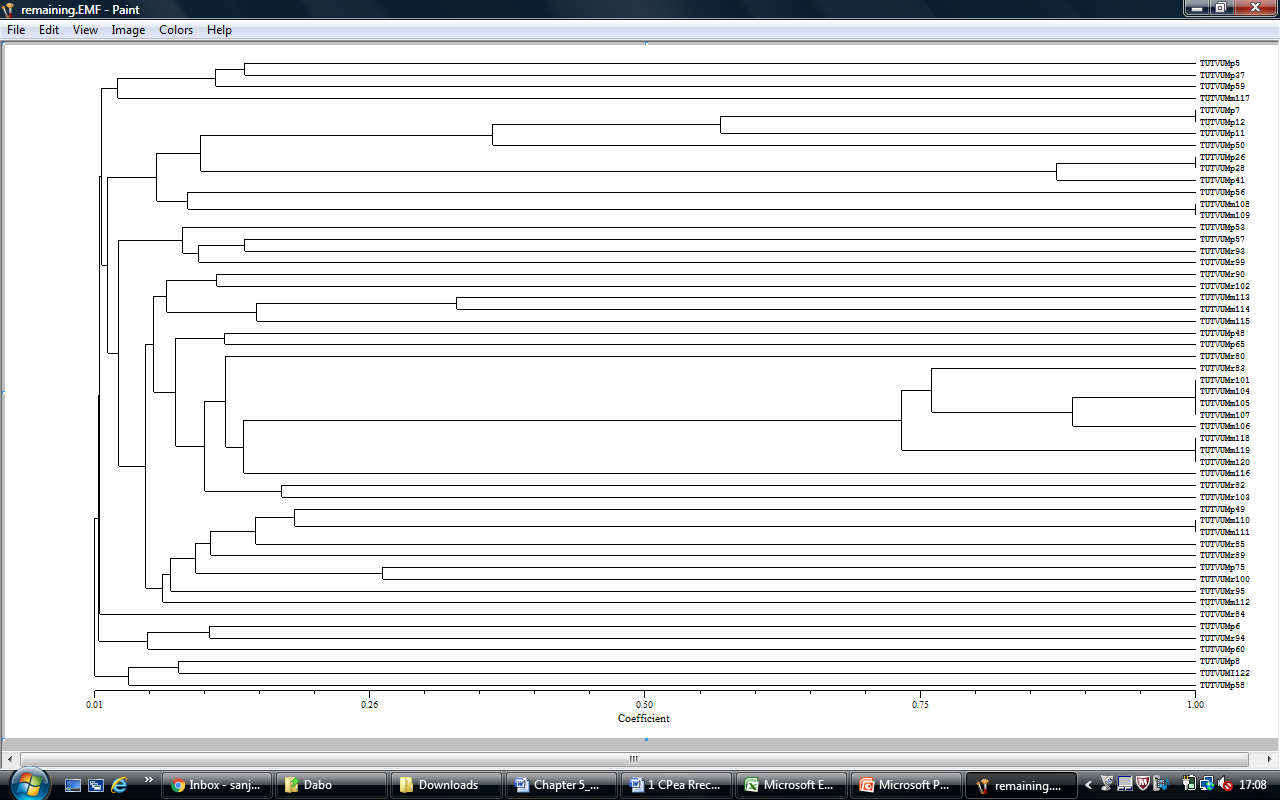


VIII

VII

VI

V

IV

III

II

I

**Fig. S4:** Dendrogram generated from Box-PCR banding pattern of 54 rhizobial isolates using Jaccard’s similarity coefficients (UPGMA) method

V

IV

I b

I a

II b

II a

I

III

*Bradyrhizobium elkanii* USDA 76^T^ (AY386758)

*Bradyrhizobium* *pachyrhizi* PAC48^T^ (FJ428208)

*Bradyrhizobium* *tropiciagri* SEMIA 6148^T^ (FJ390968)

*Bradyrhizobium* *embrapense* SEMIA 6208^T^ (HQ634875)

*Bradyrhizobium* *macuxiense* BR 10303^T^ (LNCU01000024.1)

**TUTVUMr82** (MK288788)

**TUTVUMr99** (MK288795)

*Bradyrhizobium* *mercantei* SEMIA 6399^T^ (NZ MKFI01000006)

**TUTVUMm117** (MK288805)

**TUTVUMp60** (MK288784)

**TUTVUMp75** (MK288786)

*Bradyrhizobium* *brasilense* UFLA03-321^T^ (KF452730.1)

*Bradyrhizobium* *japonicum* LMG 6138^T^ (AM418753.1)

*Bradyrhizobium* *ingae* BR 10250^T^ (KY753593.1)

*Bradyrhizobium* *iriomotense*^T^ (AB300994.1)

**TUTVUMr80** (MK288787)

**TUTVUMr93** (MK288792)

**TUTVUMr85** (MK288790)

**TUTVUMr95** (MK288794)

*Bradyrhizobium* *forestalis* INPA54B^T^ (KF452722.1)

*Bradyrhizobium* *subterraneum* 58 2-1^T^ (KX661391.1)

**TUTVUMp6** (MK288778)

*Bradyrhizobium* *yuanmingense* CCBAU 10071^T^ (AY386760)

**TUTVUMr84** (MK288789)

**TUTVUMr94** (MK88793)

**TUTVUMm105** (MK288797)

**TUTVUMr103** (MK288796)

**TUTVUMm110** (MK288799)

**TUTVUMm112** (MK288800)

**TUTVUMm113** (MK288801)

**TUTVUMm114** (MK288802)

**TUTVUMm115** (MK288803)

**TUTVUMm116** (MK288804)

*Bradyrhizobium* *kavangense* 14-3^T^ (KY753592.1)

*Bradyrhizobium* *centrosematis* A9^T^ (KC247129.1)

*Bradyrhizobium* *denitrificans* LMG 8443^T^ (FM253153.1)

*Bradyrhizobium* *liaoningense* bv. *glycinearum* LMG 18230^T^ (AY386752)

**TUTVUMm108** (MK288798)

**TUTVUI122** (MK288806)

*Bradyrhizobium* *arachidis* CCBAU 45332^T^ (JQ011347.1)

*Bradyrhizobium* *huanghuaihaiense* CCBAU 23303^T^ (HQ231682)

*Bradyrhizobium* *sacchari* BR10280^T^ (KX065107.1)

*Bradyrhizobium* *guangxiense* CCBAU 53363^T^ (KC508926)

*Bradyrhizobium* *rifense* CTAW71^T^ (GU001617)

*Bradyrhizobium* *diazoefficiens* USDA 110^T^ (CP011360.1)

*Bradyrhizobium* *guangdongense* CCBAU 51649^T^ (KC508916)

*Bradyrhizobium* *ganzhouense* RITF807^T^ (JX277183)

*Bradyrhizobium* *americanum* CMVU44^T^ (KC247125.1)

*Bradyrhizobium* *daqingense* CCBAU 15774^T^ (HQ231289)

*Bradyrhizobium* *betae* LMG 21987^T^ (FM253129.1)

*Bradyrhizobium* *ottawaense* OO99^T^ (HQ455212)

*Bradyrhizobium* *shewense* ERR11^T^ (NZ FMAI01000019.1)

*Bradyrhizobium* *canariense* LMG 22265^T^ (FM253135)

*Bradyrhizobium* *lupini* USDA 3051^T^ (KU738808.1)

*Bradyrhizobium* *cytisi* CTAW11^T^ (GU001613)

**TUTVUMp56** (MK288783)

**TUTVUMp8** (MK288779)

**TUTVUMp48** (MK288780)

**TUTVUMp5** (MK288777)

**TUTVUMr89** (MK288791)

**TUTVUMp65** (MK288785)

**TUTVUMp50** (MK288781)

**TUTVUMp53** (MK288782)

*Bradyrhizobium* *jicamae* PAC68^T^ (FJ428211)

*Bradyrhizobium* *namibiense* 5-10^T^ (KX661387.1)

*Bradyrhizobium* *paxllaeri* LMTR 21^T^ (KF896186)

*Bradyrhizobium* *lablabi* CCBAU 23086^T^ (GU433473)

*Bradyrhizobium* *algeriense* RST89^T^ (KF956544.1)

*Bradyrhizobium* *valentinum* LmjM3^T^ (JX518561)

*Bradyrhizobium* *icense* LMTR 13^T^ (KF896192)

*Bradyrhizobium* *retamae* Ro19^T^ (KC247101)

*Bradyrhizobium* *oligotrophicum* LMG 10732^T^ (JQ619232)

*Rhizobium* *lusitanum* p1-7^T^ (DQ431671)

98

91

77

93

97

94

61

97

97

99

86

96

99

72

78

90

85

88

91

97

81

56

87

81

72

56

63

57

51

0.02

II

**Fig. S5:** Maximum-likelihood molecular phylogenetic analysis of *atpD* nucleotide sequences of cowpea nodulating rhizobia collected from South Africa and Mozambique. The associated taxa clustered together in the 1000 bootstrap support and percentage are shown next to the branches. The evolutionary distances were computed using the Kimura 2-parameter method and are in the units of the number of base substitutions per site. The analysis involved 73 nucleotide sequences. Codon positions included were 1st+2nd+3rd+Noncoding. All positions containing gaps and missing data were eliminated. Evolutionary analyses were conducted in MEGA7.

*Bradyrhizobium* *canariense* bv. genistearum BTA-1^T^ (AY386765.1)

*Bradyrhizobium* *lupini* USDA 3051^T^ (KM114862)

*Bradyrhizobium* *ingae* BR 10250^T^ (KF927067)

*Bradyrhizobium* *iriomotense*^T^ (AB300995.1)

*Bradyrhizobium* *ganzhouense* RITF807^T^ (JX277111)

*Bradyrhizobium* *cytisi* CTAW11^T^ (GU001594)

*Bradyrhizobium* *rifense* CTAW71^T^ (GU001604)

*Bradyrhizobium* *huanghuaihaiense* CCBAU 23303^T^ (HQ231639)

*Bradyrhizobium* *stylosanthis* BR 446^T^ (KU724148.1)

*Bradyrhizobium* *japonicum* LMG 6138^T^ (AF169582)

*Bradyrhizobium* *betae* LMG 21987^T^ (AB353733.1)

*Bradyrhizobium* *diazoefficiens* USDA 110^T^ (CP011360.1)

*Bradyrhizobium* *liaoningense* bv. glycinearum LMG 18230^T^ (AY386775)

*Bradyrhizobium* *ottawaense* OO99^T^ (HQ587750)

*Bradyrhizobium* *shewense* ERR11^T^ (JQ809893.1)

*Bradyrhizobium* *cajani* AMBPC1010^T^ (KY349442.1)

*Bradyrhizobium* *americanum* CMVU44^T^ (KX012942)

*Bradyrhizobium* *daqingense* CCBAU 15774^T^ (HQ231301)

**TUTVUMp5** (MK295028)

**TUTVUMp56** (MK295034)

**TUTVUMp48** (MK295031)

**TUTVUMp50** (MK295032)

**TUTVUMr89** (MK295041)

**TUTVUMp8** (MK295030)

**TUTVUMp53** (MK295033)

**TUTVUMp65** (MK295036)

*Bradyrhizobium* *centrolobii* BR 10245^T^ (KX527991.1)

*Bradyrhizobium* *neotropicale* BR 10247^T^ (KJ661700.1)

**TUTVUMr84** (MK295039)

*Bradyrhizobium* *arachidis* CCBAU 051107^T^ (HM107251)

**TUTVUMm108** (MK295048)

**TUTVUMm110** (MK295049)

**TUTVUI122** (MK295056)

*Bradyrhizobium* *forestalis* INPA54B^T^

*Bradyrhizobium* *subterraneum* 60 2-1^T^ (KM378485.1)

**TUTVUMr94** (MK295043)

**TUTVUMm105** (MK295047)

**TUTVUMp6** (MK295029)

**TUTVUMm112** (MK295050)

**TUTVUMm115** (MK295053)

**TUTVUMm116** (MK295054)

**TUTVUMr103** (MK295046)

**TUTVUMm113** (MK295051)

**TUTVUMm114** (MK295052)

*Bradyrhizobium* *yuanmingense* CCBAU 10071^T^ (AY386780)

**TUTVUMr93** (MK295042)

**TUTVUMr95** (MK295044)

**TUTVUMr85** (MK295040)

**TUTVUMr80** (MK850408)

*Bradyrhizobium* *manausense* BR 3351^T^ (KF785986)

*Bradyrhizobium* *vignae* 7-2^T^ (KM378443)

*Bradyrhizobium* *kavangense* 14-3^T^ (KM378446)

*Bradyrhizobium* *guangdongense* CCBAU 51649^T^ (KC509023)

*Bradyrhizobium* *centrosematis* A9^T^ (KX012940.1)

*Bradyrhizobium* *guangxiense* CCBAU 53363^T^ (KC509033)

*Bradyrhizobium* *erythrophlei* CCBAU 53325^T^ (KF114693)

*Bradyrhizobium* *macuxiense* BR 10303^T^ (KX527995.1)

*Bradyrhizobium* *sacchari* BR 10303^T^ (KX527995.1)

*Bradyrhizobium* *mercantei* SEMIA 6399^T^ (KX690621.1)

**TUTVUMr82** (MK295038)

**TUTVUMr99** (MK295045)

*Bradyrhizobium* *viridifuturi* SEMIA 690^T^ (KR149131)

*Bradyrhizobium* *elkanii* USDA 76^T^ (AY599117)

*Bradyrhizobium* *ripae* WR4^T^ (MF593086.1)

*Bradyrhizobium* *tropiciagri* SEMIA 6148^T^ (FJ391048)

*Bradyrhizobium* *embrapense* SEMIA 6208^T^ (GQ160500)

*Bradyrhizobium* *ferriligni* CCBAU 51502^T^ (KJ818099)

**TUTVUMp60** (MK295035)

**TUTVUMp75** (MK295037)

**TUTVUMm117** (MK295055)

*Bradyrhizobium* *pachyrhizi* PAC48^T^ (FJ428201)

*Bradyrhizobium* *denitrificans* LMG 8443^T^ (HM047121)

*Bradyrhizobium* *oligotrophicum* LMG 10732^T^ (JQ619233)

*Rhizobium* *lusitanum* P1-7^T^ (EF639841.1)

*Bradyrhizobium* *namibiense* 5-10^T^ (KM378440.1)

*Bradyrhizobium* *icense* LMTR 13^T^ (KF896175)

*Bradyrhizobium* *retamae* Ro19^T^ (KC247108)

*Bradyrhizobium* *algeriense* RST89^T^ (FJ264924.1)

*Bradyrhizobium* *valentinum* LmjM3^T^ (JX518575)

*Bradyrhizobium* *lablabi* CCBAU 23086^T^ (GU433498)

*Bradyrhizobium* *jicamae* PAC68^T^ (FJ428204)

*Bradyrhizobium* *paxllaeri* LMTR 21^T^ (KF896169)

99

99

98

98

73

84

99

74

80

87

64

94

90

56

63

91

99

87

69

81

96

99

74

99

80

94

71

99

91

61

92

61

52

88

97

56

99

96

62

84

54

87

98

84

53

54

97

**0.01**

II

IV

V

I

**Fig. S6:** Maximum-likelihood molecular phylogenetic analysis of *glnII* nucleotide sequences of cowpea nodulating rhizobia collected from South Africa and Mozambique. The associated taxa clustered together in the 1000 bootstrap support andpercentage are shown next to the branches. The evolutionary distances were computed using the Kimura 2-parameter method and are in the units of the number of base substitutions per site. The analysis involved 82 nucleotide sequences. Codon positions included were 1st+2nd+3rd+Noncoding. All positions containing gaps and missing data were eliminated. Evolutionary analyses

were conducted in MEGA7

III

IIb

IIa

**Fig. S7:** Maximum-likelihood molecular phylogenetic analysis of *gyrB* nucleotide sequences of cowpea nodulating rhizobia collected from South Africa and Mozambique. The associated taxa clustered together in the 1000 bootstrap support and percentage are shown next to the branches. The evolutionary distances were computed using the Kimura 2-parameter method and are in the units of the number of base substitutions per site. The analysis involved 78 nucleotide sequences. Codon positions included were 1st+2nd+3rd+Noncoding. All positions containing gaps and missing data were eliminated. Evolutionary analyses were conducted in MEGA7.

II

II a

IV

I

III

I a

I b

IV b

IV a

**TUTVUMr80** (MK288817)

**TUTVUMr93** (MK850411)

**TUTVUMr95** (MK288823)

**TUTVUMr85** (MK288820)

*Bradyrhizobium* *manausense* BR 3351^T^ (KF786000.1)

**TUTVUMm105** (MK288826)

**TUTVUMr94** (MK850414)

*Bradyrhizobium* *forestalis* INPA54B^T^ (KF452831.1)

*Bradyrhizobium* *subterraneum* 58 2-1^T^ (KX661396.1)

*Bradyrhizobium* *vignae* 7-2^T^ (KX683216.1)

*Bradyrhizobium* *daqingense* CCBAU 15774^T^ (KF962694)

*Bradyrhizobium* *huanghuaihaiense* CCBAU 23303^T^ (KF962695)

*Bradyrhizobium* *kavangense* 14-3^T^ (KX661397.1)

*Bradyrhizobium* *guangxiense* CCBAU 53363^T^ (KC509082)

**TUTVUMp6** (MK288808)

*Bradyrhizobium* *yuanmingense* LMG 21827^T^ (FM253226.1)

**TUTVUMr103** (MK288825)

**TUTVUMm115** (MK850409)

**TUTVUMm112** (MK288828)

**TUTVUMm113** (MK288829)

**TUTVUMm114** (MK288830)

**TUTVUMm116** (MK850412)

**TUTVUMm110** (MK288827)

**TUTVUMm108** (MK850410)

**TUTVUI122** (MK288831)

*Bradyrhizobium* *arachidis* CCBAU 45332^T^ (JX437674.1)

**TUTVUMr84** (MK288819)

*Bradyrhizobium* *stylosanthis* BR 446^T^ (KU724151.1)

**TUTVUMp48** (MK288810)

*Bradyrhizobium* *guangdongense* CCBAU 51649^T^ (KC509072)

*Bradyrhizobium* *japonicum* LMG 6138^T^ (AM418801.1)

**TUTVUMp53** (MK288812)

**TUTVUMp5** (MK288807)

**TUTVUMp56** (MK288813)

**TUTVUMp8** (MK288809)

**TUTVUMr89** (MK288821)

**TUTVUMp50** (MK288811)

**TUTVUMp65** (MK288815)

*Bradyrhizobium* *liaoningense* LMG 18230^T^ (FM253223.1)

*Bradyrhizobium* *diazoefficiens* USDA 110^T^ (CP011360.1)

*Bradyrhizobium* *ottawaense* OO99^T^ (HQ873179)

*Bradyrhizobium* *betae* LMG 21987^T^ (FM253217.1)

*Bradyrhizobium* *canariense* LMG 22265^T^ (FM253220.1)

*Bradyrhizobium* *rifense* CTAW71^T^ (KC569466)

*Bradyrhizobium* *cytisi* CTAW11^T^ (KF532653)

*Bradyrhizobium* *ganzhouense*^T^ (KP420022)

*Bradyrhizobium* *iriomotense* EK05^T^ (AB300997.1)

*Bradyrhizobium* *ingae* BR 10250^T^ (KF927079)

*Bradyrhizobium* *neotropicale* BR 10247^T^ (KJ661707.1)

*Bradyrhizobium* *centrolobii* BR 10245^T^ (KX528004.1)

*Bradyrhizobium* *macuxiense* BR 10303^T^ (KX528008.1)

*Bradyrhizobium* *sacchari* BR 10303^T^ (KX528008.1)

*Bradyrhizobium* *ripae* WR4^T^ (MF593094.1)

**TUTVUMr82** (MK288818)

**TUTVUMr99** (MK288824)

*Bradyrhizobium* *ferriligni* CCBAU 51502^T^ (KJ818102)

*Bradyrhizobium* *mercantei* SEMIA 6399^T^ (KX690623.1)

*Bradyrhizobium* *tropiciagri* SEMIA 6148^T^ (HQ634890)

*Bradyrhizobium* *viridifuturi* SEMIA 690^T^ (KR149134)

*Bradyrhizobium* *enbrapense* SEMIA 6208^T^ (HQ634891)

*Bradyrhizobium* *elkanii* LMG 6134^T^ (AM418800.1)

*Bradyrhizobium* *pachyrhizi* PAC 48^T^ (HQ873310)

**TUTVUMp60** (MK288814)

**TUTVUMp75** (MK288816)

**TUTVUMm117** (MK850413)

*Bradyrhizobium* *brasilense* UFLA03-321^T^ (KF452827.1)

*Bradyrhizobium* *erythrophlei* CCBAU 53325^T^ (KF114717)

*Bradyrhizobium* *namibiense* 5-10^T^ (KX661393.1)

*Bradyrhizobium* *icense* LMTR 13^T^ (KF896201)

*Bradyrhizobium* *retamae* Ro19^T^ (KF962698)

*Bradyrhizobium* *jicamae* PAC 68^T^ (HQ873309)

*Bradyrhizobium* *lablabi* CCBAU 23086^T^ (KF962696)

*Bradyrhizobium* *paxllaeri* LMTR 21^T^ (KF896195)

*Bradyrhizobium* *denitrificans* LMG 8443^T^ (FM253239.1)

*Bradyrhizobium* *oligotrophicum* LMG 10732^T^ (KF962697.1)

*Bradyrhizobium* *algeriense* RST91 (NZ PYCN01000001)

*Bradyrhizobium* *valentinum* LmjM3 (LLXX01000044)

*Rhizobium* *lusitanum* P1-7^T^ (KC293525.1)

99

98

88

96

83

99

99

99

97

99

86

88

96

73

99

65

76

65

61

99

61

81

92

99

64

54

54

52

86

78

94

87

65

55

62

98

80

73

63

55

51

50

64

**0.05**

II b
